# Novel TaqMan^®^ real-time PCR targeting *invJ* gene for 8-h detection of *Salmonella* from food matrices

**DOI:** 10.3389/fmicb.2025.1517680

**Published:** 2025-07-16

**Authors:** Ann Mary Isaac, Harish Babu Kolla, K. P. Pallavi, Sharat Bandyadka, Chinmayi Nandkishor Mhatre, Radhika Madan Urs, J. Joseph Kingston

**Affiliations:** Defence Institute of Biodefence Technologies (DIBT-DRDO), Mysore, India

**Keywords:** salmonellosis, qPCR, enrichment, novel gene target, artificial contamination

## Abstract

*Salmonella*, a well-known food-borne zoonotic pathogen, is the causative agent of Salmonellosis affecting public health in both developed and developing countries. Traditional *Salmonella* detection methods are time-consuming, involving multiple steps like pre-enrichment, selective plating, and biochemical confirmation. In this study, a faster and sensitive TaqMan^®^ real-time PCR assay was developed with a 6 h enrichment paradigm for the direct detection of *Salmonella* from food matrices using a novel target gene, *invJ*, which is part of the *Salmonella* Type 3 Secretion System (T3SS). The assay was found to be highly specific and had a limit of detection of 10^2^ CFU/mL *Salmonella* in pure culture. With a 6-h enrichment step, the assay was able to detect even low bacterial inoculum like 10^0^ CFU/mL of *S.* Typhimurium in both artificially contaminated raw foods (raw egg and frozen chicken) and cooked Indian food matrices (ready-to-eat chicken biriyani and chicken pulao). The assay demonstrated 100% relative sensitivity, specificity, and accuracy across 52 natural (raw and processed) food samples. In summary, the real-time *Salmonella* detection method developed is faster, specific, sensitive and a potential tool for regular *Salmonella* monitoring in diverse food matrices within 8 h.

## 1 Introduction

The risk of food-borne diseases has been on the rise owing to multiple reasons including fast population growth, pathogen evolution, and increased global trade of foods and farm animals from countries without appropriate microbiological hygiene procedures (NAAS, 2020). *Salmonella* is the etiological agent for the food-borne bacterial zoonotic illness salmonellosis that manifests as gastroenteritis, fever, diarrhea, and serious systemic infections resulting in hospitalization (Gunn et al., 2014; NAAS, 2020). According to CDC (2024a, 2024b), *Salmonella* is among the top three food-borne pathogens in the United States, causing an estimated 1.35 million infections, 26,500 hospitalizations, and 420 deaths annually. In 2022 alone, it was responsible for 13% of all food recalls in the U.S. (Murray, 2023). Among *Salmonella* spp. infections, the nontyphoidal serovars that cause self-limiting infections are highly prevalent among humans and animals (Majowicz et al., 2010).

Approximately 95% of salmonellosis cases result from the consumption of contaminated food, especially meat, poultry, eggs and raw milk (Ehuwa et al., 2021). Animal carcasses may become contaminated with *Salmonella* during slaughter due to contact with hides and/or GI tract materials, insufficiency in animal manure disposal and human related animal processing practices (Sofos, 2008). Factors including insufficient cooking, lack of refrigeration, slow cooling of food and insufficient reheating before serving contribute to the proliferation of *Salmonella* in foods (NAAS, 2020). Similarly, RTE foods such as salad mix (Vestrheim et al., 2016) and poultry meat products (Akbar and Anal, 2015) which are processed and packaged under sublethal temperatures, can harbor viable *Salmonella* cells and have been associated with multiple salmonellosis outbreaks.

Food regulatory agencies generally require the absence of *Salmonella* in 25 g of RTE food (FSSAI, 2011). Well-defined conventional gold standard methods are being followed globally for the detection of *Salmonella* from food matrices. These *Salmonella* detection methods (ISO 6579-1:2017) include sequential steps such as pre-enrichment, selective enrichment, selective and non-selective plating, followed by bacteriological and serological confirmation, which tends to be time-consuming and laborious, with occasional chances of ambiguous results (Schrader et al., 2008).

Detection of food pathogens by quantitative real-time PCR using TaqMan^®^ chemistry is faster and more sensitive than other nucleic acid amplification methods, providing reproducible data in real-time with reduced chances of carry-over contamination (Salihah et al., 2016; Zeng et al., 2016). Over the years, multiple target genes have been used for developing *Salmonella* real-time PCR assays (Rodriguez-Lazaro et al., 2014; Maurischat et al., 2015; Zeng et al., 2016), of which *invA* of type III secretion system (T3SS) within Salmonella Pathogenicity Island-I (SPI-1) is a well-established marker till date (Dmitric et al., 2018). However, extensive research on *invA*-based detection assays have led to few reports describing *invA* polymorphism and its absence among few *Salmonella* strains, potentially complicating accurate identification (Ginocchio et al., 1997; Malorny et al., 2003; Turki et al., 2012). Furthermore, Buehler et al. (2019) demonstrated that some non-*Salmonella enterica* subsp. *enterica* strains were only detectable at concentrations 100,000 times higher than the assay’s limit of detection (LOD), indicating significantly reduced sensitivity.

**Figure 1:**
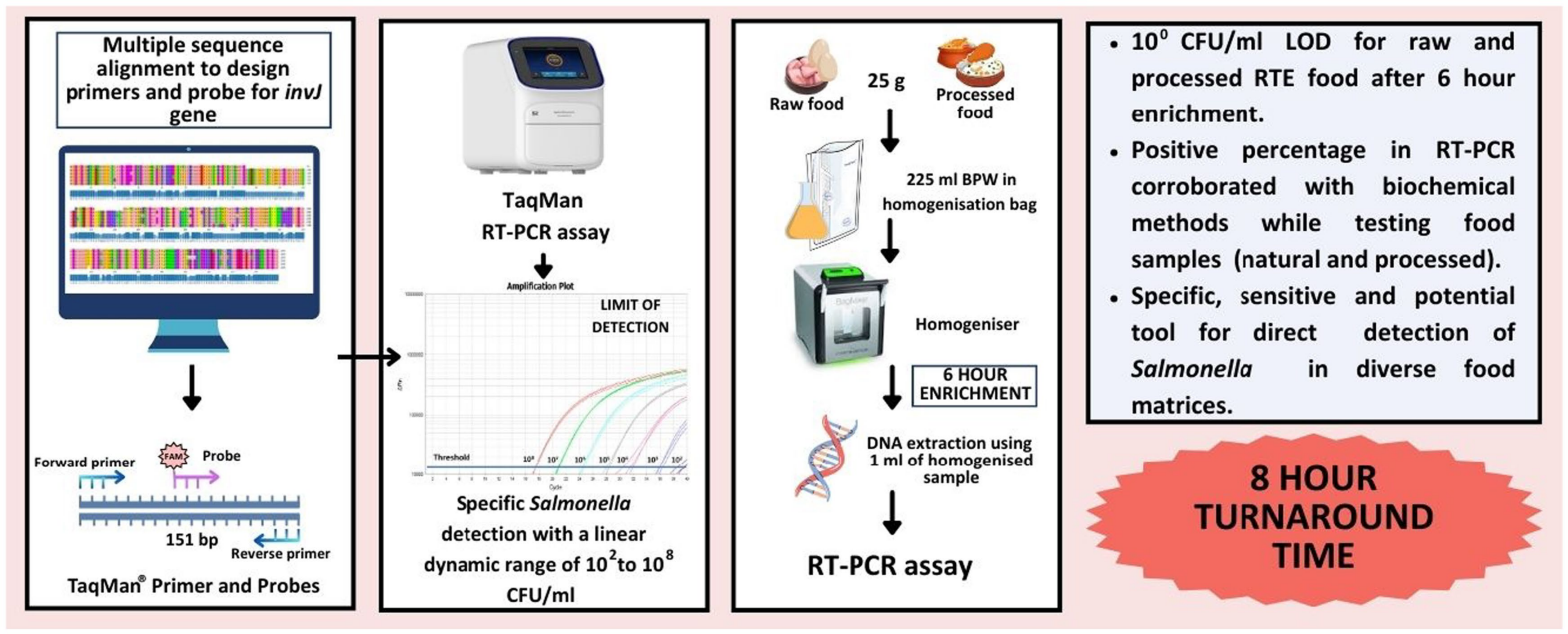
Schematic representation of the *Salmonella invJ* real-time PCR workflow.

The objective of this study was to develop a TaqMan^®^ real-time PCR assay targeting a relatively conserved novel target gene *invJ*, also from the T3SS gene cluster located in the Salmonella Pathogenicity Island-1(Collazo et al., 1995) for faster detection of *Salmonella in* diverse food matrices. Besides inclusivity and exclusivity studies, the limit of detection of the assay was assessed using both raw (egg and frozen chicken) and cooked Indian food samples (chicken biriyani and chicken pulao). A schematic workflow is provided in Figure 1. The performance of this novel method was also assessed against the ISO 6579-1:2017 gold standard for *Salmonella* detection in naturally contaminated samples.

## 2 Materials and methods

### 2.1 Bacterial strains and preparation of thermal lysate

The bacterial strains used in this study were from the culture collection at Defence Institute of Biodefence Technologies, Mysore, India and are listed in Table 1. All bacterial strains (16 *Salmonella* and 18 non-*Salmonella*) used in the inclusivity and exclusivity studies were confirmed biochemically using automated microbial identification system BD Phoenix^™^ M50 (BD Diagnostic Systems, Sparks, MD, United States) before use in the assay. The bacterial strains were maintained as glycerol stocks and cultured overnight in Brain Heart Infusion broth (Himedia, India) with shaking (160 rpm) at 37°C.

**Table 1:** Inclusivity and exclusivity test strains-34 bacterial strains (*Salmonella*: 16; non-*Salmonella*: 18) used for the determination of specificity of the real-time PCR^a^.

No.	Cultures used	Source	Strains	Ct value ± SD
1	*S. enterica* ser Typhimurium	ATCC	ATCC 14028	17.4 ± 0.7
2	*S. enterica* ser Typhimurium	ATCC	ATCC 13311	16.8 ± 0.2
3	*S. enterica* ser Typhimurium	MTCC, India	MTCC 98	17.9 ± 0.5
4	*S. enterica* ser Typhimurium	MTCC	MTCC 1251	19.3 ± 0.1
5	*S. enterica* ser Typhimurium	NICED, Calcutta	BCH 703	17.9 ± 0.1
6	*S. enterica* ser Weltevreden	MTCC	MTCC 1169	19.7 ± 0.1
7	*S. enterica* ser Virchow	MTCC	MTCC 1163	21.6 ± 0.1
8	*S. enterica* ser Bovismorbificans	MTCC	MTCC 1162	20.4 ± 0.2
9	*S. enterica* ser Infantis	MTCC	MTCC 1167	20.7 ± 0.9
10	*S. enterica* ser Bruneii	MTCC	MTCC 1168	18.5 ± 0.2
11	*S. enterica* ser Newport	ATCC	ATCC 6962	19.4 ± 0.1
12	*S. enterica* ser Typhi	ATCC	ATCC 6539	21.5 ± 0.2
13	*S. enterica* ser Paratyphi	MTCC	MTCC 735	20.7 ± 0.5
14	*S. enterica* ser Abony	NCIM	NCIM 2257	18.1 ± 0.1
15	*S. enterica* ser Enteritidis	DIBT	DIBT1	16.7 ± 0.6
16	*S. enterica* subsp. *arizonae*	ATCC	ATCC 13314	21.5 ± 0.1
17	*Escherichia coli*	ATCC	ATCC 10536	—
18	*Yersinia enterocolitica*	NCIM	str. 8081	—
19	*Yersinia enterocolitica*	MTCC	MTCC 840	—
20	*Enterobacter cloacae*	DIBT	DIBT1	—
21	*Enterobacter cloacae*	MTCC	MTCC 12828	—
22	*Citrobacter freundii*	MTCC	MTCC 1658	—
23	*Klebsiella oxytoca*	MTCC	MTCC 3030	—
24	*Klebsiella pneumoniae*	ATCC	ATCC10031	—
25	*Klebsiella pneumoniae*	ATCC	ATCC13883	—
26	*Klebsiella pneumoniae*	MTCC	MTCC 39	—
27	*Serratia marcascens*	MTCC	MTCC 86	—
28	*Proteus mirabilis*	MTCC	MTCC 3310	—
29	*Proteus vulgaris*	ATCC	ATCC33420	—
30	*Shigella boydii*	DIBT	DIBT 1	—
31	*Shigella flexneri*	MTCC	MTCC 1457	—
32	*Providencia alcalifaciens*	ATCC	ATCC51902	—
33	*Listeria monocytogenes*	ATCC	ATCC 15313	—
34	*Staphylococcus aureus*	ATCC	ATCC60079	—

a Ct values ± SD (standard deviations) represented from duplicates.

Viable plate counts of *Salmonella* from overnight culture or artificially spiked food enrichments were obtained by serially diluting the cultures in 1X Phosphate Buffered Saline (PBS) and plating in Salmonella-Shigella (SS) agar (Himedia, India). Following overnight incubation for 12–16 h at 37°C, *Salmonella* appeared as colourless colonies with black centres on SS agar plates.

Thermal lysates were prepared from the bacterial cultures or food enrichments for use in real-time PCR assay. Briefly, 1 mL of overnight culture (12–18 h) or food enrichments were pelleted and resuspended in 300 μL of sterile distilled water, boiled at 100°C for 15 min to lyse the cells, and centrifuged at 10,000 rpm for 5 min to remove cell debris. The supernatant (thermal lysate) was transferred to a new Eppendorf tube. The thermal lysates thus prepared were stored at −20°C and used as template DNA (1 μL) in the real-time PCR assay as described in Section 2.3. The same method for thermal lysate preparation was followed for enriched food samples in Sections 2.7 and 2.9 as well.

### 2.2 Foods used in the study

Artificial contamination experiments were performed with both processed (retort processed, ready-to-eat (RTE) chicken pulao and chicken biriyani) and raw (frozen chicken and egg) foods. RTE chicken pulao and chicken biriyani were acquired from the production facility at Defence Institute of Biodefence Technologies, Mysore, India. Frozen chicken and raw eggs were obtained from local supermarkets in Mysore, India.

A comparative study was conducted to evaluate the efficacy of the *invJ* real-time PCR method against the conventional ISO 6579-1:2017 method for detection of *Salmonella* contamination in natural (raw and processed) food samples. The study encompassed 52 food samples and included raw chicken (*n* = 8), frozen chicken (*n* = 2), raw meat (beef, pork and mutton) (*n* = 17), raw egg (*n* = 5), raw milk (*n* = 10), processed food (*n* = 6) and fresh fruits and vegetables comprising carrots, tomatoes, radish, and lettuce (*n* = 4). All food samples, except the processed ones, were sourced from local supermarkets in Mysore and stored at 4°C until testing. The processed food samples were retort processed and were sourced directly from the DIBT facility.

### 2.3 Analysis of *invJ* gene sequences for TaqMan^®^ probe and degenerate primer design

Complete coding sequence of *invJ* gene was retrieved from the genome of *Salmonella enterica* subsp. *enterica* serovar Typhimurium str. LT2 (Genbank accession ID NC_003197.2:3034342–3035253). Using the above sequence as query, BLAST search was performed and consequently, a total of 122 nucleotide sequences of *Salmonella invJ* gene from various serovars were downloaded from NCBI BLAST^1^ results. The nucleotide sequences were aligned using the multiple sequence alignment program CLUSTAL-W2 using default parameters and the alignments were examined further by alignment explorer (MEGA-11) before phylogenetic analysis. The alignment file was saved with an extension “.mas” for the phylogeny construction. The phylogenetic tree was constructed with MEGA-11 using neighbor-joining method (Saitou and Nei, 1987) with 1,000 bootstrap replicates. The evolutionary distances were computed using the maximum composite likelihood method (Tamura et al., 2004) and are in the units of the number of base substitutions per site. The number of synonymous substitutions per synonymous site (dS) and the number of non-synonymous substitutions per non-synonymous site (dN) averaging over all sequence pairs was calculated with Nei–Gojobori model (Nei and Gojobori, 1986) using MEGA-11.

The multiple sequence alignment was used to design *invJ* oligonucleotide primers with degenerate nucleotide positions and TaqMan^®^ MGB (minor groove binder) probe for use in the *Salmonella* real-time PCR assay. Nucleotide variations in the forward and the reverse primer binding regions among the 122 *invJ* gene sequences from diverse serovars were accounted for, using degenerate bases such as R and Y. Sequences of the primers and probes used for the real-time PCR assay are provided in Table 2. To assess primer specificity, a virtual PCR was conducted using a set of 690 nucleotide sequences derived from BLAST results of the *invJ* gene. This dataset included representative sequences from *Salmonella bongori* (17 strains), *S. enterica* subsp. *salamae* (40), *S. enterica* subsp. *arizonae* (14), *S. enterica* subsp. *diarizonae* (9), and *S. enterica* subsp. *houtenae* (11), using the tool available at https://www.bioinformatics.org/sms2/pcr_products.html. The High Purity Salt Free^®^-purified Primers (Eurofins Genomics, India) and HPLC purified TaqMan^®^ MGB probe (431064, Applied Biosystems^™^, United States) with FAM fluorophore and NFQ-MGB quencher were custom synthesized for the assay.

**Table 2:** Primers and TaqMan probe sequences for specific detection of *Salmonella* using the *invJ* real-time PCR assay.

Primers and probe	Sequences^a^	Length	Tm	Amplicon
*invJ* FW	CARCGCTGGGGAAATGAC	18	57.1°C	151 bp
*invJ* RV	GATCRTCTCGCRYCAGGTG	19	59.9°C	
*invJ* Probe	6 FAM-TACCGTCAAATACGCA-MGB NFQ	16		

In the primer sequences, R indicates A/G while Y indicates C/T.

a FAM, fluorescein amidite; MGB NFQ, minor groove binder-non fluorescent quencher.

### 2.4 Optimization and development of real-time PCR assay

The real-time PCR assay was optimized using *Salmonella enterica* Typhimurium ATCC 14028. The final 10 μL reaction volume comprised of: 1X TaqMan^®^ Mastermix (Premix Eq Taq RR390A, Takara, Japan), 0.5 μM Forward Primer, 0.5 μM Reverse Primer, 0.5 μM TaqMan^®^ MGB Probe and 1 μL of DNA template. Reaction conditions were optimized as: denaturation at 95°C for 30 s, followed by 40 cycles of denaturation at 95°C for 3 s and 57°C for 35 s annealing/extension. Sterile distilled water was used as no-template control for the experiments. The real-time PCR was performed in QuantStudio^™^ 3 Real-Time PCR System (Applied Biosystems, United States). All analyses were performed in QuantStudio^™^ Design and Analysis Software v1.4.3.

### 2.5 Specificity of real-time PCR assay

Specificity of the real-time assay was evaluated with the inclusivity and exclusivity test strains provided in Table 1. Thermal lysate prepared from the all the bacterial strains, as described in Section 2.1, was used as DNA template to determine the specificity of the assay. The assay was carried out as per the optimized protocol in Section 2.4 in duplicates. Sterile distilled water was used as no-template negative control.

### 2.6 Standard curve and limit of detection

Amplification efficiency and limit of detection (LOD) of the real-time PCR assay were determined using 10-fold serial dilutions of *Salmonella enterica* ser. Typhimurium ATCC 14028 bacterial culture ranging from 10^1^–10^8^ CFU/mL, which were confirmed by plating in SS Agar. Thermal lysates prepared from the bacterial culture dilutions using the procedure described in Section 2.1 were used as DNA template. The assay was carried out in triplicates and sterile distilled water was used as no-template negative control. The experiment was repeated independently two times. The standard curve was constructed by plotting mean Ct values against bacterial culture concentrations [expressed in log_10_(CFU/mL)] while amplification efficiency was calculated with the equation *E* = (10^−1/slope^) −1 using the slope of the standard curve.

### 2.7 Limit of detection of real-time PCR in artificially contaminated food samples

Four food types-RTE chicken biriyani, RTE chicken pulao, frozen chicken and raw egg were used for the assessing the LOD of real-time PCR in artificially contaminated food samples as described in Section 2.2. All food samples were confirmed to be *Salmonella*-free by standard microbiological methods. Prior to testing, the frozen chicken was allowed to thaw at refrigeration temperatures for 1 h. The background flora level of the samples was evaluated by Aerobic Plate Count and the results are presented in Supplementary Table 1. Total aerobic counts (TAC) were measured using Petrifilm^™^ Aerobic Count Plates (3 M^™^, United States). Following homogenization of a 10 g food sample in 90 mL of sterile diluent (0.9% NaCl), 1 mL of the homogenate was drawn, serially diluted, and 1 mL of each dilution was inoculated onto Petrifilm^™^ plates according to the manufacturer’s instructions. After incubation at 35°C for 48 h, red colonies were counted to determine the TAC.

For the artificial contamination experiments, the food samples were artificially spiked with *Salmonella* Typhimurium ATCC 14028 such that the net inoculum was 10^0^–10^3^ CFU/mL of food homogenate. Artificially spiked food sample (25 g) incubated overnight at 4°C was aseptically transferred into a homogenizer bag (Nasco Sampling Bag, Himedia, India) and homogenized in 225 mL buffered peptone water (BPW) (Himedia, India) using a lab blender (BagMixer 400, Interscience, France) at 6 strokes/s for 2 min. The homogenates were kept for enrichment at 37°C and shaking (160 rpm). At 6 h, six technical replicates of 1 mL enriched samples were drawn from the homogenates, pelleted at 10,000 rpm for 5 min, and were subsequently prepared for real-time PCR testing. Three samples each were prepared by two different methods-thermal lysis (Section 2.1) and kit-based DNA extraction (Qiagen DNeasy Blood and Tissue Kit, 69506, Germany) with slight modifications. Briefly, the pelleted 1 mL enriched samples were resuspended in 180 μL ATL buffer and 5 μL Proteinase K provided with the kit for lysis. After 1 h, 200 μL of AL buffer was added to the samples and pelleted at 10,000 rpm for 2 min. Absolute ethanol (200 μL) was added to the supernatant followed by addition of the entire mixture to spin column. DNA was eluted in 100 μL of MilliQ water and was used as template for real-time PCR (Section 2.4).

Limit of *Salmonella* detection in various food matrices after 6 h enrichment was obtained from the Ct values of real-time PCR. Viable *Salmonella* count after 6 h enrichment in the spiked samples was enumerated by plating the serial dilutions (1X PBS) in SS agar and used to understand the effect of food matrices in Ct values. The experiment was performed independently three times with the food matrices.

### 2.8 Detection limit of *Salmonella* in the presence of high background flora

Detection limit of *Salmonella* in the presence of natural background flora in food was determined using background flora from frozen chicken. The chicken sample was confirmed to be *Salmonella*-free by standard microbiological methods prior to use for experiments. Chicken sample (25 g) after homogenization in 225 mL BPW, were kept for incubation for 6 h at 37°C with 160 rpm shaking. Chicken homogenates (900 μL) drawn from 6 h enrichment (triplicates) were used as the source of background flora. To each replicate, 100 μL of *Salmonella enterica* ser. Typhimurium ATCC 14028 culture dilutions (10^8^–10^1^ CFU/mL) was added, mixed and pelleted for thermal lysate preparation. The thermal lysate was prepared as per Section 2.1. The supernatant from the thermal lysate was used for real-time PCR for assessing the detection limit of *Salmonella* in the presence of natural background flora from the chicken homogenate. One independent experiment was performed. Total aerobic count of the chicken homogenates at 6 h was assessed using Petrifilm^™^ aerobic count plates as described above in Section 2.7.

### 2.9 Detection of *Salmonella* in natural food samples (raw and processed) using real-time PCR vs. ISO 6579-1:2017 standard

Presence of *Salmonella* in 52 natural food samples (raw and processed) (Section 2.2) was examined by the real-time PCR assay and compared with the ISO 6579-1:2017 method of *Salmonella* detection. For both methods of detection, 25 g of food sample was homogenized with 225 mL of buffered peptone water (BPW). The samples were enriched at 37°C with shaking at 160 rpm. At 6 h, 1 mL enriched samples were drawn in triplicate for real-time PCR analysis. Thermal lysate was prepared from these drawn samples as described in Section 2.1, and was subsequently analysed by real-time PCR.

Following sample collection for real-time PCR, the remaining enrichment cultures were incubated overnight to proceed with detection according to the standard ISO 6579-1:2017 protocol. This method was followed exactly up to the selective plating step, where samples were streaked onto Xylose Lysine Deoxycholate (XLD) agar and Hektoen Enteric (HE) agar. Presumptive colonies in XLD and HE agar were then sub-cultured onto non-selective Brain Heart Infusion (BHI) agar and incubated overnight at 37°C. The resulting colonies were subjected to automated biochemical identification using the BD Phoenix^™^ M50 system with NID panels, following the manufacturer’s instructions.

### 2.10 Statistical analysis

The real-time PCR data obtained from the instrument were analyzed using QuantStudio Design and Analysis Software v1.4.3. The cycle threshold (Ct) values of amplification were determined by the software. Construction of standard curve and other data analysis was done using Microsoft Excel, 2007. Relative sensitivity, specificity, and diagnostic accuracy describe the ability of the PCR method for detection of analyte as compared to the reference method. The relative sensitivity, specificity, and accuracy were calculated as described:


Relative sensitivity=(TP/(TP+FN))×100

Relative specificity=(TN/(TN+FP))×100

Relative accuracy=((TP+TN)/(TP+TN+FP+FN))×100

where TP, true positive, FP, false positive, TN, true negative, FN, false negative as compared to the ISO 6579-1:2017 method.

## 3 Results

### 3.1 *invJ* gene is conserved in *Salmonella* serovars and is a potential target for detection

BLAST analysis of the *invJ* gene sequence (Genbank accession ID NC_003197.2:3034342–3035253) with the default parameters showed hits with other *Salmonella invJ* nucleotide sequences with 84 to 100% sequence identity and 100% query coverage. The BLAST hits with other *Enterobacteriaceae* strains showed less than 15% query coverage indicating the absence of *invJ* homologs in *Enterobacteriaceae* strains. Multiple sequence alignment (MSA) of the 122 *invJ* gene nucleotide sequences from *Salmonella* strains obtained from NCBI was used to determine the conserved nature of the *invJ* gene among *Salmonella* strains. Among the 122 sequences that were analyzed, the *Salmonella invJ* gene was found to have 676 conserved sites and 335 variable polymorphic sites (289 parsimonious informative sites) that resulted in 195 conserved, and 141 variable amino acid polymorphic sites (121 Pi sites). The type of selection acting on *Salmonella invJ* gene was understood using dN/dS values. The dN-dS was observed to be −0.072, while dN/dS ratio was observed as 0.27 which is indicative of purifying selection. The rate of non-synonymous substitution (dN −0.027) was 3.7-fold lesser than of synonymous substitution (dS −0.099). This evolutionarily conserved nature of *Salmonella invJ* gene qualified it as a viable molecular target for specific detection of *Salmonella* in food samples.

**Figure 2:**
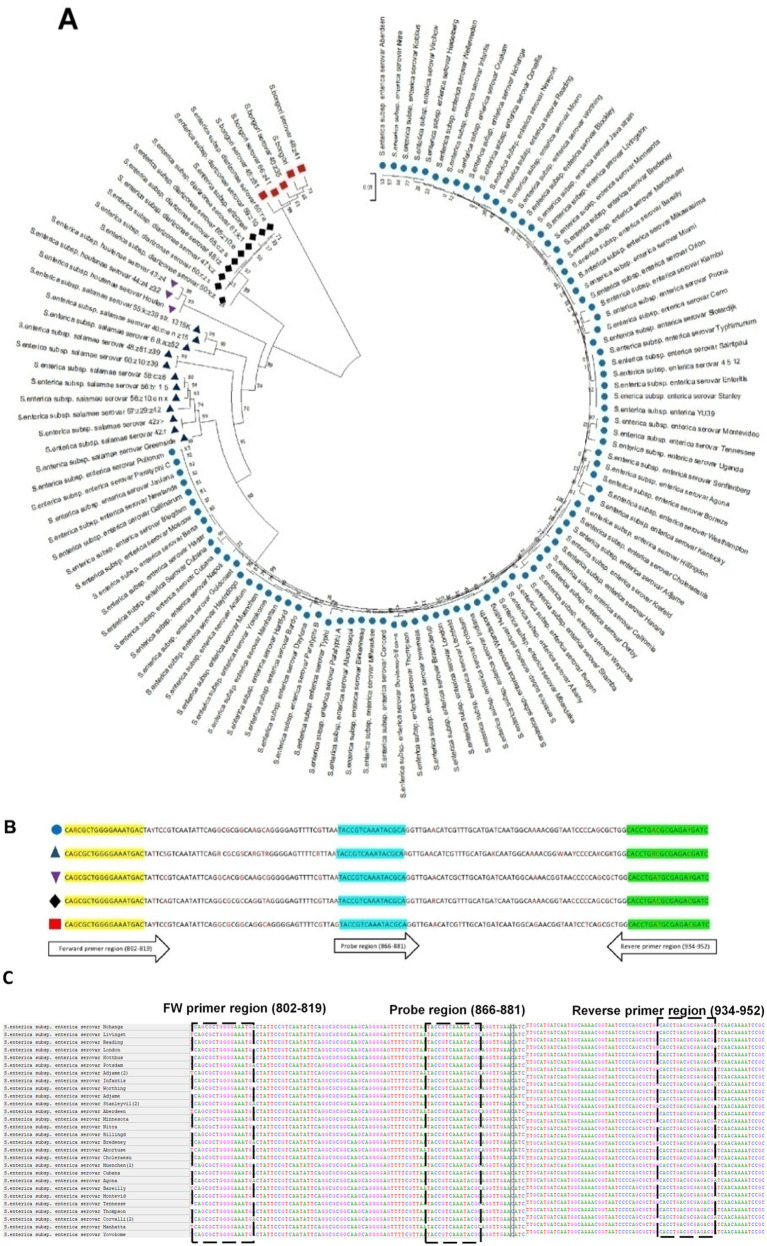
**(A)** Phylogenetic tree of *invJ* gene constructed using multiple sequence alignment of *invJ* gene sequences obtained from 92 *Salmonella* strains from NCBI. *Salmonella bongori* (red square), *Salmonella enterica* subsp. *enterica* (blue circle), *Salmonella enterica* subsp. *arizonae* and *diarizonae* (black diamond), *Salmonella enterica* subsp. *salamae* (dark blue triangle), *Salmonella enterica* subsp. *houtenae* (purple inverted triangle). **(B)** Multiple sequence alignment (MSA) of *Salmonella* subspecies *invJ* sequences indicating location of the forward primer, reverse primer and probe with coloured symbols indicated above. **(C)** Multiple sequence alignment of *Salmonella invJ* gene from representative serovars indicating location of primers (forward: 802–819, reverse: 934–952) and probe (866–881).

Additionally, phylogenetic analysis of *invJ* by MEGA-11 revealed a phylogenetic tree with five clusters based on the species and subspecies variation in *Salmonella* which has been highlighted in Figures 2A,B using multi-colored symbols.

### 3.2 Analysis of *invJ* gene sequences for TaqMan^®^ probe and degenerate primer design

Degenerate real-time PCR primers and the TaqMan^®^ probe for the specific detection of *Salmonella* were designed targeting the conserved regions identified by multiple sequence alignment of *Salmonella invJ* gene sequences. The forward, reverse primers and probe were derived from three conserved regions viz. i.e., 802–819 bp, 934–952 bp and 866–881 bp, respectively, to provide an amplicon of 151 bp and spanning 802 bp to 952 bp region of *Salmonella invJ* gene (Figure 2C). The highly conserved 866–881 region without any nucleotide variability among serovars, was used for designing the TaqMan^®^ MGB probe. To sum up, the primers FW: 5′-CARCGCTGGGGAAATGAC-3′, RV: 5′-GATCRTCTCGCRYCAGGTG-3′, and the *invJ* gene specific probe 6 FAM-TACCGTCAAATACGCA-MGB NFQ were designed, custom synthesized and used for optimizing the real-time PCR assay conditions for the specific detection of *Salmonella* in food samples. Virtual PCR results indicate that all assessed 690 *Salmonella invJ* sequences, including *S. bongori*, *S. enterica* subsp. *enterica*, *S. enterica* subsp. *houtenae*, *S. enterica* subsp. *arizonae*, *S. enterica* subsp. *diarizonae* and *S. enterica* subsp. *salamae* (Supplementary Data), showed an amplicon of 151 bp.

### 3.3 Specificity of real-time PCR assay

**Figure 3:**
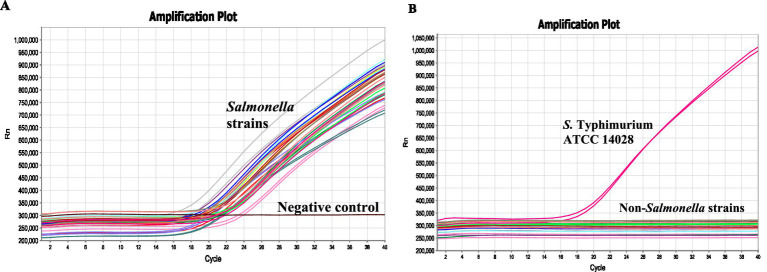
Specificity of the real-time PCR assay determined using **(A)** *Salmonella* serovars (1–16), **(B)** and non-*Salmonella* strains (17–34) as indicated in Table 1. No-template control indicates negative control where sterile distilled water was used in the reaction.

Specificity of the *invJ* real-time PCR involving primers and probe designed in this study was determined using 34 bacterial test strains [*Salmonella*: 16; non-*Salmonella*: 18 (Table 1)]. Amplification was observed from all the *Salmonella* strains with Ct values ranging from 16.7 to 21.6 for the *invJ* target while Ct values of *S.* Typhimurium strains ranged from 16.8 to 19.3 in the real-time PCR assay (Table 1 and Figure 3A). No amplification was observed for the non-*Salmonella* strains used in the assay (Figure 3B). The specificity studies confirmed that only *Salmonella* strains were detected by the assay and no cross-reactivity was observed with non-*Salmonella* strains used in the study.

### 3.4 Standard curve and limit of detection

**Figure 4:**
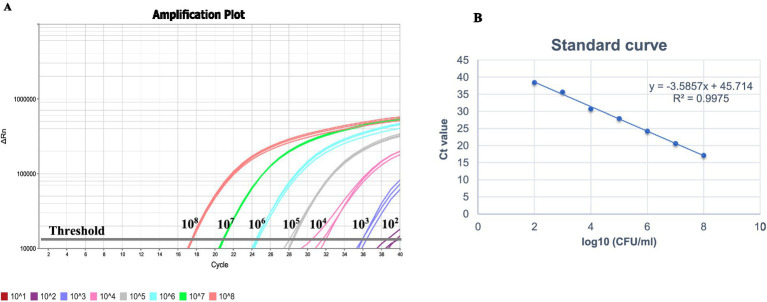
**(A)** Limit of detection of the real-time PCR assay using 10-fold logarithmic dilutions (10^1^–10^8^ CFU/mL) of overnight *Salmonella* Typhimurium ATCC 14028. **(B)** Standard curve obtained by plotting mean Ct values against *Salmonella* culture dilutions (10^1^–10^8^ CFU/mL). Thermal lysates prepared from the culture dilutions was used as DNA template.

The analytical limit of *Salmonella* detection by the real-time PCR method with respect to CFU/mL count was determined using logarithmic dilutions of *S.* Typhimurium (ATCC 14028). A linear relationship was observed over the range of 10^2^ to 10^8^ CFU/mL with Ct values ranging from 17.2 to 38.4 (Figure 4A and Table 3). The resulting equation for the straight line was −3.5857x + 45.714, which showed a good correlation between Ct values and Log_10_ CFU/mL with an *R*^2^ value of 0.9975 (Figure 4B). Results demonstrated that the LOD was 10^2^ CFU/mL of *Salmonella*. The efficiency of amplification determined using the slope of the standard curve was found to be 90%.

**Table 3:** Determination of limit of detection of *Salmonella* real-time PCR assay using 10-fold logarithmic dilutions (10^1^–10^8^ CFU/mL) of overnight *Salmonella* Typhimurium ATCC 14028^a^.

No.	*Salmonella* count (CFU/mL)	Ct value ± SD
1	10^8^	17.2 ± 0.06
2	10^7^	20.6 ± 0.06
3	10^6^	24.2 ± 0.12
4	10^5^	27.9 ± 0.28
5	10^4^	30.8 ± 1.00
6	10^3^	35.7 ± 0.24
7	10^2^	38.4 ± 0.65
8	10^1^	—

a Ct values ± SD (standard deviations) represented from three triplicates per experiment for two independent experiments.

### 3.5 Limit of detection of real-time PCR in artificially contaminated food samples

The limit of direct *Salmonella* detection (LOD) from food matrices was determined using 6 h enrichments obtained by spiking *Salmonella* Typhimurium ATCC 14028 in food (initial inoculum—10^0^–10^3^ CFU/mL). Irrespective of the food matrices used, *Salmonella* could be detected from all the culture dilution enrichments with detection probability 1 (100%) even at the lowest level of initial inoculum (10^0^ CFU/mL).

In food homogenates viz. egg, frozen chicken, RTE chicken biriyani, and RTE chicken pulao having an initial inoculum of 10^3^ CFU/mL, the 6 h enrichment yielded Ct values of 15.6, 23.7, 15.4, and 14.6, respectively using kit extracted DNA (Table 4). The Ct values for other 6 h enrichments are shown in Table 4. No amplification was observed in the no-template control. It is observable that thermal lysate derived DNA showed a higher Ct value than kit derived DNA (Table 4). In all cases, the replicates showed uniform detection leading to a probability of detection (p/n) value equal to 1. The *Salmonella* CFU counts after 6 h enrichment was determined by plating in SS agar. At the end of 6 h of enrichment, ~10^5^ CFU/mL *Salmonella* was observed in all food matrices that were initially spiked with the lowest 10^0^ CFU/mL of food, except RTE chicken biriyani. Alternatively, 10^3^ CFU/mL spiking multiplied to 10^8^ CFU/mL (or 10^7^ CFU/mL for RTE chicken biriyani). Collectively, at the end of 6 h enrichment the CFU/mL values ranged from 10^5^–10^8^ CFU/mL for the initial inoculum levels of 10^0^–10^3^ CFU/mL, respectively, in all food matrices except in RTE chicken biriyani (Table 4). RTE chicken biriyani had the largest dynamic range of Ct values ranging from 15.4 to 27.6 for kit derived DNA as well as 19.1 to 32.4 for thermal lysate derived DNA, as compared to other food matrices (Table 4C).

**Table 4:** Limit of detection of real-time PCR in artificially contaminated food samples^a^.

(A) Frozen chicken					
No.	Initial inoculum (CFU/mL)	VBC at 6 h (CFU/mL)	Ct values ± SD (kit)	Ct values ± SD (thermal lysate)	p/n
1	10^3^	6.4 × 10^8^	23.7 ± 1.7	27.4 ± 0.0	1
2	10^2^	2.4 × 10^7^	25.2 ± 0.3	28.1 ± 0.7	1
3	10^1^	1.1 × 10^6^	29.4 ± 0.6	34.5 ± 0.6	1
4	10^0^	1.1 × 10^5^	33.5 ± 0.5	39.1 ± 0.0	1

(B) Raw egg					
No.	Initial inoculum (CFU/mL)	VBC at 6 h (CFU/mL)	Ct values ± SD (kit)	Ct values ± SD (thermal lysate)	p/n
1	10^3^	8.4 × 10^8^	15.6 ± 0.1	21.5 ± 0.7	1
2	10^2^	1.7 × 10^7^	19.4 ± 1.2	26.2 ± 0.3	1
3	10^1^	4.2 × 10^6^	21.9 ± 0.8	29 ± 0.5	1
4	10^0^	3 × 10^5^	23.8 ± 0.4	31.9 ± 0.5	1

(C) RTE chicken biriyani					
No.	Initial inoculum (CFU/mL)	VBC at 6 h (CFU/mL)	Ct values ± SD (kit)	Ct values ± SD (thermal lysate)	p/n
1	10^3^	5.1 × 10^7^	15.4 ± 0.4	19.1 ± 0.1	1
2	10^2^	4.3 × 10^6^	17.9 ± 0.3	20.7 ± 0.1	1
3	10^1^	3.3 × 10^5^	20.7 ± 0.5	24.6 ± 0.2	1
4	10^0^	1.3 × 10^4^	27.6 ± 0.1	32.4 ± 0.6	1

(D) RTE chicken pulao					
No.	Initial inoculum (CFU/mL)	VBC at 6 h (CFU/mL)	Ct values ± SD (kit)	Ct values ± SD (thermal lysate)	p/n
1	10^3^	1.4 × 10^8^	14.6 ± 0.2	16.3 ± 0.4	1
2	10^2^	1.6 × 10^7^	17.8 ± 0.3	19.1 ± 0.8	1
3	10^1^	3.6 × 10^6^	20.5 ± 0.3	22.3 ± 1.8	1
4	10^0^	2.5 × 10^5^	22.7 ± 0.2	28.3 ± 1.4	1

Food homogenates from four different food matrices-frozen chicken, raw egg, RTE chicken biriyani and RTE chicken pulao (A–D) were artificially contaminated with *Salmonella* (10^0^ to 10^3^ CFU/mL) and viable bacterial count (VBC) (CFU/mL) was recorded after 6 h enrichment. DNA obtained using thermal lysis and kit-based extractions were used in real-time PCR assay and Ct values ± SD recorded.

a All values are recorded as mean value ± standard deviation (*n* = 6) from one representative experiment. p/n, probability of detection where p, no of positive replicates, and n, total no of replicates.

### 3.6 Detection of *Salmonella* in the presence of high background flora

**Figure 5:**
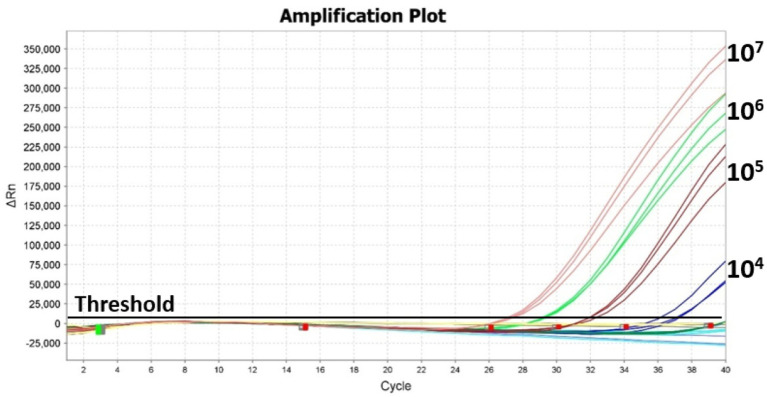
Effect of background flora on limit of detection of *Salmonella* using real-time PCR. Chicken homogenate drawn after 6 h enrichment was used as the source of background flora. Amplification plot from real-time PCR of *Salmonella* culture dilutions (10^8^–10^1^ CFU/mL) mixed with background flora of 9 × 10^7^ CFU/mL.

The limit of *Salmonella* detection by real-time PCR in the presence of background flora was determined by mixing *Salmonella* Typhimurium ATCC 14028 culture dilutions (10^0^–10^7^ CFU/mL) with 6 h enrichment culture obtained from frozen chicken (Figure 5). The total aerobic plate count from the 6 h enrichment culture was 9 × 10^7^ CFU/mL (Table 5). In the presence of this high background flora, the LOD was estimated at 10^4^ CFU/mL with Ct values ranging from 27.1 to 36.6 for 10^7^ to 10^4^ CFU/mL *Salmonella* culture dilutions (Table 5) using thermal lysate derived DNA. No Ct value was observed in the No-template control. It was also observed that the linearity of the Ct values is affected by the presence of high background flora level as indicated by the standard curve that yielded an *R*^2^ value of 0.51 (Supplementary Data).

**Table 5:** Effect of background flora on limit of detection of *Salmonella* using real-time PCR.

Net *Salmonella* concentration (CFU/mL)	Mean Ct value^a^
10^7^	27.1 ± 0.2
10^6^	29.1 ± 0.1
10^5^	31.9 ± 0.2
10^4^	36.6 ± 0.6
10^3^	—
10^2^	—
10^1^	—
10^0^	—

Chicken homogenate drawn after 6 h enrichment was used as the source of background flora (9 × 10^7^ CFU/mL). Mean Ct values obtained by real-time PCR detection of *Salmonella* culture dilutions (10^8^–10^1^ CFU/mL) chicken-derived background flora.

a All values are recorded as mean value ± standard deviation (*n* = 3).

### 3.7 Detection of *Salmonella* in natural food samples (raw and processed) using real-time PCR vs. ISO 6579-1:2017 standard

A total of 52 natural food samples (raw and processed) were analyzed by the real-time PCR assay and it was compared with ISO 6579-1:2017 for *Salmonella* detection. Out of 52 samples, 14 samples were found to be positive for *Salmonella* by real-time PCR assay after 6 h enrichment (Section 2.8). Similar results were observed with the automated microbial identification as well (Table 6). Raw egg and raw meat yielded a *Salmonella* positivity rate of 80 and 64.7% respectively, followed by raw milk and raw chicken (40 and 37.5%), which was corroborated by the automated microbial identification (Table 6). Processed food, fresh vegetables and frozen chicken was found to be negative for *Salmonella*. It is to be noted that the selective agar plating step in the ISO 6579-1:2017 method yielded *Salmonella*-like colonies in 100% of raw chicken, 50% of frozen chicken, 88.2% of raw meat, and 100% of fresh fruits and vegetables. However, the subsequent automated microbial identification step provided the actual result revealing 37.5, 0, 64.7, and 0% *Salmonella* contamination, respectively, in the samples (Table 6). The real-time PCR assay was found to have 100% relative specificity, 100% relative sensitivity and 100% relative accuracy as shown in Table 7.

**Table 6:** Detection of *Salmonella* in 52 natural food samples (raw and processed) using real-time PCR vs. ISO 6579-1:2017.

No.	Samples	Real-time-PCR positivity %	Positivity under ISO 6579-1:2017 (%)	
			Post selective agar plating as per ISO 6579-1:2017^a^	Confirmation as *Salmonella* by automated microbial identification^b^
1	Raw chicken (*n* = 8)	37.5 (3/8)	100 (8/8)	37.5 (3/8)
2	Frozen chicken (*n* = 2)	0 (0/2)	50 (1/2)	0 (0/2)
3	Raw meat (*n* = 17)	64.7 (11/17)	88.2 (15/17)	64.7 (11/17)
4	Egg (*n* = 5)	80 (4/5)	80 (4/5)	80 (4/5)
5	Raw milk (*n* = 10)	40 (4/10)	40 (4/10)	40 (4/10)
6	Processed food (*n* = 6)	0 (0/6)	0 (0/6)	0 (0/6)
7	Fresh vegetables and fruits (*n* = 4)	0 (0/4)	100 (4/4)	0 (0/4)

a Positivity (%) calculated from presumptive *Salmonella*-like colonies (colourless black-centred colonies) obtained from various natural and processed food samples.

b Positivity (%) calculated post automated microbial identification with BD Phoenix^™^ M50 from the presumptive *Salmonella* colonies.

**Table 7:** Detection of *Salmonella* in 52 natural food samples (raw and processed) using real-time PCR vs. ISO 6579-1:2017.

Foods	Total No. of samples	ISO 6579-1:2017 method		Real-time PCR		Relative sensitivity^a^ (%)	Relative specificity^b^ (%)	Relative accuracy^c^ (%)
		No. of +ve samples	No. of –ve samples	No. of FN samples	No. of FP samples			
Raw chicken	8	3	5	0	0	100	100	100
Frozen chicken	2	0	2	0	0	N.A.^d^	100	100
Raw meat	17	11	6	0	0	100	100	100
Egg	5	4	1	0	0	100	100	100
Raw milk	10	4	6	0	0	100	100	100
Processed food	6	0	6	0	0	N.A.	100	100
Fresh vegetables	4	0	4	0	0	N.A.	100	100
Total	52	14	23	0	0	100	100	100

a Relative sensitivity = (TP/(TP + FN)) × 100,

b Relative specificity = (TN/(TN + FP)) × 100,

c Relative accuracy = ((TP + TN)/(TP + TN + FP + FN)) × 100, where TP, true positive; FP, false positive; TN, true negative; FN, false negative as compared to the ISO 6579-1:2017 method.

d N.A.—Data not applicable.

## 4 Discussion

Rapid and reliable detection of *Salmonella* from food matrices is of great significance as food safety regulations mandate the absence of *Salmonella* in 25 g food (FSSAI, 2011). To meet this requirement, food homogenates (final volume: 250 mL) are typically subjected to overnight enrichment, allowing bacterial levels to reach the threshold detectable in a 1 mL aliquot used for analysis (ISO 6579-1:2017). However, this enrichment step represents a major bottleneck for same-day detection, even when employing advanced methods such as real-time PCR, which are significantly faster and more robust than conventional approaches like the ISO 6579-1:2017 method that typically requires 4 to 5 days for confirmation. In order to detect prominent food borne *Salmonella* serovars, use of a highly conserved *Salmonella* specific marker in a sensitive detection platform is of prime importance.

Here, a novel *Salmonella* real-time PCR assay targeting the *Salmonella invJ* gene was designed. The gene is part of *Salmonella* T3SS located in *Salmonella* Pathogenicity Island and the 36.4 kDa InvJ protein (Collazo et al., 1995) forms an integral component of infection needle complex playing important role in the infection process by regulating the length of the needle segment (Kubori et al., 2000) acting as a secreted molecular ruler (Wee and Hughes, 2015). It is essential for effector protein secretion and for invasion into epithelial cells (Rüssmann et al., 2002). BLAST search using the *Salmonella enterica* subsp. *enterica* serovar Typhimurium str. LT2 Genbank accession ID NC_003197.2:3034342–3035253 as a probe revealed the distribution of *invJ* gene homologs among *Salmonella* serotypes and its absence in other bacterial species including *Enterobacteriaceae* members in NCBI database, qualifying *invJ* as an ideal target for designing primers and probe for TaqMan^®^ real-time PCR assay. The *invJ* gene homologs (122 sequences) identified by BLAST search were retrieved from NCBI and the multiple sequence alignment revealed that the gene underwent purifying selection with a dN/dS ratio of 0.27 (<1) indicating that *invJ* is evolutionarily conserved. Additionally, virtual PCR showing the amplification of both *Salmonella* species (*enterica* and *bongori*) as well as all five *Salmonella* subspecies (except *S. enterica* subsp. *indica*, where data was not retrievable) further qualifies *invJ* as a potential gene target for *Salmonella* detection. The designed DNA probe targeting *invJ* region conserved across all the sequences and degenerate primers flanking the probe were used in optimizing the real-time PCR that specifically detected all *Salmonella* strains used in the study (Table 2).

The limit of detection of *Salmonella* Typhimurium pure culture in PBS was found to be 10^2^ CFU/mL. Alternatively, the possibility for direct detection from food matrices with enrichment was examined by spiking *Salmonella* in food (10^0^ to 10^3^ CFU/mL). It is known that processed food matrices are a heterogenous combination of multiple components including oil, herbs, spices, inorganic particles, preservatives, biochemical compounds while raw food have indigenous microflora that bring about some degree of inhibition in the detection sensitivity of real time PCR (Wang and Salazar, 2016). The influence of complex food matrices in the detection limit of the assay could be through the enrichment rates of microorganisms and/or due to presence of PCR inhibitors/background flora (Wilson, 1997; Schrader et al., 2012). High food processing temperature used especially in Indian food also affects the DNA quality available for detection assays (Ahmed et al., 2018).

In artificially spiked food matrices, raw (frozen chicken, egg) and processed (RTE chicken pulao and RTE chicken biriyani), the assay showed a detection limit of 10^0^ CFU/mL with 6 h enrichment. The lowest *Salmonella* inoculum 10^0^ CFU/mL increased up to 10^4^ CFU/mL in RTE chicken biriyani and 10^5^ CFU/mL in egg, frozen chicken and RTE chicken pulao homogenates and the Ct values occurred well within reasonable range after 6 h incubation. The dynamic Ct value range of 27.6 to 15.4 and 22.7 to 14.6 was observed for kit extracted DNA from the food homogenates RTE chicken biriyani and RTE chicken pulao, respectively, that were spiked with 10^0^ to 10^3^ CFU/mL *Salmonella* (Table 4). The one log lower enrichment of *Salmonella* makes us speculate that higher level of spices in the former had an inhibitory role in the bacterial enrichment and thus resulted in higher Ct values (Table 4). Even though both raw egg, chicken as well as RTE chicken pulao homogenates with 10^0^–10^3^ CFU/mL exhibited similar *Salmonella* CFU counts at the end of 6 h (Table 4), their dynamic range of Ct values (kit-DNA) varied from 23.8 to 15.6, 33.5 to 23.7 and 22.7 to 14.6, respectively. Here frozen chicken had a background total aerobic count of 2.5 × 10^2^ CFU/mL (Supplementary Table 1) and though the presence of this background flora did not affect the enrichment of *Salmonella* (Table 4), it resulted in higher dynamic Ct values for the frozen chicken enrichment (33.5 to 23.7) (Table 4) when compared to the raw egg enrichments without the background flora (Table 4). This demonstrated the effect of matrix inhibition observed in raw chicken which is possibly because of the background flora content (Supplementary Table 1) as corroborated by the lower detection sensitivity/higher Ct value observed in the background flora experiments (Figure 5 and Table 5). It is also observable that the purified DNA obtained by kit-based sample preparation yielded improved Ct values as compared to the thermal lysate prepared from the enrichments (Table 4).

The real-time PCR assay was compared with the ISO 6579-1:2017 for detection of *Salmonella* from natural (processed and raw) food samples. The real-time PCR showed 100% relative specificity, 100% relative sensitivity and 100% relative accuracy in *Salmonella* detection with respect to the natural food samples (raw and processed) tested. None of the processed food was found to contain *Salmonella* while certain natural samples showed *Salmonella* content by real-time PCR which was confirmed as described in Section 3.7. In culture-based *Salmonella* detection approaches such as ISO/BAM methods, presumptive colonies for subsequent biochemical tests are chosen based on their morphology from selective agar plates. We observed *Salmonella*-like presumptive colonies in all raw chicken and fresh fruit/vegetable samples and large percentage of frozen chicken, raw meat samples during the initial selective agar plating on Xylose Lysine Deoxycholate (XLD) agar and Hektoen Enteric Agar (HE) agar that were subsequently identified as *Proteus* spp., *Citrobacter* spp., *Enterobacter* spp. through automated microbial identification (Data not shown). As only limited number of colonies are chosen for biochemical analysis (≥2) by culture-based *Salmonella* detection methods from food matrices, similarity in colony morphology of *Salmonella* with the above bacterial species leads to a scenario of choosing *Salmonella*-mimicking colonies that are present in abundance in the selective agar plates, resulting in false negatives (Manafi, 2000; Pławińska-Czarnak et al., 2021). Since occasional similarity is observed in the biochemical properties of *Salmonella*, *Citrobacter* and *Proteus*, studies indicate that 24% of the isolates previously identified presumptively as *Salmonella* due to biochemical characterization was found to be genetically identified as *Citrobacter* spp., while 16.4% of the colonies was observed to be *Proteus* spp. pointing to the insufficiency of biochemical tests in identifying *Salmonella* (Turki et al., 2013).

The real-time PCR assay described here could specifically identify *Salmonella* when present in the enrichments irrespective of predominance of *Proteus*, *Citrobacter* etc. with a turnaround time of less than 8 h against the 3–5 day workflow in standard methods. Our method is comparable to reports by Zheng et al. (2016) who demonstrated a method for detection of viable *Salmonella* cells in mung bean sprouts, requiring a minimum enrichment time of 8 h and employing real-time PCR with immunomagnetic separation or centrifugation. Our approach achieves a comparable LOD in 6 h enrichment without the need for additional concentration steps such as immunomagnetic separation, thereby offering a more practical alternative for routine use in food testing laboratories.

Future studies could focus on evaluating the applicability of the assay across a wider range of food types and in larger field studies conducted in diverse geographic regions. Additionally, incorporating an internal amplification control (IAC) into the assay would help identify potential false-negative results caused by inhibitors present in various food matrices.

## 5 Conclusion

In summary, the real-time PCR assay developed using a novel target gene *invJ* could specifically detect *Salmonella* from food matrices in a shorter time frame of about 8 h without cross reactivity. The assay showed a limit of detection of 10^2^ CFU/mL for *Salmonella* pure culture and 10^0^ CFU/mL for artificially spiked food matrices (frozen chicken, egg, RTE chicken pulao, RTE chicken biriyani) using 6 h enrichment. The assay was found to have 100% relative sensitivity, 100% relative specificity as well as 100% relative accuracy for the detection of *Salmonella* when it was evaluated using 52 natural raw as well as processed food samples. The assay would be a useful tool for regular *Salmonella* screening from diverse food matrices with additional validation studies.

## Data Availability

The original contributions presented in the study are included in the article/Supplementary material, further inquiries can be directed to the corresponding author.

## Footnotes

^1^ https://www.ncbi.nlm.nih.gov/
