# Supplementary material for: Novel TaqMan® real-time PCR targeting invJ gene for 8-h detection of Salmonella from food matrices
Source: Front Microbiol. 2025 Jul 16;16:1517680. doi: 10.3389/fmicb.2025.1517680 (PMC12307383; doi:10.3389/fmicb.2025.1517680)
Supplement: Supplementary file 1 [file Table_1.docx]

**Supplementary Data**

**Table 1. Aerobic Plate count (APC) values of food homogenates from the various food matrices used for artificial spiking studies.**

| **No** | **Sample** | **Aerobic Plate Count (APC) (CFU/ml)** |
| --- | --- | --- |
| 1 | Egg | - |
| 2 | RTE Chicken Pulao | - |
| 3 | RTE Chicken Biriyani | - |
| 4 | Raw chicken | 2.5 x 10^2^ |

**Figure 1. Standard curve generatd by plotting mean Ct values against net *Salmonella* concentrations to evaluate the effect of background flora on limit of detection of *Salmonella* using real-time PCR**

**In silico PCR analysis**

**Tool used:**

<https://www.bioinformatics.org/sms2/pcr_products.html>

**Sequences used:** All the fasta sequences of *Salmonella* that were included in the manuscript/bioinformatics analysis mined through the BLAST.

**Primers used:** Primer sequences that are included in the manuscript.

PCR Products results

>151 bp product from linear template S.enterica subsp. enterica serovar Typhimurium, base 802 to base 952 (FW - RV).

CAGCGCTGGGGAAATGACTATTCCGTCAATATTCAGGCGCGGCAAGCAGGGGAGTTTTCG

TTAATACCGTCAAATACGCAGGTTGAACATCGTTTGCATGATCAATGGCAAAACGGTAAT

CCCCAGCGCTGGCACCTGACGCGAGACGATC

>151 bp product from linear template S.enterica subsp. enterica serovar Saintpaul, base 802 to base 952 (FW - RV).

CAGCGCTGGGGAAATGACTATTCCGTCAATATTCAGGCGCGGCAAGCAGGGGAGTTTTCG

TTAATACCGTCAAATACGCAGGTTGAACATCGTTTGCATGATCAATGGCAAAACGGTAAT

CCCCAGCGCTGGCACCTGACGCGAGACGATC

>151 bp product from linear template S.enterica subsp. enterica serovar 4 5 12, base 802 to base 952 (FW - RV).

CAGCGCTGGGGAAATGACTATTCCGTCAATATTCAGGCGCGGCAAGCAGGGGAGTTTTCG

TTAATACCGTCAAATACGCAGGTTGAACATCGTTTGCATGATCAATGGCAAAACGGTAAT

CCCCAGCGCTGGCACCTGACGCGAGACGATC

>151 bp product from linear template S.enterica subsp. enterica serovar Enteritis, base 802 to base 952 (FW - RV).

CAGCGCTGGGGAAATGACTATTCCGTCAATATTCAGGCGCGGCAAGCAGGGGAGTTTTCG

TTAATACCGTCAAATACGCAGGTTGAACATCGTTTGCATGATCAATGGCAAAACGGTAAT

CCCCAGCGCTGGCACCTGACGCGAGACGATC

>151 bp product from linear template S.enterica subsp. enterica serovar Stanley, base 802 to base 952 (FW - RV).

CAGCGCTGGGGAAATGACTATTCCGTCAATATTCAGGCGCGGCAAGCAGGGGAGTTTTCG

TTAATACCGTCAAATACGCAGGTTGAACATCGTTTGCATGATCAATGGCAAAACGGTAAT

CCCCAGCGCTGGCACCTGACGCGAGACGATC

>151 bp product from linear template S.enterica subsp. enterica YU39, base 802 to base 952 (FW - RV).

CAGCGCTGGGGAAATGACTATTCCGTCAATATTCAGGCGCGGCAAGCAGGGGAGTTTTCG

TTAATACCGTCAAATACGCAGGTTGAACATCGTTTGCATGATCAATGGCAAAACGGTAAT

CCCCAGCGCTGGCACCTGACGCGAGACGATC

>151 bp product from linear template S.enterica subsp. enterica serovar Blockley, base 802 to base 952 (FW - RV).

CAGCGCTGGGGAAATGACTATTCCGTCAATATTCAGGCGCGGCAAGCAGGGGAGTTTTCG

TTAATACCGTCAAATACGCAGGTTGAACATCGTTTGCATGATCAATGGCAAAACGGTAAT

CCCCAGCGCTGGCACCTGACGCGAGACGATC

>151 bp product from linear template S.enterica subsp. enterica serovar Sloterdijk, base 802 to base 952 (FW - RV).

CAGCGCTGGGGAAATGACTATTCCGTCAATATTCAGGCGCGGCAAGCAGGGGAGTTTTCG

TTAATACCGTCAAATACGCAGGTTGAACATCGTTTGCATGATCAATGGCAAAACGGTAAT

CCCCAGCGCTGGCACCTGACGCGAGACGATC

>151 bp product from linear template S.enterica subsp. enterica serovar Braenderup, base 802 to base 952 (FW - RV).

CAGCGCTGGGGAAATGACTATTCCGTCAATATTCAGGCGCGGCAAGCAGGGGAGTTTTCG

TTAATACCGTCAAATACGCAGGTTGAACATCGTTTGCATGATCAATGGCAAAACGGTAAT

CCCCAGCGCTGGCACCTGACGCGAGACGATC

>151 bp product from linear template S.enterica subsp. enterica serovar Goldcoast, base 802 to base 952 (FW - RV).

CAGCGCTGGGGAAATGACTATTCCGTCAATATTCAGGCGCGGCAAGCAGGGGAGTTTTCG

TTAATACCGTCAAATACGCAGGTTGAACATCGTTTGCATGATCAATGGCAAAACGGTAAT

CCCCAGCGCTGGCACCTGACGCGAGACGATC

>151 bp product from linear template S.enterica subsp. enterica serovar Bareilly, base 802 to base 952 (FW - RV).

CAGCGCTGGGGAAATGACTATTCCGTCAATATTCAGGCGCGGCAAGCAGGGGAGTTTTCG

TTAATACCGTCAAATACGCAGGTTGAACATCGTTTGCATGATCAATGGCAAAACGGTAAT

CCCCAGCGCTGGCACCTGACGCGAGACGATC

>151 bp product from linear template S.enterica subsp. enterica serovar Mikawasima, base 802 to base 952 (FW - RV).

CAGCGCTGGGGAAATGACTATTCCGTCAATATTCAGGCGCGGCAAGCAGGGGAGTTTTCG

TTAATACCGTCAAATACGCAGGTTGAACATCGTTTGCATGATCAATGGCAAAACGGTAAT

CCCCAGCGCTGGCACCTGACGCGAGACGATC

>151 bp product from linear template S.enterica subsp. enterica serovar Bergen, base 802 to base 952 (FW - RV).

CAGCGCTGGGGAAATGACTATTCCGTCAATATTCAGGCGCGGCAAGCAGGGGAGTTTTCG

TTAATACCGTCAAATACGCAGGTTGAACATCGTTTGCATGATCAATGGCAAAACGGTAAT

CCCCAGCGCTGGCACCTGACGCGAGACGATC

>151 bp product from linear template S.enterica subsp. enterica serovar London, base 802 to base 952 (FW - RV).

CAGCGCTGGGGAAATGACTATTCCGTCAATATTCAGGCGCGGCAAGCAGGGGAGTTTTCG

TTAATACCGTCAAATACGCAGGTTGAACATCGTTTGCATGATCAATGGCAAAACGGTAAT

CCCCAGCGCTGGCACCTGACGCGAGACGATC

>151 bp product from linear template S.enterica subsp. enterica serovar Litchfield, base 802 to base 952 (FW - RV).

CAGCGCTGGGGAAATGACTATTCCGTCAATATTCAGGCGCGGCAAGCAGGGGAGTTTTCG

TTAATACCGTCAAATACGCAGGTTGAACATCGTTTGCATGATCAATGGCAAAACGGTAAT

CCCCAGCGCTGGCACCTGACGCGAGACGATC

>151 bp product from linear template S.enterica subsp. enterica serovar Java strain, base 802 to base 952 (FW - RV).

CAGCGCTGGGGAAATGACTATTCCGTCAATATTCAGGCGCGGCAAGCAGGGGAGTTTTCG

TTAATACCGTCAAATACGCAGGTTGAACATCGTTTGCATGATCAATGGCAAAACGGTAAT

CCCCAGCGCTGGCACCTGACGCGAGACGATC

>151 bp product from linear template S.enterica subsp. enterica serovar Miami, base 802 to base 952 (FW - RV).

CAGCGCTGGGGAAATGACTATTCCGTCAATATTCAGGCGCGGCAAGCAGGGGAGTTTTCG

TTAATACCGTCAAATACGCAGGTTGAACATCGTTTGCATGATCAATGGCAAAACGGTAAT

CCCCAGCGCTGGCACCTGACGCGAGACGATC

>151 bp product from linear template S.enterica subsp. enterica serovar Albany, base 802 to base 952 (FW - RV).

CAGCGCTGGGGAAATGACTATTCCGTCAATATTCAGGCGCGGCAAGCAGGGGAGTTTTCG

TTAATACCGTCAAATACGCAGGTTGAACATCGTTTGCATGATCAATGGCAAAACGGTAAT

CCCCAGCGCTGGCACCTGACGCGAGACGATC

>151 bp product from linear template S.enterica subsp. enterica serovar Heidelberg, base 802 to base 952 (FW - RV).

CAGCGCTGGGGAAATGACTATTCCGTCAATATTCAGGCGCGGCAAGCAGGGGAGTTTTCG

TTAATACCGTCAAATACGCAGGTTGAACATCGTTTGCATGATCAATGGCAAAACGGTAAT

CCCCAGCGCTGGCACCTGACGCGAGACGATC

>151 bp product from linear template S.enterica subsp. enterica serovar Indiana, base 802 to base 952 (FW - RV).

CAGCGCTGGGGAAATGACTATTCCGTCAATATTCAGGCGCGGCAAGCAGGGGAGTTTTCG

TTAATACCGTCAAATACGCAGGTTGAACATCGTTTGCATGATCAATGGCAAAACGGTAAT

CCCCAGCGCTGGCACCTGACGCGAGACGATC

>151 bp product from linear template S.enterica subsp. enterica serovar Birkenhead, base 802 to base 952 (FW - RV).

CAGCGCTGGGGAAATGACTATTCCGTCAATATTCAGGCGCGGCAAGCAGGGGAGTTTTCG

TTAATACCGTCAAATACGCAGGTTGAACATCGTTTGCATGATCAATGGCAAAACGGTAAT

CCCCAGCGCTGGCACCTGACGCGAGACGATC

>151 bp product from linear template S.enterica subsp. enterica serovar Concord, base 802 to base 952 (FW - RV).

CAGCGCTGGGGAAATGACTATTCCGTCAATATTCAGGCGCGGCAAGCAGGGGAGTTTTCG

TTAATACCGTCAAATACGCAGGTTGAACATCGTTTGCATGATCAATGGCAAAACGGTAAT

CCCCAGCGCTGGCACCTGACGCGAGACGATC

>151 bp product from linear template S.enterica subsp. enterica serovar Newport, base 802 to base 952 (FW - RV).

CAGCGCTGGGGAAATGACTATTCCGTCAATATTCAGGCGCGGCAAGCAGGGGAGTTTTCG

TTAATACCGTCAAATACGCAGGTTGAACATCGTTTGCATGATCAATGGCAAAACGGTAAT

CCCCAGCGCTGGCACCTGACGCGAGACGATC

>151 bp product from linear template S.enterica subsp. enterica serovar California, base 802 to base 952 (FW - RV).

CAGCGCTGGGGAAATGACTATTCCGTCAATATTCAGGCGCGGCAAGCAGGGGAGTTTTCG

TTAATACCGTCAAATACGCAGGTTGAACATCGTTTGCATGATCAATGGCAAAACGGTAAT

CCCCAGCGCTGGCACCTGACGCGAGACGATC

>151 bp product from linear template S.enterica subsp. enterica serovar Mbandaka, base 802 to base 952 (FW - RV).

CAGCGCTGGGGAAATGACTATTCCGTCAATATTCAGGCGCGGCAAGCAGGGGAGTTTTCG

TTAATACCGTCAAATACGCAGGTTGAACATCGTTTGCATGATCAATGGCAAAACGGTAAT

CCCCAGCGCTGGCACCTGACGCGAGACGATC

>151 bp product from linear template S.enterica subsp. enterica serovar Moero, base 802 to base 952 (FW - RV).

CAGCGCTGGGGAAATGACTATTCCGTCAATATTCAGGCGCGGCAAGCAGGGGAGTTTTCG

TTAATACCGTCAAATACGCAGGTTGAACATCGTTTGCATGATCAATGGCAAAACGGTAAT

CCCCAGCGCTGGCACCTGACGCGAGACGATC

>151 bp product from linear template S.enterica subsp. enterica serovar Milwaukee, base 802 to base 952 (FW - RV).

CAGCGCTGGGGAAATGACTATTCCGTCAATATTCAGGCGCGGCAAGCAGGGGAGTTTTCG

TTAATACCGTCAAATACGCAGGTTGAACATCGTTTGCATGATCAATGGCAAAACGGTAAT

CCCCAGCGCTGGCACCTGACGCGAGACGATC

>151 bp product from linear template S.enterica subsp. enterica serovar Hvitting, base 802 to base 952 (FW - RV).

CAGCGCTGGGGAAATGACTATTCCGTCAATATTCAGGCGCGGCAAGCAGGGGAGTTTTCG

TTAATACCGTCAAATACGCAGGTTGAACATCGTTTGCATGATCAATGGCAAAACGGTAAT

CCCCAGCGCTGGCACCTGACGCGAGACGATC

>151 bp product from linear template S.enterica subsp. enterica serovar Ouakam, base 802 to base 952 (FW - RV).

CAGCGCTGGGGAAATGACTATTCCGTCAATATTCAGGCGCGGCAAGCAGGGGAGTTTTCG

TTAATACCGTCAAATACGCAGGTTGAACATCGTTTGCATGATCAATGGCAAAACGGTAAT

CCCCAGCGCTGGCACCTGACGCGAGACGATC

>151 bp product from linear template S.enterica subsp. enterica serovar Wandsworth, base 802 to base 952 (FW - RV).

CAGCGCTGGGGAAATGACTATTCCGTCAATATTCAGGCGCGGCAAGCAGGGGAGTTTTCG

TTAATACCGTCAAATACGCAGGTTGAACATCGTTTGCATGATCAATGGCAAAACGGTAAT

CCCCAGCGCTGGCACCTGACGCGAGACGATC

>151 bp product from linear template S.enterica subsp. enterica serovar Manchester, base 802 to base 952 (FW - RV).

CAGCGCTGGGGAAATGACTATTCCGTCAATATTCAGGCGCGGCAAGCAGGGGAGTTTTCG

TTAATACCGTCAAATACGCAGGTTGAACATCGTTTGCATGATCAATGGCAAAACGGTAAT

CCCCAGCGCTGGCACCTGACGCGAGACGATC

>151 bp product from linear template S.enterica subsp. enterica serovar Krefeld, base 802 to base 952 (FW - RV).

CAGCGCTGGGGAAATGACTATTCCGTCAATATTCAGGCGCGGCAAGCAGGGGAGTTTTCG

TTAATACCGTCAAATACGCAGGTTGAACATCGTTTGCATGATCAATGGCAAAACGGTAAT

CCCCAGCGCTGGCACCTGACGCGAGACGATC

>151 bp product from linear template S.enterica subsp. enterica serovar Inverness, base 802 to base 952 (FW - RV).

CAGCGCTGGGGAAATGACTATTCCGTCAATATTCAGGCGCGGCAAGCAGGGGAGTTTTCG

TTAATACCGTCAAATACGCAGGTTGAACATCGTTTGCATGATCAATGGCAAAACGGTAAT

CCCCAGCGCTGGCACCTGACGCGAGACGATC

>151 bp product from linear template S.enterica subsp. enterica serovar Virchow, base 802 to base 952 (FW - RV).

CAGCGCTGGGGAAATGACTATTCCGTCAATATTCAGGCGCGGCAAGCAGGGGAGTTTTCG

TTAATACCGTCAAATACGCAGGTTGAACATCGTTTGCATGATCAATGGCAAAACGGTAAT

CCCCAGCGCTGGCACCTGACGCGAGACGATC

>151 bp product from linear template S.enterica subsp. enterica serovar Orion, base 802 to base 952 (FW - RV).

CAGCGCTGGGGAAATGACTATTCCGTCAATATTCAGGCGCGGCAAGCAGGGGAGTTTTCG

TTAATACCGTCAAATACGCAGGTTGAACATCGTTTGCATGATCAATGGCAAAACGGTAAT

CCCCAGCGCTGGCACCTGACGCGAGACGATC

>151 bp product from linear template S.enterica subsp. enterica serovar Derby, base 802 to base 952 (FW - RV).

CAGCGCTGGGGAAATGACTATTCCGTCAATATTCAGGCGCGGCAAGCAGGGGAGTTTTCG

TTAATACCGTCAAATACGCAGGTTGAACATCGTTTGCATGATCAATGGCAAAACGGTAAT

CCCCAGCGCTGGCACCTGACGCGAGACGATC

>151 bp product from linear template S.enterica subsp. enterica serovar Nchanga, base 802 to base 952 (FW - RV).

CAGCGCTGGGGAAATGACTATTCCGTCAATATTCAGGCGCGGCAAGCAGGGGAGTTTTCG

TTAATACCGTCAAATACGCAGGTTGAACATCGTTTGCATGATCAATGGCAAAACGGTAAT

CCCCAGCGCTGGCACCTGACGCGAGACGATC

>151 bp product from linear template S.enterica subsp. enterica serovar Livingston, base 802 to base 952 (FW - RV).

CAGCGCTGGGGAAATGACTATTCCGTCAATATTCAGGCGCGGCAAGCAGGGGAGTTTTCG

TTAATACCGTCAAATACGCAGGTTGAACATCGTTTGCATGATCAATGGCAAAACGGTAAT

CCCCAGCGCTGGCACCTGACGCGAGACGATC

>151 bp product from linear template S.enterica subsp. enterica serovar Reading, base 802 to base 952 (FW - RV).

CAGCGCTGGGGAAATGACTATTCCGTCAATATTCAGGCGCGGCAAGCAGGGGAGTTTTCG

TTAATACCGTCAAATACGCAGGTTGAACATCGTTTGCATGATCAATGGCAAAACGGTAAT

CCCCAGCGCTGGCACCTGACGCGAGACGATC

>151 bp product from linear template S.enterica subsp. enterica serovar Kottbus, base 802 to base 952 (FW - RV).

CAGCGCTGGGGAAATGACTATTCCGTCAATATTCAGGCGCGGCAAGCAGGGGAGTTTTCG

TTAATACCGTCAAATACGCAGGTTGAACATCGTTTGCATGATCAATGGCAAAACGGTAAT

CCCCAGCGCTGGCACCTGACGCGAGACGATC

>151 bp product from linear template S.enterica subsp. enterica serovar Potsdam, base 801 to base 951 (FW - RV).

CAGCGCTGGGGAAATGACTATTCCGTCAATATTCAGGCGCGGCAAGCAGGGGAGTTTTCG

TTAATACCGTCAAATACGCAGGTTGAACATCGTTTGCATGATCAATGGCAAAACGGTAAT

CCCCAGCGCTGGCACCTGACGCGAGACGATC

>151 bp product from linear template S.enterica subsp. enterica serovar Infantis, base 802 to base 952 (FW - RV).

CAGCGCTGGGGAAATGACTATTCCGTCAATATTCAGGCGCGGCAAGCAGGGGAGTTTTCG

TTAATACCGTCAAATACGCAGGTTGAACATCGTTTGCATGATCAATGGCAAAACGGTAAT

CCCCAGCGCTGGCACCTGACGCGAGACGATC

>151 bp product from linear template S.enterica subsp. enterica serovar Worthing, base 802 to base 952 (FW - RV).

CAGCGCTGGGGAAATGACTATTCCGTCAATATTCAGGCGCGGCAAGCAGGGGAGTTTTCG

TTAATACCGTCAAATACGCAGGTTGAACATCGTTTGCATGATCAATGGCAAAACGGTAAT

CCCCAGCGCTGGCACCTGACGCGAGACGATC

>151 bp product from linear template S.enterica subsp. enterica serovar Adjame, base 802 to base 952 (FW - RV).

CAGCGCTGGGGAAATGACTATTCCGTCAATATTCAGGCGCGGCAAGCAGGGGAGTTTTCG

TTAATACCGTCAAATACGCAGGTTGAACATCGTTTGCATGATCAATGGCAAAACGGTAAT

CCCCAGCGCTGGCACCTGACGCGAGACGATC

>151 bp product from linear template S.enterica subsp. enterica serovar Aberdeen, base 802 to base 952 (FW - RV).

CAGCGCTGGGGAAATGACTATTCCGTCAATATTCAGGCGCGGCAAGCAGGGGAGTTTTCG

TTAATACCGTCAAATACGCAGGTTGAACATCGTTTGCATGATCAATGGCAAAACGGTAAT

CCCCAGCGCTGGCACCTGACGCGAGACGATC

>151 bp product from linear template S.enterica subsp. enterica serovar Minnesota, base 802 to base 952 (FW - RV).

CAGCGCTGGGGAAATGACTATTCCGTCAATATTCAGGCGCGGCAAGCAGGGGAGTTTTCG

TTAATACCGTCAAATACGCAGGTTGAACATCGTTTGCATGATCAATGGCAAAACGGTAAT

CCCCAGCGCTGGCACCTGACGCGAGACGATC

>151 bp product from linear template S.enterica subsp. enterica serovar Nitra, base 802 to base 952 (FW - RV).

CAGCGCTGGGGAAATGACTATTCCGTCAATATTCAGGCGCGGCAAGCAGGGGAGTTTTCG

TTAATACCGTCAAATACGCAGGTTGAACATCGTTTGCATGATCAATGGCAAAACGGTAAT

CCCCAGCGCTGGCACCTGACGCGAGACGATC

>151 bp product from linear template S.enterica subsp. enterica serovar Hillingdon, base 802 to base 952 (FW - RV).

CAGCGCTGGGGAAATGACTATTCCGTCAATATTCAGGCGCGGCAAGCAGGGGAGTTTTCG

TTAATACCGTCAAATACGCAGGTTGAACATCGTTTGCATGATCAATGGCAAAACGGTAAT

CCCCAGCGCTGGCACCTGACGCGAGACGATC

>151 bp product from linear template S.enterica subsp. enterica serovar Bredeney, base 802 to base 952 (FW - RV).

CAGCGCTGGGGAAATGACTATTCCGTCAATATTCAGGCGCGGCAAGCAGGGGAGTTTTCG

TTAATACCGTCAAATACGCAGGTTGAACATCGTTTGCATGATCAATGGCAAAACGGTAAT

CCCCAGCGCTGGCACCTGACGCGAGACGATC

>151 bp product from linear template S.enterica subsp. enterica serovar Abortusequi, base 802 to base 952 (FW - RV).

CAGCGCTGGGGAAATGACTATTCCGTCAATATTCAGGCGCGGCAAGCAGGGGAGTTTTCG

TTAATACCGTCAAATACGCAGGTTGAACATCGTTTGCATGATCAATGGCAAAACGGTAAT

CCCCAGCGCTGGCACCTGACGCGAGACGATC

>151 bp product from linear template S.enterica subsp. enterica serovar Choleraesuis, base 802 to base 952 (FW - RV).

CAGCGCTGGGGAAATGACTATTCCGTCAATATTCAGGCGCGGCAAGCAGGGGAGTTTTCG

TTAATACCGTCAAATACGCAGGTTGAACATCGTTTGCATGATCAATGGCAAAACGGTAAT

CCCCAGCGCTGGCACCTGACGCGAGACGATC

>151 bp product from linear template S.enterica subsp. enterica serovar Cubana, base 802 to base 952 (FW - RV).

CAGCGCTGGGGAAATGACTATTCCGTCAATATTCAGGCGCGGCAAGCAGGGGAGTTTTCG

TTAATACCGTCAAATACGCAGGTTGAACATCGTTTGCATGATCAATGGCAAAACGGTAAT

CCCCAGCGCTGGCACCTGACGCGAGACGATC

>151 bp product from linear template S.enterica subsp. enterica serovar Agona, base 802 to base 952 (FW - RV).

CAGCGCTGGGGAAATGACTATTCCGTCAATATTCAGGCGCGGCAAGCAGGGGAGTTTTCG

TTAATACCGTCAAATACGCAGGTTGAACATCGTTTGCATGATCAATGGCAAAACGGTAAT

CCCCAGCGCTGGCACCTGACGCGAGACGATC

>151 bp product from linear template S.enterica subsp. enterica serovar Montevideo, base 802 to base 952 (FW - RV).

CAGCGCTGGGGAAATGACTATTCCGTCAATATTCAGGCGCGGCAAGCAGGGGAGTTTTCG

TTAATACCGTCAAATACGCAGGTTGAACATCGTTTGCATGATCAATGGCAAAACGGTAAT

CCCCAGCGCTGGCACCTGACGCGAGACGATC

>151 bp product from linear template S.enterica subsp. enterica serovar Tennessee, base 802 to base 952 (FW - RV).

CAGCGCTGGGGAAATGACTATTCCGTCAATATTCAGGCGCGGCAAGCAGGGGAGTTTTCG

TTAATACCGTCAAATACGCAGGTTGAACATCGTTTGCATGATCAATGGCAAAACGGTAAT

CCCCAGCGCTGGCACCTGACGCGAGACGATC

>151 bp product from linear template S.enterica subsp. enterica serovar Thompson, base 802 to base 952 (FW - RV).

CAGCGCTGGGGAAATGACTATTCCGTCAATATTCAGGCGCGGCAAGCAGGGGAGTTTTCG

TTAATACCGTCAAATACGCAGGTTGAACATCGTTTGCATGATCAATGGCAAAACGGTAAT

CCCCAGCGCTGGCACCTGACGCGAGACGATC

>151 bp product from linear template S.enterica subsp. enterica serovar Manhattan, base 802 to base 952 (FW - RV).

CAGCGCTGGGGAAATGACTATTCCGTCAATATTCAGGCGCGGCAAGCAGGGGAGTTTTCG

TTAATACCGTCAAATACGCAGGTTGAACATCGTTTGCATGATCAATGGCAAAACGGTAAT

CCCCAGCGCTGGCACCTGACGCGAGACGATC

>151 bp product from linear template S.enterica subsp. enterica serovar Yovokome, base 802 to base 952 (FW - RV).

CAGCGCTGGGGAAATGACTATTCCGTCAATATTCAGGCGCGGCAAGCAGGGGAGTTTTCG

TTAATACCGTCAAATACGCAGGTTGAACATCGTTTGCATGATCAATGGCAAAACGGTAAT

CCCCAGCGCTGGCACCTGACGCGAGACGATC

>151 bp product from linear template S.enterica subsp. enterica serovar Bardo, base 802 to base 952 (FW - RV).

CAGCGCTGGGGAAATGACTATTCCGTCAATATTCAGGCGCGGCAAGCAGGGGAGTTTTCG

TTAATACCGTCAAATACGCAGGTTGAACATCGTTTGCATGATCAATGGCAAAACGGTAAT

CCCCAGCGCTGGCACCTGACGCGAGACGATC

>151 bp product from linear template S.enterica subsp. enterica serovar Cerro, base 802 to base 952 (FW - RV).

CAGCGCTGGGGAAATGACTACTCCGTCAATATTCAGGCGCGGCAAGCAGGGGAGTTTTCG

TTAATACCGTCAAATACGCAGGTTGAACATCGTTTGCATGATCAATGGCAAAACGGTAAT

CCCCAGCGCTGGCACCTGACGCGAGACGATC

>151 bp product from linear template S.enterica subsp. enterica serovar Borreze, base 802 to base 952 (FW - RV).

CAGCGCTGGGGAAATGACTATTCCGTCAATATTCAGGCGCGGCAAGCAGGGGAGTTTTCG

TTAATACCGTCAAATACGCAGGTTGAACATCGTTTGCATGATCAATGGCAAAACGGTAAT

CCCCAGCGCTGGCACCTGACGCGAGACGATC

>151 bp product from linear template S.enterica subsp. enterica Serovar Cubana, base 802 to base 952 (FW - RV).

CAGCGCTGGGGAAATGACTATTCCGTCAATATTCAGGCGCGGCAAGCAGGGGAGTTTTCG

TTAATACCGTCAAATACGCAGGTTGAACATCGTTTGCATGATCAATGGCAAAACGGTAAT

CCCCAGCGCTGGCACCTGACGCGAGACGATC

>151 bp product from linear template S.enterica subsp. enterica serovar Bovismorbificans, base 802 to base 952 (FW - RV).

CAGCGCTGGGGAAATGACTATTCCGTCAATATTCAGGCGCGGCAAGCAGGGGAGTTTTCG

TTAATACCGTCAAATACGCAGGTTGAACATCGTTTGCATGATCAATGGCAAAACGGTAAT

CCCCAGCGCTGGCACCTGACGCGAGACGATC

>151 bp product from linear template S.enterica subsp. enterica serovar Hartford, base 802 to base 952 (FW - RV).

CAGCGCTGGGGAAATGACTATTCCGTCAATATTCAGGCGCGGCAAGCAGGGGAGTTTTCG

TTAATACCGTCAAATACGCAGGTTGAACATCGTTTGCATGATCAATGGCAAAACGGTAAT

CCCCAGCGCTGGCACCTGACGCGAGACGATC

>151 bp product from linear template S.enterica subsp. enterica serovar Senftenberg, base 802 to base 952 (FW - RV).

CAACGCTGGGGAAATGACTATTCCGTCAATATTCAGGCGCGGCAAGCAGGGGAGTTTTCG

TTAATACCGTCAAATACGCAGGTTGAACATCGTTTGCATGATCAATGGCAAAACGGTAAT

CCCCAGCGCTGGCACCTGACGCGAGACGATC

>151 bp product from linear template S.enterica subsp. enterica serovar Muenchen, base 802 to base 952 (FW - RV).

CAGCGCTGGGGAAATGACTATTCCGTCAATATTCAGGCGCGGCAAGCAGGGGAGTTTTCG

TTAATACCGTCAAATACGCAGGTTGAACATCGTTTGCATGATCAATGGCAAAACGGTAAT

CCCCAGCGCTGGCACCTGACGCGAGACGATC

>151 bp product from linear template S.enterica subsp. enterica serovar Napoli, base 802 to base 952 (FW - RV).

CAGCGCTGGGGAAATGACTATTCCGTCAATATTCAGGCGCGGCAAGCAGGGGAGTTTTCG

TTAATACCGTCAAATACGCAGGTTGAACATCGTTTGCATGATCAATGGCAAAACGGTAAT

CCCCAGCGCTGGCACCTGACGCGAGACGATC

>151 bp product from linear template S.enterica subsp. enterica serovar Havana, base 802 to base 952 (FW - RV).

CAGCGCTGGGGAAATGACTATTCCGTCAATATTCAGGCGCGGCAAGCAGGGGAGTTTTCG

TTAATACCGTCAAATACGCAGGTTGAACATCGTTTGCATGATCAATGGCAAAACGGTAAT

CCCCAGCGCTGGCACCTGACGCGAGACGATC

>151 bp product from linear template S.enterica subsp. enterica serovar Anatum, base 802 to base 952 (FW - RV).

CAGCGCTGGGGAAATGACTATTCCGTCAATATTCAGGCGCGGCAAGCAGGGGAGTTTTCG

TTAATACCGTCAAATACGCAGGTTGAACATCGTTTGCATGATCAATGGCAAAACGGTAAT

CCCCAGCGCTGGCACCTGACGCGAGACGATC

>151 bp product from linear template S.enterica subsp. enterica serovar Hadar, base 802 to base 952 (FW - RV).

CAGCGCTGGGGAAATGACTATTCCGTCAATATTCAGGCGCGGCAAGCAGGGGAGTTTTCG

TTAATACCGTCAAATACGCAGGTTGAACATCGTTTGCATGATCAATGGCAAAACGGTAAT

CCCCAGCGCTGGCACCTGACGCGAGACGATC

>151 bp product from linear template S.enterica subsp. enterica serovar Waycross, base 802 to base 952 (FW - RV).

CAGCGCTGGGGAAATGACTATTCCGTCAATATTCAGGCGCGGCAAGCAGGGGAGTTTTCG

TTAATACCGTCAAATACGCAGGTTGAACATCGTTTGCATGATCAATGGCAAAACGGTAAT

CCCCAGCGCTGGCACCTGACGCGAGACGATC

>151 bp product from linear template S.enterica subsp. enterica serovar Berta, base 802 to base 952 (FW - RV).

CAGCGCTGGGGAAATGACTATTCCGTCAATATTCAGGCGCGGCAAGCAGGGGAGTTTTCG

TTAATACCGTCAAATACGCAGGTTGAACATCGTTTGCATGATCAATGGCAAAACGGTAAT

CCCCAGCGCTGGCACCTGACGCGAGACGATC

>151 bp product from linear template S.enterica subsp. enterica serovar Hayindogo, base 802 to base 952 (FW - RV).

CAGCGCTGGGGAAATGACTATTCCGTCAATATTCAGGCGCGGCAAGCAGGGGAGTTTTCG

TTAATACCGTCAAATACGCAGGTTGAACATCGTTTGCATGATCAATGGCAAAACGGTAAT

CCCCAGCGCTGGCACCTGACGCGAGACGATC

>151 bp product from linear template S.enterica subsp. enterica serovar Moscow, base 802 to base 952 (FW - RV).

CAGCGCTGGGGAAATGACTATTCCGTCAATATTCAGGCGCGGCAAGCAGGGGAGTTTTCG

TTAATACCGTCAAATACGCAGGTTGAACATCGTTTGCATGATCAATGGCAAAACGGTAAT

CCCCAGCGCTGGCACCTGACGCGAGACGATC

>151 bp product from linear template S.enterica subsp. enterica serovar Blegdam, base 802 to base 952 (FW - RV).

CAGCGCTGGGGAAATGACTATTCCGTCAATATTCAGGCGCGGCAAGCAGGGGAGTTTTCG

TTAATACCGTCAAATACGCAGGTTGAACATCGTTTGCATGATCAATGGCAAAACGGTAAT

CCCCAGCGCTGGCACCTGACGCGAGACGATC

>151 bp product from linear template S.enterica subsp. enterica serovar Gallinarum, base 802 to base 952 (FW - RV).

CAGCGCTGGGGAAATGACTATTCCGTCAATATTCAGGCGCGGCAAGCAGGGGAGTTTTCG

TTAATACCGTCAAATACGCAGGTTGAACATCGTTTGCATGATCAATGGCAAAACGGTAAT

CCCCAGCGCTGGCACCTGACGCGAGACGATC

>151 bp product from linear template S.enterica subsp. enterica serovar Newlands, base 802 to base 952 (FW - RV).

CAGCGCTGGGGAAATGACTATTCCGTCAATATTCAGGCGCGGCAAGCAGGGGAGTTTTCG

TTAATACCGTCAAATACGCAGGTTGAACATCGTTTGCATGATCAATGGCAAAACGGTAAT

CCCCAGCGCTGGCACCTGACGCGAGACGATC

>151 bp product from linear template S.enterica subsp. enterica serovar Kiambu, base 802 to base 952 (FW - RV).

CAGCGCTGGGGAAATGACTATTCCGTCAATATTCAGGCGCGGCAAGCAGGGGAGTTTTCG

TTAATACCGTCAAATACGCAGGTTGAACATCGTTTGCATGATCAATGGCAAAACGGTAAT

CCCCAGCGCTGGCACCTGACGCGAGACGATC

>151 bp product from linear template S.enterica subsp. enterica serovar Uganda, base 802 to base 952 (FW - RV).

CAGCGCTGGGGAAATGACTATTCCGTCAATATTCAGGCGCGGCAAGCAGGGGAGTTTTCG

TTAATACCGTCAAATACGCAGGTTGAACATCGTTTGCATGATCAATGGCAAAACGGTAAT

CCCCAGCGCTGGCACCTGACGCGAGACGATC

>151 bp product from linear template S.enterica subsp. enterica serovar Corvallis, base 802 to base 952 (FW - RV).

CAGCGCTGGGGAAATGACTATTCCGTCAATATTCAGGCGCGGCAAGCAGGGGAGTTTTCG

TTAATACCGTCAAATACGCAGGTTGAACATCGTTTGCATGATCAATGGCAAAACGGTAAT

CCCCAGCGCTGGCACCTGACGCGAGATGATC

>151 bp product from linear template S.enterica subsp. enterica serovar Shamba, base 802 to base 952 (FW - RV).

CAGCGCTGGGGAAATGACTATTCCGTCAATATTCAGGCGCGGCAAGCAGGGGAGTTTTCG

TTAATACCGTCAAATACGCAGGTTGAACATCGTTTGCATGATCAATGGCAAAACGGTAAT

CCCCAGCGCTGGCACCTGACGCGAGACGATC

>151 bp product from linear template S.enterica subsp. enterica serovar Javiana, base 802 to base 952 (FW - RV).

CAGCGCTGGGGAAATGACTATTCCGTCAATATTCAGGCGCGGCAAGCAGGGGAGTTTTCG

TTAATACCGTCAAATACGCAGGTTGAACATCGTTTGCATGATCAATGGCAAAACGGTAAT

CCCCAGCGCTGGCACCTGACGCGAGACGATC

>151 bp product from linear template S.enterica subsp. enterica serovar Paratyphi C, base 802 to base 952 (FW - RV).

CAGCGCTGGGGAAATGACTATTCCGTCAATATTCAGGCGCGGCAAGCAGGGGAGTTTTCG

TTAATACCGTCAAATACGCAGGTTGAACATCGTTTGCATGATCAATGGCAAAACGGTAAT

CCCCAGCGCTGGCACCTGACGCGAGACGATC

>151 bp product from linear template S.enterica subsp. enterica serovar Paratyphi A, base 802 to base 952 (FW - RV).

CAGCGCTGGGGAAATGACTATTCCGTCAATATTCAGGCGCGGCAAGCAGGGGAGTTTTCG

TTAATACCGTCAAATACGCAGGTTGAACATCGTTTGCATGATCAATGGCAAAACGGTAAT

CCCCAGCGCTGGCACCTGACGCGAGACGATC

>151 bp product from linear template S.enterica subsp. enterica serovar Paratyphi B, base 802 to base 952 (FW - RV).

CAGCGCTGGGGAAATGACTATTCCGTCAATATTCAGGCGCGGCAAGCAGGGGAGTTTTCG

TTAATACCGTCAAATACGCAGGTTGAACATCGTTTGCATGATCAATGGCAAAACGGTAAT

CCCCAGCGCTGGCACCTGACGCGAGACGATC

>151 bp product from linear template S.enterica subsp. enterica serovar Typhi, base 802 to base 952 (FW - RV).

CAGCGCTGGGGAAATGACTATTCCGTCAATATTCAGGCGCGGCAAGCAGGGGAGTTTTCG

TTAATACCGTCAAATACGCAGGTTGAACATCGTTTGCATGATCAATGGCAAAACGGTAAT

CCCCAGCGCTGGCACCTGACGCGAGACGATC

>151 bp product from linear template S.enterica subsp. enterica serovar Daytona, base 802 to base 952 (FW - RV).

CAGCGCTGGGGAAATGACTATTCCGTCAATATTCAGGCGCGGCAAGCAGGGGAGTTTTCG

TTAATACCGTCAAATACGCAGGTTGAACATCGTTTGCATGATCAATGGCAAAACGGTAAT

CCCCAGCGCTGGCACCTGACGCGAGACGATC

>151 bp product from linear template S.enterica subsp. enterica serovar Poona, base 802 to base 952 (FW - RV).

CAGCGCTGGGGAAATGACTATTCCGTCAATATTCAGGCGCGGCAAGCAGGGGAGTTTTCG

TTAATACCGTCAAATACGCAGGTTGAACATCGTTTGCATGATCAATGGCAAAACGGTAAT

CCCCAGCGCTGGCACCTGACGCGAGACGATC

>151 bp product from linear template S.enterica subsp. enterica serovar Pullorum, base 802 to base 952 (FW - RV).

CAGCGCTGGGGAAATGACTATTCCGTCAATATTCAGGCGCGGCAAGCAGGGGAGTTTTCG

TTAATACCGTCAAATACGCAGGTTGAACATCGTTTGCATGATCAATGGCAAAACGGTAAT

CCCCAGCGCTGGCACCTGACGCGAGACGATC

>151 bp product from linear template S.enterica subsp. arizonae, base 802 to base 952 (FW - RV).

CAGCGCTGGGGAAATGACTATTCAGTCAATATTCAGGCGCGCCAGGTAGGGGAGTTTTCG

TTAATACCGTCAAATACGCAGGTTGAGCATCGTTTGCATGATCAATGGCAAAACGGTAAC

CCCCAGCGCTGGCACCTGACGCGAGACGATC

>151 bp product from linear template S.enterica subsp. enterica serovar Westhampton, base 802 to base 952 (FW - RV).

CAGCGCTGGGGAAATGACTATTCCGTCAATATTCAGGCGCGGCAAGCAGGGGAGTTTTCG

TTAATACCGTCAAATACGCAGGTTGAACATCGTTTGCATGATCAATGGCAAAACGGTAAT

CCCCAGCGCTGGCACCTGACGCGAGACGATC

>151 bp product from linear template S.enterica subsp. enterica serovar Kentucky, base 802 to base 952 (FW - RV).

CAGCGCTGGGGAAATGACTATTCCGTCAATATTCAGGCGCGGCAAGCAGGGGAGTTTTCG

TTAATACCGTCAAATACGCAGGTTGAACATCGTTTGCATGATCAATGGCAAAACGGTAAT

CCCCAGCGCTGGCACCTGACGCGAGACGATC

>151 bp product from linear template S.enterica subsp. enterica serovar Weltevreden, base 802 to base 952 (FW - RV).

CAGCGCTGGGGAAATGACTATTCCGTCAATATTCAGGCGCGGCAAGCAGGGGAGTTTTCG

TTAATACCGTCAAATACGCAGGTTGAACATCGTTTGCATGATCAATGGCAAAACGGTAAT

CCCCAGCGCTGGCACCTGACGCGAGACGATC

>151 bp product from linear template S.bongori serovar 66:z41, base 793 to base 943 (FW - RV).

CAGCGCTGGGGAAATGACTACTCCGTCAATATTCAGGCGCGGCAGGCAGGGGAGTTTTCG

TTAATACCGTCAAATACGCAGGTTGAGCATCGTTTGCATGATCAATGGCAGAACGGTAAC

CCTCAGCGCTGGCACCTGATGCGAGACGATC

>151 bp product from linear template S.bongori serovar 48:z81, base 793 to base 943 (FW - RV).

CAGCGCTGGGGAAATGACTATTCCGTCAATATTCAGGCGCGGCAGGCAGGGGAGTTTTCG

TTAGTACCGTCAAATACGCAGGTTGAGCATCGTTTGCATGATCAATGGCAGAACGGTAAC

CCTCAGCGCTGGCACCTGATGCGAGACGATC

>151 bp product from linear template S.bongori serovar 40:z35, base 793 to base 943 (FW - RV).

CAGCGCTGGGGAAATGACTATTCCGTCAATATTCAGGCGCGGCAGGCAGGGGAGTTTTCG

TTAGTACCGTCAAATACGCAGGTTGAGCATCGTTTGCATGATCAATGGCAGAACGGTAAC

CCTCAGCGCTGGCACCTGATGCGAGACGATC

>151 bp product from linear template S.bongori, base 793 to base 943 (FW - RV).

CAGCGCTGGGGAAATGACTATTCCGTCAATATTCAGGCGCGGCAGGCAGGGGAGTTTTCG

TTAGTACCGTCAAATACGCAGGTTGAGCATCGTTTGCATGATCAATGGCAGAACGGTAAC

CCTCAGCGCTGGCACCTGATGCGAGACGATC

>151 bp product from linear template S.bongori serovar 48:z41, base 793 to base 943 (FW - RV).

CAGCGCTGGGGAAATGACTATTCCGTCAATATTCAGGCGCGGCAGGCAGGGGAGTTTTCG

TTAGTACCGTCAAATACGCAGGTTGAGCATCGTTTGCATGATCAATGGCAGAACGGTAAC

CCTCAGCGCTGGCACCTGATGCGAGACGATC

>151 bp product from linear template S.enterica subsp. houtenae serovar Houten, base 802 to base 952 (FW - RV).

CAGCGCTGGGGAAATGACTATTCCGTCAATATTCAGGCACGGCAGGCGGGGGAGTTTTCG

TTAATACCGTCAAATACGCAGGTTGAGCATCGCTTGCATGATCAATGGCAAAACGGTAAT

CCCCAGCGCTGGCACCTGATGCGAGATGATC

>151 bp product from linear template S.enterica subsp. houtenae serovar 44:z4 z32, base 802 to base 952 (FW - RV).

CAGCGCTGGGGAAATGACTATTCCGTCAATATTCAGGCACGGCAGGCGGGGGAGTTTTCG

TTAATACCGTCAAATACGCAGGTTGAACATCGCTTGCATGATCAATGGCAAAACGGTAAT

CCCCAGCGCTGGCACCTGATGCGAGATGATC

>151 bp product from linear template S.enterica subsp. houtenae serovar 43:z4, base 802 to base 952 (FW - RV).

CAGCGCTGGGGAAATGACTATTCCGTCAATATTCAGGCACGGCAGGCGGGGGAGTTTTCG

TTAATACCGTCAAATACGCAGGTTGAACATCGCTTGCATGATCAATGGCAAAACGGTAAT

CCCCAGCGCTGGCACCTGATGCGAGATGATC

>151 bp product from linear template S.enterica subsp. salamae serovar 42:r, base 802 to base 952 (FW - RV).

CAGCGCTGGGGAAATGACTATTCCGTCAATATTCAGACGCGGCAAGTGGGGGAGTTTTCG

TTAATACCGTCAAATACGCAGGTTGAACATCGTTTGCATGATCAATGGCAAAACGGTAAT

CCCCAGCGCTGGCACCTGACGCGAGACGATC

>151 bp product from linear template S.enterica subsp. diarizonae serovar 50:k:z, base 802 to base 952 (FW - RV).

CAGCGCTGGGGAAATGACTATTCAGTCAATATTCAGGCGCGCCAGGTAGGGGAGTTTTCG

TTAATACCGTCAAATACGCAGGTTGAGCATCGTTTGCATGATCAATGGCAAAACGGTAAC

CCCCAGCGCTGGCACCTGACGCGAGACGATC

>151 bp product from linear template S.enterica subsp. diarizonae serovar 60:r:z s, base 802 to base 952 (FW - RV).

CAGCGCTGGGGAAATGACTATTCAGTCAATATTCAGGCGCGCCAGGTAGGGGAGTTTTCG

TTAATACCGTCAAATACGCAGGTTGAACATCGTTTGCATGATCAATGGCAAAACGGTAAC

CCCCAGCGCTGGCACCTGACGCGAGACGATC

>151 bp product from linear template S.enterica subsp. diarizonae serovar 61:k:1, base 802 to base 952 (FW - RV).

CAGCGCTGGGGAAATGACTATTCAGTCAATATTCAGGCGCGCCAGGTAGGGGAGTTTTCG

TTAATACCGTCAAATACGCAGGTTGAGCATCGTTTGCATGATCAATGGCAAAACGGTAAC

CCCCAGCGCTGGCACCTGACGCGAGACGATC

>151 bp product from linear template S.enterica subsp. diarizonae serovar 47:k:z, base 802 to base 952 (FW - RV).

CAGCGCTGGGGAAATGACTATTCAGTCAATATTCAGGCGCGCCAGGTAGGGGAGTTTTCG

TTAATACCGTCAAATACGCAGGTTGAACATCGTTTGCATGATCAATGGCAAAACGGTAAC

CCCCAGCGCTGGCACCTGACGCGAGACGATC

>151 bp product from linear template S.enterica subsp. diarizonae serovar 65:z10:e, base 802 to base 952 (FW - RV).

CAGCGCTGGGGAAATGACTATTCAGTCAATATTCAGGCGCGCCAGGTAGGGGAGTTTTCG

TTAATACCGTCAAATACGCAGGTTGAGCATCGTTTGCATGATCAATGGCAAAACGGTAAC

CCCCAGCGCTGGCACCTGACGCGAGACGATC

>151 bp product from linear template S. enterica subsp. diarizonae serovar 48:i:z, base 802 to base 952 (FW - RV).

CAGCGCTGGGGAAATGACTATTCAGTCAATATTCAGGCGCGCCAGGTAGGGGAGTTTTCG

TTAATACCGTCAAATACGCAGGTTGAGCATCGTTTGCATGATCAATGGCAAAACGGTAAC

CCCCAGCGCTGGCACCTGACGCGAGACGATC

>151 bp product from linear template S.enterica subsp. diarizonae serovar 65:c:z s, base 802 to base 952 (FW - RV).

CAGCGCTGGGGAAATGACTATTCAGTCAATATTCAGGCGCGCCAGGTAGGGGAGTTTTCG

TTAATACCGTCAAATACGCAGGTTGAGCATCGTTTGCATGATCAATGGCAAAACGGTAAC

CCCCAGCGCTGGCACCTGACGCGAGACGATC

>151 bp product from linear template S.enterica subsp. diarizonae serovar 59:z10, base 802 to base 952 (FW - RV).

CAGCGCTGGGGAAATGACTATTCAGTCAATATTCAGGCGCGCCAGGTAGGGGAGTTTTCG

TTAATACCGTCAAATACGCAGGTTGAGCATCGTTTGCATGATCAATGGCAAAACGGTAAC

CCCCAGCGCTGGCACCTGACGCGAGACGATC

>151 bp product from linear template S.enterica subsp. diarizonae serovar 60:r:e, base 802 to base 952 (FW - RV).

CAGCGCTGGGGAAATGACTATTCAGTCAATATTCAGGCGCGCCAGGTAGGGGAGTTTTCG

TTAATACCGTCAAATACGCAGGTTGAGCATCGTTTGCATGATCAATGGCAAAACGGTAAC

CCCCAGCGCTGGCACCTGACGCGAGACGATC

>151 bp product from linear template S.enterica subsp. salamae serovar 6 8:a:z52, base 802 to base 952 (FW - RV).

CAGCGCTGGGGAAATGACTATTCGGTCAATATTCAGGCGCGCCAGGTAGGGGAGTTTTCG

TTAATACCGTCAAATACGCAGGTTGAACATCGTTTGCATGATCAATGGCAAAACGGTAAC

CCCCAGCGCTGGCACCTGACGCGAGACGATC

>151 bp product from linear template S.enterica subsp. salamae serovar 40:c:e n z15, base 802 to base 952 (FW - RV).

CAGCGCTGGGGAAATGACTATTCGGTCAATATTCAGGCGCGCCAGGTAGGGGAGTTTTCG

TTAATACCGTCAAATACGCAGGTTGAACATCGTTTGCATGAGCAATGGCAAAACGGTAAC

CCCCAGCGCTGGCACCTGACGCGAGACGATC

>151 bp product from linear template S.enterica subsp. salamae serovar 55:k:z39 str. 1315K, base 802 to base 952 (FW - RV).

CAGCGCTGGGGAAATGACTATTCGGTCAATATTCAGGCGCGCCAGGTAGGGGAGTTTTCG

TTAATACCGTCAAATACGCAGGTTGAACATCGTTTGCATGAGCAATGGCAAAACGGTAAC

CCCCAGCGCTGGCACCTGACGCGAGACGATC

>151 bp product from linear template S.enterica subsp. salamae serovar Greenside, base 802 to base 952 (FW - RV).

CAGCGCTGGGGAAATGACTATTCCGTCAATATTCAGACGCGGCAAGTGGGGGAGTTTTCG

TTAATACCGTCAAATACGCAGGTTGAACATCGTTTGCATGATCAATGGCAAAACGGTAAT

CCCCAGCGCTGGCACCTGACGCGAGACGATC

>151 bp product from linear template S.enterica subsp. salamae serovar 56:b: 1 5, base 802 to base 952 (FW - RV).

CAGCGCTGGGGAAATGACTATTCCGTCAATATTCAGGCGCGGCAAGTGGGGGAGTTTTCG

TTAATACCGTCAAATACGCAGGTTGAACATCGTTTGCATGATCAATGGCAAAACGGTAAT

CCCCAGCGCTGGCACCTGACGCGAGACGATC

>151 bp product from linear template S.enterica subsp. salamae serovar 56:z10:e n x, base 802 to base 952 (FW - RV).

CAGCGCTGGGGAAATGACTATTCCGTCAATATTCAGGCGCGGCAAGTGGGGGAGTTTTCG

TTAATACCGTCAAATACGCAGGTTGAACATCGTTTGCATGATCAATGGCAAAACGGTAAT

CCCCAGCGCTGGCACCTGACGCGAGACGATC

>151 bp product from linear template S.enterica subsp. salamae serovar 58:c:z6, base 802 to base 952 (FW - RV).

CAGCGCTGGGGAAATGACTATTCCGTCAATATTCAGGCGCGGCAAGTGGGGGAGTTTTCA

TTAATACCGTCAAATACGCAGGTTGAACATCGTTTGCATGATCAATGGCAAAACGGTAAT

CCCCAGCGCTGGCACCTGACGCGAGACGATC

>151 bp product from linear template S.enterica subsp. salamae serovar 42:r:-, base 802 to base 952 (FW - RV).

CAGCGCTGGGGAAATGACTATTCCGTCAATATTCAGACGCGGCAAGTGGGGGAGTTTTCG

TTAATACCGTCAAATACGCAGGTTGAACATCGTTTGCATGATCAATGGCAAAACGGTAAT

CCCCAGCGCTGGCACCTGACGCGAGACGATC

>151 bp product from linear template S.enterica subsp. salamae serovar 57:z29:z42, base 802 to base 952 (FW - RV).

CAGCGCTGGGGAAATGACTATTCCGTCAATATTCAGGCGCGGCAAGTGGGGGAGTTTTCG

TTAATACCGTCAAATACGCAGGTTGAACATCGTTTGCATGATCAATGGCAAAACGGTAAT

CCCCAGCGCTGGCACCTGACGCGAGACGATC

>151 bp product from linear template S.enterica subsp. salamae serovar 60:z10:z39, base 802 to base 952 (FW - RV).

CAGCGCTGGGGAAATGACTATTCCGTCAATATTCAGGCGCGGCAGGTGGGGGAGTTTTCG

TTAATACCGTCAAATACGCAAGTTGAACATCGTTTGCATGATCAATGGCAAAACGGAAAT

CCCCATCGTTGGCACCTGGCGCGAGACGATC

>151 bp product from linear template S.enterica subsp. salamae serovar 48:z81:z39, base 802 to base 952 (FW - RV).

CAGCGCTGGGGAAATGACTATTCCGTCAATATTCAGGCGCGGCAGGTGGGGGAGTTTTCG

TTAATACCGTCAAATACGCAAGTTGAACATCGTTTGCATGATCAATGGCAAAACGGAAAT

CCCCATCGTTGGCACCTGGCGCGAGACGATC

PCR Products results

>151 bp product from linear template CP074649.1:976378-977385 Salmonella enterica subsp. salamae serovar 6,7:m,t:- strain CFSAN028548 chromosome, complete genome, base 802 to base 952 (Forward invj - Reverse invj).

CAGCGCTGGGGAAATGACTATTCCGTCAATATTCAGGCGCGGCAAGCAGGGGAGTTTTCG

TTAATACCGTCAAATACGCAGGTTGAACATCGTTTGCATGATCAATGGCAAAACGGTAAT

CCCCAGCGCTGGCACCTGACGCGAGACGATC

>151 bp product from linear template LS483456.1:1185917-1186924 Salmonella enterica subsp. salamae strain NCTC9930 genome assembly, chromosome: 1, base 802 to base 952 (Forward invj - Reverse invj).

CAGCGCTGGGGAAATGACTATTCCGTCAATATTCAGGCGCGGCAAGTGGGGGAGTTTTCG

TTAATACCGTCAAATACGCAGGTTGAACATCGTTTGCATGATCAATGGCAAAACGGTAAT

CCCCAGCGCTGGCACCTGACGCGAGACGATC

>151 bp product from linear template CP074596.1:1035836-1036843 Salmonella enterica subsp. salamae strain CFSAN001015 chromosome, complete genome, base 802 to base 952 (Forward invj - Reverse invj).

CAGCGCTGGGGAAATGACTATTCCGTCAATATTCAGACGCGGCAAGTGGGGGAGTTTTCG

TTAATACCGTCAAATACGCAGGTTGAACATCGTTTGCATGATCAATGGCAAAACGGTAAT

CCCCAGCGCTGGCACCTGACGCGAGACGATC

>151 bp product from linear template CP053325.1:3631088-3632095 Salmonella enterica subsp. salamae serovar 40:c:e,n,z15 strain 2013K-0524 chromosome, complete genome, base 802 to base 952 (Forward invj - Reverse invj).

CAGCGCTGGGGAAATGACTATTCGGTCAATATTCAGGCGCGCCAGGTAGGGGAGTTTTCG

TTAATACCGTCAAATACGCAGGTTGAACATCGTTTGCATGAGCAATGGCAAAACGGTAAC

CCCCAGCGCTGGCACCTGACGCGAGACGATC

>151 bp product from linear template CP022139.1:1088577-1089584 Salmonella enterica subsp. salamae serovar 55:k:z39 str. 1315K, complete genome, base 802 to base 952 (Forward invj - Reverse invj).

CAGCGCTGGGGAAATGACTATTCGGTCAATATTCAGGCGCGCCAGGTAGGGGAGTTTTCG

TTAATACCGTCAAATACGCAGGTTGAACATCGTTTGCATGAGCAATGGCAAAACGGTAAC

CCCCAGCGCTGGCACCTGACGCGAGACGATC

>151 bp product from linear template LR134141.1:1011173-1012180 Salmonella enterica subsp. salamae strain NCTC5773 genome assembly, chromosome: 1, base 802 to base 952 (Forward invj - Reverse invj).

CAGCGCTGGGGAAATGACTATTCCGTCAATATTCAGGCGCGGCAAGTGGGGGAGTTTTCG

TTGATACCGTCAAATACGCAGGTTGAACATCGTTTGCATGATCAATGGCAAAACGGTAAT

CCACAGCGCTGGCACCTGACGCGAGACGATC

>151 bp product from linear template CP079837.1:960740-961747 Salmonella enterica subsp. salamae strain LHICA_SA2 chromosome, complete genome, base 802 to base 952 (Forward invj - Reverse invj).

CAGCGCTGGGGAAATGACTATTCCGTCAATATTCAGACGCGGCAAGTGGGGGAGTTTTCG

TTAATACCGTCAAATACGCAGGTTGAACATCGTTTGCATGATCAATGGCAAAACGGTAAT

CCCCAGCGCTGGCACCTGACGCGAGACGATC

>151 bp product from linear template LS483475.1:1016577-1017584 Salmonella enterica subsp. salamae serovar Greenside strain NCTC9936 genome assembly, chromosome: 1, base 802 to base 952 (Forward invj - Reverse invj).

CAGCGCTGGGGAAATGACTATTCCGTCAATATTCAGACGCGGCAAGTGGGGGAGTTTTCG

TTAATACCGTCAAATACGCAGGTTGAACATCGTTTGCATGATCAATGGCAAAACGGTAAT

CCCCAGCGCTGGCACCTGACGCGAGACGATC

>151 bp product from linear template CP053318.1:c3801419-3800412 Salmonella enterica subsp. salamae serovar 6,8:a:z52 strain 62-3163 chromosome, complete genome, base 802 to base 952 (Forward invj - Reverse invj).

CAGCGCTGGGGAAATGACTATTCGGTCAATATTCAGGCGCGCCAGGTAGGGGAGTTTTCG

TTAATACCGTCAAATACGCAGGTTGAACATCGTTTGCATGATCAATGGCAAAACGGTAAC

CCCCAGCGCTGGCACCTGACGCGAGACGATC

>151 bp product from linear template CP074598.1:956296-957303 Salmonella enterica subsp. salamae strain CFSAN001013 chromosome, complete genome, base 802 to base 952 (Forward invj - Reverse invj).

CAGCGCTGGGGAAATGACTATTCCGTCAATATTCAGGCGCGGCAAGTGGGGGAGTTTTCG

TTAATACCGTCAAATACGCAGGTTGAACATCGTTTGCATGATCAATGGCAAAACGGTAAT

CCCCAGCGCTGGCACCTGACGCGAGACGATC

>151 bp product from linear template CP029992.1:3136431-3137438 Salmonella enterica subsp. salamae serovar 56:z10:e,n,x strain SA20011914 chromosome, complete genome, base 802 to base 952 (Forward invj - Reverse invj).

CAGCGCTGGGGAAATGACTATTCCGTCAATATTCAGGCGCGGCAAGTGGGGGAGTTTTCG

TTAATACCGTCAAATACGCAGGTTGAACATCGTTTGCATGATCAATGGCAAAACGGTAAT

CCCCAGCGCTGGCACCTGACGCGAGACGATC

>151 bp product from linear template CP079836.1:1127384-1128391 Salmonella enterica subsp. salamae strain LHICA_SA1 chromosome, complete genome, base 802 to base 952 (Forward invj - Reverse invj).

CAGCGCTGGGGAAATGACTATTCCGTCAATATTCAGGCGCGGCAAGTGGGGGAGTTTTCG

TTAATACCGTCAAATACGCAGGTTGAACATCGTTTGCATGATCAATGGCAAAACGGTAAT

CCCCAGCGCTGGCACCTGACGCGAGACGATC

>151 bp product from linear template CP079838.1:1146463-1147470 Salmonella enterica subsp. salamae strain LHICA_SA3 chromosome, complete genome, base 802 to base 952 (Forward invj - Reverse invj).

CAGCGCTGGGGAAATGACTATTCCGTCAATATTCAGGCGCGGCAAGTGGGGGAGTTTTCG

TTAATACCGTCAAATACGCAGGTTGAACATCGTTTGCATGATCAATGGCAAAACGGTAAT

CCCCAGCGCTGGCACCTGACGCGAGACGATC

>151 bp product from linear template CP022467.1:1054228-1055235 Salmonella enterica subsp. salamae serovar 57:z29:z42 strain ST114, complete genome, base 802 to base 952 (Forward invj - Reverse invj).

CAGCGCTGGGGAAATGACTATTCCGTCAATATTCAGGCGCGGCAAGTGGGGGAGTTTTCG

TTAATACCGTCAAATACGCAGGTTGAACATCGTTTGCATGATCAATGGCAAAACGGTAAT

CCCCAGCGCTGGCACCTGACGCGAGACGATC

>151 bp product from linear template CP074599.1:1042692-1043699 Salmonella enterica subsp. salamae strain CFSAN001011 chromosome, complete genome, base 802 to base 952 (Forward invj - Reverse invj).

CAGCGCTGGGGAAATGACTATTCCGTCAATATTCAGGCGCGGCAAGTGGGGGAGTTTTCG

TTAATACCGTCAAATACGCAGGTTGAACATCGTTTGCATGATCAATGGCAAAACGGTAAT

CCCCAGCGCTGGCACCTGACGCGAGACGATC

>151 bp product from linear template CP053326.1:1416883-1417890 Salmonella enterica subsp. salamae serovar 58:c:z6 strain 2013K-0366 chromosome, complete genome, base 802 to base 952 (Forward invj - Reverse invj).

CAGCGCTGGGGAAATGACTATTCCGTCAATATTCAGGCGCGGCAAGTGGGGGAGTTTTCA

TTAATACCGTCAAATACGCAGGTTGAACATCGTTTGCATGATCAATGGCAAAACGGTAAT

CCCCAGCGCTGGCACCTGACGCGAGACGATC

>151 bp product from linear template CP074601.1:1059609-1060616 Salmonella enterica subsp. salamae strain CFSAN001010 chromosome, complete genome, base 802 to base 952 (Forward invj - Reverse invj).

CAGCGCTGGGGAAATGACTATTCCGTCAATATTCAGGCGCGGCAAGTGGGGGAGTTTTCG

TTAATACCGTCAAATACGCAGGTTGAACATCGTTTGCATGATCAATGGCAAAACGGTAAT

CCCCAGCGCTGGCACCTGACGCGAGACGATC

>151 bp product from linear template CP029995.1:1075245-1076252 Salmonella enterica subsp. salamae serovar 56:b:[1,5] strain SA20053897 chromosome, complete genome, base 802 to base 952 (Forward invj - Reverse invj).

CAGCGCTGGGGAAATGACTATTCCGTCAATATTCAGGCGCGGCAAGTGGGGGAGTTTTCG

TTAATACCGTCAAATACGCAGGTTGAACATCGTTTGCATGATCAATGGCAAAACGGTAAT

CCCCAGCGCTGGCACCTGACGCGAGACGATC

>151 bp product from linear template CP053322.1:2704033-2705040 Salmonella enterica subsp. salamae serovar 48:z81:z39 strain 2015K-0023 chromosome, complete genome, base 802 to base 952 (Forward invj - Reverse invj).

CAGCGCTGGGGAAATGACTATTCCGTCAATATTCAGGCGCGGCAGGTGGGGGAGTTTTCG

TTAATACCGTCAAATACGCAAGTTGAACATCGTTTGCATGATCAATGGCAAAACGGAAAT

CCCCATCGTTGGCACCTGGCGCGAGACGATC

>151 bp product from linear template CP053330.1:1129766-1130773 Salmonella enterica subsp. salamae serovar 60:z10:z39 strain 2011K-1889 chromosome, complete genome, base 802 to base 952 (Forward invj - Reverse invj).

CAGCGCTGGGGAAATGACTATTCCGTCAATATTCAGGCGCGGCAGGTGGGGGAGTTTTCG

TTAATACCGTCAAATACGCAAGTTGAACATCGTTTGCATGATCAATGGCAAAACGGAAAT

CCCCATCGTTGGCACCTGGCGCGAGACGATC

>151 bp product from linear template U43303.1:444-1451 Salmonella enterica invasion gene complex SpaM and SpaN genes, complete cds, base 802 to base 952 (Forward invj - Reverse invj).

CAGCGCTGGGGAAATGACTATTCCGTCAATATTCAGGCGCGGCAAGTGGGGGAGTTTTCG

TTAATACCGTCAAATACGCAGGTTGAACATCGTTTGCATGATCAATGGCAAAACGGTAAT

CCCCAGCGCTGGCACCTGACGCGAGACGATC

>151 bp product from linear template LS483477.1:1068005-1069012 Salmonella enterica subsp. salamae strain NCTC10310 genome assembly, chromosome: 1, base 802 to base 952 (Forward invj - Reverse invj).

CAGCGCTGGGGAAATGACTATTCCGTCAATATTCAGGCGCGGCAAGTGGGGGAGTTTTCG

TTAATACCGTCAAATACGCAGGTTGAACATCGTTTGCATGATCAATGGCAAAACGGTAAT

CCCCAGCGCTGGCACCTGACGCGAGACGATC

>151 bp product from linear template U43302.1:444-1451 Salmonella enterica invasion gene complex SpaM and SpaN genes, complete cds, base 802 to base 952 (Forward invj - Reverse invj).

CAGCGCTGGGGAAATGACTATTCCGTCAATATTCAGGCGCGGCAAGTGGGGGAGTTTTCG

TTAATACCGTCAAATACGCAGGTTGAACATCGTTTGCATGATCAATGGCAAAACGGTAAT

CCCCAGCGCTGGCACCTGACGCGAGACGATC

>151 bp product from linear template CP100412.1:2661325-2662332 Salmonella enterica subsp. salamae serovar 42:f,g,t:-- strain RKS2986 chromosome, complete genome, base 802 to base 952 (Forward invj - Reverse invj).

CAGCGCTGGGGAAATGACTATTCCGTCAATATTCAGGCGCGGCAAGTGGGGGAGTTTTCG

TTAATACCGTCAAATACGCAGGTTGAACATCGTTTGCATGATCAATGGCAAAACGGTAAT

CCCCAGCGCTGGCACCTGACGCGAGACGATC

>151 bp product from linear template U43313.1:444-1448 Salmonella enterica invasion gene complex SpaM and SpaN genes, complete cds, base 799 to base 949 (Forward invj - Reverse invj).

CAGCGCTGGGGAAATGACTACTCCGTCAATATTCAGGCGCGGCAGGTAGGGGAGTTTTCG

TTAATACCGTCAAATACGCAGGTTGAGCATCGCTTGCACGATCAATGGCAAAACGGTAAC

CCCCAGCGCTGGCACCTGACGCGAGATGATC

>151 bp product from linear template U43312.1:444-1448 Salmonella enterica invasion gene complex SpaM and SpaN genes, complete cds, base 799 to base 949 (Forward invj - Reverse invj).

CAGCGCTGGGGAAATGACTACTCCGTCAATATTCAGGCGCGGCAGGTAGGGGAGTTTTCG

TTAATACCGTCAAATACGCAGGTTGAGCATCGCTTGCACGATCAATGGCAAAACGGTAAC

CCCCAGCGCTGGCACCTGACGCGAGATGATC

>151 bp product from linear template LR134156.1:1020661-1021668 Salmonella enterica subsp. arizonae strain NCTC10047 genome assembly, chromosome: 1, base 802 to base 952 (Forward invj - Reverse invj).

CAGCGCTGGGGAAATGACTATTCAGTCAATATTCAGGCGCGCCAGGTAGGGGAGTTTTCG

TTAATACCGTCAAATACGCAGGTTGAGCATCGTTTGCATGATCAATGGCAAAACGGTAAC

CCCCAGCGCTGGCACCTGACGCGAGACGATC

>151 bp product from linear template FJ496648.1:c30865-29858 Salmonella enterica subsp. salamae serovar Sofia pathogenicity island 1, complete sequence, base 802 to base 952 (Forward invj - Reverse invj).

CAGCGCTGGGGAAATGACTATTCCGTCAATATTCAGGCGCGGCAAGTGGGGGAGTTTTCG

TTAATACCGTCAAATACGCAGGTTGAACATCGTTTGCATGATCAATGGCAAAACGGTAAT

CCCCAGCGCTGGCACCTGACGCGAGACGATC

>151 bp product from linear template CP074594.1:1092873-1093880 Salmonella enterica subsp. salamae strain CFSAN001016 chromosome, complete genome, base 802 to base 952 (Forward invj - Reverse invj).

CAGCGCTGGGGAAATGACTATTCCGTCAATATTCAGGCGCGGCAAGTGGGGGAGTTTTCG

TTAATACCGTCAAATACGCAGGTTGAACATCGTTTGCATGATCAATGGCAAAACGGTAAT

CCCCAGCGCTGGCACCTGACGCGAGACGATC

>151 bp product from linear template CP034702.1:1072071-1073078 Salmonella enterica subsp. salamae serovar 42:r:- strain RSE36 chromosome, complete genome, base 802 to base 952 (Forward invj - Reverse invj).

CAGCGCTGGGGAAATGACTATTCCGTCAATATTCAGACGCGGCAAGTGGGGGAGTTTTCG

TTAATACCGTCAAATACGCAGGTTGAACATCGTTTGCATGATCAATGGCAAAACGGTAAT

CCCCAGCGCTGGCACCTGACGCGAGACGATC

>151 bp product from linear template CP034717.1:1072644-1073651 Salmonella enterica subsp. salamae serovar 42:r:- strain RSE09 chromosome, complete genome, base 802 to base 952 (Forward invj - Reverse invj).

CAGCGCTGGGGAAATGACTATTCCGTCAATATTCAGACGCGGCAAGTGGGGGAGTTTTCG

TTAATACCGTCAAATACGCAGGTTGAACATCGTTTGCATGATCAATGGCAAAACGGTAAT

CCCCAGCGCTGGCACCTGACGCGAGACGATC

>151 bp product from linear template CP034697.1:1073190-1074197 Salmonella enterica subsp. salamae serovar 42:r:- strain RSE42 chromosome, complete genome, base 802 to base 952 (Forward invj - Reverse invj).

CAGCGCTGGGGAAATGACTATTCCGTCAATATTCAGACGCGGCAAGTGGGGGAGTTTTCG

TTAATACCGTCAAATACGCAGGTTGAACATCGTTTGCATGATCAATGGCAAAACGGTAAT

CCCCAGCGCTGGCACCTGACGCGAGACGATC

>151 bp product from linear template CP079713.1:4114698-4115702 Salmonella enterica subsp. arizonae strain LHICA_AZ23 chromosome, complete genome, base 799 to base 949 (Forward invj - Reverse invj).

CAGCGCTGGGGAAATGACTACTCCGTCAATATTCAGGCGCGGCAGGTAGGGGAGTTTTCG

TTAATACCGTCAAATACGCAGGTTGAGCATCGCTTGCACGATCAATGGCAAAACGGTAAC

CCCCAGCGCTGGCACCTGACGCGAGATGATC

>151 bp product from linear template CP053401.1:c3210768-3209764 Salmonella enterica subsp. arizonae serovar 13:g,z51:- strain 2009K0967 chromosome, complete genome, base 799 to base 949 (Forward invj - Reverse invj).

CAGCGCTGGGGAAATGACTACTCCGTCAATATTCAGGCGCGGCAGGTAGGGGAGTTTTCG

TTAATACCGTCAAATACGCAGGTTGAGCATCGCTTGCACGATCAATGGCAAAACGGTAAC

CCCCAGCGCTGGCACCTGACGCGAGATGATC

>151 bp product from linear template CP064363.1:c1571012-1570008 Salmonella enterica subsp. arizonae strain PartD-Sarizonae-RM8376 chromosome, complete genome, base 799 to base 949 (Forward invj - Reverse invj).

CAGCGCTGGGGAAATGACTACTCCGTCAATATTCAGGCGCGGCAGGTAGGGGAGTTTTCG

TTAATACCGTCAAATACGCAGGTTGAGCATCGCTTGCACGATCAATGGCAAAACGGTAAC

CCCCAGCGCTGGCACCTGACGCGAGATGATC

>151 bp product from linear template CP006693.1:78603-79607 Salmonella enterica subsp. arizonae serovar 62:z36:- str. RKS2983, complete genome, base 799 to base 949 (Forward invj - Reverse invj).

CAGCGCTGGGGAAATGACTACTCCGTCAATATTCAGGCGCGGCAGGTAGGGGAGTTTTCG

TTAATACCGTCAAATACGCAGGTTGAGCATCGCTTGCACGATCAATGGCAAAACGGTAAC

CCCCAGCGCTGGCACCTGACGCGAGATGATC

>151 bp product from linear template CP022504.1:3362006-3363010 Salmonella enterica subsp. arizonae serovar 53:-:- str. SA20100345 chromosome, complete genome, base 799 to base 949 (Forward invj - Reverse invj).

CAGCGCTGGGGAAATGACTACTCCGTCAATATTCAGGCGCGGCAGGTAGGGGAGTTTTCG

TTAATACCGTCAAATACGCAGGTTGAGCATCGCTTGCACGATCAATGGCAAAACGGTAAC

CCCCAGCGCTGGCACCTGACGCGAGATGATC

>151 bp product from linear template CP029991.1:908426-909430 Salmonella enterica subsp. arizonae serovar 63:g,z51:- strain SA19981204 chromosome, complete genome, base 799 to base 949 (Forward invj - Reverse invj).

CAGCGCTGGGGAAATGACTACTCCGTCAATATTCAGGCGCGGCAGGTAGGGGAGTTTTCG

TTAATACCGTCAAATACGCAGGTTGAGCATCGCTTGCACGATCAATGGCAAAACGGTAAC

CCCCAGCGCTGGCACCTGACGCGAGATGATC

>151 bp product from linear template CP000880.1:78735-79739 Salmonella enterica subsp. arizonae serovar 62:z4,z23:- strain RSK2980 chromosome, complete genome, base 799 to base 949 (Forward invj - Reverse invj).

CAGCGCTGGGGAAATGACTACTCCGTCAATATTCAGGCGCGGCAGGTAGGGGAGTTTTCG

TTAATACCGTCAAATACGCAGGTTGAGCATCGCTTGCACGATCAATGGCAAAACGGTAAC

CCCCAGCGCTGGCACCTGACGCGAGATGATC

>151 bp product from linear template CP074593.1:921617-922621 Salmonella enterica subsp. arizonae serovar 41:z4,z23:- str. 01-0089 chromosome, complete genome, base 799 to base 949 (Forward invj - Reverse invj).

CAGCGCTGGGGAAATGACTACTCCGTCAATATTCAGGCGCGGCAGGTAGGGGAGTTTTCG

TTAATACCGTCAAATACGCAGGTTGAGCATCGCTTGCACGATCAATGGCAAAACGGTAAC

CCCCAGCGCTGGCACCTGACGCGAGATGATC

>151 bp product from linear template CP053321.1:1892317-1893321 Salmonella enterica subsp. arizonae serovar 41:z4,z23:- strain 2016K-0011 chromosome, complete genome, base 799 to base 949 (Forward invj - Reverse invj).

CAGCGCTGGGGAAATGACTACTCCGTCAATATTCAGGCGCGGCAGGTAGGGGAGTTTTCG

TTAATACCGTCAAATACGCAGGTTGAGCATCGCTTGCACGATCAATGGCAAAACGGTAAC

CCCCAGCGCTGGCACCTGACGCGAGATGATC

>151 bp product from linear template LR134150.1:915499-916503 Salmonella enterica subsp. arizonae strain NCTC7306 genome assembly, chromosome: 1, base 799 to base 949 (Forward invj - Reverse invj).

CAGCGCTGGGGAAATGACTACTCCGTCAATATTCAGGCGCGGCAGGTAGGGGAGTTTTCG

TTAATACCGTCAAATACGCAGGTTGAGCATCGCTTGCACGATCAATGGCAAAACGGTAAC

CCCCAGCGCTGGCACCTGACGCGAGATGATC

>151 bp product from linear template LR133910.1:917846-918850 Salmonella enterica subsp. arizonae strain NCTC7300 genome assembly, chromosome: 1, base 799 to base 949 (Forward invj - Reverse invj).

CAGCGCTGGGGAAATGACTACTCCGTCAATATTCAGGCGCGGCAGGTAGGGGAGTTTTCG

TTAATACCGTCAAATACGCAGGTTGAGCATCGCTTGCACGATCAATGGCAAAACGGTAAC

CCCCAGCGCTGGCACCTGACGCGAGATGATC

>151 bp product from linear template LR134154.1:1061246-1061845 Salmonella enterica subsp. salamae strain NCTC8273 genome assembly, chromosome: 1, base 394 to base 544 (Forward invj - Reverse invj).

CAGCGCTGGGGAAATGACTATTCCGTCAATATTCAGACGCGGCAAGTGGGGGAGTTTTCG

TTAATACCGTCAAATACGCAGGTTGAACATCGTTTGCATGATCAATGGCAAAACGGTAAT

CCCCAGCGCTGGCACCTGACGCGAGACGATC

>151 bp product from linear template U43308.1:444-1451 Salmonella enterica invasion gene complex SpaN gene, complete cds, base 802 to base 952 (Forward invj - Reverse invj).

CAGCGCTGGGGAAATGACTATTCCGTCAATATTCAGGCGCGGCAGGCGGGGGAGTTTTCG

CTAATACCGTCAAATACGCAGGTTGAGCATCGCTTGCATGATCAATGGCAAAACGGTAAT

CCCCAGCGCTGGCACCTGATGCGAGACGATC

>151 bp product from linear template LS483478.1:921975-922982 Salmonella enterica subsp. houtenae serovar Houten strain NCTC10401 genome assembly, chromosome: 1, base 802 to base 952 (Forward invj - Reverse invj).

CAGCGCTGGGGAAATGACTATTCCGTCAATATTCAGGCACGGCAGGCGGGGGAGTTTTCG

TTAATACCGTCAAATACGCAGGTTGAGCATCGCTTGCATGATCAATGGCAAAACGGTAAT

CCCCAGCGCTGGCACCTGATGCGAGATGATC

>151 bp product from linear template CP053332.1:927063-928070 Salmonella enterica subsp. houtenae serovar 44:z4,z32:- strain 2009K1701 chromosome, complete genome, base 802 to base 952 (Forward invj - Reverse invj).

CAGCGCTGGGGAAATGACTATTCCGTCAATATTCAGGCACGGCAGGCGGGGGAGTTTTCG

TTAATACCGTCAAATACGCAGGTTGAACATCGCTTGCATGATCAATGGCAAAACGGTAAT

CCCCAGCGCTGGCACCTGATGCGAGATGATC

>151 bp product from linear template CP051368.1:938210-939217 Salmonella enterica subsp. houtenae serovar 43:z4 strain CVM 24399 chromosome, complete genome, base 802 to base 952 (Forward invj - Reverse invj).

CAGCGCTGGGGAAATGACTATTCCGTCAATATTCAGGCACGGCAGGCGGGGGAGTTTTCG

TTAATACCGTCAAATACGCAGGTTGAACATCGCTTGCATGATCAATGGCAAAACGGTAAT

CCCCAGCGCTGGCACCTGATGCGAGATGATC

>151 bp product from linear template CP045761.1:c594804-593797 Salmonella enterica subsp. houtenae str. CFSAN000552 isolate SARC10 chromosome, complete genome, base 802 to base 952 (Forward invj - Reverse invj).

CAGCGCTGGGGAAATGACTATTCCGTCAATATTCAGGCACGGCAGGCGGGGGAGTTTTCG

TTAATACCGTCAAATACGCAGGTTGAACATCGCTTGCATGATCAATGGCAAAACGGTAAT

CCCCAGCGCTGGCACCTGATGCGAGATGATC

>151 bp product from linear template CP100411.1:2392052-2393059 Salmonella enterica subsp. houtenae serovar 16:z4,z32:-- strain RKS3027 chromosome, complete genome, base 802 to base 952 (Forward invj - Reverse invj).

CAGCGCTGGGGAAATGACTATTCCGTCAATATTCAGGCACGGCAGGCGGGGGAGTTTTCG

TTAATACCGTCAAATACGCAGGTTGAACATCGCTTGCATGATCAATGGCAAAACGGTAAT

CCCCAGCGCTGGCACCTGATGCGAGATGATC

>151 bp product from linear template LR134159.1:947197-948204 Salmonella enterica subsp. houtenae strain NCTC7318 genome assembly, chromosome: 1, base 802 to base 952 (Forward invj - Reverse invj).

CAGCGCTGGGGAAATGACTATTCCGTCAATATTCAGGCACGGCAGGCGGGGGAGTTTTCG

TTAATACCGTCAAATACGCAGGTTGAACATCGCTTGCATGATCAATGGCAAAACGGTAAT

CCCCAGCGCTGGCACCTGATGCGAGATGATC

>151 bp product from linear template U43309.1:444-1472 Salmonella enterica invasion gene complex SpaM and SpaN genes, complete cds, base 823 to base 973 (Forward invj - Reverse invj).

CAGCGCTGGGGAAATGACTATTCCGTCAATATTCAGGCACGGCAGGCGGGGGAGTTTTCG

TTAATACCGTCAAATACGCAGGTTGAACATCGCTTGCATGATCAATGGCAAAACGGTAAT

CCCCAGCGCTGGCACCTGATGCGAGATGATC

>151 bp product from linear template CP075174.1:941833-942861 Salmonella enterica subsp. houtenae serovar 45:g,z51:- strain 20-369 chromosome, complete genome, base 823 to base 973 (Forward invj - Reverse invj).

CAGCGCTGGGGAAATGACTATTCCGTCAATATTCAGGCACGGCAGGCGGGGGAGTTTTCG

TTAATACCGTCAAATACGCAGGTTGAACATCGCTTGCATGATCAATGGCAAAACGGTAAT

CCCCAGCGCTGGCACCTGATGCGAGATGATC

>151 bp product from linear template CP100413.1:2468037-2469065 Salmonella enterica subsp. houtenae serovar 1,40:g,z51:- strain RKS3013 chromosome, complete genome, base 823 to base 973 (Forward invj - Reverse invj).

CAGCGCTGGGGAAATGACTATTCCGTCAATATTCAGGCGCGGCAGGCGGGGGAGTTTTCG

TTAATACCGTCAAATACGCAGGTTGAGCATCGCTTGCATGATCAATGGCAAAACGGTAAT

CCCCAGCGCTGGCACCTGATGCGAGATGATC

>151 bp product from linear template U43315.1:444-1442 Salmonella enterica invasion gene complex SpaM and SpaN genes, complete cds, base 793 to base 943 (Forward invj - Reverse invj).

CAGCGCTGGGGAAATGACTATTCCGTCAATATTCAGGCGCGGCAGGCAGGGGAGTTTTCG

TTAGTACCGTCAAATACGCAGGTTGAGCATCGTTTGCATGATCAATGGCAGAACGGTAAC

CCTCAGCGCTGGCACCTGATGCGAGACGATC

>151 bp product from linear template U43314.1:444-1442 Salmonella enterica invasion gene complex SpaM and SpaN genes, complete cds, base 793 to base 943 (Forward invj - Reverse invj).

CAGCGCTGGGGAAATGACTACTCCGTCAATATTCAGGCGCGGCAGGCAGGGGAGTTTTCG

TTAATACCGTCAAATACGCAGGTTGAGCATCGTTTGCATGATCAATGGCAGAACGGTAAC

CCTCAGCGCTGGCACCTGATGCGAGACGATC

>151 bp product from linear template CP074592.1:941698-942696 Salmonella bongori serovar 40:z35:- strain CFSAN001045 chromosome, complete genome, base 793 to base 943 (Forward invj - Reverse invj).

CAGCGCTGGGGAAATGACTATTCCGTCAATATTCAGGCGCGGCAGGCAGGGGAGTTTTCG

TTAGTACCGTCAAATACGCAGGTTGAGCATCGTTTGCATGATCAATGGCAGAACGGTAAC

CCTCAGCGCTGGCACCTGATGCGAGACGATC

>151 bp product from linear template CP035676.1:c417637-416639 Salmonella bongori strain 04-0440 chromosome, complete genome, base 793 to base 943 (Forward invj - Reverse invj).

CAGCGCTGGGGAAATGACTATTCCGTCAATATTCAGGCGCGGCAGGCAGGGGAGTTTTCG

TTAGTACCGTCAAATACGCAGGTTGAGCATCGTTTGCATGATCAATGGCAGAACGGTAAC

CCTCAGCGCTGGCACCTGATGCGAGACGATC

>151 bp product from linear template CP006692.1:c2626282-2625284 Salmonella bongori serovar 48:z41:-- str. RKS3044, complete genome, base 793 to base 943 (Forward invj - Reverse invj).

CAGCGCTGGGGAAATGACTATTCCGTCAATATTCAGGCGCGGCAGGCAGGGGAGTTTTCG

TTAGTACCGTCAAATACGCAGGTTGAGCATCGTTTGCATGATCAATGGCAGAACGGTAAC

CCTCAGCGCTGGCACCTGATGCGAGACGATC

>151 bp product from linear template CP045766.1:1977914-1978912 Salmonella bongori CFSAN000510 chromosome, complete genome, base 793 to base 943 (Forward invj - Reverse invj).

CAGCGCTGGGGAAATGACTACTCCGTCAATATTCAGGCGCGGCAGGCAGGGGAGTTTTCG

TTAATACCGTCAAATACGCAGGTTGAGCATCGTTTGCATGATCAATGGCAGAACGGTAAC

CCTCAGCGCTGGCACCTGATGCGAGACGATC

>151 bp product from linear template CP067369.1:976340-977338 Salmonella bongori strain Se40 chromosome, complete genome, base 793 to base 943 (Forward invj - Reverse invj).

CAGCGCTGGGGAAATGACTACTCCGTCAATATTCAGGCGCGGCAGGCAGGGGAGTTTTCG

TTAATACCGTCAAATACGCAGGTTGAGCATCGTTTGCATGATCAATGGCAGAACGGTAAC

CCTCAGCGCTGGCACCTGATGCGAGACGATC

>151 bp product from linear template CP074233.1:976331-977329 Salmonella bongori CFSAN000510 strain SGSC 3100 chromosome, complete genome, base 793 to base 943 (Forward invj - Reverse invj).

CAGCGCTGGGGAAATGACTACTCCGTCAATATTCAGGCGCGGCAGGCAGGGGAGTTTTCG

TTAATACCGTCAAATACGCAGGTTGAGCATCGTTTGCATGATCAATGGCAGAACGGTAAC

CCTCAGCGCTGGCACCTGATGCGAGACGATC

>151 bp product from linear template FR877557.1:c2755846-2754848 Salmonella bongori NCTC 12419, culture collection SGSC SARC11, complete genome, base 793 to base 943 (Forward invj - Reverse invj).

CAGCGCTGGGGAAATGACTACTCCGTCAATATTCAGGCGCGGCAGGCAGGGGAGTTTTCG

TTAATACCGTCAAATACGCAGGTTGAGCATCGTTTGCATGATCAATGGCAGAACGGTAAC

CCTCAGCGCTGGCACCTGATGCGAGACGATC

>151 bp product from linear template CP022120.1:976332-977330 Salmonella bongori serovar 66:z41:- str. SA19983605, complete genome, base 793 to base 943 (Forward invj - Reverse invj).

CAGCGCTGGGGAAATGACTACTCCGTCAATATTCAGGCGCGGCAGGCAGGGGAGTTTTCG

TTAATACCGTCAAATACGCAGGTTGAGCATCGTTTGCATGATCAATGGCAGAACGGTAAC

CCTCAGCGCTGGCACCTGATGCGAGACGATC

>151 bp product from linear template CP053416.1:c856376-855378 Salmonella bongori strain 85-0051 chromosome, complete genome, base 793 to base 943 (Forward invj - Reverse invj).

CAGCGCTGGGGAAATGACTATTCCGTCAATATTCAGGCGCGGCAGGCAGGGGAGTTTTCG

TTAGTACCGTCAAATACGCAGGTTGAGCATCGTTTGCATGATCAATGGCAGAACGGTAAC

CCTCAGCGCTGGCACCTGATGCGAGACGATC

>151 bp product from linear template CP006608.1:c2902597-2901599 Salmonella bongori N268-08, complete genome, base 793 to base 943 (Forward invj - Reverse invj).

CAGCGCTGGGGAAATGACTATTCCGTCAATATTCAGGCGCGGCAGGCAGGGGAGTTTTCG

TTAGTACCGTCAAATACGCAGGTTGAGCATCGTTTGCATGATCAATGGCAGAACGGTAAC

CCTCAGCGCTGGCACCTGATGCGAGACGATC

>151 bp product from linear template CP053336.1:3665341-3666339 Salmonella bongori serovar 48:z81:- strain 08-0158 chromosome, complete genome, base 793 to base 943 (Forward invj - Reverse invj).

CAGCGCTGGGGAAATGACTATTCCGTCAATATTCAGGCGCGGCAGGCAGGGGAGTTTTCG

TTAGTACCGTCAAATACGCAGGTTGAGCATCGTTTGCATGATCAATGGCAGAACGGTAAC

CCTCAGCGCTGGCACCTGATGCGAGACGATC

>151 bp product from linear template CP053417.1:2750711-2751709 Salmonella bongori strain 92-0238 chromosome, complete genome, base 793 to base 943 (Forward invj - Reverse invj).

CAGCGCTGGGGAAATGACTATTCCGTCAATATTCAGGCGCGGCAGGCAGGGGAGTTTTCG

TTAGTACCGTCAAATACGCAGGTTGAGCATCGTTTGCATGATCAATGGCAGAACGGTAAC

CCTCAGCGCTGGCACCTGATGCGAGACGATC

>151 bp product from linear template X73525.1:1430-2437 S.enterica Typhimurium genes for surface presentation of antigens, base 802 to base 952 (Forward invj - Reverse invj).

CAGCGCTGGGGAAATGACTATTCCGTCAATATTCAGGCGCGGCAAGCAGGGGAGTTTTCG

TTAATACCGTCAAATACGCAGGTTGAACATCGTTTGCATGATCAATGGCAAAACGGTAAT

CCCCAGCGCTGGCACCTGACGCGAGACGATC

>151 bp product from linear template CP074306.1:1030480-1031487 Salmonella enterica subsp. enterica strain CFSAN004114 chromosome, complete genome, base 802 to base 952 (Forward invj - Reverse invj).

CAGCGCTGGGGAAATGACTATTCCGTCAATATTCAGGCGCGGCAAGCAGGGGAGTTTTCG

TTAATACCGTCAAATACGCAGGTTGAACATCGTTTGCATGATCAATGGCAAAACGGTAAT

CCCCAGCGCTGGCACCTGACGCGAGACGATC

>151 bp product from linear template CP150844.1:c3571553-3570546 Salmonella enterica subsp. enterica serovar Typhimurium strain Z1323HSL0023 chromosome, complete genome, base 802 to base 952 (Forward invj - Reverse invj).

CAGCGCTGGGGAAATGACTATTCCGTCAATATTCAGGCGCGGCAAGCAGGGGAGTTTTCG

TTAATACCGTCAAATACGCAGGTTGAACATCGTTTGCATGATCAATGGCAAAACGGTAAT

CCCCAGCGCTGGCACCTGACGCGAGACGATC

>151 bp product from linear template CP065125.1:1002504-1003511 Salmonella enterica subsp. enterica strain 172 chromosome, complete genome, base 802 to base 952 (Forward invj - Reverse invj).

CAGCGCTGGGGAAATGACTATTCCGTCAATATTCAGGCGCGGCAAGCAGGGGAGTTTTCG

TTAATACCGTCAAATACGCAGGTTGAACATCGTTTGCATGATCAATGGCAAAACGGTAAT

CCCCAGCGCTGGCACCTGACGCGAGACGATC

>151 bp product from linear template CP082662.1:1007747-1008754 Salmonella enterica subsp. enterica serovar Typhimurium var. 5- strain CVM N16S089 chromosome, complete genome, base 802 to base 952 (Forward invj - Reverse invj).

CAGCGCTGGGGAAATGACTATTCCGTCAATATTCAGGCGCGGCAAGCAGGGGAGTTTTCG

TTAATACCGTCAAATACGCAGGTTGAACATCGTTTGCATGATCAATGGCAAAACGGTAAT

CCCCAGCGCTGGCACCTGACGCGAGACGATC

>151 bp product from linear template CP133212.1:1088496-1089503 Salmonella enterica strain AJM4 chromosome, complete genome, base 802 to base 952 (Forward invj - Reverse invj).

CAGCGCTGGGGAAATGACTATTCCGTCAATATTCAGGCGCGGCAAGCAGGGGAGTTTTCG

TTAATACCGTCAAATACGCAGGTTGAACATCGTTTGCATGATCAATGGCAAAACGGTAAT

CCCCAGCGCTGGCACCTGACGCGAGACGATC

>151 bp product from linear template CP149356.1:559925-560932 Salmonella enterica subsp. enterica serovar Typhimurium strain Z1323HSL0075 chromosome, complete genome, base 802 to base 952 (Forward invj - Reverse invj).

CAGCGCTGGGGAAATGACTATTCCGTCAATATTCAGGCGCGGCAAGCAGGGGAGTTTTCG

TTAATACCGTCAAATACGCAGGTTGAACATCGTTTGCATGATCAATGGCAAAACGGTAAT

CCCCAGCGCTGGCACCTGACGCGAGACGATC

>151 bp product from linear template CP014965.1:1046891-1047898 Salmonella enterica subsp. enterica serovar Typhimurium str. CDC 2010K-1587 chromosome, complete genome, base 802 to base 952 (Forward invj - Reverse invj).

CAGCGCTGGGGAAATGACTATTCCGTCAATATTCAGGCGCGGCAAGCAGGGGAGTTTTCG

TTAATACCGTCAAATACGCAGGTTGAACATCGTTTGCATGATCAATGGCAAAACGGTAAT

CCCCAGCGCTGGCACCTGACGCGAGACGATC

>151 bp product from linear template CP033226.2:1039828-1040835 Salmonella enterica subsp. enterica strain CFSA122 chromosome, complete genome, base 802 to base 952 (Forward invj - Reverse invj).

CAGCGCTGGGGAAATGACTATTCCGTCAATATTCAGGCGCGGCAAGCAGGGGAGTTTTCG

TTAATACCGTCAAATACGCAGGTTGAACATCGTTTGCATGATCAATGGCAAAACGGTAAT

CCCCAGCGCTGGCACCTGACGCGAGACGATC

>151 bp product from linear template CP149365.1:c3626138-3625131 Salmonella enterica subsp. enterica serovar Typhimurium strain Z1323HSL0048 chromosome, complete genome, base 802 to base 952 (Forward invj - Reverse invj).

CAGCGCTGGGGAAATGACTATTCCGTCAATATTCAGGCGCGGCAAGCAGGGGAGTTTTCG

TTAATACCGTCAAATACGCAGGTTGAACATCGTTTGCATGATCAATGGCAAAACGGTAAT

CCCCAGCGCTGGCACCTGACGCGAGACGATC

>151 bp product from linear template CP149307.1:1008514-1009521 Salmonella enterica subsp. enterica serovar Typhimurium strain Z1323SSL0041 chromosome, complete genome, base 802 to base 952 (Forward invj - Reverse invj).

CAGCGCTGGGGAAATGACTATTCCGTCAATATTCAGGCGCGGCAAGCAGGGGAGTTTTCG

TTAATACCGTCAAATACGCAGGTTGAACATCGTTTGCATGATCAATGGCAAAACGGTAAT

CCCCAGCGCTGGCACCTGACGCGAGACGATC

>151 bp product from linear template CP132950.1:1498372-1499379 Salmonella enterica subsp. enterica serovar 4,12:i:- strain CFIAFB20160237 chromosome, complete genome, base 802 to base 952 (Forward invj - Reverse invj).

CAGCGCTGGGGAAATGACTATTCCGTCAATATTCAGGCGCGGCAAGCAGGGGAGTTTTCG

TTAATACCGTCAAATACGCAGGTTGAACATCGTTTGCATGATCAATGGCAAAACGGTAAT

CCCCAGCGCTGGCACCTGACGCGAGACGATC

>151 bp product from linear template CP034230.1:1002215-1003222 Salmonella enterica subsp. enterica serovar Typhimurium strain ATCC 14028 chromosome, complete genome, base 802 to base 952 (Forward invj - Reverse invj).

CAGCGCTGGGGAAATGACTATTCCGTCAATATTCAGGCGCGGCAAGCAGGGGAGTTTTCG

TTAATACCGTCAAATACGCAGGTTGAACATCGTTTGCATGATCAATGGCAAAACGGTAAT

CCCCAGCGCTGGCACCTGACGCGAGACGATC

>151 bp product from linear template CP162902.1:1120629-1121636 Salmonella enterica subsp. enterica strain 2742 chromosome, complete genome, base 802 to base 952 (Forward invj - Reverse invj).

CAGCGCTGGGGAAATGACTATTCCGTCAATATTCAGGCGCGGCAAGCAGGGGAGTTTTCG

TTAATACCGTCAAATACGCAGGTTGAACATCGTTTGCATGATCAATGGCAAAACGGTAAT

CCCCAGCGCTGGCACCTGACGCGAGACGATC

>151 bp product from linear template CP047522.1:c3902579-3901572 Salmonella enterica subsp. enterica serovar Typhimurium strain SJTUF11216 chromosome, complete genome, base 802 to base 952 (Forward invj - Reverse invj).

CAGCGCTGGGGAAATGACTATTCCGTCAATATTCAGGCGCGGCAAGCAGGGGAGTTTTCG

TTAATACCGTCAAATACGCAGGTTGAACATCGTTTGCATGATCAATGGCAAAACGGTAAT

CCCCAGCGCTGGCACCTGACGCGAGACGATC

>151 bp product from linear template CP043400.1:c3056495-3055488 Salmonella enterica subsp. enterica serovar Typhimurium str. 14028S substr. JY996 chromosome, complete genome, base 802 to base 952 (Forward invj - Reverse invj).

CAGCGCTGGGGAAATGACTATTCCGTCAATATTCAGGCGCGGCAAGCAGGGGAGTTTTCG

TTAATACCGTCAAATACGCAGGTTGAACATCGTTTGCATGATCAATGGCAAAACGGTAAT

CCCCAGCGCTGGCACCTGACGCGAGACGATC

>151 bp product from linear template CP149335.1:c3447430-3446423 Salmonella enterica subsp. enterica serovar Typhimurium strain Z1323SSL0010 chromosome, complete genome, base 802 to base 952 (Forward invj - Reverse invj).

CAGCGCTGGGGAAATGACTATTCCGTCAATATTCAGGCGCGGCAAGCAGGGGAGTTTTCG

TTAATACCGTCAAATACGCAGGTTGAACATCGTTTGCATGATCAATGGCAAAACGGTAAT

CCCCAGCGCTGGCACCTGACGCGAGACGATC

>151 bp product from linear template CP040458.1:1120625-1121632 Salmonella enterica subsp. enterica serovar Typhimurium strain TJWQ005 chromosome, complete genome, base 802 to base 952 (Forward invj - Reverse invj).

CAGCGCTGGGGAAATGACTATTCCGTCAATATTCAGGCGCGGCAAGCAGGGGAGTTTTCG

TTAATACCGTCAAATACGCAGGTTGAACATCGTTTGCATGATCAATGGCAAAACGGTAAT

CCCCAGCGCTGGCACCTGACGCGAGACGATC

>151 bp product from linear template CP039716.1:c2975157-2974150 Salmonella enterica subsp. enterica serovar 1,4,[5],12:i:- strain PNCS000211 chromosome, complete genome, base 802 to base 952 (Forward invj - Reverse invj).

CAGCGCTGGGGAAATGACTATTCCGTCAATATTCAGGCGCGGCAAGCAGGGGAGTTTTCG

TTAATACCGTCAAATACGCAGGTTGAACATCGTTTGCATGATCAATGGCAAAACGGTAAT

CCCCAGCGCTGGCACCTGACGCGAGACGATC

>151 bp product from linear template CP112994.1:4572575-4573582 Salmonella enterica subsp. enterica serovar Typhimurium strain MeganVac1 chromosome, base 802 to base 952 (Forward invj - Reverse invj).

CAGCGCTGGGGAAATGACTATTCCGTCAATATTCAGGCGCGGCAAGCAGGGGAGTTTTCG

TTAATACCGTCAAATACGCAGGTTGAACATCGTTTGCATGATCAATGGCAAAACGGTAAT

CCCCAGCGCTGGCACCTGACGCGAGACGATC

>151 bp product from linear template CP020565.1:999656-1000663 Salmonella enterica subsp. enterica serovar Typhimurium strain FORC58 chromosome, complete genome, base 802 to base 952 (Forward invj - Reverse invj).

CAGCGCTGGGGAAATGACTATTCCGTCAATATTCAGGCGCGGCAAGCAGGGGAGTTTTCG

TTAATACCGTCAAATACGCAGGTTGAACATCGTTTGCATGATCAATGGCAAAACGGTAAT

CCCCAGCGCTGGCACCTGACGCGAGACGATC

>151 bp product from linear template CP104366.1:1007151-1008158 Salmonella enterica subsp. enterica serovar Typhimurium strain PNUSAS028809 chromosome, complete genome, base 802 to base 952 (Forward invj - Reverse invj).

CAGCGCTGGGGAAATGACTATTCCGTCAATATTCAGGCGCGGCAAGCAGGGGAGTTTTCG

TTAATACCGTCAAATACGCAGGTTGAACATCGTTTGCATGATCAATGGCAAAACGGTAAT

CCCCAGCGCTGGCACCTGACGCGAGACGATC

>151 bp product from linear template CP019442.1:2939120-2940127 Salmonella enterica subsp. enterica serovar Typhimurium strain 81741, complete genome, base 802 to base 952 (Forward invj - Reverse invj).

CAGCGCTGGGGAAATGACTATTCCGTCAATATTCAGGCGCGGCAAGCAGGGGAGTTTTCG

TTAATACCGTCAAATACGCAGGTTGAACATCGTTTGCATGATCAATGGCAAAACGGTAAT

CCCCAGCGCTGGCACCTGACGCGAGACGATC

>151 bp product from linear template HF937208.1:c3071500-3070493 Salmonella enterica subsp. enterica serovar Typhimurium DT104 main chromosome, complete genome, base 802 to base 952 (Forward invj - Reverse invj).

CAGCGCTGGGGAAATGACTATTCCGTCAATATTCAGGCGCGGCAAGCAGGGGAGTTTTCG

TTAATACCGTCAAATACGCAGGTTGAACATCGTTTGCATGATCAATGGCAAAACGGTAAT

CCCCAGCGCTGGCACCTGACGCGAGACGATC

>151 bp product from linear template CP149291.1:535888-536895 Salmonella enterica subsp. enterica serovar Typhimurium strain Z1323SSL0073 chromosome, complete genome, base 802 to base 952 (Forward invj - Reverse invj).

CAGCGCTGGGGAAATGACTATTCCGTCAATATTCAGGCGCGGCAAGCAGGGGAGTTTTCG

TTAATACCGTCAAATACGCAGGTTGAACATCGTTTGCATGATCAATGGCAAAACGGTAAT

CCCCAGCGCTGGCACCTGACGCGAGACGATC

>151 bp product from linear template CP082577.1:c3942347-3941340 Salmonella enterica subsp. enterica serovar Typhimurium strain CVM N18S0981 chromosome, complete genome, base 802 to base 952 (Forward invj - Reverse invj).

CAGCGCTGGGGAAATGACTATTCCGTCAATATTCAGGCGCGGCAAGCAGGGGAGTTTTCG

TTAATACCGTCAAATACGCAGGTTGAACATCGTTTGCATGATCAATGGCAAAACGGTAAT

CCCCAGCGCTGGCACCTGACGCGAGACGATC

>151 bp product from linear template CP123924.1:1004388-1005395 Salmonella sp. SA15303 chromosome, complete genome, base 802 to base 952 (Forward invj - Reverse invj).

CAGCGCTGGGGAAATGACTATTCCGTCAATATTCAGGCGCGGCAAGCAGGGGAGTTTTCG

TTAATACCGTCAAATACGCAGGTTGAACATCGTTTGCATGATCAATGGCAAAACGGTAAT

CCCCAGCGCTGGCACCTGACGCGAGACGATC

>151 bp product from linear template CP022062.2:3677922-3678929 Salmonella enterica subsp. enterica serovar Typhimurium var. 5- strain FDAARGOS_312 chromosome, complete genome, base 802 to base 952 (Forward invj - Reverse invj).

CAGCGCTGGGGAAATGACTATTCCGTCAATATTCAGGCGCGGCAAGCAGGGGAGTTTTCG

TTAATACCGTCAAATACGCAGGTTGAACATCGTTTGCATGATCAATGGCAAAACGGTAAT

CCCCAGCGCTGGCACCTGACGCGAGACGATC

>151 bp product from linear template AP023292.1:1007955-1008962 Salmonella enterica subsp. enterica serovar 4,[5],12:i:- L-4233 DNA, complete genome, base 802 to base 952 (Forward invj - Reverse invj).

CAGCGCTGGGGAAATGACTATTCCGTCAATATTCAGGCGCGGCAAGCAGGGGAGTTTTCG

TTAATACCGTCAAATACGCAGGTTGAACATCGTTTGCATGATCAATGGCAAAACGGTAAT

CCCCAGCGCTGGCACCTGACGCGAGACGATC

>151 bp product from linear template AP023303.1:1040025-1041032 Salmonella enterica subsp. enterica serovar 4,[5],12:i:- L-4526 DNA, complete genome, base 802 to base 952 (Forward invj - Reverse invj).

CAGCGCTGGGGAAATGACTATTCCGTCAATATTCAGGCGCGGCAAGCAGGGGAGTTTTCG

TTAATACCGTCAAATACGCAGGTTGAACATCGTTTGCATGATCAATGGCAAAACGGTAAT

CCCCAGCGCTGGCACCTGACGCGAGACGATC

>151 bp product from linear template CP047323.1:c3056493-3055486 Salmonella enterica subsp. enterica serovar Typhimurium strain RM13672 chromosome, complete genome, base 802 to base 952 (Forward invj - Reverse invj).

CAGCGCTGGGGAAATGACTATTCCGTCAATATTCAGGCGCGGCAAGCAGGGGAGTTTTCG

TTAATACCGTCAAATACGCAGGTTGAACATCGTTTGCATGATCAATGGCAAAACGGTAAT

CCCCAGCGCTGGCACCTGACGCGAGACGATC

>151 bp product from linear template CP060515.1:1036274-1037281 Salmonella enterica strain SLR1_8094 chromosome, complete genome, base 802 to base 952 (Forward invj - Reverse invj).

CAGCGCTGGGGAAATGACTATTCCGTCAATATTCAGGCGCGGCAAGCAGGGGAGTTTTCG

TTAATACCGTCAAATACGCAGGTTGAACATCGTTTGCATGATCAATGGCAAAACGGTAAT

CCCCAGCGCTGGCACCTGACGCGAGACGATC

>151 bp product from linear template CP149175.1:1039918-1040925 Salmonella enterica subsp. enterica serovar 4,[5],12:i:- strain Z1323SSL0051 chromosome, complete genome, base 802 to base 952 (Forward invj - Reverse invj).

CAGCGCTGGGGAAATGACTATTCCGTCAATATTCAGGCGCGGCAAGCAGGGGAGTTTTCG

TTAATACCGTCAAATACGCAGGTTGAACATCGTTTGCATGATCAATGGCAAAACGGTAAT

CCCCAGCGCTGGCACCTGACGCGAGACGATC

>151 bp product from linear template CP149200.1:c3701562-3700555 Salmonella enterica subsp. enterica serovar 4,[5],12:i:- strain Z1323HSL0094 chromosome, complete genome, base 802 to base 952 (Forward invj - Reverse invj).

CAGCGCTGGGGAAATGACTATTCCGTCAATATTCAGGCGCGGCAAGCAGGGGAGTTTTCG

TTAATACCGTCAAATACGCAGGTTGAACATCGTTTGCATGATCAATGGCAAAACGGTAAT

CCCCAGCGCTGGCACCTGACGCGAGACGATC

>151 bp product from linear template CP074291.1:1005353-1006360 Salmonella enterica subsp. enterica serovar Saintpaul strain CFSAN006195 chromosome, complete genome, base 802 to base 952 (Forward invj - Reverse invj).

CAGCGCTGGGGAAATGACTATTCCGTCAATATTCAGGCGCGGCAAGCAGGGGAGTTTTCG

TTAATACCGTCAAATACGCAGGTTGAACATCGTTTGCATGATCAATGGCAAAACGGTAAT

CCCCAGCGCTGGCACCTGACGCGAGACGATC

>151 bp product from linear template CP082374.1:1007883-1008890 Salmonella enterica subsp. enterica serovar Typhimurium strain FSIS1702508 chromosome, complete genome, base 802 to base 952 (Forward invj - Reverse invj).

CAGCGCTGGGGAAATGACTATTCCGTCAATATTCAGGCGCGGCAAGCAGGGGAGTTTTCG

TTAATACCGTCAAATACGCAGGTTGAACATCGTTTGCATGATCAATGGCAAAACGGTAAT

CCCCAGCGCTGGCACCTGACGCGAGACGATC

>151 bp product from linear template CP149412.1:c3626630-3625623 Salmonella enterica subsp. enterica serovar Typhimurium strain Z1322HSL0016 chromosome, complete genome, base 802 to base 952 (Forward invj - Reverse invj).

CAGCGCTGGGGAAATGACTATTCCGTCAATATTCAGGCGCGGCAAGCAGGGGAGTTTTCG

TTAATACCGTCAAATACGCAGGTTGAACATCGTTTGCATGATCAATGGCAAAACGGTAAT

CCCCAGCGCTGGCACCTGACGCGAGACGATC

>151 bp product from linear template CP149299.1:1008077-1009084 Salmonella enterica subsp. enterica serovar Typhimurium strain Z1323SSL0046 chromosome, complete genome, base 802 to base 952 (Forward invj - Reverse invj).

CAGCGCTGGGGAAATGACTATTCCGTCAATATTCAGGCGCGGCAAGCAGGGGAGTTTTCG

TTAATACCGTCAAATACGCAGGTTGAACATCGTTTGCATGATCAATGGCAAAACGGTAAT

CCCCAGCGCTGGCACCTGACGCGAGACGATC

>151 bp product from linear template CP039593.1:c3005845-3004838 Salmonella enterica subsp. enterica serovar 1,4,[5],12:i:- strain PNCS014863 chromosome, complete genome, base 802 to base 952 (Forward invj - Reverse invj).

CAGCGCTGGGGAAATGACTATTCCGTCAATATTCAGGCGCGGCAAGCAGGGGAGTTTTCG

TTAATACCGTCAAATACGCAGGTTGAACATCGTTTGCATGATCAATGGCAAAACGGTAAT

CCCCAGCGCTGGCACCTGACGCGAGACGATC

>151 bp product from linear template CP101375.1:1694041-1695048 Salmonella enterica strain SC2016090 chromosome, complete genome, base 802 to base 952 (Forward invj - Reverse invj).

CAGCGCTGGGGAAATGACTATTCCGTCAATATTCAGGCGCGGCAAGCAGGGGAGTTTTCG

TTAATACCGTCAAATACGCAGGTTGAACATCGTTTGCATGATCAATGGCAAAACGGTAAT

CCCCAGCGCTGGCACCTGACGCGAGACGATC

>151 bp product from linear template CP047115.1:1007965-1008972 Salmonella enterica subsp. enterica strain SCSM4.1 chromosome, complete genome, base 802 to base 952 (Forward invj - Reverse invj).

CAGCGCTGGGGAAATGACTATTCCGTCAATATTCAGGCGCGGCAAGCAGGGGAGTTTTCG

TTAATACCGTCAAATACGCAGGTTGAACATCGTTTGCATGATCAATGGCAAAACGGTAAT

CCCCAGCGCTGGCACCTGACGCGAGACGATC

>151 bp product from linear template CP007235.2:c3810336-3809329 Salmonella enterica subsp. enterica serovar Typhimurium str. USDA-ARS-USMARC-1899, complete genome, base 802 to base 952 (Forward invj - Reverse invj).

CAGCGCTGGGGAAATGACTATTCCGTCAATATTCAGGCGCGGCAAGCAGGGGAGTTTTCG

TTAATACCGTCAAATACGCAGGTTGAACATCGTTTGCATGATCAATGGCAAAACGGTAAT

CCCCAGCGCTGGCACCTGACGCGAGACGATC

>151 bp product from linear template CP149338.1:c640159-639152 Salmonella enterica subsp. enterica serovar Typhimurium strain Z1323SSL0003 chromosome, complete genome, base 802 to base 952 (Forward invj - Reverse invj).

CAGCGCTGGGGAAATGACTATTCCGTCAATATTCAGGCGCGGCAAGCAGGGGAGTTTTCG

TTAATACCGTCAAATACGCAGGTTGAACATCGTTTGCATGATCAATGGCAAAACGGTAAT

CCCCAGCGCTGGCACCTGACGCGAGACGATC

>151 bp product from linear template CP049986.1:1063206-1064213 Salmonella enterica subsp. enterica serovar Saintpaul strain CVM N16S270 chromosome, complete genome, base 802 to base 952 (Forward invj - Reverse invj).

CAGCGCTGGGGAAATGACTATTCCGTCAATATTCAGGCGCGGCAAGCAGGGGAGTTTTCG

TTAATACCGTCAAATACGCAGGTTGAACATCGTTTGCATGATCAATGGCAAAACGGTAAT

CCCCAGCGCTGGCACCTGACGCGAGACGATC

>151 bp product from linear template CP140755.1:1039937-1040944 Salmonella enterica subsp. enterica serovar 1,4,[5],12:i:- strain PNCS017412 chromosome, complete genome, base 802 to base 952 (Forward invj - Reverse invj).

CAGCGCTGGGGAAATGACTATTCCGTCAATATTCAGGCGCGGCAAGCAGGGGAGTTTTCG

TTAATACCGTCAAATACGCAGGTTGAACATCGTTTGCATGATCAATGGCAAAACGGTAAT

CCCCAGCGCTGGCACCTGACGCGAGACGATC

>151 bp product from linear template CP043907.1:c1015054-1014047 Salmonella enterica subsp. enterica serovar Typhimurium strain ATCC 14028 chromosome, complete genome, base 802 to base 952 (Forward invj - Reverse invj).

CAGCGCTGGGGAAATGACTATTCCGTCAATATTCAGGCGCGGCAAGCAGGGGAGTTTTCG

TTAATACCGTCAAATACGCAGGTTGAACATCGTTTGCATGATCAATGGCAAAACGGTAAT

CCCCAGCGCTGGCACCTGACGCGAGACGATC

>151 bp product from linear template CP019172.1:1005421-1006428 Salmonella enterica subsp. enterica serovar Saintpaul strain CFSAN004175, complete genome, base 802 to base 952 (Forward invj - Reverse invj).

CAGCGCTGGGGAAATGACTATTCCGTCAATATTCAGGCGCGGCAAGCAGGGGAGTTTTCG

TTAATACCGTCAAATACGCAGGTTGAACATCGTTTGCATGATCAATGGCAAAACGGTAAT

CCCCAGCGCTGGCACCTGACGCGAGACGATC

>151 bp product from linear template CP086118.1:1039919-1040926 Salmonella enterica subsp. enterica serovar Typhimurium strain S34 chromosome, complete genome, base 802 to base 952 (Forward invj - Reverse invj).

CAGCGCTGGGGAAATGACTATTCCGTCAATATTCAGGCGCGGCAAGCAGGGGAGTTTTCG

TTAATACCGTCAAATACGCAGGTTGAACATCGTTTGCATGATCAATGGCAAAACGGTAAT

CCCCAGCGCTGGCACCTGACGCGAGACGATC

>151 bp product from linear template CP070319.1:c2939977-2938970 Salmonella enterica subsp. enterica serovar Typhimurium strain Colony541 chromosome, base 802 to base 952 (Forward invj - Reverse invj).

CAGCGCTGGGGAAATGACTATTCCGTCAATATTCAGGCGCGGCAAGCAGGGGAGTTTTCG

TTAATACCGTCAAATACGCAGGTTGAACATCGTTTGCATGATCAATGGCAAAACGGTAAT

CCCCAGCGCTGGCACCTGACGCGAGACGATC

>151 bp product from linear template OU015337.1:1041096-1042103 Salmonella enterica subsp. enterica serovar Typhimurium strain AUSMDU00018340 genome assembly, chromosome: C1, base 802 to base 952 (Forward invj - Reverse invj).

CAGCGCTGGGGAAATGACTATTCCGTCAATATTCAGGCGCGGCAAGCAGGGGAGTTTTCG

TTAATACCGTCAAATACGCAGGTTGAACATCGTTTGCATGATCAATGGCAAAACGGTAAT

CCCCAGCGCTGGCACCTGACGCGAGACGATC

>151 bp product from linear template CP015157.1:c3032562-3031555 Salmonella enterica subsp. enterica serovar Typhimurium strain NC983 chromosome, complete genome, base 802 to base 952 (Forward invj - Reverse invj).

CAGCGCTGGGGAAATGACTATTCCGTCAATATTCAGGCGCGGCAAGCAGGGGAGTTTTCG

TTAATACCGTCAAATACGCAGGTTGAACATCGTTTGCATGATCAATGGCAAAACGGTAAT

CCCCAGCGCTGGCACCTGACGCGAGACGATC

>151 bp product from linear template CP082652.1:1007404-1008411 Salmonella enterica subsp. enterica serovar Typhimurium var. 5- strain CVM N16S132 chromosome, complete genome, base 802 to base 952 (Forward invj - Reverse invj).

CAGCGCTGGGGAAATGACTATTCCGTCAATATTCAGGCGCGGCAAGCAGGGGAGTTTTCG

TTAATACCGTCAAATACGCAGGTTGAACATCGTTTGCATGATCAATGGCAAAACGGTAAT

CCCCAGCGCTGGCACCTGACGCGAGACGATC

>151 bp product from linear template CP067339.1:1002226-1003233 Salmonella enterica subsp. enterica strain Se32 chromosome, complete genome, base 802 to base 952 (Forward invj - Reverse invj).

CAGCGCTGGGGAAATGACTATTCCGTCAATATTCAGGCGCGGCAAGCAGGGGAGTTTTCG

TTAATACCGTCAAATACGCAGGTTGAACATCGTTTGCATGATCAATGGCAAAACGGTAAT

CCCCAGCGCTGGCACCTGACGCGAGACGATC

>151 bp product from linear template CP040568.1:1007414-1008421 Salmonella enterica subsp. enterica serovar Typhimurium strain SAP17-8290 chromosome, complete genome, base 802 to base 952 (Forward invj - Reverse invj).

CAGCGCTGGGGAAATGACTATTCCGTCAATATTCAGGCGCGGCAAGCAGGGGAGTTTTCG

TTAATACCGTCAAATACGCAGGTTGAACATCGTTTGCATGATCAATGGCAAAACGGTAAT

CCCCAGCGCTGGCACCTGACGCGAGACGATC

>151 bp product from linear template AP019374.1:1120712-1121719 Salmonella enterica subsp. enterica serovar 4,[5],12:i:- L-3838 DNA, complete genome, base 802 to base 952 (Forward invj - Reverse invj).

CAGCGCTGGGGAAATGACTATTCCGTCAATATTCAGGCGCGGCAAGCAGGGGAGTTTTCG

TTAATACCGTCAAATACGCAGGTTGAACATCGTTTGCATGATCAATGGCAAAACGGTAAT

CCCCAGCGCTGGCACCTGACGCGAGACGATC

>151 bp product from linear template CP039558.1:c2970571-2969564 Salmonella enterica subsp. enterica serovar 1,4,[5],12:i:- strain PNCS014846 chromosome, complete genome, base 802 to base 952 (Forward invj - Reverse invj).

CAGCGCTGGGGAAATGACTATTCCGTCAATATTCAGGCGCGGCAAGCAGGGGAGTTTTCG

TTAATACCGTCAAATACGCAGGTTGAACATCGTTTGCATGATCAATGGCAAAACGGTAAT

CCCCAGCGCTGGCACCTGACGCGAGACGATC

>151 bp product from linear template CP029839.1:c3045045-3044038 Salmonella enterica subsp. enterica serovar Typhimurium strain 10ST07093 chromosome, complete genome, base 802 to base 952 (Forward invj - Reverse invj).

CAGCGCTGGGGAAATGACTATTCCGTCAATATTCAGGCGCGGCAAGCAGGGGAGTTTTCG

TTAATACCGTCAAATACGCAGGTTGAACATCGTTTGCATGATCAATGGCAAAACGGTAAT

CCCCAGCGCTGGCACCTGACGCGAGACGATC

>151 bp product from linear template CP050130.1:c3704815-3703808 Salmonella enterica subsp. enterica serovar Typhimurium strain GSJ/2017-Sal-008 chromosome, complete genome, base 802 to base 952 (Forward invj - Reverse invj).

CAGCGCTGGGGAAATGACTATTCCGTCAATATTCAGGCGCGGCAAGCAGGGGAGTTTTCG

TTAATACCGTCAAATACGCAGGTTGAACATCGTTTGCATGATCAATGGCAAAACGGTAAT

CCCCAGCGCTGGCACCTGACGCGAGACGATC

>151 bp product from linear template CP017621.1:c2923530-2922523 Salmonella enterica subsp. enterica serovar Typhimurium strain 22792, complete genome, base 802 to base 952 (Forward invj - Reverse invj).

CAGCGCTGGGGAAATGACTATTCCGTCAATATTCAGGCGCGGCAAGCAGGGGAGTTTTCG

TTAATACCGTCAAATACGCAGGTTGAACATCGTTTGCATGATCAATGGCAAAACGGTAAT

CCCCAGCGCTGGCACCTGACGCGAGACGATC

>151 bp product from linear template CP060165.1:c3025902-3024895 Salmonella enterica subsp. enterica serovar Typhimurium strain D37712 chromosome, complete genome, base 802 to base 952 (Forward invj - Reverse invj).

CAGCGCTGGGGAAATGACTATTCCGTCAATATTCAGGCGCGGCAAGCAGGGGAGTTTTCG

TTAATACCGTCAAATACGCAGGTTGAACATCGTTTGCATGATCAATGGCAAAACGGTAAT

CCCCAGCGCTGGCACCTGACGCGAGACGATC

>151 bp product from linear template CP129206.1:1006323-1007330 Salmonella enterica subsp. enterica serovar Typhimurium strain HS_187Salm chromosome, complete genome, base 802 to base 952 (Forward invj - Reverse invj).

CAGCGCTGGGGAAATGACTATTCCGTCAATATTCAGGCGCGGCAAGCAGGGGAGTTTTCG

TTAATACCGTCAAATACGCAGGTTGAACATCGTTTGCATGATCAATGGCAAAACGGTAAT

CCCCAGCGCTGGCACCTGACGCGAGACGATC

>151 bp product from linear template CP065062.1:3664938-3665945 Salmonella enterica subsp. enterica strain RG21060838 chromosome, complete genome, base 802 to base 952 (Forward invj - Reverse invj).

CAGCGCTGGGGAAATGACTATTCCGTCAATATTCAGGCGCGGCAAGCAGGGGAGTTTTCG

TTAATACCGTCAAATACGCAGGTTGAACATCGTTTGCATGATCAATGGCAAAACGGTAAT

CCCCAGCGCTGGCACCTGACGCGAGACGATC

>151 bp product from linear template CP016385.1:c3038508-3037501 Salmonella enterica subsp. enterica serovar Typhimurium strain 13-931, complete genome, base 802 to base 952 (Forward invj - Reverse invj).

CAGCGCTGGGGAAATGACTATTCCGTCAATATTCAGGCGCGGCAAGCAGGGGAGTTTTCG

TTAATACCGTCAAATACGCAGGTTGAACATCGTTTGCATGATCAATGGCAAAACGGTAAT

CCCCAGCGCTGGCACCTGACGCGAGACGATC

>151 bp product from linear template U10872.1:514-1521 Salmonella enterica typhimurium SR11 InvI and InvJ genes, complete cds, and InvC gene, partial cds, base 802 to base 952 (Forward invj - Reverse invj).

CAGCGCTGGGGAAATGACTATTCCGTCAATATTCAGGCGCGGCAAGCAGGGGAGTTTTCG

TTAATACCGTCAAATACGCAGGTTGAACATCGTTTGCATGATCAATGGCAAAACGGTAAT

CCCCAGCGCTGGCACCTGACGCGAGACGATC

>151 bp product from linear template CP082571.1:1041673-1042680 Salmonella enterica subsp. enterica serovar 4,[5],12:i:- strain CVM N18S0993 chromosome, complete genome, base 802 to base 952 (Forward invj - Reverse invj).

CAGCGCTGGGGAAATGACTATTCCGTCAATATTCAGGCGCGGCAAGCAGGGGAGTTTTCG

TTAATACCGTCAAATACGCAGGTTGAACATCGTTTGCATGATCAATGGCAAAACGGTAAT

CCCCAGCGCTGGCACCTGACGCGAGACGATC

>151 bp product from linear template CP114540.1:3579210-3580217 Salmonella enterica strain 2011K-0052 chromosome, base 802 to base 952 (Forward invj - Reverse invj).

CAGCGCTGGGGAAATGACTATTCCGTCAATATTCAGGCGCGGCAAGCAGGGGAGTTTTCG

TTAATACCGTCAAATACGCAGGTTGAACATCGTTTGCATGATCAATGGCAAAACGGTAAT

CCCCAGCGCTGGCACCTGACGCGAGACGATC

>151 bp product from linear template CP082526.1:c3944749-3943742 Salmonella enterica subsp. enterica serovar Typhimurium strain CVM N18S2170 chromosome, complete genome, base 802 to base 952 (Forward invj - Reverse invj).

CAGCGCTGGGGAAATGACTATTCCGTCAATATTCAGGCGCGGCAAGCAGGGGAGTTTTCG

TTAATACCGTCAAATACGCAGGTTGAACATCGTTTGCATGATCAATGGCAAAACGGTAAT

CCCCAGCGCTGGCACCTGACGCGAGACGATC

>151 bp product from linear template CP117244.1:1002220-1003227 Salmonella enterica subsp. enterica serovar Typhimurium strain ATCC 14028 chromosome, complete genome, base 802 to base 952 (Forward invj - Reverse invj).

CAGCGCTGGGGAAATGACTATTCCGTCAATATTCAGGCGCGGCAAGCAGGGGAGTTTTCG

TTAATACCGTCAAATACGCAGGTTGAACATCGTTTGCATGATCAATGGCAAAACGGTAAT

CCCCAGCGCTGGCACCTGACGCGAGACGATC

>151 bp product from linear template CP014536.1:c3062682-3061675 Salmonella enterica subsp. enterica serovar Typhimurium strain SO3, complete genome, base 802 to base 952 (Forward invj - Reverse invj).

CAGCGCTGGGGAAATGACTATTCCGTCAATATTCAGGCGCGGCAAGCAGGGGAGTTTTCG

TTAATACCGTCAAATACGCAGGTTGAACATCGTTTGCATGATCAATGGCAAAACGGTAAT

CCCCAGCGCTGGCACCTGACGCGAGACGATC

>151 bp product from linear template CP091542.1:1007828-1008835 Salmonella enterica subsp. enterica serovar Typhimurium strain S58L2 chromosome, complete genome, base 802 to base 952 (Forward invj - Reverse invj).

CAGCGCTGGGGAAATGACTATTCCGTCAATATTCAGGCGCGGCAAGCAGGGGAGTTTTCG

TTAATACCGTCAAATACGCAGGTTGAACATCGTTTGCATGATCAATGGCAAAACGGTAAT

CCCCAGCGCTGGCACCTGACGCGAGACGATC

>151 bp product from linear template CP040668.1:c3042084-3041077 Salmonella enterica subsp. enterica serovar 1,4,[5],12:i:- strain SA20082869 chromosome, complete genome, base 802 to base 952 (Forward invj - Reverse invj).

CAGCGCTGGGGAAATGACTATTCCGTCAATATTCAGGCGCGGCAAGCAGGGGAGTTTTCG

TTAATACCGTCAAATACGCAGGTTGAACATCGTTTGCATGATCAATGGCAAAACGGTAAT

CCCCAGCGCTGGCACCTGACGCGAGACGATC

>151 bp product from linear template CP065718.1:c4864529-4863522 Salmonella enterica strain FDAARGOS_878 chromosome, complete genome, base 802 to base 952 (Forward invj - Reverse invj).

CAGCGCTGGGGAAATGACTATTCCGTCAATATTCAGGCGCGGCAAGCAGGGGAGTTTTCG

TTAATACCGTCAAATACGCAGGTTGAACATCGTTTGCATGATCAATGGCAAAACGGTAAT

CCCCAGCGCTGGCACCTGACGCGAGACGATC

>151 bp product from linear template CP040700.1:1005485-1006492 Salmonella enterica subsp. enterica serovar Saintpaul strain 5 isolate CFSAN047351 chromosome, complete genome, base 802 to base 952 (Forward invj - Reverse invj).

CAGCGCTGGGGAAATGACTATTCCGTCAATATTCAGGCGCGGCAAGCAGGGGAGTTTTCG

TTAATACCGTCAAATACGCAGGTTGAACATCGTTTGCATGATCAATGGCAAAACGGTAAT

CCCCAGCGCTGGCACCTGACGCGAGACGATC

>151 bp product from linear template CP149360.1:c3615073-3614066 Salmonella enterica subsp. enterica serovar Typhimurium strain Z1323HSL0061 chromosome, complete genome, base 802 to base 952 (Forward invj - Reverse invj).

CAGCGCTGGGGAAATGACTATTCCGTCAATATTCAGGCGCGGCAAGCAGGGGAGTTTTCG

TTAATACCGTCAAATACGCAGGTTGAACATCGTTTGCATGATCAATGGCAAAACGGTAAT

CCCCAGCGCTGGCACCTGACGCGAGACGATC

>151 bp product from linear template OU015323.1:4293741-4294748 Salmonella enterica subsp. enterica serovar Typhimurium strain AUSMDU00004549 genome assembly, chromosome: C1, base 802 to base 952 (Forward invj - Reverse invj).

CAGCGCTGGGGAAATGACTATTCCGTCAATATTCAGGCGCGGCAAGCAGGGGAGTTTTCG

TTAATACCGTCAAATACGCAGGTTGAACATCGTTTGCATGATCAATGGCAAAACGGTAAT

CCCCAGCGCTGGCACCTGACGCGAGACGATC

>151 bp product from linear template CP061045.1:1001519-1002526 Salmonella enterica subsp. enterica serovar Typhimurium strain BBS1406 chromosome, complete genome, base 802 to base 952 (Forward invj - Reverse invj).

CAGCGCTGGGGAAATGACTATTCCGTCAATATTCAGGCGCGGCAAGCAGGGGAGTTTTCG

TTAATACCGTCAAATACGCAGGTTGAACATCGTTTGCATGATCAATGGCAAAACGGTAAT

CCCCAGCGCTGGCACCTGACGCGAGACGATC

>151 bp product from linear template CP074307.1:1004949-1005956 Salmonella enterica subsp. enterica strain CFSAN004112 chromosome, complete genome, base 802 to base 952 (Forward invj - Reverse invj).

CAGCGCTGGGGAAATGACTATTCCGTCAATATTCAGGCGCGGCAAGCAGGGGAGTTTTCG

TTAATACCGTCAAATACGCAGGTTGAACATCGTTTGCATGATCAATGGCAAAACGGTAAT

CCCCAGCGCTGGCACCTGACGCGAGACGATC

>151 bp product from linear template CP119510.1:c3956032-3955025 Salmonella enterica subsp. enterica serovar 4,[5],12:i:- strain FSIS31901558 chromosome, complete genome, base 802 to base 952 (Forward invj - Reverse invj).

CAGCGCTGGGGAAATGACTATTCCGTCAATATTCAGGCGCGGCAAGCAGGGGAGTTTTCG

TTAATACCGTCAAATACGCAGGTTGAACATCGTTTGCATGATCAATGGCAAAACGGTAAT

CCCCAGCGCTGGCACCTGACGCGAGACGATC

>151 bp product from linear template CP149239.1:c3666689-3665682 Salmonella enterica subsp. enterica serovar 4,[5],12:i:- strain Z1323HSL0001 chromosome, complete genome, base 802 to base 952 (Forward invj - Reverse invj).

CAGCGCTGGGGAAATGACTATTCCGTCAATATTCAGGCGCGGCAAGCAGGGGAGTTTTCG

TTAATACCGTCAAATACGCAGGTTGAACATCGTTTGCATGATCAATGGCAAAACGGTAAT

CCCCAGCGCTGGCACCTGACGCGAGACGATC

>151 bp product from linear template CP061124.1:1088644-1089651 Salmonella enterica subsp. enterica serovar Typhimurium strain S438 chromosome, complete genome, base 802 to base 952 (Forward invj - Reverse invj).

CAGCGCTGGGGAAATGACTATTCCGTCAATATTCAGGCGCGGCAAGCAGGGGAGTTTTCG

TTAATACCGTCAAATACGCAGGTTGAACATCGTTTGCATGATCAATGGCAAAACGGTAAT

CCCCAGCGCTGGCACCTGACGCGAGACGATC

>151 bp product from linear template CP047529.1:c3938336-3937329 Salmonella enterica subsp. enterica serovar Typhimurium strain SJTUF10640 chromosome, complete genome, base 802 to base 952 (Forward invj - Reverse invj).

CAGCGCTGGGGAAATGACTATTCCGTCAATATTCAGGCGCGGCAAGCAGGGGAGTTTTCG

TTAATACCGTCAAATACGCAGGTTGAACATCGTTTGCATGATCAATGGCAAAACGGTAAT

CCCCAGCGCTGGCACCTGACGCGAGACGATC

>151 bp product from linear template CP066009.1:c468733-467726 Salmonella enterica strain FDAARGOS_1067 chromosome, complete genome, base 802 to base 952 (Forward invj - Reverse invj).

CAGCGCTGGGGAAATGACTATTCCGTCAATATTCAGGCGCGGCAAGCAGGGGAGTTTTCG

TTAATACCGTCAAATACGCAGGTTGAACATCGTTTGCATGATCAATGGCAAAACGGTAAT

CCCCAGCGCTGGCACCTGACGCGAGACGATC

>151 bp product from linear template CP148776.1:c801853-800846 Salmonella enterica subsp. enterica serovar Infantis strain Z1323HSL0097 chromosome, complete genome, base 802 to base 952 (Forward invj - Reverse invj).

CAGCGCTGGGGAAATGACTATTCCGTCAATATTCAGGCGCGGCAAGCAGGGGAGTTTTCG

TTAATACCGTCAAATACGCAGGTTGAACATCGTTTGCATGATCAATGGCAAAACGGTAAT

CCCCAGCGCTGGCACCTGACGCGAGACGATC

>151 bp product from linear template CP038432.1:c1730192-1729185 Salmonella enterica subsp. enterica serovar Typhimurium strain E40 chromosome, complete genome, base 802 to base 952 (Forward invj - Reverse invj).

CAGCGCTGGGGAAATGACTATTCCGTCAATATTCAGGCGCGGCAAGCAGGGGAGTTTTCG

TTAATACCGTCAAATACGCAGGTTGAACATCGTTTGCATGATCAATGGCAAAACGGTAAT

CCCCAGCGCTGGCACCTGACGCGAGACGATC

>151 bp product from linear template CP041976.1:1168140-1169147 Salmonella enterica subsp. enterica serovar Typhimurium strain NCCP 16207 chromosome, complete genome, base 802 to base 952 (Forward invj - Reverse invj).

CAGCGCTGGGGAAATGACTATTCCGTCAATATTCAGGCGCGGCAAGCAGGGGAGTTTTCG

TTAATACCGTCAAATACGCAGGTTGAACATCGTTTGCATGATCAATGGCAAAACGGTAAT

CCCCAGCGCTGGCACCTGACGCGAGACGATC

>151 bp product from linear template CP033352.2:c4238789-4237782 Salmonella enterica subsp. enterica strain CFSA629 chromosome, complete genome, base 802 to base 952 (Forward invj - Reverse invj).

CAGCGCTGGGGAAATGACTATTCCGTCAATATTCAGGCGCGGCAAGCAGGGGAGTTTTCG

TTAATACCGTCAAATACGCAGGTTGAACATCGTTTGCATGATCAATGGCAAAACGGTAAT

CCCCAGCGCTGGCACCTGACGCGAGACGATC

>151 bp product from linear template CP074310.1:c3839313-3838306 Salmonella enterica subsp. enterica strain CFSAN004085 chromosome, complete genome, base 802 to base 952 (Forward invj - Reverse invj).

CAGCGCTGGGGAAATGACTATTCCGTCAATATTCAGGCGCGGCAAGCAGGGGAGTTTTCG

TTAATACCGTCAAATACGCAGGTTGAACATCGTTTGCATGATCAATGGCAAAACGGTAAT

CCCCAGCGCTGGCACCTGACGCGAGACGATC

>151 bp product from linear template CP061119.1:4248333-4249340 Salmonella enterica subsp. enterica serovar Typhimurium strain S520 chromosome, complete genome, base 802 to base 952 (Forward invj - Reverse invj).

CAGCGCTGGGGAAATGACTATTCCGTCAATATTCAGGCGCGGCAAGCAGGGGAGTTTTCG

TTAATACCGTCAAATACGCAGGTTGAACATCGTTTGCATGATCAATGGCAAAACGGTAAT

CCCCAGCGCTGGCACCTGACGCGAGACGATC

>151 bp product from linear template CP032494.1:c2944227-2943220 Salmonella enterica subsp. enterica serovar Typhimurium strain SO21 chromosome, complete genome, base 802 to base 952 (Forward invj - Reverse invj).

CAGCGCTGGGGAAATGACTATTCCGTCAATATTCAGGCGCGGCAAGCAGGGGAGTTTTCG

TTAATACCGTCAAATACGCAGGTTGAACATCGTTTGCATGATCAATGGCAAAACGGTAAT

CCCCAGCGCTGGCACCTGACGCGAGACGATC

>151 bp product from linear template CP113538.1:1036104-1037111 Salmonella enterica strain XSK chromosome, complete genome, base 802 to base 952 (Forward invj - Reverse invj).

CAGCGCTGGGGAAATGACTATTCCGTCAATATTCAGGCGCGGCAAGCAGGGGAGTTTTCG

TTAATACCGTCAAATACGCAGGTTGAACATCGTTTGCATGATCAATGGCAAAACGGTAAT

CCCCAGCGCTGGCACCTGACGCGAGACGATC

>151 bp product from linear template CP149243.1:3089897-3090904 Salmonella enterica subsp. enterica serovar 4,[5],12:i:- strain Z1322HSL0066 chromosome, complete genome, base 802 to base 952 (Forward invj - Reverse invj).

CAGCGCTGGGGAAATGACTATTCCGTCAATATTCAGGCGCGGCAAGCAGGGGAGTTTTCG

TTAATACCGTCAAATACGCAGGTTGAACATCGTTTGCATGATCAATGGCAAAACGGTAAT

CCCCAGCGCTGGCACCTGACGCGAGACGATC

>151 bp product from linear template CP092491.1:2904786-2905793 Salmonella enterica subsp. enterica serovar 4,[5],12:i:- strain H1 - 120 chromosome, complete genome, base 802 to base 952 (Forward invj - Reverse invj).

CAGCGCTGGGGAAATGACTATTCCGTCAATATTCAGGCGCGGCAAGCAGGGGAGTTTTCG

TTAATACCGTCAAATACGCAGGTTGAACATCGTTTGCATGATCAATGGCAAAACGGTAAT

CCCCAGCGCTGGCACCTGACGCGAGACGATC

>151 bp product from linear template CP014977.1:1049241-1050248 Salmonella enterica subsp. enterica serovar Typhimurium str. USDA-ARS-USMARC-1896 chromosome, complete genome, base 802 to base 952 (Forward invj - Reverse invj).

CAGCGCTGGGGAAATGACTATTCCGTCAATATTCAGGCGCGGCAAGCAGGGGAGTTTTCG

TTAATACCGTCAAATACGCAGGTTGAACATCGTTTGCATGATCAATGGCAAAACGGTAAT

CCCCAGCGCTGGCACCTGACGCGAGACGATC

>151 bp product from linear template CP101386.1:1120498-1121505 Salmonella enterica strain SC2017100 chromosome, complete genome, base 802 to base 952 (Forward invj - Reverse invj).

CAGCGCTGGGGAAATGACTATTCCGTCAATATTCAGGCGCGGCAAGCAGGGGAGTTTTCG

TTAATACCGTCAAATACGCAGGTTGAACATCGTTTGCATGATCAATGGCAAAACGGTAAT

CCCCAGCGCTGGCACCTGACGCGAGACGATC

>151 bp product from linear template CP082468.1:1010451-1011458 Salmonella enterica subsp. enterica serovar Typhimurium strain FSIS1607455 chromosome, complete genome, base 802 to base 952 (Forward invj - Reverse invj).

CAGCGCTGGGGAAATGACTATTCCGTCAATATTCAGGCGCGGCAAGCAGGGGAGTTTTCG

TTAATACCGTCAAATACGCAGGTTGAACATCGTTTGCATGATCAATGGCAAAACGGTAAT

CCCCAGCGCTGGCACCTGACGCGAGACGATC

>151 bp product from linear template CP050753.1:1039566-1040573 Salmonella enterica subsp. enterica serovar Typhimurium strain ST45 chromosome, complete genome, base 802 to base 952 (Forward invj - Reverse invj).

CAGCGCTGGGGAAATGACTATTCCGTCAATATTCAGGCGCGGCAAGCAGGGGAGTTTTCG

TTAATACCGTCAAATACGCAGGTTGAACATCGTTTGCATGATCAATGGCAAAACGGTAAT

CCCCAGCGCTGGCACCTGACGCGAGACGATC

>151 bp product from linear template CP160178.1:1006651-1007658 Salmonella enterica subsp. enterica strain N23-2949 chromosome, complete genome, base 802 to base 952 (Forward invj - Reverse invj).

CAGCGCTGGGGAAATGACTATTCCGTCAATATTCAGGCGCGGCAAGCAGGGGAGTTTTCG

TTAATACCGTCAAATACGCAGGTTGAACATCGTTTGCATGATCAATGGCAAAACGGTAAT

CCCCAGCGCTGGCACCTGACGCGAGACGATC

>151 bp product from linear template CP017728.1:1007717-1008724 Salmonella enterica subsp. enterica serovar Typhimurium str. SARA13, complete sequence., base 802 to base 952 (Forward invj - Reverse invj).

CAGCGCTGGGGAAATGACTATTCCGTCAATATTCAGGCGCGGCAAGCAGGGGAGTTTTCG

TTAATACCGTCAAATACGCAGGTTGAACATCGTTTGCATGATCAATGGCAAAACGGTAAT

CCCCAGCGCTGGCACCTGACGCGAGACGATC

>151 bp product from linear template CP039579.1:c3042033-3041026 Salmonella enterica subsp. enterica serovar 1,4,[5],12:i:- strain PNCS014857 chromosome, complete genome, base 802 to base 952 (Forward invj - Reverse invj).

CAGCGCTGGGGAAATGACTATTCCGTCAATATTCAGGCGCGGCAAGCAGGGGAGTTTTCG

TTAATACCGTCAAATACGCAGGTTGAACATCGTTTGCATGATCAATGGCAAAACGGTAAT

CCCCAGCGCTGGCACCTGACGCGAGACGATC

>151 bp product from linear template CP050726.1:1040948-1041955 Salmonella enterica subsp. enterica serovar Typhimurium strain ST113 chromosome, complete genome, base 802 to base 952 (Forward invj - Reverse invj).

CAGCGCTGGGGAAATGACTATTCCGTCAATATTCAGGCGCGGCAAGCAGGGGAGTTTTCG

TTAATACCGTCAAATACGCAGGTTGAACATCGTTTGCATGATCAATGGCAAAACGGTAAT

CCCCAGCGCTGGCACCTGACGCGAGACGATC

>151 bp product from linear template CP082609.1:1039931-1040938 Salmonella enterica subsp. enterica serovar 4,12:i:- strain CVM N17S056 isolate 17IA01GT15-S H2 chromosome, complete genome, base 802 to base 952 (Forward invj - Reverse invj).

CAGCGCTGGGGAAATGACTATTCCGTCAATATTCAGGCGCGGCAAGCAGGGGAGTTTTCG

TTAATACCGTCAAATACGCAGGTTGAACATCGTTTGCATGATCAATGGCAAAACGGTAAT

CCCCAGCGCTGGCACCTGACGCGAGACGATC

>151 bp product from linear template CP074213.1:1029738-1030745 Salmonella enterica subsp. enterica serovar Saintpaul str. SARA26 strain SGSC 2206 chromosome, complete genome, base 802 to base 952 (Forward invj - Reverse invj).

CAGCGCTGGGGAAATGACTATTCCGTCAATATTCAGGCGCGGCAAGCAGGGGAGTTTTCG

TTAATACCGTCAAATACGCAGGTTGAACATCGTTTGCATGATCAATGGCAAAACGGTAAT

CCCCAGCGCTGGCACCTGACGCGAGACGATC

>151 bp product from linear template CP149207.1:c3610938-3609931 Salmonella enterica subsp. enterica serovar 4,[5],12:i:- strain Z1323HSL0072 chromosome, complete genome, base 802 to base 952 (Forward invj - Reverse invj).

CAGCGCTGGGGAAATGACTATTCCGTCAATATTCAGGCGCGGCAAGCAGGGGAGTTTTCG

TTAATACCGTCAAATACGCAGGTTGAACATCGTTTGCATGATCAATGGCAAAACGGTAAT

CCCCAGCGCTGGCACCTGACGCGAGACGATC

>151 bp product from linear template CP047544.1:1039897-1040904 Salmonella enterica subsp. enterica serovar Typhimurium strain SJTUF10250 chromosome, complete genome, base 802 to base 952 (Forward invj - Reverse invj).

CAGCGCTGGGGAAATGACTATTCCGTCAATATTCAGGCGCGGCAAGCAGGGGAGTTTTCG

TTAATACCGTCAAATACGCAGGTTGAACATCGTTTGCATGATCAATGGCAAAACGGTAAT

CCCCAGCGCTGGCACCTGACGCGAGACGATC

>151 bp product from linear template CP014967.1:1091298-1092305 Salmonella enterica subsp. enterica serovar Typhimurium str. CDC 2011K-1702 chromosome, complete genome, base 802 to base 952 (Forward invj - Reverse invj).

CAGCGCTGGGGAAATGACTATTCCGTCAATATTCAGGCGCGGCAAGCAGGGGAGTTTTCG

TTAATACCGTCAAATACGCAGGTTGAACATCGTTTGCATGATCAATGGCAAAACGGTAAT

CCCCAGCGCTGGCACCTGACGCGAGACGATC

>151 bp product from linear template CP085987.1:c4176276-4175269 Salmonella enterica strain SZL 38 chromosome, complete genome, base 802 to base 952 (Forward invj - Reverse invj).

CAGCGCTGGGGAAATGACTATTCCGTCAATATTCAGGCGCGGCAAGCAGGGGAGTTTTCG

TTAATACCGTCAAATACGCAGGTTGAACATCGTTTGCATGATCAATGGCAAAACGGTAAT

CCCCAGCGCTGGCACCTGACGCGAGACGATC

>151 bp product from linear template LN999997.1:2366933-2367940 Salmonella enterica subsp. enterica serovar Typhimurium isolate SO4698-09 genome assembly, chromosome: I, base 802 to base 952 (Forward invj - Reverse invj).

CAGCGCTGGGGAAATGACTATTCCGTCAATATTCAGGCGCGGCAAGCAGGGGAGTTTTCG

TTAATACCGTCAAATACGCAGGTTGAACATCGTTTGCATGATCAATGGCAAAACGGTAAT

CCCCAGCGCTGGCACCTGACGCGAGACGATC

>151 bp product from linear template CP050731.1:1040608-1041615 Salmonella enterica subsp. enterica serovar Typhimurium strain ST101 chromosome, complete genome, base 802 to base 952 (Forward invj - Reverse invj).

CAGCGCTGGGGAAATGACTATTCCGTCAATATTCAGGCGCGGCAAGCAGGGGAGTTTTCG

TTAATACCGTCAAATACGCAGGTTGAACATCGTTTGCATGATCAATGGCAAAACGGTAAT

CCCCAGCGCTGGCACCTGACGCGAGACGATC

>151 bp product from linear template CP058807.1:1007747-1008754 Salmonella enterica strain SRC27 chromosome, complete genome, base 802 to base 952 (Forward invj - Reverse invj).

CAGCGCTGGGGAAATGACTATTCCGTCAATATTCAGGCGCGGCAAGCAGGGGAGTTTTCG

TTAATACCGTCAAATACGCAGGTTGAACATCGTTTGCATGATCAATGGCAAAACGGTAAT

CCCCAGCGCTGGCACCTGACGCGAGACGATC

>151 bp product from linear template CP036168.1:1040729-1041736 Salmonella enterica subsp. enterica serovar Typhimurium strain sg_wt7 chromosome, complete genome, base 802 to base 952 (Forward invj - Reverse invj).

CAGCGCTGGGGAAATGACTATTCCGTCAATATTCAGGCGCGGCAAGCAGGGGAGTTTTCG

TTAATACCGTCAAATACGCAGGTTGAACATCGTTTGCATGATCAATGGCAAAACGGTAAT

CCCCAGCGCTGGCACCTGACGCGAGACGATC

>151 bp product from linear template CP048926.1:1000728-1001735 Salmonella enterica subsp. enterica serovar Saintpaul strain NY-N14748 chromosome, complete genome, base 802 to base 952 (Forward invj - Reverse invj).

CAGCGCTGGGGAAATGACTATTCCGTCAATATTCAGGCGCGGCAAGCAGGGGAGTTTTCG

TTAATACCGTCAAATACGCAGGTTGAACATCGTTTGCATGATCAATGGCAAAACGGTAAT

CCCCAGCGCTGGCACCTGACGCGAGACGATC

>151 bp product from linear template LR792437.1:480562-481569 Salmonella enterica subsp. enterica serovar Typhimurium isolate SV68791 genome assembly, chromosome: 1, base 802 to base 952 (Forward invj - Reverse invj).

CAGCGCTGGGGAAATGACTATTCCGTCAATATTCAGGCGCGGCAAGCAGGGGAGTTTTCG

TTAATACCGTCAAATACGCAGGTTGAACATCGTTTGCATGATCAATGGCAAAACGGTAAT

CCCCAGCGCTGGCACCTGACGCGAGACGATC

>151 bp product from linear template CP014979.2:1089023-1090030 Salmonella enterica subsp. enterica serovar Typhimurium str. CDC H2662, complete genome, base 802 to base 952 (Forward invj - Reverse invj).

CAGCGCTGGGGAAATGACTATTCCGTCAATATTCAGGCGCGGCAAGCAGGGGAGTTTTCG

TTAATACCGTCAAATACGCAGGTTGAACATCGTTTGCATGATCAATGGCAAAACGGTAAT

CCCCAGCGCTGGCACCTGACGCGAGACGATC

>151 bp product from linear template CP021909.1:c3847795-3846788 Salmonella enterica subsp. enterica strain ST1120, complete genome, base 802 to base 952 (Forward invj - Reverse invj).

CAGCGCTGGGGAAATGACTATTCCGTCAATATTCAGGCGCGGCAAGCAGGGGAGTTTTCG

TTAATACCGTCAAATACGCAGGTTGAACATCGTTTGCATGATCAATGGCAAAACGGTAAT

CCCCAGCGCTGGCACCTGACGCGAGACGATC

>151 bp product from linear template CP055130.1:c4849034-4848027 Salmonella enterica strain FDAARGOS_711 chromosome, base 802 to base 952 (Forward invj - Reverse invj).

CAGCGCTGGGGAAATGACTATTCCGTCAATATTCAGGCGCGGCAAGCAGGGGAGTTTTCG

TTAATACCGTCAAATACGCAGGTTGAACATCGTTTGCATGATCAATGGCAAAACGGTAAT

CCCCAGCGCTGGCACCTGACGCGAGACGATC

>151 bp product from linear template CP149221.1:c2365475-2364468 Salmonella enterica subsp. enterica serovar 4,[5],12:i:- strain Z1323HSL0018 chromosome, complete genome, base 802 to base 952 (Forward invj - Reverse invj).

CAGCGCTGGGGAAATGACTATTCCGTCAATATTCAGGCGCGGCAAGCAGGGGAGTTTTCG

TTAATACCGTCAAATACGCAGGTTGAACATCGTTTGCATGATCAATGGCAAAACGGTAAT

CCCCAGCGCTGGCACCTGACGCGAGACGATC

>151 bp product from linear template CP034719.1:1008707-1009714 Salmonella enterica subsp. enterica serovar Typhimurium strain RSE04 chromosome, complete genome, base 802 to base 952 (Forward invj - Reverse invj).

CAGCGCTGGGGAAATGACTATTCCGTCAATATTCAGGCGCGGCAAGCAGGGGAGTTTTCG

TTAATACCGTCAAATACGCAGGTTGAACATCGTTTGCATGATCAATGGCAAAACGGTAAT

CCCCAGCGCTGGCACCTGACGCGAGACGATC

>151 bp product from linear template CP090304.1:c3036263-3035256 Salmonella enterica subsp. enterica serovar Typhimurium strain ZJUJY chromosome, complete genome, base 802 to base 952 (Forward invj - Reverse invj).

CAGCGCTGGGGAAATGACTATTCCGTCAATATTCAGGCGCGGCAAGCAGGGGAGTTTTCG

TTAATACCGTCAAATACGCAGGTTGAACATCGTTTGCATGATCAATGGCAAAACGGTAAT

CCCCAGCGCTGGCACCTGACGCGAGACGATC

>151 bp product from linear template CP149350.1:1431582-1432589 Salmonella enterica subsp. enterica serovar Typhimurium strain Z1323HSL0082 chromosome, complete genome, base 802 to base 952 (Forward invj - Reverse invj).

CAGCGCTGGGGAAATGACTATTCCGTCAATATTCAGGCGCGGCAAGCAGGGGAGTTTTCG

TTAATACCGTCAAATACGCAGGTTGAACATCGTTTGCATGATCAATGGCAAAACGGTAAT

CCCCAGCGCTGGCACCTGACGCGAGACGATC

>151 bp product from linear template CP091571.1:1039870-1040877 Salmonella enterica strain 2 chromosome, complete genome, base 802 to base 952 (Forward invj - Reverse invj).

CAGCGCTGGGGAAATGACTATTCCGTCAATATTCAGGCGCGGCAAGCAGGGGAGTTTTCG

TTAATACCGTCAAATACGCAGGTTGAACATCGTTTGCATGATCAATGGCAAAACGGTAAT

CCCCAGCGCTGGCACCTGACGCGAGACGATC

>151 bp product from linear template OY754829.1:1345110-1346117 Salmonella enterica subsp. enterica serovar Typhimurium isolate Reference genome assembly, chromosome: GPT21-001_1, base 802 to base 952 (Forward invj - Reverse invj).

CAGCGCTGGGGAAATGACTATTCCGTCAATATTCAGGCGCGGCAAGCAGGGGAGTTTTCG

TTAATACCGTCAAATACGCAGGTTGAACATCGTTTGCATGATCAATGGCAAAACGGTAAT

CCCCAGCGCTGGCACCTGACGCGAGACGATC

>151 bp product from linear template AP023306.1:1008055-1009062 Salmonella enterica subsp. enterica serovar 4,[5],12:i:- L-4567 DNA, complete genome, base 802 to base 952 (Forward invj - Reverse invj).

CAGCGCTGGGGAAATGACTATTCCGTCAATATTCAGGCGCGGCAAGCAGGGGAGTTTTCG

TTAATACCGTCAAATACGCAGGTTGAACATCGTTTGCATGATCAATGGCAAAACGGTAAT

CCCCAGCGCTGGCACCTGACGCGAGACGATC

>151 bp product from linear template AP023294.1:1007953-1008960 Salmonella enterica subsp. enterica serovar 4,[5],12:i:- L-4261 DNA, complete genome, base 802 to base 952 (Forward invj - Reverse invj).

CAGCGCTGGGGAAATGACTATTCCGTCAATATTCAGGCGCGGCAAGCAGGGGAGTTTTCG

TTAATACCGTCAAATACGCAGGTTGAACATCGTTTGCATGATCAATGGCAAAACGGTAAT

CCCCAGCGCTGGCACCTGACGCGAGACGATC

>151 bp product from linear template AP023317.1:1040756-1041763 Salmonella enterica subsp. enterica serovar 4,[5],12:i:- L-4681 DNA, complete genome, base 802 to base 952 (Forward invj - Reverse invj).

CAGCGCTGGGGAAATGACTATTCCGTCAATATTCAGGCGCGGCAAGCAGGGGAGTTTTCG

TTAATACCGTCAAATACGCAGGTTGAACATCGTTTGCATGATCAATGGCAAAACGGTAAT

CCCCAGCGCTGGCACCTGACGCGAGACGATC

>151 bp product from linear template CP065131.1:1007186-1008193 Salmonella enterica subsp. enterica strain 178 chromosome, complete genome, base 802 to base 952 (Forward invj - Reverse invj).

CAGCGCTGGGGAAATGACTATTCCGTCAATATTCAGGCGCGGCAAGCAGGGGAGTTTTCG

TTAATACCGTCAAATACGCAGGTTGAACATCGTTTGCATGATCAATGGCAAAACGGTAAT

CCCCAGCGCTGGCACCTGACGCGAGACGATC

>151 bp product from linear template CP082645.1:1010457-1011464 Salmonella enterica subsp. enterica serovar Typhimurium var. 5- strain CVM N16S214 chromosome, complete genome, base 802 to base 952 (Forward invj - Reverse invj).

CAGCGCTGGGGAAATGACTATTCCGTCAATATTCAGGCGCGGCAAGCAGGGGAGTTTTCG

TTAATACCGTCAAATACGCAGGTTGAACATCGTTTGCATGATCAATGGCAAAACGGTAAT

CCCCAGCGCTGGCACCTGACGCGAGACGATC

>151 bp product from linear template CP149240.1:3089966-3090973 Salmonella enterica subsp. enterica serovar 4,[5],12:i:- strain Z1322HSL0070 chromosome, complete genome, base 802 to base 952 (Forward invj - Reverse invj).

CAGCGCTGGGGAAATGACTATTCCGTCAATATTCAGGCGCGGCAAGCAGGGGAGTTTTCG

TTAATACCGTCAAATACGCAGGTTGAACATCGTTTGCATGATCAATGGCAAAACGGTAAT

CCCCAGCGCTGGCACCTGACGCGAGACGATC

>151 bp product from linear template KP279312.1:c29889-28882 Salmonella enterica subsp. enterica serovar Typhimurium strain KC14TY170_108 specific pathogenicity island SPI-1 genomic sequence, base 802 to base 952 (Forward invj - Reverse invj).

CAGCGCTGGGGAAATGACTATTCCGTCAATATTCAGGCGCGGCAAGCAGGGGAGTTTTCG

TTAATACCGTCAAATACGCAGGTTGAACATCGTTTGCATGATCAATGGCAAAACGGTAAT

CCCCAGCGCTGGCACCTGACGCGAGACGATC

>151 bp product from linear template CP082543.1:1057568-1058575 Salmonella enterica subsp. enterica serovar Typhimurium strain CVM N18S1677 chromosome, complete genome, base 802 to base 952 (Forward invj - Reverse invj).

CAGCGCTGGGGAAATGACTATTCCGTCAATATTCAGGCGCGGCAAGCAGGGGAGTTTTCG

TTAATACCGTCAAATACGCAGGTTGAACATCGTTTGCATGATCAATGGCAAAACGGTAAT

CCCCAGCGCTGGCACCTGACGCGAGACGATC

>151 bp product from linear template CP074615.1:1001348-1002355 Salmonella enterica subsp. enterica serovar Typhimurium strain SGSC 2187 chromosome, complete genome, base 802 to base 952 (Forward invj - Reverse invj).

CAGCGCTGGGGAAATGACTATTCCGTCAATATTCAGGCGCGGCAAGCAGGGGAGTTTTCG

TTAATACCGTCAAATACGCAGGTTGAACATCGTTTGCATGATCAATGGCAAAACGGTAAT

CCCCAGCGCTGGCACCTGACGCGAGACGATC

>151 bp product from linear template CP007581.1:c3071495-3070488 Salmonella enterica subsp. enterica serovar Typhimurium strain 138736, complete genome, base 802 to base 952 (Forward invj - Reverse invj).

CAGCGCTGGGGAAATGACTATTCCGTCAATATTCAGGCGCGGCAAGCAGGGGAGTTTTCG

TTAATACCGTCAAATACGCAGGTTGAACATCGTTTGCATGATCAATGGCAAAACGGTAAT

CCCCAGCGCTGGCACCTGACGCGAGACGATC

>151 bp product from linear template CP047537.1:c3878603-3877596 Salmonella enterica subsp. enterica serovar Typhimurium strain SJTUF10405 chromosome, complete genome, base 802 to base 952 (Forward invj - Reverse invj).

CAGCGCTGGGGAAATGACTATTCCGTCAATATTCAGGCGCGGCAAGCAGGGGAGTTTTCG

TTAATACCGTCAAATACGCAGGTTGAACATCGTTTGCATGATCAATGGCAAAACGGTAAT

CCCCAGCGCTGGCACCTGACGCGAGACGATC

>151 bp product from linear template CP076095.1:1009474-1010481 Salmonella enterica strain CFSAN057220 chromosome, complete genome, base 802 to base 952 (Forward invj - Reverse invj).

CAGCGCTGGGGAAATGACTATTCCGTCAATATTCAGGCGCGGCAAGCAGGGGAGTTTTCG

TTAATACCGTCAAATACGCAGGTTGAACATCGTTTGCATGATCAATGGCAAAACGGTAAT

CCCCAGCGCTGGCACCTGACGCGAGACGATC

>151 bp product from linear template CP149194.1:1039755-1040762 Salmonella enterica subsp. enterica serovar 4,[5],12:i:- strain Z1323SSL0017 chromosome, complete genome, base 802 to base 952 (Forward invj - Reverse invj).

CAGCGCTGGGGAAATGACTATTCCGTCAATATTCAGGCGCGGCAAGCAGGGGAGTTTTCG

TTAATACCGTCAAATACGCAGGTTGAACATCGTTTGCATGATCAATGGCAAAACGGTAAT

CCCCAGCGCTGGCACCTGACGCGAGACGATC

>151 bp product from linear template CP082706.1:1039929-1040936 Salmonella enterica subsp. enterica serovar 4,[5],12:i:- strain CVM N17S380 chromosome, complete genome, base 802 to base 952 (Forward invj - Reverse invj).

CAGCGCTGGGGAAATGACTATTCCGTCAATATTCAGGCGCGGCAAGCAGGGGAGTTTTCG

TTAATACCGTCAAATACGCAGGTTGAACATCGTTTGCATGATCAATGGCAAAACGGTAAT

CCCCAGCGCTGGCACCTGACGCGAGACGATC

>151 bp product from linear template CP123703.1:c3919858-3918851 Salmonella enterica subsp. enterica serovar 4,[5],12:i:- strain FSIS11920112 chromosome, complete genome, base 802 to base 952 (Forward invj - Reverse invj).

CAGCGCTGGGGAAATGACTATTCCGTCAATATTCAGGCGCGGCAAGCAGGGGAGTTTTCG

TTAATACCGTCAAATACGCAGGTTGAACATCGTTTGCATGATCAATGGCAAAACGGTAAT

CCCCAGCGCTGGCACCTGACGCGAGACGATC

>151 bp product from linear template CP149400.1:c3623409-3622402 Salmonella enterica subsp. enterica serovar Typhimurium strain Z1323CSL0045 chromosome, complete genome, base 802 to base 952 (Forward invj - Reverse invj).

CAGCGCTGGGGAAATGACTATTCCGTCAATATTCAGGCGCGGCAAGCAGGGGAGTTTTCG

TTAATACCGTCAAATACGCAGGTTGAACATCGTTTGCATGATCAATGGCAAAACGGTAAT

CCCCAGCGCTGGCACCTGACGCGAGACGATC

>151 bp product from linear template CP006048.1:532919-533926 Salmonella enterica subsp. enterica serovar Typhimurium var. 5- str. CFSAN001921, complete genome, base 802 to base 952 (Forward invj - Reverse invj).

CAGCGCTGGGGAAATGACTATTCCGTCAATATTCAGGCGCGGCAAGCAGGGGAGTTTTCG

TTAATACCGTCAAATACGCAGGTTGAACATCGTTTGCATGATCAATGGCAAAACGGTAAT

CCCCAGCGCTGGCACCTGACGCGAGACGATC

>151 bp product from linear template CP117400.1:1009689-1010696 Salmonella enterica subsp. enterica serovar Typhimurium strain RM014 chromosome, complete genome, base 802 to base 952 (Forward invj - Reverse invj).

CAGCGCTGGGGAAATGACTATTCCGTCAATATTCAGGCGCGGCAAGCAGGGGAGTTTTCG

TTAATACCGTCAAATACGCAGGTTGAACATCGTTTGCATGATCAATGGCAAAACGGTAAT

CCCCAGCGCTGGCACCTGACGCGAGACGATC

>151 bp product from linear template CP053055.1:1024327-1025334 Salmonella enterica subsp. enterica serovar Saintpaul strain CVM 22543 chromosome, complete genome, base 802 to base 952 (Forward invj - Reverse invj).

CAGCGCTGGGGAAATGACTATTCCGTCAATATTCAGGCGCGGCAAGCAGGGGAGTTTTCG

TTAATACCGTCAAATACGCAGGTTGAACATCGTTTGCATGATCAATGGCAAAACGGTAAT

CCCCAGCGCTGGCACCTGACGCGAGACGATC

>151 bp product from linear template OU015325.1:1039759-1040766 Salmonella enterica subsp. enterica serovar Typhimurium strain AUSMDU00005182 genome assembly, chromosome: C1, base 802 to base 952 (Forward invj - Reverse invj).

CAGCGCTGGGGAAATGACTATTCCGTCAATATTCAGGCGCGGCAAGCAGGGGAGTTTTCG

TTAATACCGTCAAATACGCAGGTTGAACATCGTTTGCATGATCAATGGCAAAACGGTAAT

CCCCAGCGCTGGCACCTGACGCGAGACGATC

>151 bp product from linear template CP040651.1:c3073468-3072461 Salmonella enterica subsp. enterica serovar 1,4,[5],12:i:- strain SA20070548 chromosome, complete genome, base 802 to base 952 (Forward invj - Reverse invj).

CAGCGCTGGGGAAATGACTATTCCGTCAATATTCAGGCGCGGCAAGCAGGGGAGTTTTCG

TTAATACCGTCAAATACGCAGGTTGAACATCGTTTGCATGATCAATGGCAAAACGGTAAT

CCCCAGCGCTGGCACCTGACGCGAGACGATC

>151 bp product from linear template U43300.1:444-1451 Salmonella enterica invasion gene complex SpaM and SpaN genes, complete cds, base 802 to base 952 (Forward invj - Reverse invj).

CAGCGCTGGGGAAATGACTATTCCGTCAATATTCAGGCGCGGCAAGCAGGGGAGTTTTCG

TTAATACCGTCAAATACGCAGGTTGAACATCGTTTGCATGATCAATGGCAAAACGGTAAT

CCCCAGCGCTGGCACCTGACGCGAGACGATC

>151 bp product from linear template CP082523.1:1052714-1053721 Salmonella enterica subsp. enterica serovar Typhimurium strain CVM N18S2188 chromosome, complete genome, base 802 to base 952 (Forward invj - Reverse invj).

CAGCGCTGGGGAAATGACTATTCCGTCAATATTCAGGCGCGGCAAGCAGGGGAGTTTTCG

TTAATACCGTCAAATACGCAGGTTGAACATCGTTTGCATGATCAATGGCAAAACGGTAAT

CCCCAGCGCTGGCACCTGACGCGAGACGATC

>151 bp product from linear template CP074280.1:1198470-1199477 Salmonella enterica subsp. enterica serovar Saintpaul strain CFSAN006241 chromosome, complete genome, base 802 to base 952 (Forward invj - Reverse invj).

CAGCGCTGGGGAAATGACTATTCCGTCAATATTCAGGCGCGGCAAGCAGGGGAGTTTTCG

TTAATACCGTCAAATACGCAGGTTGAACATCGTTTGCATGATCAATGGCAAAACGGTAAT

CCCCAGCGCTGGCACCTGACGCGAGACGATC

>151 bp product from linear template CP149368.1:559925-560932 Salmonella enterica subsp. enterica serovar Typhimurium strain Z1323HSL0042 chromosome, complete genome, base 802 to base 952 (Forward invj - Reverse invj).

CAGCGCTGGGGAAATGACTATTCCGTCAATATTCAGGCGCGGCAAGCAGGGGAGTTTTCG

TTAATACCGTCAAATACGCAGGTTGAACATCGTTTGCATGATCAATGGCAAAACGGTAAT

CCCCAGCGCTGGCACCTGACGCGAGACGATC

>151 bp product from linear template CP102424.1:1039719-1040726 Salmonella enterica subsp. enterica serovar Gloucester strain GSJ/2017-Sal-014 chromosome, complete genome, base 802 to base 952 (Forward invj - Reverse invj).

CAGCGCTGGGGAAATGACTATTCCGTCAATATTCAGGCGCGGCAAGCAGGGGAGTTTTCG

TTAATACCGTCAAATACGCAGGTTGAACATCGTTTGCATGATCAATGGCAAAACGGTAAT

CCCCAGCGCTGGCACCTGACGCGAGACGATC

>151 bp product from linear template CP028314.1:1010138-1011145 Salmonella enterica subsp. enterica serovar Typhimurium var. 5- strain CFSAN067217 chromosome, complete genome, base 802 to base 952 (Forward invj - Reverse invj).

CAGCGCTGGGGAAATGACTATTCCGTCAATATTCAGGCGCGGCAAGCAGGGGAGTTTTCG

TTAATACCGTCAAATACGCAGGTTGAACATCGTTTGCATGATCAATGGCAAAACGGTAAT

CCCCAGCGCTGGCACCTGACGCGAGACGATC

>151 bp product from linear template CP104805.1:1039615-1040622 Salmonella enterica subsp. enterica serovar Typhimurium strain STMC246 chromosome, complete genome, base 802 to base 952 (Forward invj - Reverse invj).

CAGCGCTGGGGAAATGACTATTCCGTCAATATTCAGGCGCGGCAAGCAGGGGAGTTTTCG

TTAATACCGTCAAATACGCAGGTTGAACATCGTTTGCATGATCAATGGCAAAACGGTAAT

CCCCAGCGCTGGCACCTGACGCGAGACGATC

>151 bp product from linear template CP047546.1:1039852-1040859 Salmonella enterica subsp. enterica serovar Typhimurium strain SJTUF10236 chromosome, complete genome, base 802 to base 952 (Forward invj - Reverse invj).

CAGCGCTGGGGAAATGACTATTCCGTCAATATTCAGGCGCGGCAAGCAGGGGAGTTTTCG

TTAATACCGTCAAATACGCAGGTTGAACATCGTTTGCATGATCAATGGCAAAACGGTAAT

CCCCAGCGCTGGCACCTGACGCGAGACGATC

>151 bp product from linear template LR861808.1:c3096152-3095145 Salmonella enterica subsp. enterica serovar Typhimurium isolate 5d8c9f00c6184d00f9da85a6:sample:O1960-05 genome assembly, chromosome: 1, base 802 to base 952 (Forward invj - Reverse invj).

CAGCGCTGGGGAAATGACTATTCCGTCAATATTCAGGCGCGGCAAGCAGGGGAGTTTTCG

TTAATACCGTCAAATACGCAGGTTGAACATCGTTTGCATGATCAATGGCAAAACGGTAAT

CCCCAGCGCTGGCACCTGACGCGAGACGATC

>151 bp product from linear template KP279310.1:c29886-28879 Salmonella enterica subsp. enterica serovar Typhimurium strain KC14TY44 specific pathogenicity island SPI-1 genomic sequence, base 802 to base 952 (Forward invj - Reverse invj).

CAGCGCTGGGGAAATGACTATTCCGTCAATATTCAGGCGCGGCAAGCAGGGGAGTTTTCG

TTAATACCGTCAAATACGCAGGTTGAACATCGTTTGCATGATCAATGGCAAAACGGTAAT

CCCCAGCGCTGGCACCTGACGCGAGACGATC

>151 bp product from linear template CP100707.1:1039958-1040965 Salmonella enterica subsp. enterica serovar Typhimurium strain R18.0409 chromosome, complete genome, base 802 to base 952 (Forward invj - Reverse invj).

CAGCGCTGGGGAAATGACTATTCCGTCAATATTCAGGCGCGGCAAGCAGGGGAGTTTTCG

TTAATACCGTCAAATACGCAGGTTGAACATCGTTTGCATGATCAATGGCAAAACGGTAAT

CCCCAGCGCTGGCACCTGACGCGAGACGATC

>151 bp product from linear template CP003386.1:c3057572-3056565 Salmonella enterica subsp. enterica serovar Typhimurium str. 798, complete genome, base 802 to base 952 (Forward invj - Reverse invj).

CAGCGCTGGGGAAATGACTATTCCGTCAATATTCAGGCGCGGCAAGCAGGGGAGTTTTCG

TTAATACCGTCAAATACGCAGGTTGAACATCGTTTGCATGATCAATGGCAAAACGGTAAT

CCCCAGCGCTGGCACCTGACGCGAGACGATC

>151 bp product from linear template CP035547.1:c3044331-3043324 Salmonella enterica subsp. enterica serovar Typhimurium strain YU07-18 chromosome, complete genome, base 802 to base 952 (Forward invj - Reverse invj).

CAGCGCTGGGGAAATGACTATTCCGTCAATATTCAGGCGCGGCAAGCAGGGGAGTTTTCG

TTAATACCGTCAAATACGCAGGTTGAACATCGTTTGCATGATCAATGGCAAAACGGTAAT

CCCCAGCGCTGGCACCTGACGCGAGACGATC

>151 bp product from linear template CP149327.1:1043647-1044654 Salmonella enterica subsp. enterica serovar Typhimurium strain Z1323SSL0026 chromosome, complete genome, base 802 to base 952 (Forward invj - Reverse invj).

CAGCGCTGGGGAAATGACTATTCCGTCAATATTCAGGCGCGGCAAGCAGGGGAGTTTTCG

TTAATACCGTCAAATACGCAGGTTGAACATCGTTTGCATGATCAATGGCAAAACGGTAAT

CCCCAGCGCTGGCACCTGACGCGAGACGATC

>151 bp product from linear template CP085820.1:c696201-695194 Salmonella enterica subsp. enterica serovar Typhimurium strain Wartortle chromosome, complete genome, base 802 to base 952 (Forward invj - Reverse invj).

CAGCGCTGGGGAAATGACTATTCCGTCAATATTCAGGCGCGGCAAGCAGGGGAGTTTTCG

TTAATACCGTCAAATACGCAGGTTGAACATCGTTTGCATGATCAATGGCAAAACGGTAAT

CCCCAGCGCTGGCACCTGACGCGAGACGATC

>151 bp product from linear template CP039567.1:c3002019-3001012 Salmonella enterica subsp. enterica serovar 1,4,[5],12:i:- strain PNCS014850 chromosome, complete genome, base 802 to base 952 (Forward invj - Reverse invj).

CAGCGCTGGGGAAATGACTATTCCGTCAATATTCAGGCGCGGCAAGCAGGGGAGTTTTCG

TTAATACCGTCAAATACGCAGGTTGAACATCGTTTGCATGATCAATGGCAAAACGGTAAT

CCCCAGCGCTGGCACCTGACGCGAGACGATC

>151 bp product from linear template CP035301.1:1007721-1008728 Salmonella enterica subsp. enterica strain ST1539 chromosome, complete genome, base 802 to base 952 (Forward invj - Reverse invj).

CAGCGCTGGGGAAATGACTATTCCGTCAATATTCAGGCGCGGCAAGCAGGGGAGTTTTCG

TTAATACCGTCAAATACGCAGGTTGAACATCGTTTGCATGATCAATGGCAAAACGGTAAT

CCCCAGCGCTGGCACCTGACGCGAGACGATC

>151 bp product from linear template CP043399.1:c3057925-3056918 Salmonella enterica subsp. enterica serovar Typhimurium str. 14028S substr. GXS275 chromosome, complete genome, base 802 to base 952 (Forward invj - Reverse invj).

CAGCGCTGGGGAAATGACTATTCCGTCAATATTCAGGCGCGGCAAGCAGGGGAGTTTTCG

TTAATACCGTCAAATACGCAGGTTGAACATCGTTTGCATGATCAATGGCAAAACGGTAAT

CCCCAGCGCTGGCACCTGACGCGAGACGATC

>151 bp product from linear template KP279311.1:c29889-28882 Salmonella enterica subsp. enterica serovar Typhimurium strain KC14TY135 specific pathogenicity island SPI-1 genomic sequence, base 802 to base 952 (Forward invj - Reverse invj).

CAGCGCTGGGGAAATGACTATTCCGTCAATATTCAGGCGCGGCAAGCAGGGGAGTTTTCG

TTAATACCGTCAAATACGCAGGTTGAACATCGTTTGCATGATCAATGGCAAAACGGTAAT

CCCCAGCGCTGGCACCTGACGCGAGACGATC

>151 bp product from linear template CP149214.1:c2712806-2711799 Salmonella enterica subsp. enterica serovar 4,[5],12:i:- strain Z1323HSL0045 chromosome, complete genome, base 802 to base 952 (Forward invj - Reverse invj).

CAGCGCTGGGGAAATGACTATTCCGTCAATATTCAGGCGCGGCAAGCAGGGGAGTTTTCG

TTAATACCGTCAAATACGCAGGTTGAACATCGTTTGCATGATCAATGGCAAAACGGTAAT

CCCCAGCGCTGGCACCTGACGCGAGACGATC

>151 bp product from linear template CP061126.1:1094247-1095254 Salmonella enterica subsp. enterica serovar Typhimurium strain S304 chromosome, complete genome, base 802 to base 952 (Forward invj - Reverse invj).

CAGCGCTGGGGAAATGACTATTCCGTCAATATTCAGGCGCGGCAAGCAGGGGAGTTTTCG

TTAATACCGTCAAATACGCAGGTTGAACATCGTTTGCATGATCAATGGCAAAACGGTAAT

CCCCAGCGCTGGCACCTGACGCGAGACGATC

>151 bp product from linear template HG326213.1:c2995252-2994245 Salmonella enterica subsp. enterica serovar Typhimurium str. DT2, complete genome, base 802 to base 952 (Forward invj - Reverse invj).

CAGCGCTGGGGAAATGACTATTCCGTCAATATTCAGGCGCGGCAAGCAGGGGAGTTTTCG

TTAATACCGTCAAATACGCAGGTTGAACATCGTTTGCATGATCAATGGCAAAACGGTAAT

CCCCAGCGCTGGCACCTGACGCGAGACGATC

>151 bp product from linear template CP039572.1:c3079548-3078541 Salmonella enterica subsp. enterica serovar 1,4,[5],12:i:- strain PNCS014855 chromosome, complete genome, base 802 to base 952 (Forward invj - Reverse invj).

CAGCGCTGGGGAAATGACTATTCCGTCAATATTCAGGCGCGGCAAGCAGGGGAGTTTTCG

TTAATACCGTCAAATACGCAGGTTGAACATCGTTTGCATGATCAATGGCAAAACGGTAAT

CCCCAGCGCTGGCACCTGACGCGAGACGATC

>151 bp product from linear template CP149196.1:1039955-1040962 Salmonella enterica subsp. enterica serovar 4,[5],12:i:- strain Z1323SSL0016 chromosome, complete genome, base 802 to base 952 (Forward invj - Reverse invj).

CAGCGCTGGGGAAATGACTATTCCGTCAATATTCAGGCGCGGCAAGCAGGGGAGTTTTCG

TTAATACCGTCAAATACGCAGGTTGAACATCGTTTGCATGATCAATGGCAAAACGGTAAT

CCCCAGCGCTGGCACCTGACGCGAGACGATC

>151 bp product from linear template CP014971.2:1047837-1048844 Salmonella enterica subsp. enterica serovar Typhimurium str. USDA-ARS-USMARC-1898, complete genome, base 802 to base 952 (Forward invj - Reverse invj).

CAGCGCTGGGGAAATGACTATTCCGTCAATATTCAGGCGCGGCAAGCAGGGGAGTTTTCG

TTAATACCGTCAAATACGCAGGTTGAACATCGTTTGCATGATCAATGGCAAAACGGTAAT

CCCCAGCGCTGGCACCTGACGCGAGACGATC

>151 bp product from linear template CP133487.1:1007765-1008772 Salmonella enterica strain 94 chromosome, complete genome, base 802 to base 952 (Forward invj - Reverse invj).

CAGCGCTGGGGAAATGACTATTCCGTCAATATTCAGGCGCGGCAAGCAGGGGAGTTTTCG

TTAATACCGTCAAATACGCAGGTTGAACATCGTTTGCATGATCAATGGCAAAACGGTAAT

CCCCAGCGCTGGCACCTGACGCGAGACGATC

>151 bp product from linear template CP149347.1:529395-530402 Salmonella enterica subsp. enterica serovar Typhimurium strain Z1323HSL0086 chromosome, complete genome, base 802 to base 952 (Forward invj - Reverse invj).

CAGCGCTGGGGAAATGACTATTCCGTCAATATTCAGGCGCGGCAAGCAGGGGAGTTTTCG

TTAATACCGTCAAATACGCAGGTTGAACATCGTTTGCATGATCAATGGCAAAACGGTAAT

CCCCAGCGCTGGCACCTGACGCGAGACGATC

>151 bp product from linear template CP082421.2:c3799451-3798444 Salmonella enterica subsp. enterica serovar Typhimurium strain FSIS11704063 chromosome, complete genome, base 802 to base 952 (Forward invj - Reverse invj).

CAGCGCTGGGGAAATGACTATTCCGTCAATATTCAGGCGCGGCAAGCAGGGGAGTTTTCG

TTAATACCGTCAAATACGCAGGTTGAACATCGTTTGCATGATCAATGGCAAAACGGTAAT

CCCCAGCGCTGGCACCTGACGCGAGACGATC

>151 bp product from linear template CP091560.1:1038221-1039228 Salmonella enterica strain 179 chromosome, complete genome, base 802 to base 952 (Forward invj - Reverse invj).

CAGCGCTGGGGAAATGACTATTCCGTCAATATTCAGGCGCGGCAAGCAGGGGAGTTTTCG

TTAATACCGTCAAATACGCAGGTTGAACATCGTTTGCATGATCAATGGCAAAACGGTAAT

CCCCAGCGCTGGCACCTGACGCGAGACGATC

>151 bp product from linear template CP074620.1:1009118-1010125 Salmonella enterica strain CFSAN029958 chromosome, complete genome, base 802 to base 952 (Forward invj - Reverse invj).

CAGCGCTGGGGAAATGACTATTCCGTCAATATTCAGGCGCGGCAAGCAGGGGAGTTTTCG

TTAATACCGTCAAATACGCAGGTTGAACATCGTTTGCATGATCAATGGCAAAACGGTAAT

CCCCAGCGCTGGCACCTGACGCGAGACGATC

>151 bp product from linear template CP027414.1:1887498-1888505 Salmonella enterica subsp. enterica serovar Typhimurium strain FDAARGOS_320 chromosome, complete genome, base 802 to base 952 (Forward invj - Reverse invj).

CAGCGCTGGGGAAATGACTATTCCGTCAATATTCAGGCGCGGCAAGCAGGGGAGTTTTCG

TTAATACCGTCAAATACGCAGGTTGAACATCGTTTGCATGATCAATGGCAAAACGGTAAT

CCCCAGCGCTGGCACCTGACGCGAGACGATC

>151 bp product from linear template CP014982.2:1079154-1080161 Salmonella enterica subsp. enterica serovar Typhimurium str. USDA-ARS-USMARC-1810, complete genome, base 802 to base 952 (Forward invj - Reverse invj).

CAGCGCTGGGGAAATGACTATTCCGTCAATATTCAGGCGCGGCAAGCAGGGGAGTTTTCG

TTAATACCGTCAAATACGCAGGTTGAACATCGTTTGCATGATCAATGGCAAAACGGTAAT

CCCCAGCGCTGGCACCTGACGCGAGACGATC

>151 bp product from linear template OU015328.1:1042788-1043795 Salmonella enterica subsp. enterica serovar Typhimurium strain AUSMDU00027951 genome assembly, chromosome: C1, base 802 to base 952 (Forward invj - Reverse invj).

CAGCGCTGGGGAAATGACTATTCCGTCAATATTCAGGCGCGGCAAGCAGGGGAGTTTTCG

TTAATACCGTCAAATACGCAGGTTGAACATCGTTTGCATGATCAATGGCAAAACGGTAAT

CCCCAGCGCTGGCACCTGACGCGAGACGATC

>151 bp product from linear template CP038849.1:c2988864-2987857 Salmonella enterica subsp. enterica serovar 1,4,[5],12:i:- strain PNCS014866 chromosome, complete genome, base 802 to base 952 (Forward invj - Reverse invj).

CAGCGCTGGGGAAATGACTATTCCGTCAATATTCAGGCGCGGCAAGCAGGGGAGTTTTCG

TTAATACCGTCAAATACGCAGGTTGAACATCGTTTGCATGATCAATGGCAAAACGGTAAT

CCCCAGCGCTGGCACCTGACGCGAGACGATC

>151 bp product from linear template CP007523.1:c3721055-3720048 Salmonella enterica subsp. enterica serovar Typhimurium str. CDC 2011K-0870, complete genome, base 802 to base 952 (Forward invj - Reverse invj).

CAGCGCTGGGGAAATGACTATTCCGTCAATATTCAGGCGCGGCAAGCAGGGGAGTTTTCG

TTAATACCGTCAAATACGCAGGTTGAACATCGTTTGCATGATCAATGGCAAAACGGTAAT

CCCCAGCGCTGGCACCTGACGCGAGACGATC

>151 bp product from linear template CP034968.1:3725959-3726966 Salmonella enterica subsp. enterica serovar Typhimurium strain B3589 chromosome, complete genome, base 802 to base 952 (Forward invj - Reverse invj).

CAGCGCTGGGGAAATGACTATTCCGTCAATATTCAGGCGCGGCAAGCAGGGGAGTTTTCG

TTAATACCGTCAAATACGCAGGTTGAACATCGTTTGCATGATCAATGGCAAAACGGTAAT

CCCCAGCGCTGGCACCTGACGCGAGACGATC

>151 bp product from linear template CP074309.1:1005477-1006484 Salmonella enterica subsp. enterica strain CFSAN004090 chromosome, complete genome, base 802 to base 952 (Forward invj - Reverse invj).

CAGCGCTGGGGAAATGACTATTCCGTCAATATTCAGGCGCGGCAAGCAGGGGAGTTTTCG

TTAATACCGTCAAATACGCAGGTTGAACATCGTTTGCATGATCAATGGCAAAACGGTAAT

CCCCAGCGCTGGCACCTGACGCGAGACGATC

>151 bp product from linear template CP091556.1:1043031-1044038 Salmonella enterica strain 632 chromosome, complete genome, base 802 to base 952 (Forward invj - Reverse invj).

CAGCGCTGGGGAAATGACTATTCCGTCAATATTCAGGCGCGGCAAGCAGGGGAGTTTTCG

TTAATACCGTCAAATACGCAGGTTGAACATCGTTTGCATGATCAATGGCAAAACGGTAAT

CCCCAGCGCTGGCACCTGACGCGAGACGATC

>151 bp product from linear template CP082650.1:1039527-1040534 Salmonella enterica subsp. enterica serovar 4,[5],12:i:- strain CVM N16S144 chromosome, complete genome, base 802 to base 952 (Forward invj - Reverse invj).

CAGCGCTGGGGAAATGACTATTCCGTCAATATTCAGGCGCGGCAAGCAGGGGAGTTTTCG

TTAATACCGTCAAATACGCAGGTTGAACATCGTTTGCATGATCAATGGCAAAACGGTAAT

CCCCAGCGCTGGCACCTGACGCGAGACGATC

>151 bp product from linear template CP039585.1:c2994814-2993807 Salmonella enterica subsp. enterica serovar 1,4,[5],12:i:- strain PNCS014859 chromosome, complete genome, base 802 to base 952 (Forward invj - Reverse invj).

CAGCGCTGGGGAAATGACTATTCCGTCAATATTCAGGCGCGGCAAGCAGGGGAGTTTTCG

TTAATACCGTCAAATACGCAGGTTGAACATCGTTTGCATGATCAATGGCAAAACGGTAAT

CCCCAGCGCTGGCACCTGACGCGAGACGATC

>151 bp product from linear template CP047527.1:c3959226-3958219 Salmonella enterica subsp. enterica serovar Typhimurium strain SJTUF10648 chromosome, complete genome, base 802 to base 952 (Forward invj - Reverse invj).

CAGCGCTGGGGAAATGACTATTCCGTCAATATTCAGGCGCGGCAAGCAGGGGAGTTTTCG

TTAATACCGTCAAATACGCAGGTTGAACATCGTTTGCATGATCAATGGCAAAACGGTAAT

CCCCAGCGCTGGCACCTGACGCGAGACGATC

>151 bp product from linear template CP070321.1:c2939977-2938970 Salmonella enterica subsp. enterica serovar Typhimurium strain Colony113 chromosome, base 802 to base 952 (Forward invj - Reverse invj).

CAGCGCTGGGGAAATGACTATTCCGTCAATATTCAGGCGCGGCAAGCAGGGGAGTTTTCG

TTAATACCGTCAAATACGCAGGTTGAACATCGTTTGCATGATCAATGGCAAAACGGTAAT

CCCCAGCGCTGGCACCTGACGCGAGACGATC

>151 bp product from linear template CP068696.1:1478810-1479817 Salmonella enterica subsp. enterica serovar Typhimurium strain NCCP16345 chromosome, complete genome, base 802 to base 952 (Forward invj - Reverse invj).

CAGCGCTGGGGAAATGACTATTCCGTCAATATTCAGGCGCGGCAAGCAGGGGAGTTTTCG

TTAATACCGTCAAATACGCAGGTTGAACATCGTTTGCATGATCAATGGCAAAACGGTAAT

CCCCAGCGCTGGCACCTGACGCGAGACGATC

>151 bp product from linear template CP149136.1:1039718-1040725 Salmonella enterica subsp. enterica serovar 4,[5],12:i:- strain Z1323SSL0064 chromosome, complete genome, base 802 to base 952 (Forward invj - Reverse invj).

CAGCGCTGGGGAAATGACTATTCCGTCAATATTCAGGCGCGGCAAGCAGGGGAGTTTTCG

TTAATACCGTCAAATACGCAGGTTGAACATCGTTTGCATGATCAATGGCAAAACGGTAAT

CCCCAGCGCTGGCACCTGACGCGAGACGATC

>151 bp product from linear template CP006602.1:c4292046-4291039 Salmonella enterica subsp. enterica serovar 4,[5],12:i:- str. 08-1736 chromosome, complete genome, base 802 to base 952 (Forward invj - Reverse invj).

CAGCGCTGGGGAAATGACTATTCCGTCAATATTCAGGCGCGGCAAGCAGGGGAGTTTTCG

TTAATACCGTCAAATACGCAGGTTGAACATCGTTTGCATGATCAATGGCAAAACGGTAAT

CCCCAGCGCTGGCACCTGACGCGAGACGATC

>151 bp product from linear template CP173644.1:c2888241-2887234 Salmonella enterica subsp. enterica serovar Enteritidis strain S-28445 chromosome, complete genome, base 802 to base 952 (Forward invj - Reverse invj).

CAGCGCTGGGGAAATGACTATTCCGTCAATATTCAGGCGCGGCAAGCAGGGGAGTTTTCG

TTAATACCGTCAAATACGCAGGTTGAACATCGTTTGCATGATCAATGGCAAAACGGTAAT

CCCCAGCGCTGGCACCTGACGCGAGACGATC

>151 bp product from linear template CP117404.1:1009602-1010609 Salmonella enterica subsp. enterica serovar Typhimurium strain RM085 chromosome, complete genome, base 802 to base 952 (Forward invj - Reverse invj).

CAGCGCTGGGGAAATGACTATTCCGTCAATATTCAGGCGCGGCAAGCAGGGGAGTTTTCG

TTAATACCGTCAAATACGCAGGTTGAACATCGTTTGCATGATCAATGGCAAAACGGTAAT

CCCCAGCGCTGGCACCTGACGCGAGACGATC

>151 bp product from linear template CP113535.1:1120623-1121630 Salmonella enterica strain ZLQ chromosome, complete genome, base 802 to base 952 (Forward invj - Reverse invj).

CAGCGCTGGGGAAATGACTATTCCGTCAATATTCAGGCGCGGCAAGCAGGGGAGTTTTCG

TTAATACCGTCAAATACGCAGGTTGAACATCGTTTGCATGATCAATGGCAAAACGGTAAT

CCCCAGCGCTGGCACCTGACGCGAGACGATC

>151 bp product from linear template CP149192.1:1039961-1040968 Salmonella enterica subsp. enterica serovar 4,[5],12:i:- strain Z1323SSL0018 chromosome, complete genome, base 802 to base 952 (Forward invj - Reverse invj).

CAGCGCTGGGGAAATGACTATTCCGTCAATATTCAGGCGCGGCAAGCAGGGGAGTTTTCG

TTAATACCGTCAAATACGCAGGTTGAACATCGTTTGCATGATCAATGGCAAAACGGTAAT

CCCCAGCGCTGGCACCTGACGCGAGACGATC

>151 bp product from linear template AP023309.1:1008027-1009034 Salmonella enterica subsp. enterica serovar 4,[5],12:i:- L-4578 DNA, complete genome, base 802 to base 952 (Forward invj - Reverse invj).

CAGCGCTGGGGAAATGACTATTCCGTCAATATTCAGGCGCGGCAAGCAGGGGAGTTTTCG

TTAATACCGTCAAATACGCAGGTTGAACATCGTTTGCATGATCAATGGCAAAACGGTAAT

CCCCAGCGCTGGCACCTGACGCGAGACGATC

>151 bp product from linear template CP022168.1:1040530-1041537 Salmonella enterica subsp. enterica serovar Typhimurium strain WW012 chromosome, complete genome, base 802 to base 952 (Forward invj - Reverse invj).

CAGCGCTGGGGAAATGACTATTCCGTCAATATTCAGGCGCGGCAAGCAGGGGAGTTTTCG

TTAATACCGTCAAATACGCAGGTTGAACATCGTTTGCATGATCAATGGCAAAACGGTAAT

CCCCAGCGCTGGCACCTGACGCGAGACGATC

>151 bp product from linear template CP082624.1:1039931-1040938 Salmonella enterica subsp. enterica serovar 4,[5],12:i:- strain CVM N17S1465 chromosome, complete genome, base 802 to base 952 (Forward invj - Reverse invj).

CAGCGCTGGGGAAATGACTATTCCGTCAATATTCAGGCGCGGCAAGCAGGGGAGTTTTCG

TTAATACCGTCAAATACGCAGGTTGAACATCGTTTGCATGATCAATGGCAAAACGGTAAT

CCCCAGCGCTGGCACCTGACGCGAGACGATC

>151 bp product from linear template CP050734.1:1062300-1063307 Salmonella enterica subsp. enterica serovar Typhimurium strain ST90 chromosome, complete genome, base 802 to base 952 (Forward invj - Reverse invj).

CAGCGCTGGGGAAATGACTATTCCGTCAATATTCAGGCGCGGCAAGCAGGGGAGTTTTCG

TTAATACCGTCAAATACGCAGGTTGAACATCGTTTGCATGATCAATGGCAAAACGGTAAT

CCCCAGCGCTGGCACCTGACGCGAGACGATC

>151 bp product from linear template CP136400.1:1008579-1009586 Salmonella enterica strain JE4313 chromosome, complete genome, base 802 to base 952 (Forward invj - Reverse invj).

CAGCGCTGGGGAAATGACTATTCCGTCAATATTCAGGCGCGGCAAGCAGGGGAGTTTTCG

TTAATACCGTCAAATACGCAGGTTGAACATCGTTTGCATGATCAATGGCAAAACGGTAAT

CCCCAGCGCTGGCACCTGACGCGAGACGATC

>151 bp product from linear template CP075372.1:1089881-1090888 Salmonella enterica strain no75 chromosome, complete genome, base 802 to base 952 (Forward invj - Reverse invj).

CAGCGCTGGGGAAATGACTATTCCGTCAATATTCAGGCGCGGCAAGCAGGGGAGTTTTCG

TTAATACCGTCAAATACGCAGGTTGAACATCGTTTGCATGATCAATGGCAAAACGGTAAT

CCCCAGCGCTGGCACCTGACGCGAGACGATC

>151 bp product from linear template CP169304.1:c1147581-1146574 Salmonella enterica subsp. enterica serovar Typhimurium strain K79 chromosome, base 802 to base 952 (Forward invj - Reverse invj).

CAGCGCTGGGGAAATGACTATTCCGTCAATATTCAGGCGCGGCAAGCAGGGGAGTTTTCG

TTAATACCGTCAAATACGCAGGTTGAACATCGTTTGCATGATCAATGGCAAAACGGTAAT

CCCCAGCGCTGGCACCTGACGCGAGACGATC

>151 bp product from linear template CP148775.1:4787902-4788909 Salmonella enterica subsp. enterica serovar Infantis strain Z1323HSL0107 chromosome, complete genome, base 802 to base 952 (Forward invj - Reverse invj).

CAGCGCTGGGGAAATGACTATTCCGTCAATATTCAGGCGCGGCAAGCAGGGGAGTTTTCG

TTAATACCGTCAAATACGCAGGTTGAACATCGTTTGCATGATCAATGGCAAAACGGTAAT

CCCCAGCGCTGGCACCTGACGCGAGACGATC

>151 bp product from linear template CP154471.1:1392297-1393304 Salmonella enterica subsp. enterica serovar Typhimurium strain ST19 chromosome, base 802 to base 952 (Forward invj - Reverse invj).

CAGCGCTGGGGAAATGACTATTCCGTCAATATTCAGGCGCGGCAAGCAGGGGAGTTTTCG

TTAATACCGTCAAATACGCAGGTTGAACATCGTTTGCATGATCAATGGCAAAACGGTAAT

CCCCAGCGCTGGCACCTGACGCGAGACGATC

>151 bp product from linear template CP061115.1:c3704773-3703766 Salmonella enterica subsp. enterica serovar Typhimurium strain S585 chromosome, complete genome, base 802 to base 952 (Forward invj - Reverse invj).

CAGCGCTGGGGAAATGACTATTCCGTCAATATTCAGGCGCGGCAAGCAGGGGAGTTTTCG

TTAATACCGTCAAATACGCAGGTTGAACATCGTTTGCATGATCAATGGCAAAACGGTAAT

CCCCAGCGCTGGCACCTGACGCGAGACGATC

>151 bp product from linear template CP038434.1:c1730182-1729175 Salmonella enterica subsp. enterica serovar Typhimurium strain E40V chromosome, complete genome, base 802 to base 952 (Forward invj - Reverse invj).

CAGCGCTGGGGAAATGACTATTCCGTCAATATTCAGGCGCGGCAAGCAGGGGAGTTTTCG

TTAATACCGTCAAATACGCAGGTTGAACATCGTTTGCATGATCAATGGCAAAACGGTAAT

CCCCAGCGCTGGCACCTGACGCGAGACGATC

>151 bp product from linear template CP176413.1:c4864528-4863521 Salmonella enterica strain S2023359 chromosome, base 802 to base 952 (Forward invj - Reverse invj).

CAGCGCTGGGGAAATGACTATTCCGTCAATATTCAGGCGCGGCAAGCAGGGGAGTTTTCG

TTAATACCGTCAAATACGCAGGTTGAACATCGTTTGCATGATCAATGGCAAAACGGTAAT

CCCCAGCGCTGGCACCTGACGCGAGACGATC

>151 bp product from linear template CP101372.1:1382779-1383786 Salmonella enterica strain SC2016025 chromosome, complete genome, base 802 to base 952 (Forward invj - Reverse invj).

CAGCGCTGGGGAAATGACTATTCCGTCAATATTCAGGCGCGGCAAGCAGGGGAGTTTTCG

TTAATACCGTCAAATACGCAGGTTGAACATCGTTTGCATGATCAATGGCAAAACGGTAAT

CCCCAGCGCTGGCACCTGACGCGAGACGATC

>151 bp product from linear template CP149388.1:c3625916-3624909 Salmonella enterica subsp. enterica serovar Typhimurium strain Z1323CSL0053 chromosome, complete genome, base 802 to base 952 (Forward invj - Reverse invj).

CAGCGCTGGGGAAATGACTATTCCGTCAATATTCAGGCGCGGCAAGCAGGGGAGTTTTCG

TTAATACCGTCAAATACGCAGGTTGAACATCGTTTGCATGATCAATGGCAAAACGGTAAT

CCCCAGCGCTGGCACCTGACGCGAGACGATC

>151 bp product from linear template OU015342.1:1039559-1040566 Salmonella enterica subsp. enterica serovar Typhimurium strain AUSMDU00007171 genome assembly, chromosome: C1, base 802 to base 952 (Forward invj - Reverse invj).

CAGCGCTGGGGAAATGACTATTCCGTCAATATTCAGGCGCGGCAAGCAGGGGAGTTTTCG

TTAATACCGTCAAATACGCAGGTTGAACATCGTTTGCATGATCAATGGCAAAACGGTAAT

CCCCAGCGCTGGCACCTGACGCGAGACGATC

>151 bp product from linear template CP104368.1:1007001-1008008 Salmonella enterica strain PNUSAS048232 chromosome, complete genome, base 802 to base 952 (Forward invj - Reverse invj).

CAGCGCTGGGGAAATGACTATTCCGTCAATATTCAGGCGCGGCAAGCAGGGGAGTTTTCG

TTAATACCGTCAAATACGCAGGTTGAACATCGTTTGCATGATCAATGGCAAAACGGTAAT

CCCCAGCGCTGGCACCTGACGCGAGACGATC

>151 bp product from linear template CP034479.1:1002220-1003227 Salmonella enterica subsp. enterica serovar Typhimurium strain 14028 chromosome, complete genome, base 802 to base 952 (Forward invj - Reverse invj).

CAGCGCTGGGGAAATGACTATTCCGTCAATATTCAGGCGCGGCAAGCAGGGGAGTTTTCG

TTAATACCGTCAAATACGCAGGTTGAACATCGTTTGCATGATCAATGGCAAAACGGTAAT

CCCCAGCGCTGGCACCTGACGCGAGACGATC

>151 bp product from linear template CP012681.1:c2989764-2988757 Salmonella enterica subsp. enterica serovar Typhimurium strain 33676 chromosome, complete genome, base 802 to base 952 (Forward invj - Reverse invj).

CAGCGCTGGGGAAATGACTATTCCGTCAATATTCAGGCGCGGCAAGCAGGGGAGTTTTCG

TTAATACCGTCAAATACGCAGGTTGAACATCGTTTGCATGATCAATGGCAAAACGGTAAT

CCCCAGCGCTGGCACCTGACGCGAGACGATC

>151 bp product from linear template CP074604.1:1037084-1038091 Salmonella enterica subsp. enterica serovar Typhimurium strain CFSAN000982 chromosome, complete genome, base 802 to base 952 (Forward invj - Reverse invj).

CAGCGCTGGGGAAATGACTATTCCGTCAATATTCAGGCGCGGCAAGCAGGGGAGTTTTCG

TTAATACCGTCAAATACGCAGGTTGAACATCGTTTGCATGATCAATGGCAAAACGGTAAT

CCCCAGCGCTGGCACCTGACGCGAGACGATC

>151 bp product from linear template CP149332.1:1008101-1009108 Salmonella enterica subsp. enterica serovar Typhimurium strain Z1323SSL0011 chromosome, complete genome, base 802 to base 952 (Forward invj - Reverse invj).

CAGCGCTGGGGAAATGACTATTCCGTCAATATTCAGGCGCGGCAAGCAGGGGAGTTTTCG

TTAATACCGTCAAATACGCAGGTTGAACATCGTTTGCATGATCAATGGCAAAACGGTAAT

CCCCAGCGCTGGCACCTGACGCGAGACGATC

>151 bp product from linear template CP050745.1:1087992-1088999 Salmonella enterica subsp. enterica serovar Typhimurium strain ST53 chromosome, complete genome, base 802 to base 952 (Forward invj - Reverse invj).

CAGCGCTGGGGAAATGACTATTCCGTCAATATTCAGGCGCGGCAAGCAGGGGAGTTTTCG

TTAATACCGTCAAATACGCAGGTTGAACATCGTTTGCATGATCAATGGCAAAACGGTAAT

CCCCAGCGCTGGCACCTGACGCGAGACGATC

>151 bp product from linear template CP026700.1:c740651-739644 Salmonella enterica subsp. enterica serovar Typhimurium strain AR_0031 chromosome, complete genome, base 802 to base 952 (Forward invj - Reverse invj).

CAGCGCTGGGGAAATGACTATTCCGTCAATATTCAGGCGCGGCAAGCAGGGGAGTTTTCG

TTAATACCGTCAAATACGCAGGTTGAACATCGTTTGCATGATCAATGGCAAAACGGTAAT

CCCCAGCGCTGGCACCTGACGCGAGACGATC

>151 bp product from linear template CP014051.2:2621074-2622081 Salmonella enterica strain LT2 chromosome, complete genome, base 802 to base 952 (Forward invj - Reverse invj).

CAGCGCTGGGGAAATGACTATTCCGTCAATATTCAGGCGCGGCAAGCAGGGGAGTTTTCG

TTAATACCGTCAAATACGCAGGTTGAACATCGTTTGCATGATCAATGGCAAAACGGTAAT

CCCCAGCGCTGGCACCTGACGCGAGACGATC

>151 bp product from linear template CP051386.1:1009299-1010306 Salmonella enterica subsp. enterica serovar Typhimurium strain CVM 24350 chromosome, complete genome, base 802 to base 952 (Forward invj - Reverse invj).

CAGCGCTGGGGAAATGACTATTCCGTCAATATTCAGGCGCGGCAAGCAGGGGAGTTTTCG

TTAATACCGTCAAATACGCAGGTTGAACATCGTTTGCATGATCAATGGCAAAACGGTAAT

CCCCAGCGCTGGCACCTGACGCGAGACGATC

>151 bp product from linear template CP082606.1:1009692-1010699 Salmonella enterica subsp. enterica serovar Typhimurium strain CVM N17S620 isolate 17KS07GB10-S2 chromosome, complete genome, base 802 to base 952 (Forward invj - Reverse invj).

CAGCGCTGGGGAAATGACTATTCCGTCAATATTCAGGCGCGGCAAGCAGGGGAGTTTTCG

TTAATACCGTCAAATACGCAGGTTGAACATCGTTTGCATGATCAATGGCAAAACGGTAAT

CCCCAGCGCTGGCACCTGACGCGAGACGATC

>151 bp product from linear template CP039565.1:c3001838-3000831 Salmonella enterica subsp. enterica serovar 1,4,[5],12:i:- strain PNCS014849 chromosome, complete genome, base 802 to base 952 (Forward invj - Reverse invj).

CAGCGCTGGGGAAATGACTATTCCGTCAATATTCAGGCGCGGCAAGCAGGGGAGTTTTCG

TTAATACCGTCAAATACGCAGGTTGAACATCGTTTGCATGATCAATGGCAAAACGGTAAT

CCCCAGCGCTGGCACCTGACGCGAGACGATC

>151 bp product from linear template CP091569.1:1040875-1041882 Salmonella enterica strain 131 chromosome, complete genome, base 802 to base 952 (Forward invj - Reverse invj).

CAGCGCTGGGGAAATGACTATTCCGTCAATATTCAGGCGCGGCAAGCAGGGGAGTTTTCG

TTAATACCGTCAAATACGCAGGTTGAACATCGTTTGCATGATCAATGGCAAAACGGTAAT

CCCCAGCGCTGGCACCTGACGCGAGACGATC

>151 bp product from linear template CP149185.1:1039718-1040725 Salmonella enterica subsp. enterica serovar 4,[5],12:i:- strain Z1323SSL0048 chromosome, complete genome, base 802 to base 952 (Forward invj - Reverse invj).

CAGCGCTGGGGAAATGACTATTCCGTCAATATTCAGGCGCGGCAAGCAGGGGAGTTTTCG

TTAATACCGTCAAATACGCAGGTTGAACATCGTTTGCATGATCAATGGCAAAACGGTAAT

CCCCAGCGCTGGCACCTGACGCGAGACGATC

>151 bp product from linear template CP017723.1:1029769-1030776 Salmonella enterica subsp. enterica serovar Stanleyville str. CFSAN000624 strain SARB61, complete genome, base 802 to base 952 (Forward invj - Reverse invj).

CAGCGCTGGGGAAATGACTATTCCGTCAATATTCAGGCGCGGCAAGCAGGGGAGTTTTCG

TTAATACCGTCAAATACGCAGGTTGAACATCGTTTGCATGATCAATGGCAAAACGGTAAT

CCCCAGCGCTGGCACCTGACGCGAGACGATC

>151 bp product from linear template CP066328.1:1002162-1003169 Salmonella enterica strain UWI-PS 6 isolate CFSAN103852 chromosome, complete genome, base 802 to base 952 (Forward invj - Reverse invj).

CAGCGCTGGGGAAATGACTATTCCGTCAATATTCAGGCGCGGCAAGCAGGGGAGTTTTCG

TTAATACCGTCAAATACGCAGGTTGAACATCGTTTGCATGATCAATGGCAAAACGGTAAT

CCCCAGCGCTGGCACCTGACGCGAGACGATC

>151 bp product from linear template CP149270.1:4278988-4279995 Salmonella enterica subsp. enterica serovar 4,[5],12:i:- strain Z1322HSL0031 chromosome, complete genome, base 802 to base 952 (Forward invj - Reverse invj).

CAGCGCTGGGGAAATGACTATTCCGTCAATATTCAGGCGCGGCAAGCAGGGGAGTTTTCG

TTAATACCGTCAAATACGCAGGTTGAACATCGTTTGCATGATCAATGGCAAAACGGTAAT

CCCCAGCGCTGGCACCTGACGCGAGACGATC

>151 bp product from linear template CP083383.1:1040532-1041539 Salmonella enterica subsp. enterica serovar Saintpaul strain CVM N18S0870 chromosome, complete genome, base 802 to base 952 (Forward invj - Reverse invj).

CAGCGCTGGGGAAATGACTATTCCGTCAATATTCAGGCGCGGCAAGCAGGGGAGTTTTCG

TTAATACCGTCAAATACGCAGGTTGAACATCGTTTGCATGATCAATGGCAAAACGGTAAT

CCCCAGCGCTGGCACCTGACGCGAGACGATC

>151 bp product from linear template CP084194.1:1008092-1009099 Salmonella sp. A39 chromosome, complete genome, base 802 to base 952 (Forward invj - Reverse invj).

CAGCGCTGGGGAAATGACTATTCCGTCAATATTCAGGCGCGGCAAGCAGGGGAGTTTTCG

TTAATACCGTCAAATACGCAGGTTGAACATCGTTTGCATGATCAATGGCAAAACGGTAAT

CCCCAGCGCTGGCACCTGACGCGAGACGATC

>151 bp product from linear template CP119478.1:1079734-1080741 Salmonella enterica subsp. enterica serovar 4,[5],12:i:- strain FSIS21925668 chromosome, complete genome, base 802 to base 952 (Forward invj - Reverse invj).

CAGCGCTGGGGAAATGACTATTCCGTCAATATTCAGGCGCGGCAAGCAGGGGAGTTTTCG

TTAATACCGTCAAATACGCAGGTTGAACATCGTTTGCATGATCAATGGCAAAACGGTAAT

CCCCAGCGCTGGCACCTGACGCGAGACGATC

>151 bp product from linear template CP090535.1:c889349-888342 Salmonella enterica strain 2016089-SE chromosome, complete genome, base 802 to base 952 (Forward invj - Reverse invj).

CAGCGCTGGGGAAATGACTATTCCGTCAATATTCAGGCGCGGCAAGCAGGGGAGTTTTCG

TTAATACCGTCAAATACGCAGGTTGAACATCGTTTGCATGATCAATGGCAAAACGGTAAT

CCCCAGCGCTGGCACCTGACGCGAGACGATC

>151 bp product from linear template CP047553.1:c3956053-3955046 Salmonella enterica subsp. enterica serovar Typhimurium strain SJTUF10231 chromosome, complete genome, base 802 to base 952 (Forward invj - Reverse invj).

CAGCGCTGGGGAAATGACTATTCCGTCAATATTCAGGCGCGGCAAGCAGGGGAGTTTTCG

TTAATACCGTCAAATACGCAGGTTGAACATCGTTTGCATGATCAATGGCAAAACGGTAAT

CCCCAGCGCTGGCACCTGACGCGAGACGATC

>151 bp product from linear template CP040321.1:c3070927-3069920 Salmonella enterica subsp. enterica serovar Typhimurium strain PNCS014879 chromosome, complete genome, base 802 to base 952 (Forward invj - Reverse invj).

CAGCGCTGGGGAAATGACTATTCCGTCAATATTCAGGCGCGGCAAGCAGGGGAGTTTTCG

TTAATACCGTCAAATACGCAGGTTGAACATCGTTTGCATGATCAATGGCAAAACGGTAAT

CCCCAGCGCTGGCACCTGACGCGAGACGATC

>151 bp product from linear template CP012985.1:c3093043-3092036 Salmonella enterica subsp. enterica serovar Typhimurium strain RM9437, complete genome, base 802 to base 952 (Forward invj - Reverse invj).

CAGCGCTGGGGAAATGACTATTCCGTCAATATTCAGGCGCGGCAAGCAGGGGAGTTTTCG

TTAATACCGTCAAATACGCAGGTTGAACATCGTTTGCATGATCAATGGCAAAACGGTAAT

CCCCAGCGCTGGCACCTGACGCGAGACGATC

>151 bp product from linear template CP053051.1:1089659-1090666 Salmonella enterica subsp. enterica serovar Typhimurium strain CVM 28321-a chromosome, complete genome, base 802 to base 952 (Forward invj - Reverse invj).

CAGCGCTGGGGAAATGACTATTCCGTCAATATTCAGGCGCGGCAAGCAGGGGAGTTTTCG

TTAATACCGTCAAATACGCAGGTTGAACATCGTTTGCATGATCAATGGCAAAACGGTAAT

CCCCAGCGCTGGCACCTGACGCGAGACGATC

>151 bp product from linear template CP037881.1:c3063288-3062281 Salmonella enterica subsp. enterica serovar 4,[5],12:i:- strain PNCS009991 chromosome, complete genome, base 802 to base 952 (Forward invj - Reverse invj).

CAGCGCTGGGGAAATGACTATTCCGTCAATATTCAGGCGCGGCAAGCAGGGGAGTTTTCG

TTAATACCGTCAAATACGCAGGTTGAACATCGTTTGCATGATCAATGGCAAAACGGTAAT

CCCCAGCGCTGGCACCTGACGCGAGACGATC

>151 bp product from linear template OU015334.1:1039732-1040739 Salmonella enterica subsp. enterica serovar Typhimurium strain AUSMDU00005124 genome assembly, chromosome: C1, base 802 to base 952 (Forward invj - Reverse invj).

CAGCGCTGGGGAAATGACTATTCCGTCAATATTCAGGCGCGGCAAGCAGGGGAGTTTTCG

TTAATACCGTCAAATACGCAGGTTGAACATCGTTTGCATGATCAATGGCAAAACGGTAAT

CCCCAGCGCTGGCACCTGACGCGAGACGATC

>151 bp product from linear template CP117357.1:1037329-1038336 Salmonella enterica subsp. enterica serovar Typhimurium strain RM096 chromosome, complete genome, base 802 to base 952 (Forward invj - Reverse invj).

CAGCGCTGGGGAAATGACTATTCCGTCAATATTCAGGCGCGGCAAGCAGGGGAGTTTTCG

TTAATACCGTCAAATACGCAGGTTGAACATCGTTTGCATGATCAATGGCAAAACGGTAAT

CCCCAGCGCTGGCACCTGACGCGAGACGATC

>151 bp product from linear template CP014983.1:1047026-1048033 Salmonella enterica subsp. enterica serovar Typhimurium str. CDC 2009K-2059 chromosome, complete genome, base 802 to base 952 (Forward invj - Reverse invj).

CAGCGCTGGGGAAATGACTATTCCGTCAATATTCAGGCGCGGCAAGCAGGGGAGTTTTCG

TTAATACCGTCAAATACGCAGGTTGAACATCGTTTGCATGATCAATGGCAAAACGGTAAT

CCCCAGCGCTGGCACCTGACGCGAGACGATC

>151 bp product from linear template CP068018.1:c3704093-3703086 Salmonella enterica strain 1722 chromosome, complete genome, base 802 to base 952 (Forward invj - Reverse invj).

CAGCGCTGGGGAAATGACTATTCCGTCAATATTCAGGCGCGGCAAGCAGGGGAGTTTTCG

TTAATACCGTCAAATACGCAGGTTGAACATCGTTTGCATGATCAATGGCAAAACGGTAAT

CCCCAGCGCTGGCACCTGACGCGAGACGATC

>151 bp product from linear template AP023311.1:1008055-1009062 Salmonella enterica subsp. enterica serovar 4,[5],12:i:- L-4596 DNA, complete genome, base 802 to base 952 (Forward invj - Reverse invj).

CAGCGCTGGGGAAATGACTATTCCGTCAATATTCAGGCGCGGCAAGCAGGGGAGTTTTCG

TTAATACCGTCAAATACGCAGGTTGAACATCGTTTGCATGATCAATGGCAAAACGGTAAT

CCCCAGCGCTGGCACCTGACGCGAGACGATC

>151 bp product from linear template CP002487.1:c3058832-3057825 Salmonella enterica subsp. enterica serovar Typhimurium str. ST4/74 chromosome, complete genome, base 802 to base 952 (Forward invj - Reverse invj).

CAGCGCTGGGGAAATGACTATTCCGTCAATATTCAGGCGCGGCAAGCAGGGGAGTTTTCG

TTAATACCGTCAAATACGCAGGTTGAACATCGTTTGCATGATCAATGGCAAAACGGTAAT

CCCCAGCGCTGGCACCTGACGCGAGACGATC

>151 bp product from linear template CP101379.1:1039922-1040929 Salmonella enterica strain SC2016290 chromosome, complete genome, base 802 to base 952 (Forward invj - Reverse invj).

CAGCGCTGGGGAAATGACTATTCCGTCAATATTCAGGCGCGGCAAGCAGGGGAGTTTTCG

TTAATACCGTCAAATACGCAGGTTGAACATCGTTTGCATGATCAATGGCAAAACGGTAAT

CCCCAGCGCTGGCACCTGACGCGAGACGATC

>151 bp product from linear template CP007804.2:c2949784-2948777 Salmonella enterica subsp. enterica serovar Typhimurium strain VNP20009, complete genome, base 802 to base 952 (Forward invj - Reverse invj).

CAGCGCTGGGGAAATGACTATTCCGTCAATATTCAGGCGCGGCAAGCAGGGGAGTTTTCG

TTAATACCGTCAAATACGCAGGTTGAACATCGTTTGCATGATCAATGGCAAAACGGTAAT

CCCCAGCGCTGGCACCTGACGCGAGACGATC

>151 bp product from linear template AP019375.1:1039929-1040936 Salmonella enterica subsp. enterica serovar 4,[5],12:i:- L-3841 DNA, complete genome, base 802 to base 952 (Forward invj - Reverse invj).

CAGCGCTGGGGAAATGACTATTCCGTCAATATTCAGGCGCGGCAAGCAGGGGAGTTTTCG

TTAATACCGTCAAATACGCAGGTTGAACATCGTTTGCATGATCAATGGCAAAACGGTAAT

CCCCAGCGCTGGCACCTGACGCGAGACGATC

>151 bp product from linear template AP014565.1:c3170132-3169125 Salmonella enterica subsp. enterica serovar Typhimurium str. L-3553 DNA, complete genome, base 802 to base 952 (Forward invj - Reverse invj).

CAGCGCTGGGGAAATGACTATTCCGTCAATATTCAGGCGCGGCAAGCAGGGGAGTTTTCG

TTAATACCGTCAAATACGCAGGTTGAACATCGTTTGCATGATCAATGGCAAAACGGTAAT

CCCCAGCGCTGGCACCTGACGCGAGACGATC

>151 bp product from linear template CP037874.1:c2970611-2969604 Salmonella enterica subsp. enterica serovar 4,[5],12:i:- strain PNCS014854 chromosome, complete genome, base 802 to base 952 (Forward invj - Reverse invj).

CAGCGCTGGGGAAATGACTATTCCGTCAATATTCAGGCGCGGCAAGCAGGGGAGTTTTCG

TTAATACCGTCAAATACGCAGGTTGAACATCGTTTGCATGATCAATGGCAAAACGGTAAT

CCCCAGCGCTGGCACCTGACGCGAGACGATC

>151 bp product from linear template CP039569.1:c3029122-3028115 Salmonella enterica subsp. enterica serovar 1,4,[5],12:i:- strain PNCS014852 chromosome, complete genome, base 802 to base 952 (Forward invj - Reverse invj).

CAGCGCTGGGGAAATGACTATTCCGTCAATATTCAGGCGCGGCAAGCAGGGGAGTTTTCG

TTAATACCGTCAAATACGCAGGTTGAACATCGTTTGCATGATCAATGGCAAAACGGTAAT

CCCCAGCGCTGGCACCTGACGCGAGACGATC

>151 bp product from linear template CP149266.1:c120224-119217 Salmonella enterica subsp. enterica serovar 4,[5],12:i:- strain Z1322HSL0037 chromosome, complete genome, base 802 to base 952 (Forward invj - Reverse invj).

CAGCGCTGGGGAAATGACTATTCCGTCAATATTCAGGCGCGGCAAGCAGGGGAGTTTTCG

TTAATACCGTCAAATACGCAGGTTGAACATCGTTTGCATGATCAATGGCAAAACGGTAAT

CCCCAGCGCTGGCACCTGACGCGAGACGATC

>151 bp product from linear template CP149312.1:c3493947-3492940 Salmonella enterica subsp. enterica serovar Typhimurium strain Z1323SSL0040 chromosome, complete genome, base 802 to base 952 (Forward invj - Reverse invj).

CAGCGCTGGGGAAATGACTATTCCGTCAATATTCAGGCGCGGCAAGCAGGGGAGTTTTCG

TTAATACCGTCAAATACGCAGGTTGAACATCGTTTGCATGATCAATGGCAAAACGGTAAT

CCCCAGCGCTGGCACCTGACGCGAGACGATC

>151 bp product from linear template CP076086.1:1009621-1010628 Salmonella enterica strain CFSAN057258 chromosome, complete genome, base 802 to base 952 (Forward invj - Reverse invj).

CAGCGCTGGGGAAATGACTATTCCGTCAATATTCAGGCGCGGCAAGCAGGGGAGTTTTCG

TTAATACCGTCAAATACGCAGGTTGAACATCGTTTGCATGATCAATGGCAAAACGGTAAT

CCCCAGCGCTGGCACCTGACGCGAGACGATC

>151 bp product from linear template CP117184.1:c3703983-3702976 Salmonella enterica subsp. enterica strain 123 chromosome, complete genome, base 802 to base 952 (Forward invj - Reverse invj).

CAGCGCTGGGGAAATGACTATTCCGTCAATATTCAGGCGCGGCAAGCAGGGGAGTTTTCG

TTAATACCGTCAAATACGCAGGTTGAACATCGTTTGCATGATCAATGGCAAAACGGTAAT

CCCCAGCGCTGGCACCTGACGCGAGACGATC

>151 bp product from linear template CP100739.1:1006658-1007665 Salmonella enterica subsp. enterica serovar Typhimurium strain R18.0292 chromosome, complete genome, base 802 to base 952 (Forward invj - Reverse invj).

CAGCGCTGGGGAAATGACTATTCCGTCAATATTCAGGCGCGGCAAGCAGGGGAGTTTTCG

TTAATACCGTCAAATACGCAGGTTGAACATCGTTTGCATGATCAATGGCAAAACGGTAAT

CCCCAGCGCTGGCACCTGACGCGAGACGATC

>151 bp product from linear template CP089209.1:1039907-1040914 Salmonella enterica strain S15 chromosome, complete genome, base 802 to base 952 (Forward invj - Reverse invj).

CAGCGCTGGGGAAATGACTATTCCGTCAATATTCAGGCGCGGCAAGCAGGGGAGTTTTCG

TTAATACCGTCAAATACGCAGGTTGAACATCGTTTGCATGATCAATGGCAAAACGGTAAT

CCCCAGCGCTGGCACCTGACGCGAGACGATC

>151 bp product from linear template CP044188.1:2346780-2347787 Salmonella enterica subsp. enterica serovar Albert strain AR-0401 chromosome, complete genome, base 802 to base 952 (Forward invj - Reverse invj).

CAGCGCTGGGGAAATGACTATTCCGTCAATATTCAGGCGCGGCAAGCAGGGGAGTTTTCG

TTAATACCGTCAAATACGCAGGTTGAACATCGTTTGCATGATCAATGGCAAAACGGTAAT

CCCCAGCGCTGGCACCTGACGCGAGACGATC

>151 bp product from linear template CP035915.1:1026003-1027010 Salmonella enterica strain S61394 chromosome, complete genome, base 802 to base 952 (Forward invj - Reverse invj).

CAGCGCTGGGGAAATGACTATTCCGTCAATATTCAGGCGCGGCAAGCAGGGGAGTTTTCG

TTAATACCGTCAAATACGCAGGTTGAACATCGTTTGCATGATCAATGGCAAAACGGTAAT

CCCCAGCGCTGGCACCTGACGCGAGACGATC

>151 bp product from linear template CP123692.1:c3912408-3911401 Salmonella enterica subsp. enterica serovar 4,[5],12:i:- strain FSIS11809860 chromosome, complete genome, base 802 to base 952 (Forward invj - Reverse invj).

CAGCGCTGGGGAAATGACTATTCCGTCAATATTCAGGCGCGGCAAGCAGGGGAGTTTTCG

TTAATACCGTCAAATACGCAGGTTGAACATCGTTTGCATGATCAATGGCAAAACGGTAAT

CCCCAGCGCTGGCACCTGACGCGAGACGATC

>151 bp product from linear template CP019204.1:1005421-1006428 Salmonella enterica subsp. enterica serovar Saintpaul strain CFSAN004173, complete genome, base 802 to base 952 (Forward invj - Reverse invj).

CAGCGCTGGGGAAATGACTATTCCGTCAATATTCAGGCGCGGCAAGCAGGGGAGTTTTCG

TTAATACCGTCAAATACGCAGGTTGAACATCGTTTGCATGATCAATGGCAAAACGGTAAT

CCCCAGCGCTGGCACCTGACGCGAGACGATC

>151 bp product from linear template CP123674.1:1079798-1080805 Salmonella enterica subsp. enterica serovar 4,[5],12:i:- strain FSIS12035116 chromosome, complete genome, base 802 to base 952 (Forward invj - Reverse invj).

CAGCGCTGGGGAAATGACTATTCCGTCAATATTCAGGCGCGGCAAGCAGGGGAGTTTTCG

TTAATACCGTCAAATACGCAGGTTGAACATCGTTTGCATGATCAATGGCAAAACGGTAAT

CCCCAGCGCTGGCACCTGACGCGAGACGATC

>151 bp product from linear template CP022491.1:1647131-1648138 Salmonella enterica subsp. enterica serovar Saintpaul strain SA20031783 chromosome, complete genome, base 802 to base 952 (Forward invj - Reverse invj).

CAGCGCTGGGGAAATGACTATTCCGTCAATATTCAGGCGCGGCAAGCAGGGGAGTTTTCG

TTAATACCGTCAAATACGCAGGTTGAACATCGTTTGCATGATCAATGGCAAAACGGTAAT

CCCCAGCGCTGGCACCTGACGCGAGACGATC

>151 bp product from linear template CP024619.1:1004373-1005380 Salmonella enterica subsp. enterica serovar Typhimurium strain BL10 chromosome, complete genome, base 802 to base 952 (Forward invj - Reverse invj).

CAGCGCTGGGGAAATGACTATTCCGTCAATATTCAGGCGCGGCAAGCAGGGGAGTTTTCG

TTAATACCGTCAAATACGCAGGTTGAACATCGTTTGCATGATCAATGGCAAAACGGTAAT

CCCCAGCGCTGGCACCTGACGCGAGACGATC

>151 bp product from linear template CP029567.1:3863188-3864195 Salmonella enterica subsp. enterica serovar Typhimurium strain DA34821 chromosome, complete genome, base 802 to base 952 (Forward invj - Reverse invj).

CAGCGCTGGGGAAATGACTATTCCGTCAATATTCAGGCGCGGCAAGCAGGGGAGTTTTCG

TTAATACCGTCAAATACGCAGGTTGAACATCGTTTGCATGATCAATGGCAAAACGGTAAT

CCCCAGCGCTGGCACCTGACGCGAGACGATC

>151 bp product from linear template CP149187.1:1039755-1040762 Salmonella enterica subsp. enterica serovar 4,[5],12:i:- strain Z1323SSL0020 chromosome, complete genome, base 802 to base 952 (Forward invj - Reverse invj).

CAGCGCTGGGGAAATGACTATTCCGTCAATATTCAGGCGCGGCAAGCAGGGGAGTTTTCG

TTAATACCGTCAAATACGCAGGTTGAACATCGTTTGCATGATCAATGGCAAAACGGTAAT

CCCCAGCGCTGGCACCTGACGCGAGACGATC

>151 bp product from linear template CP160150.1:1002121-1003128 Salmonella enterica subsp. enterica strain N23-2537 chromosome, complete genome, base 802 to base 952 (Forward invj - Reverse invj).

CAGCGCTGGGGAAATGACTATTCCGTCAATATTCAGGCGCGGCAAGCAGGGGAGTTTTCG

TTAATACCGTCAAATACGCAGGTTGAACATCGTTTGCATGATCAATGGCAAAACGGTAAT

CCCCAGCGCTGGCACCTGACGCGAGACGATC

>151 bp product from linear template CP149294.1:1008607-1009614 Salmonella enterica subsp. enterica serovar Typhimurium strain Z1323SSL0058 chromosome, complete genome, base 802 to base 952 (Forward invj - Reverse invj).

CAGCGCTGGGGAAATGACTATTCCGTCAATATTCAGGCGCGGCAAGCAGGGGAGTTTTCG

TTAATACCGTCAAATACGCAGGTTGAACATCGTTTGCATGATCAATGGCAAAACGGTAAT

CCCCAGCGCTGGCACCTGACGCGAGACGATC

>151 bp product from linear template CP094332.1:1007959-1008966 Salmonella enterica subsp. enterica strain 3018683606 chromosome, complete genome, base 802 to base 952 (Forward invj - Reverse invj).

CAGCGCTGGGGAAATGACTATTCCGTCAATATTCAGGCGCGGCAAGCAGGGGAGTTTTCG

TTAATACCGTCAAATACGCAGGTTGAACATCGTTTGCATGATCAATGGCAAAACGGTAAT

CCCCAGCGCTGGCACCTGACGCGAGACGATC

>151 bp product from linear template CP090533.1:c3701710-3700703 Salmonella enterica strain 2017005-SE chromosome, complete genome, base 802 to base 952 (Forward invj - Reverse invj).

CAGCGCTGGGGAAATGACTATTCCGTCAATATTCAGGCGCGGCAAGCAGGGGAGTTTTCG

TTAATACCGTCAAATACGCAGGTTGAACATCGTTTGCATGATCAATGGCAAAACGGTAAT

CCCCAGCGCTGGCACCTGACGCGAGACGATC

>151 bp product from linear template CP027410.1:c3487777-3486770 Salmonella enterica subsp. enterica serovar Typhimurium strain FDAARGOS_317 chromosome, complete genome, base 802 to base 952 (Forward invj - Reverse invj).

CAGCGCTGGGGAAATGACTATTCCGTCAATATTCAGGCGCGGCAAGCAGGGGAGTTTTCG

TTAATACCGTCAAATACGCAGGTTGAACATCGTTTGCATGATCAATGGCAAAACGGTAAT

CCCCAGCGCTGGCACCTGACGCGAGACGATC

>151 bp product from linear template CP011233.1:c2997372-2996365 Salmonella enterica subsp. enterica serovar Typhimurium strain SL1344RX genome, base 802 to base 952 (Forward invj - Reverse invj).

CAGCGCTGGGGAAATGACTATTCCGTCAATATTCAGGCGCGGCAAGCAGGGGAGTTTTCG

TTAATACCGTCAAATACGCAGGTTGAACATCGTTTGCATGATCAATGGCAAAACGGTAAT

CCCCAGCGCTGGCACCTGACGCGAGACGATC

>151 bp product from linear template CP101388.1:1040627-1041634 Salmonella enterica strain SC2017167 chromosome, complete genome, base 802 to base 952 (Forward invj - Reverse invj).

CAGCGCTGGGGAAATGACTATTCCGTCAATATTCAGGCGCGGCAAGCAGGGGAGTTTTCG

TTAATACCGTCAAATACGCAGGTTGAACATCGTTTGCATGATCAATGGCAAAACGGTAAT

CCCCAGCGCTGGCACCTGACGCGAGACGATC

>151 bp product from linear template CP082739.1:1007816-1008823 Salmonella enterica subsp. enterica serovar Typhimurium var. 5- strain CVM N16S070 chromosome, complete genome, base 802 to base 952 (Forward invj - Reverse invj).

CAGCGCTGGGGAAATGACTATTCCGTCAATATTCAGGCGCGGCAAGCAGGGGAGTTTTCG

TTAATACCGTCAAATACGCAGGTTGAACATCGTTTGCATGATCAATGGCAAAACGGTAAT

CCCCAGCGCTGGCACCTGACGCGAGACGATC

>151 bp product from linear template CP051380.1:1076029-1077036 Salmonella enterica subsp. enterica serovar Typhimurium strain CVM 24362 chromosome, complete genome, base 802 to base 952 (Forward invj - Reverse invj).

CAGCGCTGGGGAAATGACTATTCCGTCAATATTCAGGCGCGGCAAGCAGGGGAGTTTTCG

TTAATACCGTCAAATACGCAGGTTGAACATCGTTTGCATGATCAATGGCAAAACGGTAAT

CCCCAGCGCTGGCACCTGACGCGAGACGATC

>151 bp product from linear template CP098741.1:c1249924-1248917 Salmonella enterica subsp. enterica serovar Typhimurium strain HJL222 chromosome, complete genome, base 802 to base 952 (Forward invj - Reverse invj).

CAGCGCTGGGGAAATGACTATTCCGTCAATATTCAGGCGCGGCAAGCAGGGGAGTTTTCG

TTAATACCGTCAAATACGCAGGTTGAACATCGTTTGCATGATCAATGGCAAAACGGTAAT

CCCCAGCGCTGGCACCTGACGCGAGACGATC

>151 bp product from linear template CP019206.1:1005421-1006428 Salmonella enterica subsp. enterica serovar Saintpaul strain CFSAN004174, complete genome, base 802 to base 952 (Forward invj - Reverse invj).

CAGCGCTGGGGAAATGACTATTCCGTCAATATTCAGGCGCGGCAAGCAGGGGAGTTTTCG

TTAATACCGTCAAATACGCAGGTTGAACATCGTTTGCATGATCAATGGCAAAACGGTAAT

CCCCAGCGCTGGCACCTGACGCGAGACGATC

>151 bp product from linear template CP133210.1:1007765-1008772 Salmonella enterica strain ZZC1 chromosome, complete genome, base 802 to base 952 (Forward invj - Reverse invj).

CAGCGCTGGGGAAATGACTATTCCGTCAATATTCAGGCGCGGCAAGCAGGGGAGTTTTCG

TTAATACCGTCAAATACGCAGGTTGAACATCGTTTGCATGATCAATGGCAAAACGGTAAT

CCCCAGCGCTGGCACCTGACGCGAGACGATC

>151 bp product from linear template CP037882.1:c3024589-3023582 Salmonella enterica subsp. enterica serovar 4,[5],12:i:- strain PNCS014875 chromosome, complete genome, base 802 to base 952 (Forward invj - Reverse invj).

CAGCGCTGGGGAAATGACTATTCCGTCAATATTCAGGCGCGGCAAGCAGGGGAGTTTTCG

TTAATACCGTCAAATACGCAGGTTGAACATCGTTTGCATGATCAATGGCAAAACGGTAAT

CCCCAGCGCTGGCACCTGACGCGAGACGATC

>151 bp product from linear template CP074092.1:c3036263-3035256 Salmonella enterica subsp. enterica serovar Typhimurium strain SO_8752_Stm chromosome, complete genome, base 802 to base 952 (Forward invj - Reverse invj).

CAGCGCTGGGGAAATGACTATTCCGTCAATATTCAGGCGCGGCAAGCAGGGGAGTTTTCG

TTAATACCGTCAAATACGCAGGTTGAACATCGTTTGCATGATCAATGGCAAAACGGTAAT

CCCCAGCGCTGGCACCTGACGCGAGACGATC

>151 bp product from linear template CP149339.1:1008813-1009820 Salmonella enterica subsp. enterica serovar Typhimurium strain Z1323SSL0002 chromosome, complete genome, base 802 to base 952 (Forward invj - Reverse invj).

CAGCGCTGGGGAAATGACTATTCCGTCAATATTCAGGCGCGGCAAGCAGGGGAGTTTTCG

TTAATACCGTCAAATACGCAGGTTGAACATCGTTTGCATGATCAATGGCAAAACGGTAAT

CCCCAGCGCTGGCACCTGACGCGAGACGATC

>151 bp product from linear template CP149233.1:1120611-1121618 Salmonella enterica subsp. enterica serovar 4,[5],12:i:- strain Z1323HSL0009 chromosome, complete genome, base 802 to base 952 (Forward invj - Reverse invj).

CAGCGCTGGGGAAATGACTATTCCGTCAATATTCAGGCGCGGCAAGCAGGGGAGTTTTCG

TTAATACCGTCAAATACGCAGGTTGAACATCGTTTGCATGATCAATGGCAAAACGGTAAT

CCCCAGCGCTGGCACCTGACGCGAGACGATC

>151 bp product from linear template CP081187.1:1039716-1040723 Salmonella enterica strain sg1722-1 chromosome, complete genome, base 802 to base 952 (Forward invj - Reverse invj).

CAGCGCTGGGGAAATGACTATTCCGTCAATATTCAGGCGCGGCAAGCAGGGGAGTTTTCG

TTAATACCGTCAAATACGCAGGTTGAACATCGTTTGCATGATCAATGGCAAAACGGTAAT

CCCCAGCGCTGGCACCTGACGCGAGACGATC

>151 bp product from linear template CP019649.1:c3026612-3025605 Salmonella enterica subsp. enterica serovar Typhimurium var. monophasic 4,5,12:i:- strain TW-Stm6 chromosome, complete genome, base 802 to base 952 (Forward invj - Reverse invj).

CAGCGCTGGGGAAATGACTATTCCGTCAATATTCAGGCGCGGCAAGCAGGGGAGTTTTCG

TTAATACCGTCAAATACGCAGGTTGAACATCGTTTGCATGATCAATGGCAAAACGGTAAT

CCCCAGCGCTGGCACCTGACGCGAGACGATC

>151 bp product from linear template CP074297.1:1005443-1006450 Salmonella enterica subsp. enterica serovar Saintpaul strain CFSAN006166 chromosome, complete genome, base 802 to base 952 (Forward invj - Reverse invj).

CAGCGCTGGGGAAATGACTATTCCGTCAATATTCAGGCGCGGCAAGCAGGGGAGTTTTCG

TTAATACCGTCAAATACGCAGGTTGAACATCGTTTGCATGATCAATGGCAAAACGGTAAT

CCCCAGCGCTGGCACCTGACGCGAGACGATC

>151 bp product from linear template CP040318.1:c3084600-3083593 Salmonella enterica subsp. enterica serovar Rough O:-:- strain PNCS009887 chromosome, complete genome, base 802 to base 952 (Forward invj - Reverse invj).

CAGCGCTGGGGAAATGACTATTCCGTCAATATTCAGGCGCGGCAAGCAGGGGAGTTTTCG

TTAATACCGTCAAATACGCAGGTTGAACATCGTTTGCATGATCAATGGCAAAACGGTAAT

CCCCAGCGCTGGCACCTGACGCGAGACGATC

>151 bp product from linear template CP003836.1:c3033906-3032899 Salmonella enterica subsp. enterica serovar Typhimurium str. U288, complete genome, base 802 to base 952 (Forward invj - Reverse invj).

CAGCGCTGGGGAAATGACTATTCCGTCAATATTCAGGCGCGGCAAGCAGGGGAGTTTTCG

TTAATACCGTCAAATACGCAGGTTGAACATCGTTTGCATGATCAATGGCAAAACGGTAAT

CCCCAGCGCTGGCACCTGACGCGAGACGATC

>151 bp product from linear template CP025555.1:1001871-1002878 Salmonella enterica subsp. enterica serovar Typhimurium strain PIR00538 chromosome, complete genome, base 802 to base 952 (Forward invj - Reverse invj).

CAGCGCTGGGGAAATGACTATTCCGTCAATATTCAGGCGCGGCAAGCAGGGGAGTTTTCG

TTAATACCGTCAAATACGCAGGTTGAACATCGTTTGCATGATCAATGGCAAAACGGTAAT

CCCCAGCGCTGGCACCTGACGCGAGACGATC

>151 bp product from linear template CP078535.1:c2939977-2938970 Salmonella enterica strain Colony628 chromosome, base 802 to base 952 (Forward invj - Reverse invj).

CAGCGCTGGGGAAATGACTATTCCGTCAATATTCAGGCGCGGCAAGCAGGGGAGTTTTCG

TTAATACCGTCAAATACGCAGGTTGAACATCGTTTGCATGATCAATGGCAAAACGGTAAT

CCCCAGCGCTGGCACCTGACGCGAGACGATC

>151 bp product from linear template CP149159.1:1039918-1040925 Salmonella enterica subsp. enterica serovar 4,[5],12:i:- strain Z1323SSL0055 chromosome, complete genome, base 802 to base 952 (Forward invj - Reverse invj).

CAGCGCTGGGGAAATGACTATTCCGTCAATATTCAGGCGCGGCAAGCAGGGGAGTTTTCG

TTAATACCGTCAAATACGCAGGTTGAACATCGTTTGCATGATCAATGGCAAAACGGTAAT

CCCCAGCGCTGGCACCTGACGCGAGACGATC

>151 bp product from linear template CP091566.1:c120224-119217 Salmonella enterica strain 137 chromosome, complete genome, base 802 to base 952 (Forward invj - Reverse invj).

CAGCGCTGGGGAAATGACTATTCCGTCAATATTCAGGCGCGGCAAGCAGGGGAGTTTTCG

TTAATACCGTCAAATACGCAGGTTGAACATCGTTTGCATGATCAATGGCAAAACGGTAAT

CCCCAGCGCTGGCACCTGACGCGAGACGATC

>151 bp product from linear template CP149409.1:c3626862-3625855 Salmonella enterica subsp. enterica serovar Typhimurium strain Z1322HSL0019 chromosome, complete genome, base 802 to base 952 (Forward invj - Reverse invj).

CAGCGCTGGGGAAATGACTATTCCGTCAATATTCAGGCGCGGCAAGCAGGGGAGTTTTCG

TTAATACCGTCAAATACGCAGGTTGAACATCGTTTGCATGATCAATGGCAAAACGGTAAT

CCCCAGCGCTGGCACCTGACGCGAGACGATC

>151 bp product from linear template CP049983.1:997289-998296 Salmonella enterica subsp. enterica serovar Saintpaul strain CVM N40391 chromosome, complete genome, base 802 to base 952 (Forward invj - Reverse invj).

CAGCGCTGGGGAAATGACTATTCCGTCAATATTCAGGCGCGGCAAGCAGGGGAGTTTTCG

TTAATACCGTCAAATACGCAGGTTGAACATCGTTTGCATGATCAATGGCAAAACGGTAAT

CCCCAGCGCTGGCACCTGACGCGAGACGATC

>151 bp product from linear template CP123698.1:c3762750-3761743 Salmonella enterica subsp. enterica serovar Typhimurium strain FSIS11815075 chromosome, complete genome, base 802 to base 952 (Forward invj - Reverse invj).

CAGCGCTGGGGAAATGACTATTCCGTCAATATTCAGGCGCGGCAAGCAGGGGAGTTTTCG

TTAATACCGTCAAATACGCAGGTTGAACATCGTTTGCATGATCAATGGCAAAACGGTAAT

CCCCAGCGCTGGCACCTGACGCGAGACGATC

>151 bp product from linear template CP104829.1:1039631-1040638 Salmonella enterica subsp. enterica serovar Typhimurium strain STMC058 chromosome, complete genome, base 802 to base 952 (Forward invj - Reverse invj).

CAGCGCTGGGGAAATGACTATTCCGTCAATATTCAGGCGCGGCAAGCAGGGGAGTTTTCG

TTAATACCGTCAAATACGCAGGTTGAACATCGTTTGCATGATCAATGGCAAAACGGTAAT

CCCCAGCGCTGGCACCTGACGCGAGACGATC

>151 bp product from linear template CP169317.1:1039731-1040738 Salmonella enterica strain SA165 chromosome, complete genome, base 802 to base 952 (Forward invj - Reverse invj).

CAGCGCTGGGGAAATGACTATTCCGTCAATATTCAGGCGCGGCAAGCAGGGGAGTTTTCG

TTAATACCGTCAAATACGCAGGTTGAACATCGTTTGCATGATCAATGGCAAAACGGTAAT

CCCCAGCGCTGGCACCTGACGCGAGACGATC

>151 bp product from linear template CP104820.1:1039627-1040634 Salmonella enterica subsp. enterica serovar Typhimurium strain STMC081 chromosome, complete genome, base 802 to base 952 (Forward invj - Reverse invj).

CAGCGCTGGGGAAATGACTATTCCGTCAATATTCAGGCGCGGCAAGCAGGGGAGTTTTCG

TTAATACCGTCAAATACGCAGGTTGAACATCGTTTGCATGATCAATGGCAAAACGGTAAT

CCCCAGCGCTGGCACCTGACGCGAGACGATC

>151 bp product from linear template CP044967.1:1002070-1003077 Salmonella enterica subsp. enterica serovar 1,4,[5],12:i:- strain PNCS007098 chromosome, complete genome, base 802 to base 952 (Forward invj - Reverse invj).

CAGCGCTGGGGAAATGACTATTCCGTCAATATTCAGGCGCGGCAAGCAGGGGAGTTTTCG

TTAATACCGTCAAATACGCAGGTTGAACATCGTTTGCATGATCAATGGCAAAACGGTAAT

CCCCAGCGCTGGCACCTGACGCGAGACGATC

>151 bp product from linear template CP129209.1:882528-883535 Salmonella enterica subsp. enterica serovar Typhimurium strain HS_180Salm chromosome, complete genome, base 802 to base 952 (Forward invj - Reverse invj).

CAGCGCTGGGGAAATGACTATTCCGTCAATATTCAGGCGCGGCAAGCAGGGGAGTTTTCG

TTAATACCGTCAAATACGCAGGTTGAACATCGTTTGCATGATCAATGGCAAAACGGTAAT

CCCCAGCGCTGGCACCTGACGCGAGACGATC

>151 bp product from linear template CP098438.1:1050535-1051542 Salmonella enterica subsp. enterica serovar Typhimurium strain ATOMSal-L6 chromosome, complete genome, base 802 to base 952 (Forward invj - Reverse invj).

CAGCGCTGGGGAAATGACTATTCCGTCAATATTCAGGCGCGGCAAGCAGGGGAGTTTTCG

TTAATACCGTCAAATACGCAGGTTGAACATCGTTTGCATGATCAATGGCAAAACGGTAAT

CCCCAGCGCTGGCACCTGACGCGAGACGATC

>151 bp product from linear template AP023304.1:1041368-1042375 Salmonella enterica subsp. enterica serovar 4,[5],12:i:- L-4551 DNA, complete genome, base 802 to base 952 (Forward invj - Reverse invj).

CAGCGCTGGGGAAATGACTATTCCGTCAATATTCAGGCGCGGCAAGCAGGGGAGTTTTCG

TTAATACCGTCAAATACGCAGGTTGAACATCGTTTGCATGATCAATGGCAAAACGGTAAT

CCCCAGCGCTGGCACCTGACGCGAGACGATC

>151 bp product from linear template CP043402.1:c3056483-3055476 Salmonella enterica subsp. enterica serovar Typhimurium str. 14028S substr. GXS254 chromosome, complete genome, base 802 to base 952 (Forward invj - Reverse invj).

CAGCGCTGGGGAAATGACTATTCCGTCAATATTCAGGCGCGGCAAGCAGGGGAGTTTTCG

TTAATACCGTCAAATACGCAGGTTGAACATCGTTTGCATGATCAATGGCAAAACGGTAAT

CCCCAGCGCTGGCACCTGACGCGAGACGATC

>151 bp product from linear template CP045954.1:1005902-1006909 Salmonella enterica subsp. enterica serovar Saintpaul strain AUSMDU00010531 chromosome, complete genome, base 802 to base 952 (Forward invj - Reverse invj).

CAGCGCTGGGGAAATGACTATTCCGTCAATATTCAGGCGCGGCAAGCAGGGGAGTTTTCG

TTAATACCGTCAAATACGCAGGTTGAACATCGTTTGCATGATCAATGGCAAAACGGTAAT

CCCCAGCGCTGGCACCTGACGCGAGACGATC

>151 bp product from linear template CP117033.1:1039597-1040604 Salmonella enterica strain PIW95_S14_0299 chromosome, complete genome, base 802 to base 952 (Forward invj - Reverse invj).

CAGCGCTGGGGAAATGACTATTCCGTCAATATTCAGGCGCGGCAAGCAGGGGAGTTTTCG

TTAATACCGTCAAATACGCAGGTTGAACATCGTTTGCATGATCAATGGCAAAACGGTAAT

CCCCAGCGCTGGCACCTGACGCGAGACGATC

>151 bp product from linear template CP149228.1:c3761831-3760824 Salmonella enterica subsp. enterica serovar 4,[5],12:i:- strain Z1323HSL0013 chromosome, complete genome, base 802 to base 952 (Forward invj - Reverse invj).

CAGCGCTGGGGAAATGACTATTCCGTCAATATTCAGGCGCGGCAAGCAGGGGAGTTTTCG

TTAATACCGTCAAATACGCAGGTTGAACATCGTTTGCATGATCAATGGCAAAACGGTAAT

CCCCAGCGCTGGCACCTGACGCGAGACGATC

>151 bp product from linear template CP039607.1:c3025917-3024910 Salmonella enterica subsp. enterica serovar 1,4,[5],12:i:- strain PNCS014873 chromosome, complete genome, base 802 to base 952 (Forward invj - Reverse invj).

CAGCGCTGGGGAAATGACTATTCCGTCAATATTCAGGCGCGGCAAGCAGGGGAGTTTTCG

TTAATACCGTCAAATACGCAGGTTGAACATCGTTTGCATGATCAATGGCAAAACGGTAAT

CCCCAGCGCTGGCACCTGACGCGAGACGATC

>151 bp product from linear template CP044961.1:c3031148-3030141 Salmonella enterica subsp. enterica serovar 1,4,[5],12:i:- strain PNCS014881 chromosome, complete genome, base 802 to base 952 (Forward invj - Reverse invj).

CAGCGCTGGGGAAATGACTATTCCGTCAATATTCAGGCGCGGCAAGCAGGGGAGTTTTCG

TTAATACCGTCAAATACGCAGGTTGAACATCGTTTGCATGATCAATGGCAAAACGGTAAT

CCCCAGCGCTGGCACCTGACGCGAGACGATC

>151 bp product from linear template CP149209.1:4518732-4519739 Salmonella enterica subsp. enterica serovar 4,[5],12:i:- strain Z1323HSL0069 chromosome, complete genome, base 802 to base 952 (Forward invj - Reverse invj).

CAGCGCTGGGGAAATGACTATTCCGTCAATATTCAGGCGCGGCAAGCAGGGGAGTTTTCG

TTAATACCGTCAAATACGCAGGTTGAACATCGTTTGCATGATCAATGGCAAAACGGTAAT

CCCCAGCGCTGGCACCTGACGCGAGACGATC

>151 bp product from linear template CP101390.1:1120612-1121619 Salmonella enterica strain SC2017297 chromosome, complete genome, base 802 to base 952 (Forward invj - Reverse invj).

CAGCGCTGGGGAAATGACTATTCCGTCAATATTCAGGCGCGGCAAGCAGGGGAGTTTTCG

TTAATACCGTCAAATACGCAGGTTGAACATCGTTTGCATGATCAATGGCAAAACGGTAAT

CCCCAGCGCTGGCACCTGACGCGAGACGATC

>151 bp product from linear template AP023300.1:1008055-1009062 Salmonella enterica subsp. enterica serovar 4,[5],12:i:- L-4445 DNA, complete genome, base 802 to base 952 (Forward invj - Reverse invj).

CAGCGCTGGGGAAATGACTATTCCGTCAATATTCAGGCGCGGCAAGCAGGGGAGTTTTCG

TTAATACCGTCAAATACGCAGGTTGAACATCGTTTGCATGATCAATGGCAAAACGGTAAT

CCCCAGCGCTGGCACCTGACGCGAGACGATC

>151 bp product from linear template CP137719.1:c4339463-4338456 Mutant Salmonella enterica isolate FELIX_MS735, complete genome, base 802 to base 952 (Forward invj - Reverse invj).

CAGCGCTGGGGAAATGACTATTCCGTCAATATTCAGGCGCGGCAAGCAGGGGAGTTTTCG

TTAATACCGTCAAATACGCAGGTTGAACATCGTTTGCATGATCAATGGCAAAACGGTAAT

CCCCAGCGCTGGCACCTGACGCGAGACGATC

>151 bp product from linear template CP039595.1:c3043380-3042373 Salmonella enterica subsp. enterica serovar 1,4,[5],12:i:- strain PNCS014865 chromosome, complete genome, base 802 to base 952 (Forward invj - Reverse invj).

CAGCGCTGGGGAAATGACTATTCCGTCAATATTCAGGCGCGGCAAGCAGGGGAGTTTTCG

TTAATACCGTCAAATACGCAGGTTGAACATCGTTTGCATGATCAATGGCAAAACGGTAAT

CCCCAGCGCTGGCACCTGACGCGAGACGATC

>151 bp product from linear template CP045952.1:c1080533-1079526 Salmonella enterica subsp. enterica serovar Typhimurium strain AUSMDU00008979 chromosome, complete genome, base 802 to base 952 (Forward invj - Reverse invj).

CAGCGCTGGGGAAATGACTATTCCGTCAATATTCAGGCGCGGCAAGCAGGGGAGTTTTCG

TTAATACCGTCAAATACGCAGGTTGAACATCGTTTGCATGATCAATGGCAAAACGGTAAT

CCCCAGCGCTGGCACCTGACGCGAGACGATC

>151 bp product from linear template CP099705.1:1037354-1038361 Salmonella enterica subsp. enterica serovar Typhimurium strain 013+ chromosome, complete genome, base 802 to base 952 (Forward invj - Reverse invj).

CAGCGCTGGGGAAATGACTATTCCGTCAATATTCAGGCGCGGCAAGCAGGGGAGTTTTCG

TTAATACCGTCAAATACGCAGGTTGAACATCGTTTGCATGATCAATGGCAAAACGGTAAT

CCCCAGCGCTGGCACCTGACGCGAGACGATC

>151 bp product from linear template CP037879.1:c3006875-3005868 Salmonella enterica subsp. enterica serovar 4,[5],12:i:- strain PNCS014863 chromosome, complete genome, base 802 to base 952 (Forward invj - Reverse invj).

CAGCGCTGGGGAAATGACTATTCCGTCAATATTCAGGCGCGGCAAGCAGGGGAGTTTTCG

TTAATACCGTCAAATACGCAGGTTGAACATCGTTTGCATGATCAATGGCAAAACGGTAAT

CCCCAGCGCTGGCACCTGACGCGAGACGATC

>151 bp product from linear template CP115834.1:1041275-1042282 Salmonella enterica subsp. enterica serovar Typhimurium strain R15.0430 chromosome, complete genome, base 802 to base 952 (Forward invj - Reverse invj).

CAGCGCTGGGGAAATGACTATTCCGTCAATATTCAGGCGCGGCAAGCAGGGGAGTTTTCG

TTAATACCGTCAAATACGCAGGTTGAACATCGTTTGCATGATCAATGGCAAAACGGTAAT

CCCCAGCGCTGGCACCTGACGCGAGACGATC

>151 bp product from linear template CP082429.1:1007296-1008303 Salmonella enterica subsp. enterica serovar Typhimurium strain FSIS1609224 chromosome, complete genome, base 802 to base 952 (Forward invj - Reverse invj).

CAGCGCTGGGGAAATGACTATTCCGTCAATATTCAGGCGCGGCAAGCAGGGGAGTTTTCG

TTAATACCGTCAAATACGCAGGTTGAACATCGTTTGCATGATCAATGGCAAAACGGTAAT

CCCCAGCGCTGGCACCTGACGCGAGACGATC

>151 bp product from linear template CP074279.1:1005527-1006534 Salmonella enterica subsp. enterica serovar Saintpaul strain CFSAN006246 chromosome, complete genome, base 802 to base 952 (Forward invj - Reverse invj).

CAGCGCTGGGGAAATGACTATTCCGTCAATATTCAGGCGCGGCAAGCAGGGGAGTTTTCG

TTAATACCGTCAAATACGCAGGTTGAACATCGTTTGCATGATCAATGGCAAAACGGTAAT

CCCCAGCGCTGGCACCTGACGCGAGACGATC

>151 bp product from linear template CP091544.1:1122972-1123979 Salmonella enterica strain 1618 chromosome, complete genome, base 802 to base 952 (Forward invj - Reverse invj).

CAGCGCTGGGGAAATGACTATTCCGTCAATATTCAGGCGCGGCAAGCAGGGGAGTTTTCG

TTAATACCGTCAAATACGCAGGTTGAACATCGTTTGCATGATCAATGGCAAAACGGTAAT

CCCCAGCGCTGGCACCTGACGCGAGACGATC

>151 bp product from linear template CP046283.1:809972-810979 Salmonella enterica strain FDAARGOS_687 chromosome, complete genome, base 802 to base 952 (Forward invj - Reverse invj).

CAGCGCTGGGGAAATGACTATTCCGTCAATATTCAGGCGCGGCAAGCAGGGGAGTTTTCG

TTAATACCGTCAAATACGCAGGTTGAACATCGTTTGCATGATCAATGGCAAAACGGTAAT

CCCCAGCGCTGGCACCTGACGCGAGACGATC

>151 bp product from linear template CP129630.1:1003043-1004050 Salmonella enterica subsp. enterica serovar Typhimurium strain ST_F0903R chromosome, complete genome, base 802 to base 952 (Forward invj - Reverse invj).

CAGCGCTGGGGAAATGACTATTCCGTCAATATTCAGGCGCGGCAAGCAGGGGAGTTTTCG

TTAATACCGTCAAATACGCAGGTTGAACATCGTTTGCATGATCAATGGCAAAACGGTAAT

CCCCAGCGCTGGCACCTGACGCGAGACGATC

>151 bp product from linear template CP149403.1:1008113-1009120 Salmonella enterica subsp. enterica serovar Typhimurium strain Z1322HSL0036 chromosome, complete genome, base 802 to base 952 (Forward invj - Reverse invj).

CAGCGCTGGGGAAATGACTATTCCGTCAATATTCAGGCGCGGCAAGCAGGGGAGTTTTCG

TTAATACCGTCAAATACGCAGGTTGAACATCGTTTGCATGATCAATGGCAAAACGGTAAT

CCCCAGCGCTGGCACCTGACGCGAGACGATC

>151 bp product from linear template CP104816.1:1039609-1040616 Salmonella enterica subsp. enterica serovar Typhimurium strain STMC125 chromosome, complete genome, base 802 to base 952 (Forward invj - Reverse invj).

CAGCGCTGGGGAAATGACTATTCCGTCAATATTCAGGCGCGGCAAGCAGGGGAGTTTTCG

TTAATACCGTCAAATACGCAGGTTGAACATCGTTTGCATGATCAATGGCAAAACGGTAAT

CCCCAGCGCTGGCACCTGACGCGAGACGATC

>151 bp product from linear template CP053870.1:c3061276-3060269 Salmonella enterica subsp. enterica serovar Typhimurium strain SS2017 chromosome, complete genome, base 802 to base 952 (Forward invj - Reverse invj).

CAGCGCTGGGGAAATGACTATTCCGTCAATATTCAGGCGCGGCAAGCAGGGGAGTTTTCG

TTAATACCGTCAAATACGCAGGTTGAACATCGTTTGCATGATCAATGGCAAAACGGTAAT

CCCCAGCGCTGGCACCTGACGCGAGACGATC

>151 bp product from linear template CP126178.1:c1114750-1113743 Salmonella enterica subsp. enterica serovar 1,4,12:i:- strain BL661 chromosome, complete genome, base 802 to base 952 (Forward invj - Reverse invj).

CAGCGCTGGGGAAATGACTATTCCGTCAATATTCAGGCGCGGCAAGCAGGGGAGTTTTCG

TTAATACCGTCAAATACGCAGGTTGAACATCGTTTGCATGATCAATGGCAAAACGGTAAT

CCCCAGCGCTGGCACCTGACGCGAGACGATC

>151 bp product from linear template CP047548.1:c3949804-3948797 Salmonella enterica subsp. enterica serovar Typhimurium strain SJTUF10169 chromosome, complete genome, base 802 to base 952 (Forward invj - Reverse invj).

CAGCGCTGGGGAAATGACTATTCCGTCAATATTCAGGCGCGGCAAGCAGGGGAGTTTTCG

TTAATACCGTCAAATACGCAGGTTGAACATCGTTTGCATGATCAATGGCAAAACGGTAAT

CCCCAGCGCTGGCACCTGACGCGAGACGATC

>151 bp product from linear template CP149154.1:1039602-1040609 Salmonella enterica subsp. enterica serovar 4,[5],12:i:- strain Z1323SSL0056 chromosome, complete genome, base 802 to base 952 (Forward invj - Reverse invj).

CAGCGCTGGGGAAATGACTATTCCGTCAATATTCAGGCGCGGCAAGCAGGGGAGTTTTCG

TTAATACCGTCAAATACGCAGGTTGAACATCGTTTGCATGATCAATGGCAAAACGGTAAT

CCCCAGCGCTGGCACCTGACGCGAGACGATC

>151 bp product from linear template CP149142.1:1039918-1040925 Salmonella enterica subsp. enterica serovar 4,[5],12:i:- strain Z1323SSL0062 chromosome, complete genome, base 802 to base 952 (Forward invj - Reverse invj).

CAGCGCTGGGGAAATGACTATTCCGTCAATATTCAGGCGCGGCAAGCAGGGGAGTTTTCG

TTAATACCGTCAAATACGCAGGTTGAACATCGTTTGCATGATCAATGGCAAAACGGTAAT

CCCCAGCGCTGGCACCTGACGCGAGACGATC

>151 bp product from linear template CP149237.1:1039755-1040762 Salmonella enterica subsp. enterica serovar 4,[5],12:i:- strain Z1323HSL0005 chromosome, complete genome, base 802 to base 952 (Forward invj - Reverse invj).

CAGCGCTGGGGAAATGACTATTCCGTCAATATTCAGGCGCGGCAAGCAGGGGAGTTTTCG

TTAATACCGTCAAATACGCAGGTTGAACATCGTTTGCATGATCAATGGCAAAACGGTAAT

CCCCAGCGCTGGCACCTGACGCGAGACGATC

>151 bp product from linear template CP091868.1:c3028193-3027186 Salmonella enterica subsp. enterica serovar 4,[5],12:- strain H1-014 chromosome, complete genome, base 802 to base 952 (Forward invj - Reverse invj).

CAGCGCTGGGGAAATGACTATTCCGTCAATATTCAGGCGCGGCAAGCAGGGGAGTTTTCG

TTAATACCGTCAAATACGCAGGTTGAACATCGTTTGCATGATCAATGGCAAAACGGTAAT

CCCCAGCGCTGGCACCTGACGCGAGACGATC

>151 bp product from linear template CP078532.1:1024564-1025571 Salmonella enterica subsp. enterica serovar Typhimurium strain Colony110 chromosome, base 802 to base 952 (Forward invj - Reverse invj).

CAGCGCTGGGGAAATGACTATTCCGTCAATATTCAGGCGCGGCAAGCAGGGGAGTTTTCG

TTAATACCGTCAAATACGCAGGTTGAACATCGTTTGCATGATCAATGGCAAAACGGTAAT

CCCCAGCGCTGGCACCTGACGCGAGACGATC

>151 bp product from linear template CP061050.1:1001549-1002556 Salmonella enterica subsp. enterica serovar Typhimurium strain BBS1358sml chromosome, complete genome, base 802 to base 952 (Forward invj - Reverse invj).

CAGCGCTGGGGAAATGACTATTCCGTCAATATTCAGGCGCGGCAAGCAGGGGAGTTTTCG

TTAATACCGTCAAATACGCAGGTTGAACATCGTTTGCATGATCAATGGCAAAACGGTAAT

CCCCAGCGCTGGCACCTGACGCGAGACGATC

>151 bp product from linear template CP149397.1:c3623593-3622586 Salmonella enterica subsp. enterica serovar Typhimurium strain Z1323CSL0047 chromosome, complete genome, base 802 to base 952 (Forward invj - Reverse invj).

CAGCGCTGGGGAAATGACTATTCCGTCAATATTCAGGCGCGGCAAGCAGGGGAGTTTTCG

TTAATACCGTCAAATACGCAGGTTGAACATCGTTTGCATGATCAATGGCAAAACGGTAAT

CCCCAGCGCTGGCACCTGACGCGAGACGATC

>151 bp product from linear template CP061047.1:1083330-1084337 Salmonella enterica subsp. enterica serovar Typhimurium strain BBS1359 chromosome, complete genome, base 802 to base 952 (Forward invj - Reverse invj).

CAGCGCTGGGGAAATGACTATTCCGTCAATATTCAGGCGCGGCAAGCAGGGGAGTTTTCG

TTAATACCGTCAAATACGCAGGTTGAACATCGTTTGCATGATCAATGGCAAAACGGTAAT

CCCCAGCGCTGGCACCTGACGCGAGACGATC

>151 bp product from linear template CP163542.1:1008775-1009782 Salmonella enterica subsp. enterica serovar Typhimurium strain LT2 chromosome, complete genome, base 802 to base 952 (Forward invj - Reverse invj).

CAGCGCTGGGGAAATGACTATTCCGTCAATATTCAGGCGCGGCAAGCAGGGGAGTTTTCG

TTAATACCGTCAAATACGCAGGTTGAACATCGTTTGCATGATCAATGGCAAAACGGTAAT

CCCCAGCGCTGGCACCTGACGCGAGACGATC

>151 bp product from linear template CP149337.1:1002929-1003936 Salmonella enterica subsp. enterica serovar Typhimurium strain Z1323SSL0009 chromosome, complete genome, base 802 to base 952 (Forward invj - Reverse invj).

CAGCGCTGGGGAAATGACTATTCCGTCAATATTCAGGCGCGGCAAGCAGGGGAGTTTTCG

TTAATACCGTCAAATACGCAGGTTGAACATCGTTTGCATGATCAATGGCAAAACGGTAAT

CCCCAGCGCTGGCACCTGACGCGAGACGATC

>151 bp product from linear template CP060169.1:c3058990-3057983 Salmonella enterica subsp. enterica serovar Typhimurium strain BKQZM9 chromosome, complete genome, base 802 to base 952 (Forward invj - Reverse invj).

CAGCGCTGGGGAAATGACTATTCCGTCAATATTCAGGCGCGGCAAGCAGGGGAGTTTTCG

TTAATACCGTCAAATACGCAGGTTGAACATCGTTTGCATGATCAATGGCAAAACGGTAAT

CCCCAGCGCTGGCACCTGACGCGAGACGATC

>151 bp product from linear template CP113541.1:1036232-1037239 Salmonella enterica strain CHC chromosome, complete genome, base 802 to base 952 (Forward invj - Reverse invj).

CAGCGCTGGGGAAATGACTATTCCGTCAATATTCAGGCGCGGCAAGCAGGGGAGTTTTCG

TTAATACCGTCAAATACGCAGGTTGAACATCGTTTGCATGATCAATGGCAAAACGGTAAT

CCCCAGCGCTGGCACCTGACGCGAGACGATC

>151 bp product from linear template CP060507.1:c3036279-3035272 Salmonella enterica subsp. enterica serovar Typhimurium strain LT2 chromosome, complete genome, base 802 to base 952 (Forward invj - Reverse invj).

CAGCGCTGGGGAAATGACTATTCCGTCAATATTCAGGCGCGGCAAGCAGGGGAGTTTTCG

TTAATACCGTCAAATACGCAGGTTGAACATCGTTTGCATGATCAATGGCAAAACGGTAAT

CCCCAGCGCTGGCACCTGACGCGAGACGATC

>151 bp product from linear template CP018657.1:c1316091-1315084 Salmonella enterica subsp. enterica serovar Enteritidis strain 92-0392 chromosome, complete genome, base 802 to base 952 (Forward invj - Reverse invj).

CAGCGCTGGGGAAATGACTATTCCGTCAATATTCAGGCGCGGCAAGCAGGGGAGTTTTCG

TTAATACCGTCAAATACGCAGGTTGAACATCGTTTGCATGATCAATGGCAAAACGGTAAT

CCCCAGCGCTGGCACCTGACGCGAGACGATC

>151 bp product from linear template CP049981.1:c100678-99671 Salmonella enterica subsp. enterica serovar Saintpaul strain CVM N52030 chromosome, complete genome, base 802 to base 952 (Forward invj - Reverse invj).

CAGCGCTGGGGAAATGACTATTCCGTCAATATTCAGGCGCGGCAAGCAGGGGAGTTTTCG

TTAATACCGTCAAATACGCAGGTTGAACATCGTTTGCATGATCAATGGCAAAACGGTAAT

CCCCAGCGCTGGCACCTGACGCGAGACGATC

>151 bp product from linear template CP082376.1:1039931-1040938 Salmonella enterica subsp. enterica serovar 4,[5],12:i:- strain FSIS1702343 chromosome, complete genome, base 802 to base 952 (Forward invj - Reverse invj).

CAGCGCTGGGGAAATGACTATTCCGTCAATATTCAGGCGCGGCAAGCAGGGGAGTTTTCG

TTAATACCGTCAAATACGCAGGTTGAACATCGTTTGCATGATCAATGGCAAAACGGTAAT

CCCCAGCGCTGGCACCTGACGCGAGACGATC

>151 bp product from linear template CP061049.1:1083330-1084337 Salmonella enterica subsp. enterica serovar Typhimurium strain BBS1358lrg chromosome, complete genome, base 802 to base 952 (Forward invj - Reverse invj).

CAGCGCTGGGGAAATGACTATTCCGTCAATATTCAGGCGCGGCAAGCAGGGGAGTTTTCG

TTAATACCGTCAAATACGCAGGTTGAACATCGTTTGCATGATCAATGGCAAAACGGTAAT

CCCCAGCGCTGGCACCTGACGCGAGACGATC

>151 bp product from linear template CP098834.1:1040058-1041065 Salmonella enterica subsp. enterica serovar Typhimurium strain GD19PS1 chromosome, complete genome, base 802 to base 952 (Forward invj - Reverse invj).

CAGCGCTGGGGAAATGACTATTCCGTCAATATTCAGGCGCGGCAAGCAGGGGAGTTTTCG

TTAATACCGTCAAATACGCAGGTTGAACATCGTTTGCATGATCAATGGCAAAACGGTAAT

CCCCAGCGCTGGCACCTGACGCGAGACGATC

>151 bp product from linear template AP023289.1:1120611-1121618 Salmonella enterica subsp. enterica serovar 4,[5],12:i:- L-3837 DNA, complete genome, base 802 to base 952 (Forward invj - Reverse invj).

CAGCGCTGGGGAAATGACTATTCCGTCAATATTCAGGCGCGGCAAGCAGGGGAGTTTTCG

TTAATACCGTCAAATACGCAGGTTGAACATCGTTTGCATGATCAATGGCAAAACGGTAAT

CCCCAGCGCTGGCACCTGACGCGAGACGATC

>151 bp product from linear template CP037877.1:c2972572-2971565 Salmonella enterica subsp. enterica serovar 4,[5],12:i:- strain PNCS015054 chromosome, complete genome, base 802 to base 952 (Forward invj - Reverse invj).

CAGCGCTGGGGAAATGACTATTCCGTCAATATTCAGGCGCGGCAAGCAGGGGAGTTTTCG

TTAATACCGTCAAATACGCAGGTTGAACATCGTTTGCATGATCAATGGCAAAACGGTAAT

CCCCAGCGCTGGCACCTGACGCGAGACGATC

>151 bp product from linear template CP044957.1:c2973122-2972115 Salmonella enterica subsp. enterica serovar 1,4,[5],12:i:- strain PNCS007087 chromosome, complete genome, base 802 to base 952 (Forward invj - Reverse invj).

CAGCGCTGGGGAAATGACTATTCCGTCAATATTCAGGCGCGGCAAGCAGGGGAGTTTTCG

TTAATACCGTCAAATACGCAGGTTGAACATCGTTTGCATGATCAATGGCAAAACGGTAAT

CCCCAGCGCTGGCACCTGACGCGAGACGATC

>151 bp product from linear template CP051273.1:991311-992318 Salmonella enterica subsp. enterica serovar Worthington strain OLF-FSR1_WB_Partridge_SW-37 chromosome, complete genome, base 802 to base 952 (Forward invj - Reverse invj).

CAGCGCTGGGGAAATGACTATTCCGTCAATATTCAGGCGCGGCAAGCAGGGGAGTTTTCG

TTAATACCGTCAAATACGCAGGTTGAACATCGTTTGCATGATCAATGGCAAAACGGTAAT

CCCCAGCGCTGGCACCTGACGCGAGACGATC

>151 bp product from linear template CP065066.1:1039624-1040631 Salmonella enterica subsp. enterica strain 1-E6 chromosome, complete genome, base 802 to base 952 (Forward invj - Reverse invj).

CAGCGCTGGGGAAATGACTATTCCGTCAATATTCAGGCGCGGCAAGCAGGGGAGTTTTCG

TTAATACCGTCAAATACGCAGGTTGAACATCGTTTGCATGATCAATGGCAAAACGGTAAT

CCCCAGCGCTGGCACCTGACGCGAGACGATC

>151 bp product from linear template CP133183.1:1039718-1040725 Salmonella enterica subsp. enterica strain Z1323SSL0063 chromosome, complete genome, base 802 to base 952 (Forward invj - Reverse invj).

CAGCGCTGGGGAAATGACTATTCCGTCAATATTCAGGCGCGGCAAGCAGGGGAGTTTTCG

TTAATACCGTCAAATACGCAGGTTGAACATCGTTTGCATGATCAATGGCAAAACGGTAAT

CCCCAGCGCTGGCACCTGACGCGAGACGATC

>151 bp product from linear template CP078531.1:1024564-1025571 Salmonella enterica subsp. enterica serovar Typhimurium strain Colony566 chromosome, base 802 to base 952 (Forward invj - Reverse invj).

CAGCGCTGGGGAAATGACTATTCCGTCAATATTCAGGCGCGGCAAGCAGGGGAGTTTTCG

TTAATACCGTCAAATACGCAGGTTGAACATCGTTTGCATGATCAATGGCAAAACGGTAAT

CCCCAGCGCTGGCACCTGACGCGAGACGATC

>151 bp product from linear template CP123680.1:c3919179-3918172 Salmonella enterica subsp. enterica serovar 4,[5],12:i:- strain FSIS11922707 chromosome, complete genome, base 802 to base 952 (Forward invj - Reverse invj).

CAGCGCTGGGGAAATGACTATTCCGTCAATATTCAGGCGCGGCAAGCAGGGGAGTTTTCG

TTAATACCGTCAAATACGCAGGTTGAACATCGTTTGCATGATCAATGGCAAAACGGTAAT

CCCCAGCGCTGGCACCTGACGCGAGACGATC

>151 bp product from linear template CP082475.1:1039528-1040535 Salmonella enterica subsp. enterica serovar 4,[5],12:i:- strain FSIS1607294 chromosome, complete genome, base 802 to base 952 (Forward invj - Reverse invj).

CAGCGCTGGGGAAATGACTATTCCGTCAATATTCAGGCGCGGCAAGCAGGGGAGTTTTCG

TTAATACCGTCAAATACGCAGGTTGAACATCGTTTGCATGATCAATGGCAAAACGGTAAT

CCCCAGCGCTGGCACCTGACGCGAGACGATC

>151 bp product from linear template CP078533.1:1024564-1025571 Salmonella enterica subsp. enterica serovar Typhimurium strain Colony560 chromosome, base 802 to base 952 (Forward invj - Reverse invj).

CAGCGCTGGGGAAATGACTATTCCGTCAATATTCAGGCGCGGCAAGCAGGGGAGTTTTCG

TTAATACCGTCAAATACGCAGGTTGAACATCGTTTGCATGATCAATGGCAAAACGGTAAT

CCCCAGCGCTGGCACCTGACGCGAGACGATC

>151 bp product from linear template AP023291.1:1007939-1008946 Salmonella enterica subsp. enterica serovar Typhimurium L-4126 DNA, complete genome, base 802 to base 952 (Forward invj - Reverse invj).

CAGCGCTGGGGAAATGACTATTCCGTCAATATTCAGGCGCGGCAAGCAGGGGAGTTTTCG

TTAATACCGTCAAATACGCAGGTTGAACATCGTTTGCATGATCAATGGCAAAACGGTAAT

CCCCAGCGCTGGCACCTGACGCGAGACGATC

>151 bp product from linear template CP091540.1:1007583-1008590 Salmonella enterica subsp. enterica serovar Typhimurium strain S46L1 chromosome, complete genome, base 802 to base 952 (Forward invj - Reverse invj).

CAGCGCTGGGGAAATGACTATTCCGTCAATATTCAGGCGCGGCAAGCAGGGGAGTTTTCG

TTAATACCGTCAAATACGCAGGTTGAACATCGTTTGCATGATCAATGGCAAAACGGTAAT

CCCCAGCGCTGGCACCTGACGCGAGACGATC

>151 bp product from linear template CP082596.1:1080191-1081198 Salmonella enterica subsp. enterica serovar Typhimurium strain CVM N18S0666 chromosome, complete genome, base 802 to base 952 (Forward invj - Reverse invj).

CAGCGCTGGGGAAATGACTATTCCGTCAATATTCAGGCGCGGCAAGCAGGGGAGTTTTCG

TTAATACCGTCAAATACGCAGGTTGAACATCGTTTGCATGATCAATGGCAAAACGGTAAT

CCCCAGCGCTGGCACCTGACGCGAGACGATC

>151 bp product from linear template CP011428.1:c3065315-3064308 Salmonella enterica subsp. enterica strain YU39, complete genome, base 802 to base 952 (Forward invj - Reverse invj).

CAGCGCTGGGGAAATGACTATTCCGTCAATATTCAGGCGCGGCAAGCAGGGGAGTTTTCG

TTAATACCGTCAAATACGCAGGTTGAACATCGTTTGCATGATCAATGGCAAAACGGTAAT

CCCCAGCGCTGGCACCTGACGCGAGACGATC

>151 bp product from linear template CP040900.1:1009580-1010587 Salmonella enterica subsp. enterica serovar Typhimurium strain SAP18-6199 chromosome, complete genome, base 802 to base 952 (Forward invj - Reverse invj).

CAGCGCTGGGGAAATGACTATTCCGTCAATATTCAGGCGCGGCAAGCAGGGGAGTTTTCG

TTAATACCGTCAAATACGCAGGTTGAACATCGTTTGCATGATCAATGGCAAAACGGTAAT

CCCCAGCGCTGGCACCTGACGCGAGACGATC

>151 bp product from linear template CP025736.1:c3856671-3855664 Salmonella enterica subsp. enterica serovar Typhimurium strain FORC_079 chromosome, complete genome, base 802 to base 952 (Forward invj - Reverse invj).

CAGCGCTGGGGAAATGACTATTCCGTCAATATTCAGGCGCGGCAAGCAGGGGAGTTTTCG

TTAATACCGTCAAATACGCAGGTTGAACATCGTTTGCATGATCAATGGCAAAACGGTAAT

CCCCAGCGCTGGCACCTGACGCGAGACGATC

>151 bp product from linear template CP074673.1:1002212-1003219 Salmonella enterica subsp. enterica strain CFSAN002003 chromosome, complete genome, base 802 to base 952 (Forward invj - Reverse invj).

CAGCGCTGGGGAAATGACTATTCCGTCAATATTCAGGCGCGGCAAGCAGGGGAGTTTTCG

TTAATACCGTCAAATACGCAGGTTGAACATCGTTTGCATGATCAATGGCAAAACGGTAAT

CCCCAGCGCTGGCACCTGACGCGAGACGATC

>151 bp product from linear template CP082553.1:c3761634-3760627 Salmonella enterica subsp. enterica serovar Typhimurium strain CVM N18S1634 chromosome, complete genome, base 802 to base 952 (Forward invj - Reverse invj).

CAGCGCTGGGGAAATGACTATTCCGTCAATATTCAGGCGCGGCAAGCAGGGGAGTTTTCG

TTAATACCGTCAAATACGCAGGTTGAACATCGTTTGCATGATCAATGGCAAAACGGTAAT

CCCCAGCGCTGGCACCTGACGCGAGACGATC

>151 bp product from linear template CP040564.1:1026957-1027964 Salmonella enterica subsp. enterica serovar Typhimurium strain SAP17-7699 chromosome, complete genome, base 802 to base 952 (Forward invj - Reverse invj).

CAGCGCTGGGGAAATGACTATTCCGTCAATATTCAGGCGCGGCAAGCAGGGGAGTTTTCG

TTAATACCGTCAAATACGCAGGTTGAACATCGTTTGCATGATCAATGGCAAAACGGTAAT

CCCCAGCGCTGGCACCTGACGCGAGACGATC

>151 bp product from linear template CP039561.1:c3043865-3042858 Salmonella enterica subsp. enterica serovar 1,4,[5],12:i:- strain PNCS014847 chromosome, complete genome, base 802 to base 952 (Forward invj - Reverse invj).

CAGCGCTGGGGAAATGACTATTCCGTCAATATTCAGGCGCGGCAAGCAGGGGAGTTTTCG

TTAATACCGTCAAATACGCAGGTTGAACATCGTTTGCATGATCAATGGCAAAACGGTAAT

CCCCAGCGCTGGCACCTGACGCGAGACGATC

>151 bp product from linear template CP050739.1:1136554-1137561 Salmonella enterica subsp. enterica serovar Typhimurium strain ST56 chromosome, complete genome, base 802 to base 952 (Forward invj - Reverse invj).

CAGCGCTGGGGAAATGACTATTCCGTCAATATTCAGGCGCGGCAAGCAGGGGAGTTTTCG

TTAATACCGTCAAATACGCAGGTTGAACATCGTTTGCATGATCAATGGCAAAACGGTAAT

CCCCAGCGCTGGCACCTGACGCGAGACGATC

>151 bp product from linear template CP085696.1:1007377-1008384 Salmonella enterica subsp. enterica serovar Saintpaul strain S25 chromosome, complete genome, base 802 to base 952 (Forward invj - Reverse invj).

CAGCGCTGGGGAAATGACTATTCCGTCAATATTCAGGCGCGGCAAGCAGGGGAGTTTTCG

TTAATACCGTCAAATACGCAGGTTGAACATCGTTTGCATGATCAATGGCAAAACGGTAAT

CCCCAGCGCTGGCACCTGACGCGAGACGATC

>151 bp product from linear template CP149262.1:1120429-1121436 Salmonella enterica subsp. enterica serovar 4,[5],12:i:- strain Z1322HSL0038 chromosome, complete genome, base 802 to base 952 (Forward invj - Reverse invj).

CAGCGCTGGGGAAATGACTATTCCGTCAATATTCAGGCGCGGCAAGCAGGGGAGTTTTCG

TTAATACCGTCAAATACGCAGGTTGAACATCGTTTGCATGATCAATGGCAAAACGGTAAT

CCCCAGCGCTGGCACCTGACGCGAGACGATC

>151 bp product from linear template CP032490.1:c3017031-3016024 Salmonella enterica subsp. enterica serovar Typhimurium strain SL26 chromosome, complete genome, base 802 to base 952 (Forward invj - Reverse invj).

CAGCGCTGGGGAAATGACTATTCCGTCAATATTCAGGCGCGGCAAGCAGGGGAGTTTTCG

TTAATACCGTCAAATACGCAGGTTGAACATCGTTTGCATGATCAATGGCAAAACGGTAAT

CCCCAGCGCTGGCACCTGACGCGAGACGATC

>151 bp product from linear template CP091563.1:c3703782-3702775 Salmonella enterica strain 143 chromosome, complete genome, base 802 to base 952 (Forward invj - Reverse invj).

CAGCGCTGGGGAAATGACTATTCCGTCAATATTCAGGCGCGGCAAGCAGGGGAGTTTTCG

TTAATACCGTCAAATACGCAGGTTGAACATCGTTTGCATGATCAATGGCAAAACGGTAAT

CCCCAGCGCTGGCACCTGACGCGAGACGATC

>151 bp product from linear template CP045947.1:1007197-1008204 Salmonella enterica subsp. enterica serovar Typhimurium strain AUSMDU00010530 chromosome, complete genome, base 802 to base 952 (Forward invj - Reverse invj).

CAGCGCTGGGGAAATGACTATTCCGTCAATATTCAGGCGCGGCAAGCAGGGGAGTTTTCG

TTAATACCGTCAAATACGCAGGTTGAACATCGTTTGCATGATCAATGGCAAAACGGTAAT

CCCCAGCGCTGGCACCTGACGCGAGACGATC

>151 bp product from linear template CP127343.1:1120437-1121444 Salmonella enterica strain SA746 chromosome, complete genome, base 802 to base 952 (Forward invj - Reverse invj).

CAGCGCTGGGGAAATGACTATTCCGTCAATATTCAGGCGCGGCAAGCAGGGGAGTTTTCG

TTAATACCGTCAAATACGCAGGTTGAACATCGTTTGCATGATCAATGGCAAAACGGTAAT

CCCCAGCGCTGGCACCTGACGCGAGACGATC

>151 bp product from linear template CP149391.1:c3625918-3624911 Salmonella enterica subsp. enterica serovar Typhimurium strain Z1323CSL0052 chromosome, complete genome, base 802 to base 952 (Forward invj - Reverse invj).

CAGCGCTGGGGAAATGACTATTCCGTCAATATTCAGGCGCGGCAAGCAGGGGAGTTTTCG

TTAATACCGTCAAATACGCAGGTTGAACATCGTTTGCATGATCAATGGCAAAACGGTAAT

CCCCAGCGCTGGCACCTGACGCGAGACGATC

>151 bp product from linear template CP090545.1:1120423-1121430 Salmonella enterica strain 2017028-SE chromosome, complete genome, base 802 to base 952 (Forward invj - Reverse invj).

CAGCGCTGGGGAAATGACTATTCCGTCAATATTCAGGCGCGGCAAGCAGGGGAGTTTTCG

TTAATACCGTCAAATACGCAGGTTGAACATCGTTTGCATGATCAATGGCAAAACGGTAAT

CCCCAGCGCTGGCACCTGACGCGAGACGATC

>151 bp product from linear template CP039576.1:c3070463-3069456 Salmonella enterica subsp. enterica serovar Typhimurium strain PNCS014856 chromosome, complete genome, base 802 to base 952 (Forward invj - Reverse invj).

CAGCGCTGGGGAAATGACTATTCCGTCAATATTCAGGCGCGGCAAGCAGGGGAGTTTTCG

TTAATACCGTCAAATACGCAGGTTGAACATCGTTTGCATGATCAATGGCAAAACGGTAAT

CCCCAGCGCTGGCACCTGACGCGAGACGATC

>151 bp product from linear template CP074324.1:1005250-1006257 Salmonella enterica subsp. enterica serovar Saintpaul strain CFSAN024564 chromosome, complete genome, base 802 to base 952 (Forward invj - Reverse invj).

CAGCGCTGGGGAAATGACTATTCCGTCAATATTCAGGCGCGGCAAGCAGGGGAGTTTTCG

TTAATACCGTCAAATACGCAGGTTGAACATCGTTTGCATGATCAATGGCAAAACGGTAAT

CCCCAGCGCTGGCACCTGACGCGAGACGATC

>151 bp product from linear template CP100732.1:1006658-1007665 Salmonella enterica subsp. enterica serovar Typhimurium strain R18.1932 chromosome, complete genome, base 802 to base 952 (Forward invj - Reverse invj).

CAGCGCTGGGGAAATGACTATTCCGTCAATATTCAGGCGCGGCAAGCAGGGGAGTTTTCG

TTAATACCGTCAAATACGCAGGTTGAACATCGTTTGCATGATCAATGGCAAAACGGTAAT

CCCCAGCGCTGGCACCTGACGCGAGACGATC

>151 bp product from linear template CP040648.1:c3083941-3082934 Salmonella enterica subsp. enterica serovar Rough O:-:- strain PNCS009880 chromosome, complete genome, base 802 to base 952 (Forward invj - Reverse invj).

CAGCGCTGGGGAAATGACTATTCCGTCAATATTCAGGCGCGGCAAGCAGGGGAGTTTTCG

TTAATACCGTCAAATACGCAGGTTGAACATCGTTTGCATGATCAATGGCAAAACGGTAAT

CCCCAGCGCTGGCACCTGACGCGAGACGATC

>151 bp product from linear template AP023299.1:1008067-1009074 Salmonella enterica subsp. enterica serovar 4,[5],12:i:- L-4334 DNA, complete genome, base 802 to base 952 (Forward invj - Reverse invj).

CAGCGCTGGGGAAATGACTATTCCGTCAATATTCAGGCGCGGCAAGCAGGGGAGTTTTCG

TTAATACCGTCAAATACGCAGGTTGAACATCGTTTGCATGATCAATGGCAAAACGGTAAT

CCCCAGCGCTGGCACCTGACGCGAGACGATC

>151 bp product from linear template CP039591.1:c3070069-3069062 Salmonella enterica subsp. enterica serovar Typhimurium strain PNCS014862 chromosome, complete genome, base 802 to base 952 (Forward invj - Reverse invj).

CAGCGCTGGGGAAATGACTATTCCGTCAATATTCAGGCGCGGCAAGCAGGGGAGTTTTCG

TTAATACCGTCAAATACGCAGGTTGAACATCGTTTGCATGATCAATGGCAAAACGGTAAT

CCCCAGCGCTGGCACCTGACGCGAGACGATC

>151 bp product from linear template CP113536.1:1041243-1042250 Salmonella enterica strain ZCX chromosome, complete genome, base 802 to base 952 (Forward invj - Reverse invj).

CAGCGCTGGGGAAATGACTATTCCGTCAATATTCAGGCGCGGCAAGCAGGGGAGTTTTCG

TTAATACCGTCAAATACGCAGGTTGAACATCGTTTGCATGATCAATGGCAAAACGGTAAT

CCCCAGCGCTGGCACCTGACGCGAGACGATC

>151 bp product from linear template CP149150.1:1039918-1040925 Salmonella enterica subsp. enterica serovar 4,[5],12:i:- strain Z1323SSL0059 chromosome, complete genome, base 802 to base 952 (Forward invj - Reverse invj).

CAGCGCTGGGGAAATGACTATTCCGTCAATATTCAGGCGCGGCAAGCAGGGGAGTTTTCG

TTAATACCGTCAAATACGCAGGTTGAACATCGTTTGCATGATCAATGGCAAAACGGTAAT

CCCCAGCGCTGGCACCTGACGCGAGACGATC

>151 bp product from linear template CP085819.1:2901338-2902345 Salmonella enterica subsp. enterica serovar Typhimurium strain Blastoise chromosome, complete genome, base 802 to base 952 (Forward invj - Reverse invj).

CAGCGCTGGGGAAATGACTATTCCGTCAATATTCAGGCGCGGCAAGCAGGGGAGTTTTCG

TTAATACCGTCAAATACGCAGGTTGAACATCGTTTGCATGATCAATGGCAAAACGGTAAT

CCCCAGCGCTGGCACCTGACGCGAGACGATC

>151 bp product from linear template CP044198.1:c2754728-2753721 Salmonella enterica subsp. enterica serovar Typhimurium strain AR-0408 chromosome, base 802 to base 952 (Forward invj - Reverse invj).

CAGCGCTGGGGAAATGACTATTCCGTCAATATTCAGGCGCGGCAAGCAGGGGAGTTTTCG

TTAATACCGTCAAATACGCAGGTTGAACATCGTTTGCATGATCAATGGCAAAACGGTAAT

CCCCAGCGCTGGCACCTGACGCGAGACGATC

>151 bp product from linear template CP149317.1:1008613-1009620 Salmonella enterica subsp. enterica serovar Typhimurium strain Z1323SSL0039 chromosome, complete genome, base 802 to base 952 (Forward invj - Reverse invj).

CAGCGCTGGGGAAATGACTATTCCGTCAATATTCAGGCGCGGCAAGCAGGGGAGTTTTCG

TTAATACCGTCAAATACGCAGGTTGAACATCGTTTGCATGATCAATGGCAAAACGGTAAT

CCCCAGCGCTGGCACCTGACGCGAGACGATC

>151 bp product from linear template CP014981.1:1048877-1049884 Salmonella enterica subsp. enterica serovar Typhimurium str. USDA-ARS-USMARC-1880 chromosome, complete genome, base 802 to base 952 (Forward invj - Reverse invj).

CAGCGCTGGGGAAATGACTATTCCGTCAATATTCAGGCGCGGCAAGCAGGGGAGTTTTCG

TTAATACCGTCAAATACGCAGGTTGAACATCGTTTGCATGATCAATGGCAAAACGGTAAT

CCCCAGCGCTGGCACCTGACGCGAGACGATC

>151 bp product from linear template CP051363.1:1007408-1008415 Salmonella enterica subsp. enterica serovar Typhimurium strain CVM 28321-b chromosome, complete genome, base 802 to base 952 (Forward invj - Reverse invj).

CAGCGCTGGGGAAATGACTATTCCGTCAATATTCAGGCGCGGCAAGCAGGGGAGTTTTCG

TTAATACCGTCAAATACGCAGGTTGAACATCGTTTGCATGATCAATGGCAAAACGGTAAT

CCCCAGCGCTGGCACCTGACGCGAGACGATC

>151 bp product from linear template CP149382.1:c3623402-3622395 Salmonella enterica subsp. enterica serovar Typhimurium strain Z1323CSL0062 chromosome, complete genome, base 802 to base 952 (Forward invj - Reverse invj).

CAGCGCTGGGGAAATGACTATTCCGTCAATATTCAGGCGCGGCAAGCAGGGGAGTTTTCG

TTAATACCGTCAAATACGCAGGTTGAACATCGTTTGCATGATCAATGGCAAAACGGTAAT

CCCCAGCGCTGGCACCTGACGCGAGACGATC

>151 bp product from linear template CP081189.2:1039916-1040923 Salmonella enterica strain sg1722-2 chromosome, complete genome, base 802 to base 952 (Forward invj - Reverse invj).

CAGCGCTGGGGAAATGACTATTCCGTCAATATTCAGGCGCGGCAAGCAGGGGAGTTTTCG

TTAATACCGTCAAATACGCAGGTTGAACATCGTTTGCATGATCAATGGCAAAACGGTAAT

CCCCAGCGCTGGCACCTGACGCGAGACGATC

>151 bp product from linear template CP149218.1:1120300-1121307 Salmonella enterica subsp. enterica serovar 4,[5],12:i:- strain Z1323HSL0022 chromosome, complete genome, base 802 to base 952 (Forward invj - Reverse invj).

CAGCGCTGGGGAAATGACTATTCCGTCAATATTCAGGCGCGGCAAGCAGGGGAGTTTTCG

TTAATACCGTCAAATACGCAGGTTGAACATCGTTTGCATGATCAATGGCAAAACGGTAAT

CCCCAGCGCTGGCACCTGACGCGAGACGATC

>151 bp product from linear template CP013720.1:c3070406-3069399 Salmonella enterica subsp. enterica serovar Typhimurium strain RM10607 chromosome, complete genome, base 802 to base 952 (Forward invj - Reverse invj).

CAGCGCTGGGGAAATGACTATTCCGTCAATATTCAGGCGCGGCAAGCAGGGGAGTTTTCG

TTAATACCGTCAAATACGCAGGTTGAACATCGTTTGCATGATCAATGGCAAAACGGTAAT

CCCCAGCGCTGGCACCTGACGCGAGACGATC

>151 bp product from linear template CP001363.1:c3056493-3055486 Salmonella enterica subsp. enterica serovar Typhimurium str. 14028S chromosome, complete genome, base 802 to base 952 (Forward invj - Reverse invj).

CAGCGCTGGGGAAATGACTATTCCGTCAATATTCAGGCGCGGCAAGCAGGGGAGTTTTCG

TTAATACCGTCAAATACGCAGGTTGAACATCGTTTGCATGATCAATGGCAAAACGGTAAT

CCCCAGCGCTGGCACCTGACGCGAGACGATC

>151 bp product from linear template LS997973.1:c3029246-3028239 Salmonella enterica subsp. enterica serovar Typhimurium strain D23580 genome assembly, chromosome: D23580_liv_o, base 802 to base 952 (Forward invj - Reverse invj).

CAGCGCTGGGGAAATGACTATTCCGTCAATATTCAGGCGCGGCAAGCAGGGGAGTTTTCG

TTAATACCGTCAAATACGCAGGTTGAACATCGTTTGCATGATCAATGGCAAAACGGTAAT

CCCCAGCGCTGGCACCTGACGCGAGACGATC

>151 bp product from linear template CP065076.1:1040982-1041989 Salmonella enterica subsp. enterica strain SENT0164 chromosome, complete genome, base 802 to base 952 (Forward invj - Reverse invj).

CAGCGCTGGGGAAATGACTATTCCGTCAATATTCAGGCGCGGCAAGCAGGGGAGTTTTCG

TTAATACCGTCAAATACGCAGGTTGAACATCGTTTGCATGATCAATGGCAAAACGGTAAT

CCCCAGCGCTGGCACCTGACGCGAGACGATC

>151 bp product from linear template AP023319.1:1008055-1009062 Salmonella enterica subsp. enterica serovar 4,[5],12:i:- L-4741 DNA, complete genome, base 802 to base 952 (Forward invj - Reverse invj).

CAGCGCTGGGGAAATGACTATTCCGTCAATATTCAGGCGCGGCAAGCAGGGGAGTTTTCG

TTAATACCGTCAAATACGCAGGTTGAACATCGTTTGCATGATCAATGGCAAAACGGTAAT

CCCCAGCGCTGGCACCTGACGCGAGACGATC

>151 bp product from linear template CP123648.1:1047820-1048827 Salmonella enterica strain 20MO07PC10-S1 chromosome, complete genome, base 802 to base 952 (Forward invj - Reverse invj).

CAGCGCTGGGGAAATGACTATTCCGTCAATATTCAGGCGCGGCAAGCAGGGGAGTTTTCG

TTAATACCGTCAAATACGCAGGTTGAACATCGTTTGCATGATCAATGGCAAAACGGTAAT

CCCCAGCGCTGGCACCTGACGCGAGACGATC

>151 bp product from linear template CP149415.1:1047612-1048619 Salmonella enterica subsp. enterica serovar Typhimurium strain Z1323SSL0037 chromosome, complete genome, base 802 to base 952 (Forward invj - Reverse invj).

CAGCGCTGGGGAAATGACTATTCCGTCAATATTCAGGCGCGGCAAGCAGGGGAGTTTTCG

TTAATACCGTCAAATACGCAGGTTGAACATCGTTTGCATGATCAATGGCAAAACGGTAAT

CCCCAGCGCTGGCACCTGACGCGAGACGATC

>151 bp product from linear template CP100715.1:1007955-1008962 Salmonella enterica subsp. enterica serovar Typhimurium strain R17.5474 chromosome, complete genome, base 802 to base 952 (Forward invj - Reverse invj).

CAGCGCTGGGGAAATGACTATTCCGTCAATATTCAGGCGCGGCAAGCAGGGGAGTTTTCG

TTAATACCGTCAAATACGCAGGTTGAACATCGTTTGCATGATCAATGGCAAAACGGTAAT

CCCCAGCGCTGGCACCTGACGCGAGACGATC

>151 bp product from linear template CP047542.1:c3959078-3958071 Salmonella enterica subsp. enterica serovar Typhimurium strain SJTUF10330 chromosome, complete genome, base 802 to base 952 (Forward invj - Reverse invj).

CAGCGCTGGGGAAATGACTATTCCGTCAATATTCAGGCGCGGCAAGCAGGGGAGTTTTCG

TTAATACCGTCAAATACGCAGGTTGAACATCGTTTGCATGATCAATGGCAAAACGGTAAT

CCCCAGCGCTGGCACCTGACGCGAGACGATC

>151 bp product from linear template CP149385.1:c3626150-3625143 Salmonella enterica subsp. enterica serovar Typhimurium strain Z1323CSL0057 chromosome, complete genome, base 802 to base 952 (Forward invj - Reverse invj).

CAGCGCTGGGGAAATGACTATTCCGTCAATATTCAGGCGCGGCAAGCAGGGGAGTTTTCG

TTAATACCGTCAAATACGCAGGTTGAACATCGTTTGCATGATCAATGGCAAAACGGTAAT

CCCCAGCGCTGGCACCTGACGCGAGACGATC

>151 bp product from linear template CP148770.1:c3504289-3503282 Salmonella enterica subsp. enterica serovar Infantis strain Z1323HSL0148 chromosome, complete genome, base 802 to base 952 (Forward invj - Reverse invj).

CAGCGCTGGGGAAATGACTATTCCGTCAATATTCAGGCGCGGCAAGCAGGGGAGTTTTCG

TTAATACCGTCAAATACGCAGGTTGAACATCGTTTGCATGATCAATGGCAAAACGGTAAT

CCCCAGCGCTGGCACCTGACGCGAGACGATC

>151 bp product from linear template CP082670.1:1018937-1019944 Salmonella enterica subsp. enterica serovar Typhimurium var. 5- strain CVM N17S016 chromosome, complete genome, base 802 to base 952 (Forward invj - Reverse invj).

CAGCGCTGGGGAAATGACTATTCCGTCAATATTCAGGCGCGGCAAGCAGGGGAGTTTTCG

TTAATACCGTCAAATACGCAGGTTGAACATCGTTTGCATGATCAATGGCAAAACGGTAAT

CCCCAGCGCTGGCACCTGACGCGAGACGATC

>151 bp product from linear template CP149359.1:c3868894-3867887 Salmonella enterica subsp. enterica serovar Typhimurium strain Z1323HSL0063 chromosome, complete genome, base 802 to base 952 (Forward invj - Reverse invj).

CAGCGCTGGGGAAATGACTATTCCGTCAATATTCAGGCGCGGCAAGCAGGGGAGTTTTCG

TTAATACCGTCAAATACGCAGGTTGAACATCGTTTGCATGATCAATGGCAAAACGGTAAT

CCCCAGCGCTGGCACCTGACGCGAGACGATC

>151 bp product from linear template KP279313.1:c29889-28882 Salmonella enterica subsp. enterica serovar Typhimurium strain KC14TY193 specific pathogenicity island SPI-1 genomic sequence, base 802 to base 952 (Forward invj - Reverse invj).

CAGCGCTGGGGAAATGACTATTCCGTCAATATTCAGGCGCGGCAAGCAGGGGAGTTTTCG

TTAATACCGTCAAATACGCAGGTTGAACATCGTTTGCATGATCAATGGCAAAACGGTAAT

CCCCAGCGCTGGCACCTGACGCGAGACGATC

>151 bp product from linear template CP091866.1:c3065061-3064054 Salmonella enterica subsp. enterica serovar 4,[5],12:- strain H1-012 chromosome, complete genome, base 802 to base 952 (Forward invj - Reverse invj).

CAGCGCTGGGGAAATGACTATTCCGTCAATATTCAGGCGCGGCAAGCAGGGGAGTTTTCG

TTAATACCGTCAAATACGCAGGTTGAACATCGTTTGCATGATCAATGGCAAAACGGTAAT

CCCCAGCGCTGGCACCTGACGCGAGACGATC

>151 bp product from linear template CP149190.1:1039955-1040962 Salmonella enterica subsp. enterica serovar 4,[5],12:i:- strain Z1323SSL0019 chromosome, complete genome, base 802 to base 952 (Forward invj - Reverse invj).

CAGCGCTGGGGAAATGACTATTCCGTCAATATTCAGGCGCGGCAAGCAGGGGAGTTTTCG

TTAATACCGTCAAATACGCAGGTTGAACATCGTTTGCATGATCAATGGCAAAACGGTAAT

CCCCAGCGCTGGCACCTGACGCGAGACGATC

>151 bp product from linear template AP023315.1:1008035-1009042 Salmonella enterica subsp. enterica serovar 4,[5],12:i:- L-4614 DNA, complete genome, base 802 to base 952 (Forward invj - Reverse invj).

CAGCGCTGGGGAAATGACTATTCCGTCAATATTCAGGCGCGGCAAGCAGGGGAGTTTTCG

TTAATACCGTCAAATACGCAGGTTGAACATCGTTTGCATGATCAATGGCAAAACGGTAAT

CCCCAGCGCTGGCACCTGACGCGAGACGATC

>151 bp product from linear template OU015340.1:4292775-4293782 Salmonella enterica subsp. enterica serovar Typhimurium strain AUSMDU00027944 genome assembly, chromosome: C1, base 802 to base 952 (Forward invj - Reverse invj).

CAGCGCTGGGGAAATGACTATTCCGTCAATATTCAGGCGCGGCAAGCAGGGGAGTTTTCG

TTAATACCGTCAAATACGCAGGTTGAACATCGTTTGCATGATCAATGGCAAAACGGTAAT

CCCCAGCGCTGGCACCTGACGCGAGACGATC

>151 bp product from linear template CP069164.1:c2920378-2919371 Salmonella enterica subsp. enterica serovar Typhimurium strain FD01851333 chromosome, base 802 to base 952 (Forward invj - Reverse invj).

CAGCGCTGGGGAAATGACTATTCCGTCAATATTCAGGCGCGGCAAGCAGGGGAGTTTTCG

TTAATACCGTCAAATACGCAGGTTGAACATCGTTTGCATGATCAATGGCAAAACGGTAAT

CCCCAGCGCTGGCACCTGACGCGAGACGATC

>151 bp product from linear template CP149146.1:1039918-1040925 Salmonella enterica subsp. enterica serovar 4,[5],12:i:- strain Z1323SSL0060 chromosome, complete genome, base 802 to base 952 (Forward invj - Reverse invj).

CAGCGCTGGGGAAATGACTATTCCGTCAATATTCAGGCGCGGCAAGCAGGGGAGTTTTCG

TTAATACCGTCAAATACGCAGGTTGAACATCGTTTGCATGATCAATGGCAAAACGGTAAT

CCCCAGCGCTGGCACCTGACGCGAGACGATC

>151 bp product from linear template CP160169.1:1000159-1001166 Salmonella enterica subsp. enterica strain N22-3274 chromosome, complete genome, base 802 to base 952 (Forward invj - Reverse invj).

CAGCGCTGGGGAAATGACTATTCCGTCAATATTCAGGCGCGGCAAGCAGGGGAGTTTTCG

TTAATACCGTCAAATACGCAGGTTGAACATCGTTTGCATGATCAATGGCAAAACGGTAAT

CCCCAGCGCTGGCACCTGACGCGAGACGATC

>151 bp product from linear template CP085699.1:1042459-1043466 Salmonella enterica subsp. enterica serovar Typhimurium strain S29 chromosome, complete genome, base 802 to base 952 (Forward invj - Reverse invj).

CAGCGCTGGGGAAATGACTATTCCGTCAATATTCAGGCGCGGCAAGCAGGGGAGTTTTCG

TTAATACCGTCAAATACGCAGGTTGAACATCGTTTGCATGATCAATGGCAAAACGGTAAT

CCCCAGCGCTGGCACCTGACGCGAGACGATC

>151 bp product from linear template CP149380.1:c3626142-3625135 Salmonella enterica subsp. enterica serovar Typhimurium strain Z1323HSL0012 chromosome, complete genome, base 802 to base 952 (Forward invj - Reverse invj).

CAGCGCTGGGGAAATGACTATTCCGTCAATATTCAGGCGCGGCAAGCAGGGGAGTTTTCG

TTAATACCGTCAAATACGCAGGTTGAACATCGTTTGCATGATCAATGGCAAAACGGTAAT

CCCCAGCGCTGGCACCTGACGCGAGACGATC

>151 bp product from linear template CP023166.1:1095655-1096662 Salmonella enterica subsp. enterica serovar Saintpaul strain SGB23 chromosome, complete genome, base 802 to base 952 (Forward invj - Reverse invj).

CAGCGCTGGGGAAATGACTATTCCGTCAATATTCAGGCGCGGCAAGCAGGGGAGTTTTCG

TTAATACCGTCAAATACGCAGGTTGAACATCGTTTGCATGATCAATGGCAAAACGGTAAT

CCCCAGCGCTGGCACCTGACGCGAGACGATC

>151 bp product from linear template AP023313.1:1008058-1009065 Salmonella enterica subsp. enterica serovar 4,[5],12:i:- L-4605 DNA, complete genome, base 802 to base 952 (Forward invj - Reverse invj).

CAGCGCTGGGGAAATGACTATTCCGTCAATATTCAGGCGCGGCAAGCAGGGGAGTTTTCG

TTAATACCGTCAAATACGCAGGTTGAACATCGTTTGCATGATCAATGGCAAAACGGTAAT

CCCCAGCGCTGGCACCTGACGCGAGACGATC

>151 bp product from linear template CP149283.1:c3759983-3758976 Salmonella enterica subsp. enterica serovar 4,[5],12:i:- strain Z1322HSL0022 chromosome, complete genome, base 802 to base 952 (Forward invj - Reverse invj).

CAGCGCTGGGGAAATGACTATTCCGTCAATATTCAGGCGCGGCAAGCAGGGGAGTTTTCG

TTAATACCGTCAAATACGCAGGTTGAACATCGTTTGCATGATCAATGGCAAAACGGTAAT

CCCCAGCGCTGGCACCTGACGCGAGACGATC

>151 bp product from linear template CP149371.1:1386891-1387898 Salmonella enterica subsp. enterica serovar Typhimurium strain Z1323HSL0038 chromosome, complete genome, base 802 to base 952 (Forward invj - Reverse invj).

CAGCGCTGGGGAAATGACTATTCCGTCAATATTCAGGCGCGGCAAGCAGGGGAGTTTTCG

TTAATACCGTCAAATACGCAGGTTGAACATCGTTTGCATGATCAATGGCAAAACGGTAAT

CCCCAGCGCTGGCACCTGACGCGAGACGATC

>151 bp product from linear template CP149210.1:c3705094-3704087 Salmonella enterica subsp. enterica serovar 4,[5],12:i:- strain Z1323HSL0050 chromosome, complete genome, base 802 to base 952 (Forward invj - Reverse invj).

CAGCGCTGGGGAAATGACTATTCCGTCAATATTCAGGCGCGGCAAGCAGGGGAGTTTTCG

TTAATACCGTCAAATACGCAGGTTGAACATCGTTTGCATGATCAATGGCAAAACGGTAAT

CCCCAGCGCTGGCACCTGACGCGAGACGATC

>151 bp product from linear template CP149406.1:1007692-1008699 Salmonella enterica subsp. enterica serovar Typhimurium strain Z1322HSL0030 chromosome, complete genome, base 802 to base 952 (Forward invj - Reverse invj).

CAGCGCTGGGGAAATGACTATTCCGTCAATATTCAGGCGCGGCAAGCAGGGGAGTTTTCG

TTAATACCGTCAAATACGCAGGTTGAACATCGTTTGCATGATCAATGGCAAAACGGTAAT

CCCCAGCGCTGGCACCTGACGCGAGACGATC

>151 bp product from linear template CP137712.1:c4326776-4325769 Mutant Salmonella enterica isolate FELIX_MS734, complete genome, base 802 to base 952 (Forward invj - Reverse invj).

CAGCGCTGGGGAAATGACTATTCCGTCAATATTCAGGCGCGGCAAGCAGGGGAGTTTTCG

TTAATACCGTCAAATACGCAGGTTGAACATCGTTTGCATGATCAATGGCAAAACGGTAAT

CCCCAGCGCTGGCACCTGACGCGAGACGATC

>151 bp product from linear template CP039582.1:c3070048-3069041 Salmonella enterica subsp. enterica serovar 1,4,[5],12:i:- strain PNCS014858 chromosome, complete genome, base 802 to base 952 (Forward invj - Reverse invj).

CAGCGCTGGGGAAATGACTATTCCGTCAATATTCAGGCGCGGCAAGCAGGGGAGTTTTCG

TTAATACCGTCAAATACGCAGGTTGAACATCGTTTGCATGATCAATGGCAAAACGGTAAT

CCCCAGCGCTGGCACCTGACGCGAGACGATC

>151 bp product from linear template CP021462.1:c3026374-3025367 Salmonella enterica subsp. enterica serovar Typhimurium strain UGA14 chromosome, complete genome, base 802 to base 952 (Forward invj - Reverse invj).

CAGCGCTGGGGAAATGACTATTCCGTCAATATTCAGGCGCGGCAAGCAGGGGAGTTTTCG

TTAATACCGTCAAATACGCAGGTTGAACATCGTTTGCATGATCAATGGCAAAACGGTAAT

CCCCAGCGCTGGCACCTGACGCGAGACGATC

>151 bp product from linear template CP149324.1:1043647-1044654 Salmonella enterica subsp. enterica serovar Typhimurium strain Z1323SSL0033 chromosome, complete genome, base 802 to base 952 (Forward invj - Reverse invj).

CAGCGCTGGGGAAATGACTATTCCGTCAATATTCAGGCGCGGCAAGCAGGGGAGTTTTCG

TTAATACCGTCAAATACGCAGGTTGAACATCGTTTGCATGATCAATGGCAAAACGGTAAT

CCCCAGCGCTGGCACCTGACGCGAGACGATC

>151 bp product from linear template LR881463.1:c3923209-3922202 Salmonella enterica subsp. enterica serovar Typhimurium isolate S.Tm LT2p22_assembled genome assembly, chromosome: 1_Linear-, base 802 to base 952 (Forward invj - Reverse invj).

CAGCGCTGGGGAAATGACTATTCCGTCAATATTCAGGCGCGGCAAGCAGGGGAGTTTTCG

TTAATACCGTCAAATACGCAGGTTGAACATCGTTTGCATGATCAATGGCAAAACGGTAAT

CCCCAGCGCTGGCACCTGACGCGAGACGATC

>151 bp product from linear template CP082515.1:3597685-3598692 Salmonella enterica subsp. enterica serovar Typhimurium strain FSIS1606077 chromosome, base 802 to base 952 (Forward invj - Reverse invj).

CAGCGCTGGGGAAATGACTATTCCGTCAATATTCAGGCGCGGCAAGCAGGGGAGTTTTCG

TTAATACCGTCAAATACGCAGGTTGAACATCGTTTGCATGATCAATGGCAAAACGGTAAT

CCCCAGCGCTGGCACCTGACGCGAGACGATC

>151 bp product from linear template CP090529.1:1039740-1040747 Salmonella enterica strain 2008079-SE chromosome, complete genome, base 802 to base 952 (Forward invj - Reverse invj).

CAGCGCTGGGGAAATGACTATTCCGTCAATATTCAGGCGCGGCAAGCAGGGGAGTTTTCG

TTAATACCGTCAAATACGCAGGTTGAACATCGTTTGCATGATCAATGGCAAAACGGTAAT

CCCCAGCGCTGGCACCTGACGCGAGACGATC

>151 bp product from linear template CP149140.1:c3800549-3799542 Salmonella enterica subsp. enterica serovar 4,[5],12:i:- strain Z1323SSL0063 chromosome, complete genome, base 802 to base 952 (Forward invj - Reverse invj).

CAGCGCTGGGGAAATGACTATTCCGTCAATATTCAGGCGCGGCAAGCAGGGGAGTTTTCG

TTAATACCGTCAAATACGCAGGTTGAACATCGTTTGCATGATCAATGGCAAAACGGTAAT

CCCCAGCGCTGGCACCTGACGCGAGACGATC

>151 bp product from linear template AP023290.1:1039931-1040938 Salmonella enterica subsp. enterica serovar 4,[5],12:i:- L-3844 DNA, complete genome, base 802 to base 952 (Forward invj - Reverse invj).

CAGCGCTGGGGAAATGACTATTCCGTCAATATTCAGGCGCGGCAAGCAGGGGAGTTTTCG

TTAATACCGTCAAATACGCAGGTTGAACATCGTTTGCATGATCAATGGCAAAACGGTAAT

CCCCAGCGCTGGCACCTGACGCGAGACGATC

>151 bp product from linear template CP149394.1:c3626150-3625143 Salmonella enterica subsp. enterica serovar Typhimurium strain Z1323CSL0051 chromosome, complete genome, base 802 to base 952 (Forward invj - Reverse invj).

CAGCGCTGGGGAAATGACTATTCCGTCAATATTCAGGCGCGGCAAGCAGGGGAGTTTTCG

TTAATACCGTCAAATACGCAGGTTGAACATCGTTTGCATGATCAATGGCAAAACGGTAAT

CCCCAGCGCTGGCACCTGACGCGAGACGATC

>151 bp product from linear template CP110201.1:1039781-1040788 Salmonella enterica subsp. enterica serovar Typhimurium strain 1104-65 chromosome, complete genome, base 802 to base 952 (Forward invj - Reverse invj).

CAGCGCTGGGGAAATGACTATTCCGTCAATATTCAGGCGCGGCAAGCAGGGGAGTTTTCG

TTAATACCGTCAAATACGCAGGTTGAACATCGTTTGCATGATCAATGGCAAAACGGTAAT

CCCCAGCGCTGGCACCTGACGCGAGACGATC

>151 bp product from linear template CP039599.1:c2985103-2984096 Salmonella enterica subsp. enterica serovar 1,4,[5],12:i:- strain PNCS014867 chromosome, complete genome, base 802 to base 952 (Forward invj - Reverse invj).

CAGCGCTGGGGAAATGACTATTCCGTCAATATTCAGGCGCGGCAAGCAGGGGAGTTTTCG

TTAATACCGTCAAATACGCAGGTTGAACATCGTTTGCATGATCAATGGCAAAACGGTAAT

CCCCAGCGCTGGCACCTGACGCGAGACGATC

>151 bp product from linear template CP082751.1:1010289-1011296 Salmonella enterica subsp. enterica serovar Typhimurium var. 5- strain CVM N16S021 chromosome, complete genome, base 802 to base 952 (Forward invj - Reverse invj).

CAGCGCTGGGGAAATGACTATTCCGTCAATATTCAGGCGCGGCAAGCAGGGGAGTTTTCG

TTAATACCGTCAAATACGCAGGTTGAACATCGTTTGCATGATCAATGGCAAAACGGTAAT

CCCCAGCGCTGGCACCTGACGCGAGACGATC

>151 bp product from linear template CP040562.1:1009554-1010561 Salmonella enterica subsp. enterica serovar Typhimurium strain SAP17-7399 chromosome, complete genome, base 802 to base 952 (Forward invj - Reverse invj).

CAGCGCTGGGGAAATGACTATTCCGTCAATATTCAGGCGCGGCAAGCAGGGGAGTTTTCG

TTAATACCGTCAAATACGCAGGTTGAACATCGTTTGCATGATCAATGGCAAAACGGTAAT

CCCCAGCGCTGGCACCTGACGCGAGACGATC

>151 bp product from linear template CP101382.1:1088705-1089712 Salmonella enterica strain SC2017030 chromosome, complete genome, base 802 to base 952 (Forward invj - Reverse invj).

CAGCGCTGGGGAAATGACTATTCCGTCAATATTCAGGCGCGGCAAGCAGGGGAGTTTTCG

TTAATACCGTCAAATACGCAGGTTGAACATCGTTTGCATGATCAATGGCAAAACGGTAAT

CCCCAGCGCTGGCACCTGACGCGAGACGATC

>151 bp product from linear template CP149278.1:4277771-4278778 Salmonella enterica subsp. enterica serovar 4,[5],12:i:- strain Z1322HSL0026 chromosome, complete genome, base 802 to base 952 (Forward invj - Reverse invj).

CAGCGCTGGGGAAATGACTATTCCGTCAATATTCAGGCGCGGCAAGCAGGGGAGTTTTCG

TTAATACCGTCAAATACGCAGGTTGAACATCGTTTGCATGATCAATGGCAAAACGGTAAT

CCCCAGCGCTGGCACCTGACGCGAGACGATC

>151 bp product from linear template CP102198.1:c659124-658117 Salmonella enterica subsp. enterica strain IdAB45 chromosome, complete genome, base 802 to base 952 (Forward invj - Reverse invj).

CAGCGCTGGGGAAATGACTATTCCGTCAATATTCAGGCGCGGCAAGCAGGGGAGTTTTCG

TTAATACCGTCAAATACGCAGGTTGAACATCGTTTGCATGATCAATGGCAAAACGGTAAT

CCCCAGCGCTGGCACCTGACGCGAGACGATC

>151 bp product from linear template CP093386.1:1007162-1008169 Salmonella enterica strain P048595 chromosome, complete genome, base 802 to base 952 (Forward invj - Reverse invj).

CAGCGCTGGGGAAATGACTATTCCGTCAATATTCAGGCGCGGCAAGCAGGGGAGTTTTCG

TTAATACCGTCAAATACGCAGGTTGAACATCGTTTGCATGATCAATGGCAAAACGGTAAT

CCCCAGCGCTGGCACCTGACGCGAGACGATC

>151 bp product from linear template CP039856.1:c3007043-3006036 Salmonella enterica subsp. enterica serovar 1,4,[5],12:i:- strain PNCS014876 chromosome, complete genome, base 802 to base 952 (Forward invj - Reverse invj).

CAGCGCTGGGGAAATGACTATTCCGTCAATATTCAGGCGCGGCAAGCAGGGGAGTTTTCG

TTAATACCGTCAAATACGCAGGTTGAACATCGTTTGCATGATCAATGGCAAAACGGTAAT

CCCCAGCGCTGGCACCTGACGCGAGACGATC

>151 bp product from linear template CP149298.1:1959479-1960486 Salmonella enterica subsp. enterica serovar Typhimurium strain Z1323SSL0057 chromosome, complete genome, base 802 to base 952 (Forward invj - Reverse invj).

CAGCGCTGGGGAAATGACTATTCCGTCAATATTCAGGCGCGGCAAGCAGGGGAGTTTTCG

TTAATACCGTCAAATACGCAGGTTGAACATCGTTTGCATGATCAATGGCAAAACGGTAAT

CCCCAGCGCTGGCACCTGACGCGAGACGATC

>151 bp product from linear template CP033257.2:1040102-1041109 Salmonella enterica subsp. enterica strain CFSA12 chromosome, complete genome, base 802 to base 952 (Forward invj - Reverse invj).

CAGCGCTGGGGAAATGACTATTCCGTCAATATTCAGGCGCGGCAAGCAGGGGAGTTTTCG

TTAATACCGTCAAATACGCAGGTTGAACATCGTTTGCATGATCAATGGCAAAACGGTAAT

CCCCAGCGCTGGCACCTGACGCGAGACGATC

>151 bp product from linear template FN424405.1:c3029244-3028237 Salmonella enterica subsp. enterica serovar Typhimurium str. D23580 complete genome, base 802 to base 952 (Forward invj - Reverse invj).

CAGCGCTGGGGAAATGACTATTCCGTCAATATTCAGGCGCGGCAAGCAGGGGAGTTTTCG

TTAATACCGTCAAATACGCAGGTTGAACATCGTTTGCATGATCAATGGCAAAACGGTAAT

CCCCAGCGCTGGCACCTGACGCGAGACGATC

>151 bp product from linear template CP033255.2:1041479-1042486 Salmonella enterica subsp. enterica strain CFSA244 chromosome, complete genome, base 802 to base 952 (Forward invj - Reverse invj).

CAGCGCTGGGGAAATGACTATTCCGTCAATATTCAGGCGCGGCAAGCAGGGGAGTTTTCG

TTAATACCGTCAAATACGCAGGTTGAACATCGTTTGCATGATCAATGGCAAAACGGTAAT

CCCCAGCGCTGGCACCTGACGCGAGACGATC

>151 bp product from linear template CP149353.1:1356994-1358001 Salmonella enterica subsp. enterica serovar Typhimurium strain Z1323HSL0078 chromosome, complete genome, base 802 to base 952 (Forward invj - Reverse invj).

CAGCGCTGGGGAAATGACTATTCCGTCAATATTCAGGCGCGGCAAGCAGGGGAGTTTTCG

TTAATACCGTCAAATACGCAGGTTGAACATCGTTTGCATGATCAATGGCAAAACGGTAAT

CCCCAGCGCTGGCACCTGACGCGAGACGATC

>151 bp product from linear template CP149198.1:1039755-1040762 Salmonella enterica subsp. enterica serovar 4,[5],12:i:- strain Z1323SSL0013 chromosome, complete genome, base 802 to base 952 (Forward invj - Reverse invj).

CAGCGCTGGGGAAATGACTATTCCGTCAATATTCAGGCGCGGCAAGCAGGGGAGTTTTCG

TTAATACCGTCAAATACGCAGGTTGAACATCGTTTGCATGATCAATGGCAAAACGGTAAT

CCCCAGCGCTGGCACCTGACGCGAGACGATC

>151 bp product from linear template CP149269.1:1039722-1040729 Salmonella enterica subsp. enterica serovar 4,[5],12:i:- strain Z1322HSL0033 chromosome, complete genome, base 802 to base 952 (Forward invj - Reverse invj).

CAGCGCTGGGGAAATGACTATTCCGTCAATATTCAGGCGCGGCAAGCAGGGGAGTTTTCG

TTAATACCGTCAAATACGCAGGTTGAACATCGTTTGCATGATCAATGGCAAAACGGTAAT

CCCCAGCGCTGGCACCTGACGCGAGACGATC

>151 bp product from linear template CP067091.1:c2973595-2972588 Salmonella enterica subsp. enterica serovar Typhimurium strain ER3625 chromosome, complete genome, base 802 to base 952 (Forward invj - Reverse invj).

CAGCGCTGGGGAAATGACTATTCCGTCAATATTCAGGCGCGGCAAGCAGGGGAGTTTTCG

TTAATACCGTCAAATACGCAGGTTGAACATCGTTTGCATGATCAATGGCAAAACGGTAAT

CCCCAGCGCTGGCACCTGACGCGAGACGATC

>151 bp product from linear template CP083385.1:1093799-1094806 Salmonella enterica subsp. enterica serovar Saintpaul strain CVM N18S0175 chromosome, complete genome, base 802 to base 952 (Forward invj - Reverse invj).

CAGCGCTGGGGAAATGACTATTCCGTCAATATTCAGGCGCGGCAAGCAGGGGAGTTTTCG

TTAATACCGTCAAATACGCAGGTTGAACATCGTTTGCATGATCAATGGCAAAACGGTAAT

CCCCAGCGCTGGCACCTGACGCGAGACGATC

>151 bp product from linear template CP082608.1:1039931-1040938 Salmonella enterica subsp. enterica serovar 4,[5],12:i:- strain CVM N17S107 isolate 17LA02GT01-S chromosome, complete genome, base 802 to base 952 (Forward invj - Reverse invj).

CAGCGCTGGGGAAATGACTATTCCGTCAATATTCAGGCGCGGCAAGCAGGGGAGTTTTCG

TTAATACCGTCAAATACGCAGGTTGAACATCGTTTGCATGATCAATGGCAAAACGGTAAT

CCCCAGCGCTGGCACCTGACGCGAGACGATC

>151 bp product from linear template CP014358.1:c3071446-3070439 Salmonella enterica subsp. enterica serovar Typhimurium strain YU15, complete genome, base 802 to base 952 (Forward invj - Reverse invj).

CAGCGCTGGGGAAATGACTATTCCGTCAATATTCAGGCGCGGCAAGCAGGGGAGTTTTCG

TTAATACCGTCAAATACGCAGGTTGAACATCGTTTGCATGATCAATGGCAAAACGGTAAT

CCCCAGCGCTGGCACCTGACGCGAGACGATC

>151 bp product from linear template CP101376.1:1039867-1040874 Salmonella enterica strain SC2016091 chromosome, complete genome, base 802 to base 952 (Forward invj - Reverse invj).

CAGCGCTGGGGAAATGACTATTCCGTCAATATTCAGGCGCGGCAAGCAGGGGAGTTTTCG

TTAATACCGTCAAATACGCAGGTTGAACATCGTTTGCATGATCAATGGCAAAACGGTAAT

CCCCAGCGCTGGCACCTGACGCGAGACGATC

>151 bp product from linear template CP018219.1:2723574-2724581 Salmonella enterica subsp. enterica strain LSP 389/97 chromosome, complete genome, base 802 to base 952 (Forward invj - Reverse invj).

CAGCGCTGGGGAAATGACTATTCCGTCAATATTCAGGCGCGGCAAGCAGGGGAGTTTTCG

TTAATACCGTCAAATACGCAGGTTGAACATCGTTTGCATGATCAATGGCAAAACGGTAAT

CCCCAGCGCTGGCACCTGACGCGAGACGATC

>151 bp product from linear template CP149203.1:4689725-4690732 Salmonella enterica subsp. enterica serovar 4,[5],12:i:- strain Z1323HSL0090 chromosome, complete genome, base 802 to base 952 (Forward invj - Reverse invj).

CAGCGCTGGGGAAATGACTATTCCGTCAATATTCAGGCGCGGCAAGCAGGGGAGTTTTCG

TTAATACCGTCAAATACGCAGGTTGAACATCGTTTGCATGATCAATGGCAAAACGGTAAT

CCCCAGCGCTGGCACCTGACGCGAGACGATC

>151 bp product from linear template CP029568.1:c3584890-3583883 Salmonella enterica subsp. enterica serovar Typhimurium strain DA34837 chromosome, complete genome, base 802 to base 952 (Forward invj - Reverse invj).

CAGCGCTGGGGAAATGACTATTCCGTCAATATTCAGGCGCGGCAAGCAGGGGAGTTTTCG

TTAATACCGTCAAATACGCAGGTTGAACATCGTTTGCATGATCAATGGCAAAACGGTAAT

CCCCAGCGCTGGCACCTGACGCGAGACGATC

>151 bp product from linear template CP050750.1:1039925-1040932 Salmonella enterica subsp. enterica serovar Typhimurium strain ST46 chromosome, complete genome, base 802 to base 952 (Forward invj - Reverse invj).

CAGCGCTGGGGAAATGACTATTCCGTCAATATTCAGGCGCGGCAAGCAGGGGAGTTTTCG

TTAATACCGTCAAATACGCAGGTTGAACATCGTTTGCATGATCAATGGCAAAACGGTAAT

CCCCAGCGCTGGCACCTGACGCGAGACGATC

>151 bp product from linear template CP047531.1:1039731-1040738 Salmonella enterica subsp. enterica serovar Typhimurium strain SJTUF10484 chromosome, complete genome, base 802 to base 952 (Forward invj - Reverse invj).

CAGCGCTGGGGAAATGACTATTCCGTCAATATTCAGGCGCGGCAAGCAGGGGAGTTTTCG

TTAATACCGTCAAATACGCAGGTTGAACATCGTTTGCATGATCAATGGCAAAACGGTAAT

CCCCAGCGCTGGCACCTGACGCGAGACGATC

>151 bp product from linear template CP039603.1:c2997605-2996598 Salmonella enterica subsp. enterica serovar 1,4,[5],12:i:- strain PNCS014868 chromosome, complete genome, base 802 to base 952 (Forward invj - Reverse invj).

CAGCGCTGGGGAAATGACTATTCCGTCAATATTCAGGCGCGGCAAGCAGGGGAGTTTTCG

TTAATACCGTCAAATACGCAGGTTGAACATCGTTTGCATGATCAATGGCAAAACGGTAAT

CCCCAGCGCTGGCACCTGACGCGAGACGATC

>151 bp product from linear template CP040686.1:c3065394-3064387 Salmonella enterica subsp. enterica serovar 4,[5],12:i:- strain USDA15WA-1 chromosome, complete genome, base 802 to base 952 (Forward invj - Reverse invj).

CAGCGCTGGGGAAATGACTATTCCGTCAATATTCAGGCGCGGCAAGCAGGGGAGTTTTCG

TTAATACCGTCAAATACGCAGGTTGAACATCGTTTGCATGATCAATGGCAAAACGGTAAT

CCCCAGCGCTGGCACCTGACGCGAGACGATC

>151 bp product from linear template CP050728.1:1039578-1040585 Salmonella enterica subsp. enterica serovar Typhimurium strain ST106 chromosome, complete genome, base 802 to base 952 (Forward invj - Reverse invj).

CAGCGCTGGGGAAATGACTATTCCGTCAATATTCAGGCGCGGCAAGCAGGGGAGTTTTCG

TTAATACCGTCAAATACGCAGGTTGAACATCGTTTGCATGATCAATGGCAAAACGGTAAT

CCCCAGCGCTGGCACCTGACGCGAGACGATC

>151 bp product from linear template CP074663.1:1007663-1008670 Salmonella enterica subsp. enterica serovar Typhimurium strain CFSAN008081 chromosome, complete genome, base 802 to base 952 (Forward invj - Reverse invj).

CAGCGCTGGGGAAATGACTATTCCGTCAATATTCAGGCGCGGCAAGCAGGGGAGTTTTCG

TTAATACCGTCAAATACGCAGGTTGAACATCGTTTGCATGATCAATGGCAAAACGGTAAT

CCCCAGCGCTGGCACCTGACGCGAGACGATC

>151 bp product from linear template CP082681.1:c3947341-3946334 Salmonella enterica subsp. enterica serovar Typhimurium strain CVM N18S0597 chromosome, complete genome, base 802 to base 952 (Forward invj - Reverse invj).

CAGCGCTGGGGAAATGACTATTCCGTCAATATTCAGGCGCGGCAAGCAGGGGAGTTTTCG

TTAATACCGTCAAATACGCAGGTTGAACATCGTTTGCATGATCAATGGCAAAACGGTAAT

CCCCAGCGCTGGCACCTGACGCGAGACGATC

>151 bp product from linear template AP011957.1:c3059686-3058679 Salmonella enterica subsp. enterica serovar Typhimurium str. T000240 DNA, complete genome, base 802 to base 952 (Forward invj - Reverse invj).

CAGCGCTGGGGAAATGACTATTCCGTCAATATTCAGGCGCGGCAAGCAGGGGAGTTTTCG

TTAATACCGTCAAATACGCAGGTTGAACATCGTTTGCATGATCAATGGCAAAACGGTAAT

CCCCAGCGCTGGCACCTGACGCGAGACGATC

>151 bp product from linear template CP149285.1:1040056-1041063 Salmonella enterica subsp. enterica serovar 4,[5],12:i:- strain Z1322HSL0011 chromosome, complete genome, base 802 to base 952 (Forward invj - Reverse invj).

CAGCGCTGGGGAAATGACTATTCCGTCAATATTCAGGCGCGGCAAGCAGGGGAGTTTTCG

TTAATACCGTCAAATACGCAGGTTGAACATCGTTTGCATGATCAATGGCAAAACGGTAAT

CCCCAGCGCTGGCACCTGACGCGAGACGATC

>151 bp product from linear template CP039713.1:c3001901-3000894 Salmonella enterica subsp. enterica serovar 1,4,[5],12:i:- strain PNCS014853 chromosome, complete genome, base 802 to base 952 (Forward invj - Reverse invj).

CAGCGCTGGGGAAATGACTATTCCGTCAATATTCAGGCGCGGCAAGCAGGGGAGTTTTCG

TTAATACCGTCAAATACGCAGGTTGAACATCGTTTGCATGATCAATGGCAAAACGGTAAT

CCCCAGCGCTGGCACCTGACGCGAGACGATC

>151 bp product from linear template CP064919.1:c3043422-3042415 Salmonella enterica subsp. enterica serovar Typhimurium strain AB42049 chromosome, complete genome, base 802 to base 952 (Forward invj - Reverse invj).

CAGCGCTGGGGAAATGACTATTCCGTCAATATTCAGGCGCGGCAAGCAGGGGAGTTTTCG

TTAATACCGTCAAATACGCAGGTTGAACATCGTTTGCATGATCAATGGCAAAACGGTAAT

CCCCAGCGCTGGCACCTGACGCGAGACGATC

>151 bp product from linear template CP082391.1:1041240-1042247 Salmonella enterica subsp. enterica serovar 4,[5],12:i:- strain FSIS1700433 chromosome, complete genome, base 802 to base 952 (Forward invj - Reverse invj).

CAGCGCTGGGGAAATGACTATTCCGTCAATATTCAGGCGCGGCAAGCAGGGGAGTTTTCG

TTAATACCGTCAAATACGCAGGTTGAACATCGTTTGCATGATCAATGGCAAAACGGTAAT

CCCCAGCGCTGGCACCTGACGCGAGACGATC

>151 bp product from linear template CP061122.1:1039692-1040699 Salmonella enterica subsp. enterica serovar Typhimurium strain S441 chromosome, complete genome, base 802 to base 952 (Forward invj - Reverse invj).

CAGCGCTGGGGAAATGACTATTCCGTCAATATTCAGGCGCGGCAAGCAGGGGAGTTTTCG

TTAATACCGTCAAATACGCAGGTTGAACATCGTTTGCATGATCAATGGCAAAACGGTAAT

CCCCAGCGCTGGCACCTGACGCGAGACGATC

>151 bp product from linear template CP149375.1:c3626274-3625267 Salmonella enterica subsp. enterica serovar Typhimurium strain Z1323HSL0029 chromosome, complete genome, base 802 to base 952 (Forward invj - Reverse invj).

CAGCGCTGGGGAAATGACTATTCCGTCAATATTCAGGCGCGGCAAGCAGGGGAGTTTTCG

TTAATACCGTCAAATACGCAGGTTGAACATCGTTTGCATGATCAATGGCAAAACGGTAAT

CCCCAGCGCTGGCACCTGACGCGAGACGATC

>151 bp product from linear template LT795114.1:1039868-1040875 Salmonella enterica subsp. enterica serovar Typhimurium isolate VNB151-sc-2315230 genome assembly, chromosome: 1, base 802 to base 952 (Forward invj - Reverse invj).

CAGCGCTGGGGAAATGACTATTCCGTCAATATTCAGGCGCGGCAAGCAGGGGAGTTTTCG

TTAATACCGTCAAATACGCAGGTTGAACATCGTTTGCATGATCAATGGCAAAACGGTAAT

CCCCAGCGCTGGCACCTGACGCGAGACGATC

>151 bp product from linear template CP145533.1:1120581-1121588 Salmonella enterica subsp. enterica serovar 4,[5],12:i:- strain ZC055 chromosome, complete genome, base 802 to base 952 (Forward invj - Reverse invj).

CAGCGCTGGGGAAATGACTATTCCGTCAATATTCAGGCGCGGCAAGCAGGGGAGTTTTCG

TTAATACCGTCAAATACGCAGGTTGAACATCGTTTGCATGATCAATGGCAAAACGGTAAT

CCCCAGCGCTGGCACCTGACGCGAGACGATC

>151 bp product from linear template CP074617.1:1039436-1040443 Salmonella enterica subsp. enterica serovar Typhimurium strain SGSC 2190 chromosome, complete genome, base 802 to base 952 (Forward invj - Reverse invj).

CAGCGCTGGGGAAATGACTATTCCGTCAATATTCAGGCGCGGCAAGCAGGGGAGTTTTCG

TTAATACCGTCAAATACGCAGGTTGAACATCGTTTGCATGATCAATGGCAAAACGGTAAT

CCCCAGCGCTGGCACCTGACGCGAGACGATC

>151 bp product from linear template CP148772.1:c689087-688080 Salmonella enterica subsp. enterica serovar Infantis strain Z1323HSL0127 chromosome, complete genome, base 802 to base 952 (Forward invj - Reverse invj).

CAGCGCTGGGGAAATGACTATTCCGTCAATATTCAGGCGCGGCAAGCAGGGGAGTTTTCG

TTAATACCGTCAAATACGCAGGTTGAACATCGTTTGCATGATCAATGGCAAAACGGTAAT

CCCCAGCGCTGGCACCTGACGCGAGACGATC

>151 bp product from linear template CP121262.1:1007448-1008455 Salmonella enterica subsp. enterica serovar Typhimurium strain 013 chromosome, complete genome, base 802 to base 952 (Forward invj - Reverse invj).

CAGCGCTGGGGAAATGACTATTCCGTCAATATTCAGGCGCGGCAAGCAGGGGAGTTTTCG

TTAATACCGTCAAATACGCAGGTTGAACATCGTTTGCATGATCAATGGCAAAACGGTAAT

CCCCAGCGCTGGCACCTGACGCGAGACGATC

>151 bp product from linear template CP077668.1:1039906-1040913 Salmonella enterica strain SP chromosome, complete genome, base 802 to base 952 (Forward invj - Reverse invj).

CAGCGCTGGGGAAATGACTATTCCGTCAATATTCAGGCGCGGCAAGCAGGGGAGTTTTCG

TTAATACCGTCAAATACGCAGGTTGAACATCGTTTGCATGATCAATGGCAAAACGGTAAT

CCCCAGCGCTGGCACCTGACGCGAGACGATC

>151 bp product from linear template CP090539.1:1120507-1121514 Salmonella enterica strain 2016062-SE chromosome, complete genome, base 802 to base 952 (Forward invj - Reverse invj).

CAGCGCTGGGGAAATGACTATTCCGTCAATATTCAGGCGCGGCAAGCAGGGGAGTTTTCG

TTAATACCGTCAAATACGCAGGTTGAACATCGTTTGCATGATCAATGGCAAAACGGTAAT

CCCCAGCGCTGGCACCTGACGCGAGACGATC

>151 bp product from linear template CP149408.1:1002875-1003882 Salmonella enterica subsp. enterica serovar Typhimurium strain Z1322HSL0025 chromosome, complete genome, base 802 to base 952 (Forward invj - Reverse invj).

CAGCGCTGGGGAAATGACTATTCCGTCAATATTCAGGCGCGGCAAGCAGGGGAGTTTTCG

TTAATACCGTCAAATACGCAGGTTGAACATCGTTTGCATGATCAATGGCAAAACGGTAAT

CCCCAGCGCTGGCACCTGACGCGAGACGATC

>151 bp product from linear template CP061044.1:1083330-1084337 Salmonella enterica subsp. enterica serovar Typhimurium strain BBS1407 chromosome, complete genome, base 802 to base 952 (Forward invj - Reverse invj).

CAGCGCTGGGGAAATGACTATTCCGTCAATATTCAGGCGCGGCAAGCAGGGGAGTTTTCG

TTAATACCGTCAAATACGCAGGTTGAACATCGTTTGCATGATCAATGGCAAAACGGTAAT

CCCCAGCGCTGGCACCTGACGCGAGACGATC

>151 bp product from linear template CP067397.1:c2974327-2973320 Salmonella enterica subsp. enterica serovar Typhimurium strain LB5000 chromosome, complete genome, base 802 to base 952 (Forward invj - Reverse invj).

CAGCGCTGGGGAAATGACTATTCCGTCAATATTCAGGCGCGGCAAGCAGGGGAGTTTTCG

TTAATACCGTCAAATACGCAGGTTGAACATCGTTTGCATGATCAATGGCAAAACGGTAAT

CCCCAGCGCTGGCACCTGACGCGAGACGATC

>151 bp product from linear template CP149257.1:c3761621-3760614 Salmonella enterica subsp. enterica serovar 4,[5],12:i:- strain Z1322HSL0042 chromosome, complete genome, base 802 to base 952 (Forward invj - Reverse invj).

CAGCGCTGGGGAAATGACTATTCCGTCAATATTCAGGCGCGGCAAGCAGGGGAGTTTTCG

TTAATACCGTCAAATACGCAGGTTGAACATCGTTTGCATGATCAATGGCAAAACGGTAAT

CCCCAGCGCTGGCACCTGACGCGAGACGATC

>151 bp product from linear template CP149303.1:1008077-1009084 Salmonella enterica subsp. enterica serovar Typhimurium strain Z1323SSL0045 chromosome, complete genome, base 802 to base 952 (Forward invj - Reverse invj).

CAGCGCTGGGGAAATGACTATTCCGTCAATATTCAGGCGCGGCAAGCAGGGGAGTTTTCG

TTAATACCGTCAAATACGCAGGTTGAACATCGTTTGCATGATCAATGGCAAAACGGTAAT

CCCCAGCGCTGGCACCTGACGCGAGACGATC

>151 bp product from linear template CP104484.1:1039797-1040804 Salmonella enterica strain SalSpp_sample_07_No.3 chromosome, complete genome, base 802 to base 952 (Forward invj - Reverse invj).

CAGCGCTGGGGAAATGACTATTCCGTCAATATTCAGGCGCGGCAAGCAGGGGAGTTTTCG

TTAATACCGTCAAATACGCAGGTTGAACATCGTTTGCATGATCAATGGCAAAACGGTAAT

CCCCAGCGCTGGCACCTGACGCGAGACGATC

>151 bp product from linear template CP074312.1:999716-1000723 Salmonella enterica subsp. enterica strain CFSAN004079 chromosome, complete genome, base 802 to base 952 (Forward invj - Reverse invj).

CAGCGCTGGGGAAATGACTATTCCGTCAATATTCAGGCGCGGCAAGCAGGGGAGTTTTCG

TTAATACCGTCAAATACGCAGGTTGAACATCGTTTGCATGATCAATGGCAAAACGGTAAT

CCCCAGCGCTGGCACCTGACGCGAGACGATC

>151 bp product from linear template CP039860.1:c3023890-3022883 Salmonella enterica subsp. enterica serovar 1,4,[5],12:i:- strain PNCS014880 chromosome, complete genome, base 802 to base 952 (Forward invj - Reverse invj).

CAGCGCTGGGGAAATGACTATTCCGTCAATATTCAGGCGCGGCAAGCAGGGGAGTTTTCG

TTAATACCGTCAAATACGCAGGTTGAACATCGTTTGCATGATCAATGGCAAAACGGTAAT

CCCCAGCGCTGGCACCTGACGCGAGACGATC

>151 bp product from linear template CP116042.1:1039700-1040707 Salmonella enterica subsp. enterica serovar 1,4,[5],12:i:- strain BL708 chromosome, complete genome, base 802 to base 952 (Forward invj - Reverse invj).

CAGCGCTGGGGAAATGACTATTCCGTCAATATTCAGGCGCGGCAAGCAGGGGAGTTTTCG

TTAATACCGTCAAATACGCAGGTTGAACATCGTTTGCATGATCAATGGCAAAACGGTAAT

CCCCAGCGCTGGCACCTGACGCGAGACGATC

>151 bp product from linear template CP091547.1:c3703301-3702294 Salmonella enterica strain 1559 chromosome, complete genome, base 802 to base 952 (Forward invj - Reverse invj).

CAGCGCTGGGGAAATGACTATTCCGTCAATATTCAGGCGCGGCAAGCAGGGGAGTTTTCG

TTAATACCGTCAAATACGCAGGTTGAACATCGTTTGCATGATCAATGGCAAAACGGTAAT

CCCCAGCGCTGGCACCTGACGCGAGACGATC

>151 bp product from linear template CP028318.1:1007452-1008459 Salmonella enterica subsp. enterica serovar Typhimurium var. 5- strain CFSAN067216 chromosome, complete genome, base 802 to base 952 (Forward invj - Reverse invj).

CAGCGCTGGGGAAATGACTATTCCGTCAATATTCAGGCGCGGCAAGCAGGGGAGTTTTCG

TTAATACCGTCAAATACGCAGGTTGAACATCGTTTGCATGATCAATGGCAAAACGGTAAT

CCCCAGCGCTGGCACCTGACGCGAGACGATC

>151 bp product from linear template CP149179.1:1039717-1040724 Salmonella enterica subsp. enterica serovar 4,[5],12:i:- strain Z1323SSL0050 chromosome, complete genome, base 802 to base 952 (Forward invj - Reverse invj).

CAGCGCTGGGGAAATGACTATTCCGTCAATATTCAGGCGCGGCAAGCAGGGGAGTTTTCG

TTAATACCGTCAAATACGCAGGTTGAACATCGTTTGCATGATCAATGGCAAAACGGTAAT

CCCCAGCGCTGGCACCTGACGCGAGACGATC

>151 bp product from linear template CP020922.1:2778781-2779788 Salmonella enterica subsp. enterica strain 16A242, complete genome, base 802 to base 952 (Forward invj - Reverse invj).

CAGCGCTGGGGAAATGACTATTCCGTCAATATTCAGGCGCGGCAAGCAGGGGAGTTTTCG

TTAATACCGTCAAATACGCAGGTTGAACATCGTTTGCATGATCAATGGCAAAACGGTAAT

CCCCAGCGCTGGCACCTGACGCGAGACGATC

>151 bp product from linear template CP009102.1:c2972864-2971857 Salmonella enterica subsp. enterica serovar Typhimurium strain ATCC 13311, complete genome, base 802 to base 952 (Forward invj - Reverse invj).

CAGCGCTGGGGAAATGACTATTCCGTCAATATTCAGGCGCGGCAAGCAGGGGAGTTTTCG

TTAATACCGTCAAATACGCAGGTTGAACATCGTTTGCATGATCAATGGCAAAACGGTAAT

CCCCAGCGCTGGCACCTGACGCGAGACGATC

>151 bp product from linear template CP064263.1:c2995338-2994331 Salmonella enterica subsp. enterica serovar Typhimurium strain LT7 chromosome, complete genome, base 802 to base 952 (Forward invj - Reverse invj).

CAGCGCTGGGGAAATGACTATTCCGTCAATATTCAGGCGCGGCAAGCAGGGGAGTTTTCG

TTAATACCGTCAAATACGCAGGTTGAACATCGTTTGCATGATCAATGGCAAAACGGTAAT

CCCCAGCGCTGGCACCTGACGCGAGACGATC

>151 bp product from linear template CP149378.1:c4020555-4019548 Salmonella enterica subsp. enterica serovar Typhimurium strain Z1323HSL0027 chromosome, complete genome, base 802 to base 952 (Forward invj - Reverse invj).

CAGCGCTGGGGAAATGACTATTCCGTCAATATTCAGGCGCGGCAAGCAGGGGAGTTTTCG

TTAATACCGTCAAATACGCAGGTTGAACATCGTTTGCATGATCAATGGCAAAACGGTAAT

CCCCAGCGCTGGCACCTGACGCGAGACGATC

>151 bp product from linear template CP160067.1:1008580-1009587 Mutant Salmonella enterica subsp. enterica serovar Typhimurium isolate FELIX_MS479 chromosome, complete genome, base 802 to base 952 (Forward invj - Reverse invj).

CAGCGCTGGGGAAATGACTATTCCGTCAATATTCAGGCGCGGCAAGCAGGGGAGTTTTCG

TTAATACCGTCAAATACGCAGGTTGAACATCGTTTGCATGATCAATGGCAAAACGGTAAT

CCCCAGCGCTGGCACCTGACGCGAGACGATC

>151 bp product from linear template CP053865.1:c3060054-3059047 Salmonella enterica subsp. enterica serovar Typhimurium strain SL7207 chromosome, complete genome, base 802 to base 952 (Forward invj - Reverse invj).

CAGCGCTGGGGAAATGACTATTCCGTCAATATTCAGGCGCGGCAAGCAGGGGAGTTTTCG

TTAATACCGTCAAATACGCAGGTTGAACATCGTTTGCATGATCAATGGCAAAACGGTAAT

CCCCAGCGCTGGCACCTGACGCGAGACGATC

>151 bp product from linear template CP064385.1:c1781884-1780877 Salmonella enterica subsp. enterica strain PartC-Senterica-RM8376 chromosome, complete genome, base 802 to base 952 (Forward invj - Reverse invj).

CAGCGCTGGGGAAATGACTATTCCGTCAATATTCAGGCGCGGCAAGCAGGGGAGTTTTCG

TTAATACCGTCAAATACGCAGGTTGAACATCGTTTGCATGATCAATGGCAAAACGGTAAT

CCCCAGCGCTGGCACCTGACGCGAGACGATC

>151 bp product from linear template CP091554.1:c3623217-3622210 Salmonella enterica strain 751 chromosome, complete genome, base 802 to base 952 (Forward invj - Reverse invj).

CAGCGCTGGGGAAATGACTATTCCGTCAATATTCAGGCGCGGCAAGCAGGGGAGTTTTCG

TTAATACCGTCAAATACGCAGGTTGAACATCGTTTGCATGATCAATGGCAAAACGGTAAT

CCCCAGCGCTGGCACCTGACGCGAGACGATC

>151 bp product from linear template CP082696.1:1079736-1080743 Salmonella enterica subsp. enterica serovar 4,[5],12:i:- strain CVM N18S0173 chromosome, complete genome, base 802 to base 952 (Forward invj - Reverse invj).

CAGCGCTGGGGAAATGACTATTCCGTCAATATTCAGGCGCGGCAAGCAGGGGAGTTTTCG

TTAATACCGTCAAATACGCAGGTTGAACATCGTTTGCATGATCAATGGCAAAACGGTAAT

CCCCAGCGCTGGCACCTGACGCGAGACGATC

>151 bp product from linear template CP082647.1:1010286-1011293 Salmonella enterica subsp. enterica serovar Typhimurium var. 5- strain CVM N16S189 chromosome, complete genome, base 802 to base 952 (Forward invj - Reverse invj).

CAGCGCTGGGGAAATGACTATTCCGTCAATATTCAGGCGCGGCAAGCAGGGGAGTTTTCG

TTAATACCGTCAAATACGCAGGTTGAACATCGTTTGCATGATCAATGGCAAAACGGTAAT

CCCCAGCGCTGGCACCTGACGCGAGACGATC

>151 bp product from linear template CP039564.1:c3023978-3022971 Salmonella enterica subsp. enterica serovar 1,4,[5],12:i:- strain PNCS014848 chromosome, complete genome, base 802 to base 952 (Forward invj - Reverse invj).

CAGCGCTGGGGAAATGACTATTCCGTCAATATTCAGGCGCGGCAAGCAGGGGAGTTTTCG

TTAATACCGTCAAATACGCAGGTTGAACATCGTTTGCATGATCAATGGCAAAACGGTAAT

CCCCAGCGCTGGCACCTGACGCGAGACGATC

>151 bp product from linear template CP041005.1:877782-878789 Salmonella enterica strain FDAARGOS_768 chromosome, complete genome, base 802 to base 952 (Forward invj - Reverse invj).

CAGCGCTGGGGAAATGACTATTCCGTCAATATTCAGGCGCGGCAAGCAGGGGAGTTTTCG

TTAATACCGTCAAATACGCAGGTTGAACATCGTTTGCATGATCAATGGCAAAACGGTAAT

CCCCAGCGCTGGCACCTGACGCGAGACGATC

>151 bp product from linear template CP047555.1:c128187-127180 Salmonella enterica subsp. enterica serovar Typhimurium strain SJTUF10112 chromosome, complete genome, base 802 to base 952 (Forward invj - Reverse invj).

CAGCGCTGGGGAAATGACTATTCCGTCAATATTCAGGCGCGGCAAGCAGGGGAGTTTTCG

TTAATACCGTCAAATACGCAGGTTGAACATCGTTTGCATGATCAATGGCAAAACGGTAAT

CCCCAGCGCTGGCACCTGACGCGAGACGATC

>151 bp product from linear template OU943321.1:513971-514978 Salmonella enterica subsp. enterica serovar Typhimurium strain AB-243 genome assembly, chromosome: 1, base 802 to base 952 (Forward invj - Reverse invj).

CAGCGCTGGGGAAATGACTATTCCGTCAATATTCAGGCGCGGCAAGCAGGGGAGTTTTCG

TTAATACCGTCAAATACGCAGGTTGAACATCGTTTGCATGATCAATGGCAAAACGGTAAT

CCCCAGCGCTGGCACCTGACGCGAGACGATC

>151 bp product from linear template CP149252.1:3020172-3021179 Salmonella enterica subsp. enterica serovar 4,[5],12:i:- strain Z1322HSL0047 chromosome, complete genome, base 802 to base 952 (Forward invj - Reverse invj).

CAGCGCTGGGGAAATGACTATTCCGTCAATATTCAGGCGCGGCAAGCAGGGGAGTTTTCG

TTAATACCGTCAAATACGCAGGTTGAACATCGTTTGCATGATCAATGGCAAAACGGTAAT

CCCCAGCGCTGGCACCTGACGCGAGACGATC

>151 bp product from linear template CP082417.1:c3931909-3930902 Salmonella enterica subsp. enterica serovar 4,[5],12:i:- strain FSIS11704067 chromosome, complete genome, base 802 to base 952 (Forward invj - Reverse invj).

CAGCGCTGGGGAAATGACTATTCCGTCAATATTCAGGCGCGGCAAGCAGGGGAGTTTTCG

TTAATACCGTCAAATACGCAGGTTGAACATCGTTTGCATGATCAATGGCAAAACGGTAAT

CCCCAGCGCTGGCACCTGACGCGAGACGATC

>151 bp product from linear template CP123659.1:1047670-1048677 Salmonella enterica strain 20SD07PC06-S1 chromosome, complete genome, base 802 to base 952 (Forward invj - Reverse invj).

CAGCGCTGGGGAAATGACTATTCCGTCAATATTCAGGCGCGGCAAGCAGGGGAGTTTTCG

TTAATACCGTCAAATACGCAGGTTGAACATCGTTTGCATGATCAATGGCAAAACGGTAAT

CCCCAGCGCTGGCACCTGACGCGAGACGATC

>151 bp product from linear template CP065639.1:1577767-1578774 Salmonella enterica strain FDAARGOS_928 chromosome, complete genome, base 802 to base 952 (Forward invj - Reverse invj).

CAGCGCTGGGGAAATGACTATTCCGTCAATATTCAGGCGCGGCAAGCAGGGGAGTTTTCG

TTAATACCGTCAAATACGCAGGTTGAACATCGTTTGCATGATCAATGGCAAAACGGTAAT

CCCCAGCGCTGGCACCTGACGCGAGACGATC

>151 bp product from linear template CP039588.1:2911792-2912799 Salmonella enterica subsp. enterica serovar 1,4,[5],12:i:- strain PNCS014861 chromosome, complete genome, base 802 to base 952 (Forward invj - Reverse invj).

CAGCGCTGGGGAAATGACTATTCCGTCAATATTCAGGCGCGGCAAGCAGGGGAGTTTTCG

TTAATACCGTCAAATACGCAGGTTGAACATCGTTTGCATGATCAATGGCAAAACGGTAAT

CCCCAGCGCTGGCACCTGACGCGAGACGATC

>151 bp product from linear template CP123676.1:c3920029-3919022 Salmonella enterica subsp. enterica serovar 4,[5],12:i:- strain FSIS11922684 chromosome, complete genome, base 802 to base 952 (Forward invj - Reverse invj).

CAGCGCTGGGGAAATGACTATTCCGTCAATATTCAGGCGCGGCAAGCAGGGGAGTTTTCG

TTAATACCGTCAAATACGCAGGTTGAACATCGTTTGCATGATCAATGGCAAAACGGTAAT

CCCCAGCGCTGGCACCTGACGCGAGACGATC

>151 bp product from linear template CP074607.1:1008439-1009446 Salmonella enterica subsp. enterica serovar Typhimurium str. CFSAN000648 strain SGSC 2189 isolate SARA9 chromosome, complete genome, base 802 to base 952 (Forward invj - Reverse invj).

CAGCGCTGGGGAAATGACTATTCCGTCAATATTCAGGCGCGGCAAGCAGGGGAGTTTTCG

TTAATACCGTCAAATACGCAGGTTGAACATCGTTTGCATGATCAATGGCAAAACGGTAAT

CCCCAGCGCTGGCACCTGACGCGAGACGATC

>151 bp product from linear template CP149180.1:c120224-119217 Salmonella enterica subsp. enterica serovar 4,[5],12:i:- strain Z1323SSL0049 chromosome, complete genome, base 802 to base 952 (Forward invj - Reverse invj).

CAGCGCTGGGGAAATGACTATTCCGTCAATATTCAGGCGCGGCAAGCAGGGGAGTTTTCG

TTAATACCGTCAAATACGCAGGTTGAACATCGTTTGCATGATCAATGGCAAAACGGTAAT

CCCCAGCGCTGGCACCTGACGCGAGACGATC

>151 bp product from linear template CP149212.1:c3704744-3703737 Salmonella enterica subsp. enterica serovar 4,[5],12:i:- strain Z1323HSL0047 chromosome, complete genome, base 802 to base 952 (Forward invj - Reverse invj).

CAGCGCTGGGGAAATGACTATTCCGTCAATATTCAGGCGCGGCAAGCAGGGGAGTTTTCG

TTAATACCGTCAAATACGCAGGTTGAACATCGTTTGCATGATCAATGGCAAAACGGTAAT

CCCCAGCGCTGGCACCTGACGCGAGACGATC
